# Supplementary material for: Biomimetic approach to the catalytic enantioselective synthesis of tetracyclic isochroman
Source: Nat Commun. 2021 Aug 16;12:4958. doi: 10.1038/s41467-021-25198-y (PMC8368254; doi:10.1038/s41467-021-25198-y)
Supplement: Supplementary file 1 — Supplementary Information [file 41467_2021_25198_MOESM1_ESM.pdf]

Supplementary Information

Biomimetic Approach to the Catalytic Enantioselective Synthesis of Tetracyclic Isochroman

Xiangfeng Lin, Xianghui Liu, Kai Wang, Qian Li, Yan Liu,* and Can Li*

Contents

1. General information ……………………………………………………………………………………….S2
2. Experimental procedure ……………………………………………………………………………...S3
3. Carbon Isotope Effects (R/R_0) calculated for **3a**..S4
4. Some condition screening of asymmetric cascade reaction...S5
5. Data of products ……………………………………………………………………….…………..S7
6. X-ray crystallographic data of **4l** ………………………………………………………….………...S23
7. ^{1}H NMR and ^{13}C NMR spectra ………………………………………………………………..……S35
8. HPLC spectra ……………………………………………………………………………………..…S72
9. HRMS (ESI) result……………………………………………………………………...………S109
10. Supplementary References………………….………………………………………...……..…S128

1. General Information

Reactions were monitored by thin layer chromatography (TLC), and column chromatography purifications were carried out using silica gel. ^{1}H and ^{13}C spectra were recorded on a 400 MHz spectrometer (100 MHz for ^{13}C) or 700 MHz spectrometer (176 MHz for ^{13}C). The following abbreviations were used to designate chemical shift multiplicities: s = singlet, d = doublet, t = triplet, q = quartert, m = multiplet, br = broad. All first-order splitting patterns were assigned on the basis of the appearance of the multiplet. Splitting patterns that could not be easily interpreted were designated as multiplet (m) or broad (br). Column chromatography was performed on silica gel (300-400 mesh). HPLC analysis was performed on Agilent HPLC 1100 equipped with Daicel chiral AD-H column. High resolution mass spectra for all the new compounds were done by an LTQ-Orbitrap instrument (ESI) (Thermo Fisher Scientific, USA). Catalysts were purchased from J&K Scientific (China) Co., LTD. Substrates **2** were synthesized by following the published procedures.[1] All α-propargyl benzyl alcohols and derivatives were synthesized by following the published procedures.[2,3]

2. Experimental Procedure

General Experimental Procedure of Aymmetric Cascade Reaction

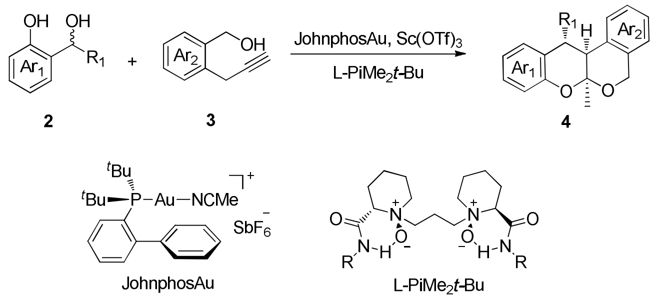

To a 10-mL test-tube were sequentially added JohnphosAu (0.010 mmol, 7.7 mg), $Sc(OTf)_3$ (0.020 mmol, 9.8 mg), L-PiMe$_2$*t*-Bu (0.022 mmol, 14.4 mg) and CH_2Cl_2 (2.0 mL). The mixture stirred for 15 min. α-Propargyl benzyl alcohol 3 (0.3 mmol) and substrate 2 (0.2 mmol) were added in turn to the solution in 6 °C. The reaction mixture was monitored by TLC. Upon completion, the residual was purified by silica gel flash chromatography (petroleum ether:ethyl acetate, 20:1) to afford the desired product **4**. The racemic examples were prepared by the catalysis of $Sc(OTf)_3$ in r.t..

Experimental Procedure for Synthesis of 6

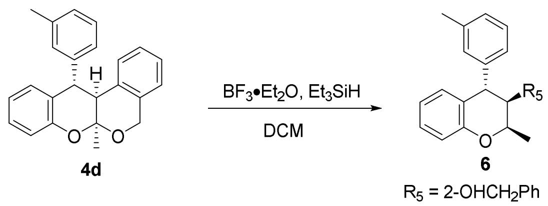

Under Ar atmosphere, to a 10-mL test-tube were sequentially added **4d** (0.4 mmol, 136.8 mg) (89 % *ee*), CH_2Cl_2 (2.0 mL), Et_3SiH (0.4 mmol, 46.4 mg) and $BF_3{\cdot}Et_2O$ (0.2 mL) in 0°C. Upon completion (2 h) the residual was purified by silica gel flash chromatography (petroleum ether:ethyl acetate, 5:1) to afford the desired product **6**.

Experimental Procedure for Synthesis of 7

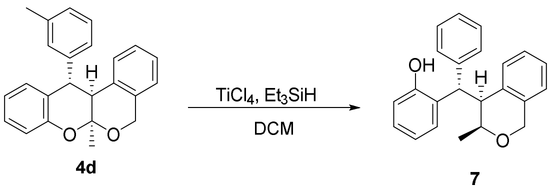

Under Ar atmosphere, to a 10-mL test-tube were sequentially added **4d** (0.2 mmol, 68.4 mg) (89 % *ee*), CH_2Cl_2 (2.0 mL), Et_3SiH (0.2 mmol, 23.2 mg) and $TiCl_4$ (0.1 mL) in 0°C. Upon completion (2 h) the residual was purified by silica gel flash chromatography (petroleum ether:ethyl acetate, 5:1) to afford the desired product **7**.

Experimental Procedure for Carbon Isotope Effects (R/R$_0$)

Experimental Procedure for Carbon isotope effects (R/R$_0$) was promoted via a modification of a procedure reported by Deng's group.[4] To a 10-mL test-tube were sequentially added JohnphosAu (0.05 mmol, 38.5 mg), $Sc(OTf)_3$ (0.10 mmol, 49 mg), L-PiMe$_2$*t*-Bu (0.11 mmol, 65 mg) and CH_2Cl_2 (2.0 mL). The mixture stirred for 15 min. α-Propargyl benzyl alcohol **3a** (1 mmol, 214 mg) and substrate **2b** (1 mmol, 146 mg) were added in turn to the solution in 6°C. The conversion of the reaction was detected by crude ^{1}H-NMR. The residual was purified by silica gel flash chromatography (petroleum ether:ethyl acetate, 20:1) to afford the desired 3-methyl-1H-isochromene **10** when the conversion of the reaction was about 70-77%. The experiment was repeated three times.

The ^{13}C analysis of the virgin and recovered samples of 3-methyl-1H-isochromene **10** was performed following Singleton's method at natural abundance.[5] The NMR(700M) samples were prepared in an identical fashion by

weighing out 40 mg of **10** in a NMR tube and adding 0.5 mL $CDCl_3$. All NMR data were summarized below in Supplementary Table 1

3. Supplementary Table 1. Carbon Isotope Effects (R/R_0) Calculated for 3a. The carbon valued in bold was taken as the internal standard.

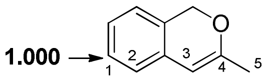

7

Run 1

Entry	Virgin	Recovered (71 %)	R/R_0	Change
1	1.000	1.000	1.000	0.000
2	0.986	1.012	1.026	0.026
3	1.008	1.002	0.994	0.006
4	0.393	0.348	0.885	0.115
5	1.000	0.964	0.964	0.036

Run 2

Entry	Virgin	Recovered (70 %)	R/R_0	Change
1	1.000	1.000	1.000	0.000
2	0.986	1.017	1.032	0.032
3	1.008	1.004	0.998	0.002
4	0.393	0.358	0.910	0.090
5	1.000	0.963	0.963	0.037

Run 3

Entry	Virgin	Recovered (76 %)	R/R_0	Change
1	1.000	1.000	1.000	0.000
2	0.986	1.011	1.025	0.025
3	1.008	1.004	0.998	0.002
4	0.393	0.354	0.900	0.100
5	1.000	0.968	0.968	0.032

4. **Supplementary Table 2. Some Condition Screening of Asymmetric Cascade Reaction**

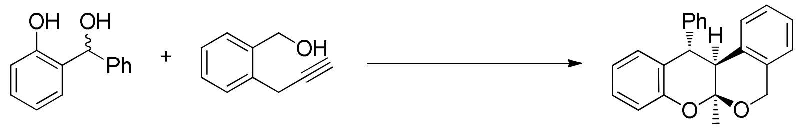

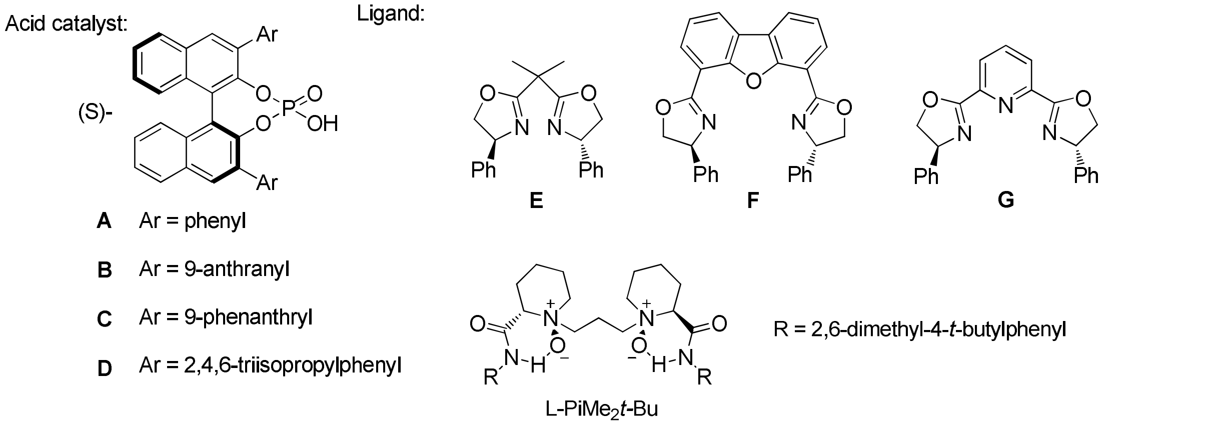

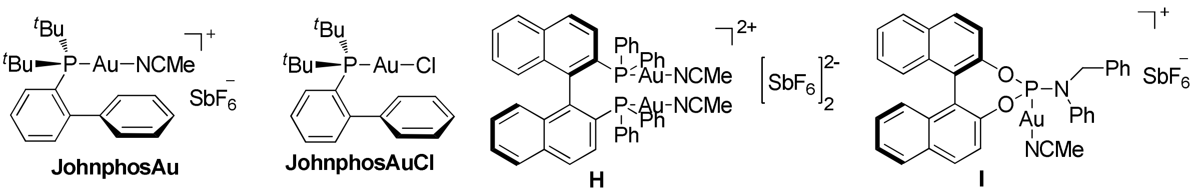

Entry	Acid catalyst	Au-complex	Yield[b]	*Ee*/% (major)[c]	d.r. [d]
1	**A**	JohnphosAu	70	0	1:1
2	**B**	JohnphosAu	76	0	1:1
3	**C**	JohnphosAu	68	0	1:1
4	**D**	JohnphosAu	60	0	1:1
5	$Sc(OTf)_3$+E	JohnphosAu	78	0	1:1
6	$Sc(OTf)_3$+F	JohnphosAu	70	0	1:1
7	$Sc(OTf)_3$+G	JohnphosAu	60	0	1:1
8	$Sc(OTf)_3$+L-PiMe$_2$*t*-Bu	JohnphosAuCl	N.R.	-	-
9	$Sc(OTf)_3$+L-PiMe$_2$*t*-Bu	JohnphosAuCl+AgOTf	N.R.	-	-
10	$Sc(OTf)_3$+L-PiMe$_2$*t*-Bu	JohnphosAuCl+$AgSbF_6$	N.R.	-	-
11[e]	$Sc(OTf)_3$+L-PiMe$_2$*t*-Bu	**H**	67	67	9:1
12[e]	$Sc(OTf)_3$+L-PiMe$_2$*t*-Bu	**I**	60	85	12:1

[a] All reactions were carried out on a 0.1 mmol scale with 1 eq precursor of *o*-QMs **2a**, 1.2 eq **3a**, 10 mol % acid catalyst and 5 mol % of Au-complex in DCM (1 mL) at room temperature. [b] Isolated yield. [c] Determined by chiral HPLC. [d] Determined by by crude ^{1}H-NMR. [e] Reaction temperature: 6 ^{o}C.

5. **Data of Products**

(6a*S*,12*S*,12a*S*)-6a-methyl-12-phenyl-6a,12a-dihydro-5H,12H-isochromeno[3,4-b]chromene

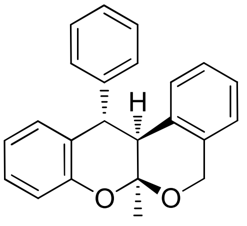

4a

White solid, yield 65%, **m.p.** 140-146°C, 9:1 d.r., ^{1}H-NMR (400 MHz, $CDCl_3$) δ 7.30-7.24 (m, 3H), 7.18-7.12 (m, 2H), 7.03 (d, *J* = 7.6 Hz, 1H), 7.00-6.94 (m, 3H), 6.86 (t, *J* = 7.6 Hz, 1H), 6.79 (t, *J* = 7.2 Hz, 1H), 6.64 (d, *J* = 7.6 Hz, 1H), 6.14 (d, *J* = 7.6 Hz, 1H), 5.22 (d, *J* = 15.8 Hz, 1H), 5.06 (d, *J* = 15.8 Hz, 1H), 3.95 (d, *J* = 10.6 Hz, 1H), 3.08 (d, *J* = 10.6 Hz, 1H), 1.57 (s, 3H).; ^{13}C-NMR (101 MHz, $CDCl_3$) δ 151.7, 142.4, 135.9, 131.7, 130.2, 129.6, 129.5, 128.3, 127.9, 126.9, 125.6, 124.7, 123.8, 121.3, 116.6, 98.5, 66.1, 48.0, 47.5, 23.2.

Enantiometric excess of the product was determined by chiral stationary phase HPLC analysis using Daicel AD-H column (*n*-Hexane/*i*-PrOH 97:3 at 0.6 ml/min, λ = 230 nm, t_{minor} = 7.8 min, t_{major} = 13.3 min, 91% *ee*, $[\alpha]_D^{20}$ = -10.18 (*c* = 0.35, in $CHCl_3$); HRMS (ESI) *m/z* calcd for $C_{23}H_{20}O_2$, $[M+Na]^+$: 351.1356, found: 351.1356.

(6a*S*,12*S*,12a*S*)-6a-methyl-12-(*p*-tolyl)-6a,12a-dihydro-5H,12H-isochromeno[3,4-b]chromene

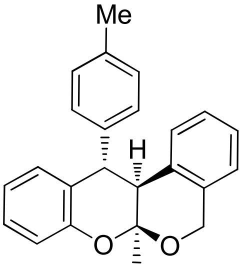

4b

White solid, yield 65%, **m.p.** 150-154°C, 14:1 d.r., ^{1}H-NMR (700 MHz, $CDCl_3$) δ 7.19 – 7.12 (m, 2H), 7.08 (d, *J* = 7.8 Hz, 2H), 7.04 (d, *J* = 7.6 Hz, 1H), 6.98 (dd, *J* = 8.2, 0.8 Hz, 1H), 6.89 (t, *J* = 7.4 Hz, 1H), 6.86 (d, *J* = 7.9 Hz, 2H), 6.82 – 6.77 (m, 1H), 6.66 (d, *J* = 7.7 Hz, 1H), 6.20 (d, *J* = 7.6 Hz, 1H), 5.23 (d, *J* = 15.7 Hz, 1H), 5.06 (d, *J* = 15.7 Hz, 1H), 3.92 (d, *J* = 10.6 Hz, 1H), 3.07 (d, *J* = 10.6 Hz, 1H), 2.36 (s, 3H), 1.58 (s, 3H).; ^{13}C-NMR (101 MHz, $CDCl_3$) δ 150.6, 138.2, 135.3, 135.0, 130.6, 129.0, 128.7, 128.5, 127.9, 126.7, 125.8, 124.5, 123.9, 122.7, 120.2, 115.5, 97.5, 65.0, 46.5, 46.4, 22.2, 20.1.

Enantiometric excess of the product was determined by chiral stationary phase HPLC analysis using Daicel AD-H column (*n*-Hexane/*i*-PrOH 97:3 at 0.6 ml/min, λ = 230 nm, t_{minor} = 8.3min, t_{major} = 13.5 min, 88% *ee*, $[\alpha]_D^{20}$ = -16.57 (*c* = 0.35, in $CHCl_3$); HRMS (ESI) *m/z* calcd for $C_{24}H_{22}O_2$, $[M+Na]^+$: 365.1512, found: 365.1511.

(6a*S*,12*S*,12a*S*)-12-(4-ethylphenyl)-6a-methyl-6a,12a-dihydro-5H,12H-isochromeno[3,4-b]chromene

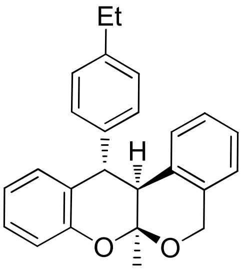

4c

White solid, yield 68%, **m.p.** 164-167°C, 14:1 d.r., ^{1}H-NMR (700 MHz, $CDCl_3$) δ 7.17 – 7.11 (m, 2H), 7.08 (d, *J* = 8.0 Hz, 2H), 7.02 (d, *J* = 7.6 Hz, 1H), 6.97 (dd, *J* = 8.2, 1.0 Hz, 1H), 6.87 (dd, *J* = 7.0, 5.0 Hz, 3H), 6.79 (td, *J* = 7.6, 1.1 Hz, 1H), 6.67 (d, *J* = 7.7 Hz, 1H), 6.17 (d, *J* = 7.6 Hz, 1H), 5.21 (d, *J* = 15.7 Hz, 1H), 5.05 (d, *J* = 15.7 Hz, 1H), 3.92 (d, *J* = 10.6 Hz, 1H), 3.06 (d, *J* = 10.6 Hz, 1H), 2.65 (q, *J* = 7.6 Hz, 2H), 1.56 (s, 4H), 1.25 (t, *J* = 7.6 Hz, 3H).; ^{13}C-NMR (176 MHz, $CDCl_3$) δ 151.6, 142.7, 139.4, 136.0, 131.7, 130.0, 129.7, 129.6, 127.8, 127.7, 126.8, 125.5, 124.9, 123.7, 121.2, 116.5, 98.5, 66.0, 47.5, 47.5, 29.7, 28.5, 23.3, 15.5.
Enantiometric excess of the product was determined by chiral stationary phase HPLC analysis using Daicel AD-H column (*n*-Hexane/*i*-PrOH 97:3 at 0.6 ml/min, λ = 230 nm, t_{minor} = 7.9 min, t_{major} = 12.4 min, 90% *ee*, $[\alpha]_D^{20}$ = 12.73 (*c* = 0.33, in $CHCl_3$); HRMS (ESI) m/z calcd for $C_{25}H_{24}O_2$, $[M+Na]^+$: 379.1669, found: 379.1667.

(6a*S*,12*S*,12a*S*)-6a-methyl-12-(*m*-tolyl)-6a,12a-dihydro-5H,12H-isochromeno[3,4-b]chromene

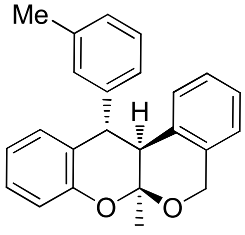

4d

White solid, yield 57%, **m.p.** 87-90°C, >20:1 d.r., ^{1}H-NMR (400 MHz, $CDCl_3$) δ 7.19 – 7.10 (m, 3H), 7.09 – 7.00 (m, 2H), 6.97 (d, *J* = 8.0 Hz, 1H), 6.87 (t, *J* = 7.6 Hz, 1H), 6.82 – 6.73 (m, 3H), 6.67 (d, *J* = 7.6 Hz, 1H), 6.16 (d, *J* = 7.6 Hz, 1H), 5.21 (d, *J* = 15.8 Hz, 1H), 5.05 (d, *J* = 15.8 Hz, 1H), 3.90 (d, *J* = 10.6 Hz, 1H), 3.07 (d, *J* = 10.6 Hz, 1H), 2.27 (s, 3H), 1.57 (s, 3H).; ^{13}C-NMR (101 MHz, $CDCl_3$) δ 151.6, 142.3, 137.8, 136.0, 131.6, 130.8, 129.7, 129.6, 128.1, 127.8, 127.6, 127.4, 126.8, 125.5, 124.8, 123.8, 121.3, 116.5, 98.5, 66.1, 47.9, 47.4, 23.3, 21.4.
Enantiometric excess of the product was determined by chiral stationary phase HPLC analysis using Daicel IA column (*n*-Hexane/*i*-PrOH 98:2 at 0.5 ml/min, λ = 230 nm, t_{minor} = 9.3 min, t_{major} = 13.1 min, 87% *ee*, $[\alpha]_D^{20}$ = 21.5 (*c* = 0.20, in $CHCl_3$); HRMS (ESI) *m/z* calcd for $C_{24}H_{22}O_2$, $[M+Na]^+$: 365.1512, found: 365.1512.

(6a*S*,12*S*,12a*S*)-12-(3-methoxyphenyl)-6a-methyl-6a,12a-dihydro-5H,12H-isochromeno[3,4-b]chromene

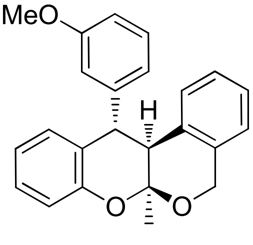

4e

White solid, yield 63%, **m.p.** 54-56°C, 6:1 d.r., ^{1}H-NMR (400 MHz, $CDCl_3$) δ 7.21-7.19 (m, 3H), 7.03 (d, *J* = 7.6 Hz, 1H), 6.97 (d, *J* = 8.0 Hz, 1H), 6.89 (t, *J* = 7.6 Hz, 1H), 6.84-6.76 (m, 2H), 6.68 (d, *J* = 7.6 Hz, 1H), 6.57 (d, *J* = 7.6 Hz, 1H), 6.50 (s, 1H), 6.22 (d, *J* = 8.0 Hz, 1H), 5.22 (d, *J* = 15.8 Hz, 1H), 5.05 (d, *J* = 15.8 Hz, 1H), 3.92 (d, *J* = 10.6 Hz, 1H), 3.72 (s, 3H), 3.08 (d, *J* = 10.6 Hz, 1H), 1.57 (s, 3H).; ^{13}C-NMR (101 MHz, $CDCl_3$) δ 159.5, 151.6, 144.0, 135.9, 131.6, 129.6, 129.5, 129.2, 127.9, 126.9, 125.6, 1 24.4, 123.8, 122.7, 121.3, 116.5, 115.8, 112.3, 98.5, 66.1, 55.2, 48.1, 47.3, 23.2.
Enantiometric excess of the product was determined by chiral stationary phase HPLC analysis using Daicel AD-H column (*n*-Hexane/*i*-PrOH 97:3 at 0.6 ml/min, λ = 230 nm, t_{minor} = 10.2 min, t_{major} = 17.3 min, 89% *ee*, $[\alpha]_D^{20}$ = -7.00 (*c* = 0.20, in $CHCl_3$); HRMS (ESI) *m/z* calcd for $C_{24}H_{22}O_3$, $[M+Na]^+$: 381.1461, found: 381.1468.

(6a*S*,12*S*,12a*S*)-12-(4-chlorophenyl)-6a-methyl-6a,12a-dihydro-5H,12H-isochromeno[3,4-b]chromene

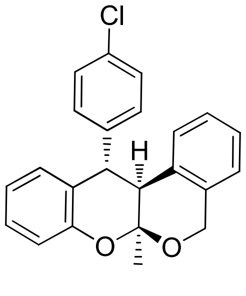

4f

White solid, yield 61%, **m.p.** 95-100°C, 10:1 d.r., ^{1}H-NMR (700 MHz, $CDCl_3$) δ 7.24 (d, *J* = 8.4 Hz, 2H), 7.20-7.13 (m, 2H), 7.04 (d, *J* = 7.6 Hz, 1H), 7.04 (d, *J* = 7.6 Hz, 1H), 6.98 (dd, *J* = 8.2, 0.9 Hz, 1H), 6.93-6.88 (m, 3H), 6.80 (td, *J* = 7.6, 1.1 Hz, 1H), 6.61 (d, *J* = 7.7 Hz, 1H), 6.17 (d, *J* = 7.6 Hz, 1H), 5.22 (d, *J* = 15.8 Hz, 1H), 5.06 (d, *J* = 15.8 Hz, 1H), 3.94 (d, *J* = 10.7 Hz, 1H), 3.01 (d, *J* = 10.7 Hz, 1H), 1.57 (s, 3H).; ^{13}C-NMR (176 MHz, $CDCl_3$) δ 151.6, 141.0, 135.6, 132.7, 131.6, 131.5, 129.6, 129.3, 128.5, 128.1, 127.1, 125.7, 124.1, 123.9, 121.3, 116.7, 98.4, 66.1, 47.5, 47.5, 23.1.

Enantiometric excess of the product was determined by chiral stationary phase HPLC analysis using Daicel AD-H column (*n*-Hexane/*i*-PrOH 97:3 at 0.6 ml/min, λ = 230 nm, t_{minor} = 9.3 min, t_{major} = 16.9 min, 94% *ee*, $[\alpha]_D^{20}$ = 5.5 (*c* = 0.20, in $CHCl_3$); HRMS (ESI) *m/z* calcd for $C_{23}H_{19}O_2Cl$, $[M+Na]^+$: 385.0966, found: 385.0969.

(6a*S*,12*S*,12a*S*)-12-(4-fluorophenyl)-6a-methyl-6a,12a-dihydro-5H,12H-isochromeno[3,4-b]chromene

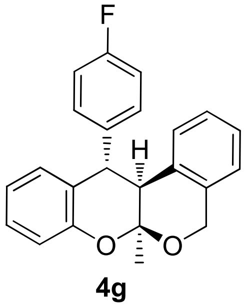

4g

White solid, yield 52%, **m.p.** 111-115°C, 17:1 d.r., ^{1}H-NMR (700 MHz, $CDCl_3$) δ 7.17 (m, 2H), 7.04 (d, *J* = 7.6 Hz, 1H), 7.00-6.94 (m, 3H), 6.94-6.87 (m, 3H), 6.80 (td, *J* = 7.6, 1.0 Hz, 1H), 6.62 (d, *J* = 7.7 Hz, 1H), 6.14 (d, *J* = 7.6 Hz, 1H), 5.22 (d, *J* = 15.7 Hz, 1H), 5.06 (d, *J* = 15.8 Hz, 1H), 3.94 (d, *J* = 10.8 Hz, 1H), 3.00 (d, *J* = 10.8 Hz, 1H), 1.57 (s, 3H).; ^{13}C-NMR (176 MHz, $CDCl_3$) δ 161.9 ($J_{C\text{-}F}$ = 244.64 Hz), 151.6, 138.1, 138.0, 135.7, 131.7, 131.6 ($J_{C\text{-}F}$ = 7.06 Hz), 129.6, 129.3, 128.1, 127.0, 125.7, 124.4, 123.9, 121.3, 116.6, 115.2 ($J_{C\text{-}F}$ = 22.88 Hz), 98.5, 66.1, 47.7, 47.3, 23.1.

Enantiometric excess of the product was determined by chiral stationary phase HPLC analysis using Daicel AD-H column (*n*-Hexane/*i*-PrOH 95:5 at 0.9 ml/min, λ = 230 nm, t_{minor} = 5.5 min, t_{major} = 9.2 min, 91% *ee*, $[\alpha]_D^{20}$ = -26.50 (*c* = 0.20, in $CHCl_3$); HRMS (ESI) *m/z* calcd for $C_{23}H_{19}O_2F$, $[M+Na]^+$: 369.1261, found: 369.1258.

(6a*S*,12*S*,12a*S*)-6a-methyl-12-(naphthalen-2-yl)-6a,12a-dihydro-5H,12H-isochromeno[3,4-b]chromene

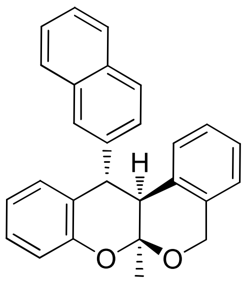

4h

White solid, yield 76%, **m.p.** 120-124°C,11:1 d.r., ^{1}H-NMR (700 MHz, $CDCl_3$) δ 7.84 (d, *J* = 7.7 Hz, 1H), 7.79 (d, *J* = 8.4 Hz, 1H), 7.68 (d, *J* = 7.6 Hz, 1H), 7.49-7.44 (m, 2H), 7.34 (s, 1H), 7.22 – 7.11 (m, 3H), 7.05 (d, *J* = 7.6 Hz, 1H), 7.01 (dd, *J* = 8.2, 1.0 Hz, 1H), 6.80 – 6.70 (m, 2H), 6.63 (d, *J* = 7.7 Hz, 1H), 6.08 (d, *J* = 7.6 Hz, 1H), 5.27 (d, *J* = 15.7 Hz, 1H), 5.10 (d, *J* = 15.7 Hz, 1H), 4.12 (d, *J* = 10.7 Hz, 2H), 3.19 (d, *J* = 10.7 Hz, 1H), 1.61 (s, 3H).; ^{13}C-NMR (176 MHz, $CDCl_3$) δ 151.6, 139.5, 135.9, 133.2, 132.6, 131.6, 129.7, 129.6, 129.6, 128.3, 128.0, 127.7, 127.7, 127.5, 126.9, 126.1, 125.7, 125.6, 124.5, 123.8, 121.3, 116.6, 98.5, 66.2, 48.2, 47.1, 23.2.
Enantiometric excess of the product was determined by chiral stationary phase HPLC analysis using Daicel AD-H column (*n*-Hexane/*i*-PrOH 97:3 at 0.7 ml/min, λ = 230 nm, t_{minor} = 11.1 min, t_{major} = 20.6 min, 90% *ee*, $[\alpha]_D^{20}$ = -65.05 (*c* = 0.33, in $CHCl_3$);HRMS (ESI) *m/z* calcd for $C_{27}H_{22}O_2$, $[M+Na]^+$: 401.1512, found: 401.1511.

(6a*S*,12*S*,12a*S*)-12-([1,1'-biphenyl]-4-yl)-6a-methyl-6a,12a-dihydro-5H,12H-isochromeno[3,4-b]chromene

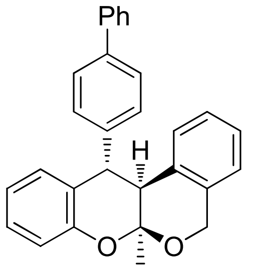

4i

White solid, yield 60%, **m.p.** 153-157°C,13:1 d.r., ^{1}H-NMR (700 MHz, $CDCl_3$) δ 7.63 (d, *J* = 7.1 Hz, 2H), 7.52 (d, *J* = 8.2 Hz, 2H), 7.45 (t, *J* = 7.7 Hz, 2H), 7.35 (t, *J* = 7.4 Hz, 1H), 7.20-7.14 (m, 2H), 7.06 – 7.02 (m, 3H), 6.99 (dd, *J* = 8.2, 0.9 Hz, 1H), 6.88 (t, *J* = 7.4 Hz, 1H), 6.82 (td, *J* = 7.7, 1.0 Hz, 1H), 6.71 (d, *J* = 7.7 Hz, 1H), 6.22 (d, *J* = 7.6 Hz, 1H), 5.24 (d, *J* = 15.7 Hz, 1H), 5.08 (d, *J* = 15.7 Hz, 1H), 4.00 (d, *J* = 10.6 Hz, 1H), 3.11 (d, *J* = 10.6 Hz, 1H), 1.59 (s, 3H).; ^{13}C-NMR (176 MHz, $CDCl_3$) δ 151.7, 141.5, 140.7, 139.6, 135.9, 131.7, 130.6, 129.7, 129.6, 128.8, 128.0, 127.3, 127.0, 126.9, 125.7, 124.6, 123.8, 121.3, 116.6, 98.5, 66.1, 47.7, 47.5, 23.2.
Enantiometric excess of the product was determined by chiral stationary phase HPLC analysis using Daicel AD-H column (*n*-Hexane/*i*-PrOH 97:3 at 0.6 ml/min, λ = 230 nm, t_{major} = 11.9 min, t_{minor} = 24.1 min, 84% *ee*, $[\alpha]_D^{20}$ = 12.00 (*c* = 0.30, in $CHCl_3$); HRMS (ESI) *m/z* calcd for $C_{27}H_{22}O_2$, $[M+Na]^+$: 427.1669, found: 427.1674.

(6a*S*,12*S*,12a*S*)-10-methoxy-6a-methyl-12-phenyl-6a,12a-dihydro-5H,12H-isochromeno[3,4-b]chromene

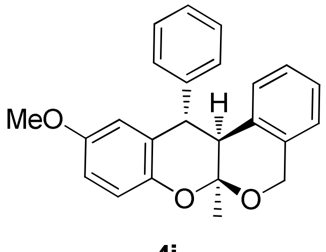

4j

White solid, yield 57%, **m.p.** 96-100°C,17:1 d.r., ^{1}H-NMR (400 MHz, $CDCl_3$) δ 7.30 – 7.22 (m, 3H), 7.15 (t, *J* = 7.2 Hz, 1H), 7.03 (d, *J* = 7.6 Hz, 1H), 7.00 – 6.94 (m, 2H), 6.91 (d, *J* = 8.8 Hz, 1H), 6.85 (t, *J* = 7.6 Hz, 1H), 6.72 (dd, *J* = 8.8, 2.8 Hz, 1H), 6.19 (d, *J* = 2.6 Hz, 1H), 6.14 (d, *J* = 7.6 Hz, 1H), 5.22 (d, *J* = 15.8 Hz, 1H), 5.05 (d, *J* = 15.8 Hz, 1H), 3.91 (d, *J* = 10.6 Hz, 1H), 3.57 (s, 3H), 3.05 (d, *J* = 10.6 Hz, 1H), 1.55 (s, 3H).; ^{13}C-NMR (101 MHz, $CDCl_3$) δ 153.9, 145.8, 142.2, 136.0, 131.7, 130.2, 129.6, 128.3, 126.9, 126.8, 125.6, 125.4, 123.8, 117.1, 114.5, 113.7, 98.4, 66.1, 55.6, 48.3, 47.5, 23.2.
Enantiometric excess of the product was determined by chiral stationary phase HPLC analysis using Daicel AD-H column (*n*-Hexane/*i*-PrOH 97:3 at 0.6 ml/min, λ = 230 nm, t_{major} = 15.9 min, t_{minor} = 22.3 min, 87% *ee*, $[\alpha]_D^{20}$ = -19.00 (*c* = 0.30, in $CHCl_3$); HRMS (ESI) *m/z* calcd for $C_{24}H_{22}O_3$, $[M+Na]^+$: 381.1461, found: 381.1454.

(6a*S*,12*S*,12a*S*)-9-(methoxymethoxy)-6a-methyl-12-phenyl-6a,12a-dihydro-5H,12H-isochromeno[3,4-b]chromene

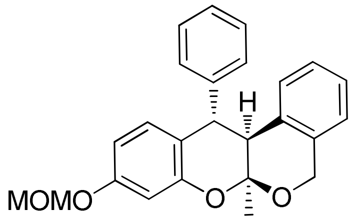

4k

White solid, yield 61%, **m.p.** 80-82°C,6:1 d.r., ^{1}H-NMR (400 MHz, $CDCl_3$) δ 7.30 – 7.22 (m, 4H), 7.15 (t, *J* = 7.6 Hz, 1H), 7.03 (d, *J* = 7.6 Hz, 1H), 6.98 – 6.91 (m, 1H), 6.85 (t, *J* = 7.4 Hz, 1H), 6.71 (d, *J* = 2.2 Hz, 1H), 6.54 (d, *J* = 8.6 Hz, 1H), 6.49 (dd, *J* = 8.4, 2.2 Hz, 1H), 6.13 (d, *J* = 7.6 Hz, 1H), 5.25-5.02 (m, 4H), 3.87 (d, *J* = 10.6 Hz, 1H), 3.46 (s, 1H), 3.05 (s, 3H), 3.03 (d, *J* = 10.6 Hz, 1H), 1.56 (s, 3H).; ^{13}C-NMR (101 MHz, $CDCl_3$) δ 157.0, 152.3, 142.4, 135.9, 131.6, 130.2, 130.1, 129.6, 128.3, 126.8, 125.6, 123.8, 118.2, 110.0, 104.0, 98.7, 94.5, 66.1, 55.9, 47.6, 23.2.

Enantiometric excess of the product was determined by chiral stationary phase HPLC analysis using Daicel AD-H column (*n*-Hexane/*i*-PrOH 97:3 at 0.6 ml/min, λ = 230 nm, t_{minor} = 8.6 min, t_{major} = 12.1 min, 83% *ee*, $[\alpha]_D^{20}$ = -21.50 (*c* = 0.35, in $CHCl_3$); HRMS (ESI) *m/z* calcd for $C_{25}H_{24}O_4$, $[M+Na]^+$: 411.1567, found: 411.1565.

(6a*S*,12*S*,12a*S*)-6a,10-dimethyl-12-phenyl-6a,12a-dihydro-5H,12H-isochromeno[3,4-b]chromene

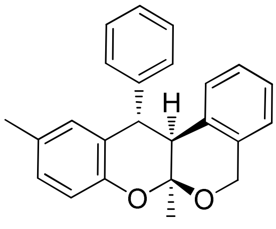

4l

White solid, yield 59%, **m.p.** 162-164°C, 20:1 d.r., ^{1}H-NMR (400 MHz, $CDCl_3$) δ 7.32 – 7.23 (m, 3H), 7.15 (t, *J* = 7.4 Hz, 1H), 7.03 (d, *J* = 7.6 Hz, 1H), 6.99-6.90 (m, 3H), 6.90-6.84 (m, 2H), 6.44 (s, 1H), 6.13 (d, *J* = 7.6 Hz, 1H), 5.21 (d, *J* = 15.8 Hz, 1H), 5.04 (d, *J* = 15.8 Hz, 1H), 3.90 (d, *J* = 10.6 Hz, 1H), 3.05 (d, *J* = 10.6 Hz, 1H), 2.11 (s, 3H), 1.55 (s, 3H).; ^{13}C-NMR (101 MHz, $CDCl_3$) δ 149.5, 142.5, 136.1, 131.7, 130.5, 130.2, 129.7, 129.6, 128.6, 128.3, 126.8, 125.5, 124.3, 123.8, 116.3, 98.4, 66.1, 48.1, 47.7, 23.3, 20.6.

Enantiometric excess of the product was determined by chiral stationary phase HPLC analysis using Daicel AD-H column (*n*-Hexane/*i*-PrOH 97:3 at 0.6 ml/min, λ = 230 nm, t_{minor} = 9.2 min, t_{major} = 14.8 min, 89% *ee*, $[\alpha]_D^{20}$ = -24.81 (*c* = 0.27, in $CHCl_3$); HRMS (ESI) *m/z* calcd for $C_{24}H_{22}O_2$, $[M+Na]^+$: 365.1512, found: 365.1509.

(6a*S*,12*S*,12a*S*)-9-chloro-6a-methyl-12-phenyl-6a,12a-dihydro-5H,12H-isochromeno[3,4-b]chromene

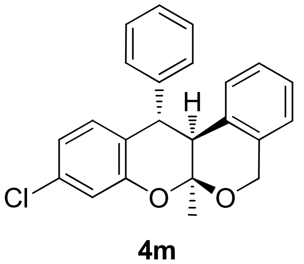

4m

White solid, yield 57%, **m.p.** 167-170°C, 8:1 d.r., ^{1}H-NMR (400 MHz, $CDCl_3$) δ 7.30 – 7.22 (m, 3H), 7.16 (t, *J* = 7.6 Hz, 1H), 7.03 (d, *J* = 7.6 Hz, 1H), 6.99 (d, *J* = 2.0 Hz, 1H), 6.96 – 6.91 (m, 2H), 6.86 (d, *J* = 8.0 Hz,, 2H), 6.77 (dd, *J* = 8.4, 1.8 Hz, 1H), 6.57 (d, *J* = 8.4 Hz, 1H), 6.13 (d, *J* = 7.6 Hz, 1H), 5.22 (d, *J* = 15.8 Hz, 1H), 5.05 (d, *J* =

15.8 Hz, 1H), 3.88 (d, J = 10.6 Hz, 1H), 3.04 (d, J = 10.6 Hz, 1H), 1.56 (s, 3H).; ^{13}C-NMR (101 MHz, $CDCl_3$) δ 152.3, 141.8, 135.5, 133.0, 131.5, 130.6, 130.1, 129.6, 128.4, 127.1, 127.0, 125.7, 123.8, 123.4, 121.5, 116.8, 98.9, 66.1, 47.6, 47.3, 23.1.

Enantiometric excess of the product was determined by chiral stationary phase HPLC analysis using Daicel AD-H column (*n*-Hexane/*i*-PrOH 97:3 at 0.6 ml/min, λ = 230 nm, t_{minor} = 8.7 min, t_{major} = 10.2 min, 82 % *ee*, $[\alpha]_D^{20}$ = -23.22 (c = 0.30, in $CHCl_3$); HRMS (ESI) *m/z* calcd for $C_{23}H_{19}O_2Cl$, $[M+Na]^+$: 385.0966, found: 385.0966.

(6a*S*,12*S*,12a*S*)-10-chloro-6a-methyl-12-phenyl-6a,12a-dihydro-5H,12H-isochromeno[3,4-b]chromene

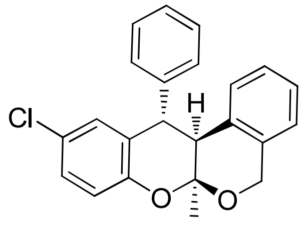

4n

White solid, yield 42%, **m.p.** 94-96°C, 7:1 d.r., ^{1}H-NMR (400 MHz, $CDCl_3$) δ 7.33 – 7.23 (m, 3H), 7.16 (t, J = 7.6 Hz, 1H), 7.10 (dd, J = 8.8, 2.4 Hz, 1H), 7.03 (d, J = 7.6 Hz, 1H), 6.98 – 6.82 (m, 4H), 6.63 (s, 1H), 6.12 (d, J = 7.7 Hz, 1H), 5.21 (d, J = 15.8 Hz, 1H), 5.05 (d, J = 15.8 Hz, 1H), 3.90 (d, J = 10.8 Hz, 1H), 3.04 (d, J = 10.8 Hz, 1H), 1.65 (s, 3H).; ^{13}C-NMR (101 MHz, $CDCl_3$) δ 150.3, 141.5, 135.5, 131.5, 130.1, 129.6, 129.1, 128.5, 127.9, 127.2, 127.0, 126.5, 126.1, 125.7, 123.8, 118.0, 98.7, 66.1, 47.9, 47.2, 23.1.

Enantiometric excess of the product was determined by chiral stationary phase HPLC analysis using Daicel AD-H column (*n*-Hexane/*i*-PrOH 95:5 at 0.9 ml/min, λ = 230 nm, t_{minor} = 5.7 min, t_{major} = 8.1 min, 85 % *ee*, $[\alpha]_D^{20}$ = -18.03 (c = 0.36, in $CHCl_3$); HRMS (ESI) *m/z* calcd for $C_{23}H_{19}O_2Cl$, $[M+Na]^+$: 385.0966, found: 385.0967.

(6a*S*,12*S*,12a*S*)-10-(benzyloxy)-6a-methyl-12-phenyl-6a,12a-dihydro-5H,12H-isochromeno[3,4-b]chromene

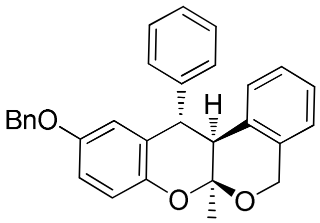

4o

White solid, yield 55%, **m.p.** 82-85°C, 13:1 d.r., ^{1}H-NMR (400 MHz, $CDCl_3$) δ 7.32 – 7.22 (m, 8H), 7.15 (t, J = 7.6 Hz, 1H), 7.03 (d, J = 7.6 Hz, 1H), 6.97 – 6.81 (m, 4H), 6.79 (dd, J = 8.8, 2.8 Hz, 1H), 6.27 (d, J = 2.4 Hz, 1H), 6.13 (d, J = 7.6 Hz, 1H), 5.22 (d, J = 15.8 Hz, 1H), 5.05 (d, J = 15.8 Hz, 1H), 4.89 – 4.67 (m, 2H), 3.90 (d, J = 10.6 Hz, 1H), 3.05 (d, J = 10.6 Hz, 1H), 1.55 (s, 3H).; ^{13}C-NMR (101 MHz, $CDCl_3$) δ 153.1, 145.9, 142.1, 137.1, 136.0, 131.7, 130.2, 129.6, 128.5, 128.3, 127.8, 127.6, 126.9, 126.8, 125.6, 125.4, 123.8, 117.1, 115.7, 114.8, 98.4, 70.5, 66.1, 48.3, 47.5, 23.2.

Enantiometric excess of the product was determined by chiral stationary phase HPLC analysis using Daicel AD-H column (*n*-Hexane/*i*-PrOH 97:3 at 0.6 ml/min, λ = 230 nm, t_{minor} = 18.2 min, t_{major} = 40.9 min, 83% *ee*, $[\alpha]_D^{20}$ = -45.21 (c = 0.23, in $CHCl_3$); HRMS (ESI) *m/z* calcd for $C_{30}H_{26}O_3$, $[M+Na]^+$: 457.1774, found: 457.1778.

(6a*S*,13*R*,13a*S*)-13-cyclohexyl-6a-methyl-6a,13a-dihydro-5H,13H-[1,3]dioxolo[4,5-g]isochromeno[3,4-b]chromene

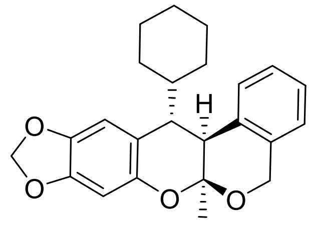

4p

Colorless oil, yield 45%, >20:1 d.r., ^{1}H-NMR (700 MHz, $CDCl_3$) δ 7.23 – 7.16 (m, 3H), 6.99 (d, *J* = 7.3 Hz, 1H), 6.50 (s, 1H), 6.49 (s, 1H), 5.87 (s, 2H), 5.30 (s, 1H), 4.95 (d, *J* = 15.3 Hz, 1H), 4.81 (d, *J* = 15.3 Hz, 1H), 3.22 (d, *J* = 4.4 Hz, 1H), 2.74 (t, *J* = 5.2 Hz, 1H), 1.77 – 1.65 (m, 2H), 1.60 – 1.47 (m, 6H), 1.40 – 1.30 (m, 1H), 1.13 – 0.92 (m, 4H), 0.65 – 0.50 (m, 1H). ^{13}C-NMR (176 MHz, $CDCl_3$) δ 147.7, 146.4, 141.5, 133.9, 133.7, 128.7, 126.9, 126.4, 123.9, 120.0, 107.4, 102.3, 100.8, 99.1, 64.2, 53.4, 47.3, 44.2, 36.7, 34.4, 31.5, 26.9, 26.6, 26.3, 25.7.
Enantiometric excess of the product was determined by chiral stationary phase HPLC analysis using Daicel IC column (*n*-Hexane/*i*-PrOH 95:5 at 1.0 ml/min, λ = 230 nm, t_{major} = 6.7 min, t_{minor} = 8.5 min, 71% *ee*, $[\alpha]_D^{20}$ = -52.78 (*c* = 0.52, in $CHCl_3$); HRMS (ESI) *m/z* calcd for $C_{24}H_{26}O_4$, $[M+Na]^+$: 401.1723, found: 401.1720.

(6aS,13R,13aS)-13-isopropyl-6a-methyl-6a,13a-dihydro-5H,13H-[1,3]dioxolo[4,5-g]isochromeno[3,4-b]chromene

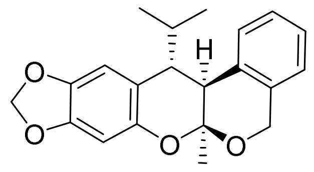

4q

Colorless oil, yield 82%, >20:1 d.r., ^{1}H-NMR (400 MHz, $CDCl_3$) δ 7.30 – 7.14 (m, 3H), 6.99 (d, *J* = 7.2 Hz, 1H), 6.51 (d, *J* = 4.0 Hz, 2H), 5.86 (d, *J* = 2.4 Hz, 2H), 4.96 (d, *J* = 15.6 Hz, 1H), 4.80 (d, *J* = 15.6 Hz, 1H), 3.23 (d, *J* = 4.4 Hz, 1H), 2.73 (t, *J* = 5.4 Hz, 1H), 2.13 – 2.00 (m, 1H), 1.58 (s, 3H), 0.80 – 0.68 (m, 6H). ^{13}C-NMR (101 MHz, $CDCl_3$) δ 147.7, 146.5, 141.6, 133.9, 133.8, 128.7, 127.0, 126.5, 124.0, 120.3, 107.5, 102.4, 100.9, 99.2, 64.1, 48.3, 44.7, 26.9, 25.7, 23.9, 21.3.
Enantiometric excess of the product was determined by chiral stationary phase HPLC analysis using Daicel IC column (*n*-Hexane/*i*-PrOH 97:3 at 0.6 ml/min, λ = 230 nm, t_{major} = 14.0 min, t_{minor} = 18.3 min, 75% *ee*, $[\alpha]_D^{20}$ = -47.20 (*c* = 0.45, in $CHCl_3$); HRMS (ESI) *m/z* calcd for $C_{21}H_{22}O_4$, $[M+Na]^+$: 361.1410, found: 361.1408.

(6a*S*,12*S*,12a*S*)-2-methoxy-6a-methyl-12-(naphthalen-2-yl)-5,6a,12,12a-tetrahydroisochromeno[3,4-b]chromene

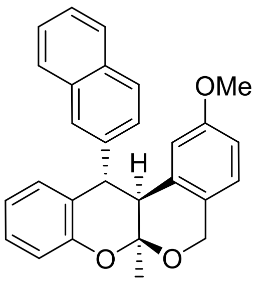

4r

White solid, yield 68 %, **m.p.** 88-91°C, 9:1 d.r., ^{1}H-NMR (400 MHz, $CDCl_3$) δ 7.87 – 7.77 (m, 2H), 7.76 – 7.64 (m, 1H), 7.50 – 7.41 (m, 2H), 7.36 (s, 1H), 7.24 – 7.13 (m, 2H), 7.01 (d, *J* = 8.2 Hz, 1H), 6.94 (d, *J* = 8.4 Hz, 1H), 6.78 (t, *J* = 7.4 Hz, 1H), 6.73 – 6.67 (m, 2H), 5.49 (s, 1H), 5.19 (d, *J* = 15.2 Hz, 1H), 5.04 (d, *J* = 15.2 Hz, 1H), 4.12 (d, *J* = 10.4 Hz, 1H), 3.10 (d, *J* = 10.4 Hz, 1H), 3.00 (s, 3H), 1.62 (s, 3H).; ^{13}C-NMR (101 MHz, $CDCl_3$) δ

157.1, 151.7, 139.6, 137.0, 133.2, 132.5, 129.8, 129.6, 128.3, 128.0, 127.6, 127.6, 127.5, 126.2, 125.8, 124.8, 124.3, 123.4, 121.3, 116.6, 114.6, 113.4, 98.4, 65.9, 54.6, 48.1, 47.5, 23.2.

Enantiometric excess of the product was determined by chiral stationary phase HPLC analysis using Daicel IC column (*n*-Hexane/*i*-PrOH 98:2 at 0.35 ml/min, λ = 230 nm, t_{minor} = 25.0 min, t_{major} = 26.8 min, 89% *ee*, $[\alpha]_D^{20}$ = -72.99 (*c* = 0.27, in $CHCl_3$).; HRMS (ESI) *m/z* calcd for $C_{28}H_{24}O_3$, $[M+Na]^+$: 431.1618, found: 431.1613.

(6a*S*,12*S*,12a*S*)-2,6a-dimethyl-12-(naphthalen-2-yl)-5,6a,12,12a-tetrahydroisochromeno[3,4-b]chromene

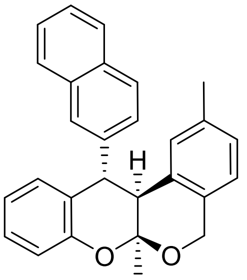

4s

White solid, yield 72 %, **m.p.** 119-122°C, 10:1 d.r., ^{1}H-NMR (700 MHz, $CDCl_3$) δ 7.84 (d, *J* = 7.8 Hz, 1H), 7.79 (d, *J* = 8.4 Hz, 1H), 7.67 (d, *J* = 7.8 Hz, 1H), 7.50 – 7.41 (m, 2H), 7.31 (s, 1H), 7.20 – 7.13 (m, 2H), 7.01 (d, *J* = 8.2 Hz, 1H), 6.96 – 6.90 (m, 2H), 6.77 (dd, *J* = 10.9, 4.0 Hz, 1H), 6.68 (d, *J* = 7.7 Hz, 1H), 5.81 (s, 1H), 5.22 (d, *J* = 15.4 Hz, 1H), 5.05 (d, *J* = 15.4 Hz, 1H), 4.09 (d, *J* = 10.5 Hz, 1H), 3.09 (d, *J* = 10.5 Hz, 1H), 1.82 (s, 3H), 1.60 (s, 3H).; ^{13}C-NMR (176 MHz, $CDCl_3$) δ 151.8, 139.7, 135.7, 135.1, 133.2, 132.5, 130.3, 129.7, 129.6, 128.5, 128.1, 128.0, 127.6, 127.6, 127.6, 127.6, 126.0, 125.7, 124.4, 123.6, 121.3, 116.7, 98.5, 66.1, 48.2, 47.3, 23.3, 20.7.

Enantiometric excess of the product was determined by chiral stationary phase HPLC analysis using Daicel IC column (*n*-Hexane/*i*-PrOH 98:2 at 0.6 ml/min, λ = 230 nm, t_{minor} = 14.7 min, t_{major} = 15.6 min, 89% *ee*, $[\alpha]_D^{20}$ = -46.66 (*c* = 0.21, in $CHCl_3$).; HRMS (ESI) *m/z* calcd for $C_{28}H_{24}O_2$, $[M+Na]^+$: 415.1669, found: 415.1671.

(6a*S*,12*S*,12a*S*)-2-fluoro-6a-methyl-12-(naphthalen-2-yl)-5,6a,12,12a-tetrahydroisochromeno[3,4-b]chromene

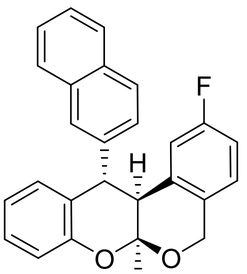

4t

White solid, yield 55 %, **m.p.** 130-134°C, 13:1 d.r.,^{1}H-NMR (700 MHz, $CDCl_3$) δ 7.87 – 7.80 (m, 2H), 7.70 (d, *J* = 7.4 Hz, 1H), 7.52 – 7.43 (m, 2H), 7.38 (s, 1H), 7.20 – 7.14 (m, 2H), 7.04 – 7.18 (m, 2H), 6.85 (td, *J* = 8.4, 2.5 Hz, 1H), 6.77 (t, *J* = 7.3 Hz, 1H), 6.64 (d, *J* = 7.7 Hz, 1H), 5.83 (dd, *J* = 9.3, 2.3 Hz, 1H), 5.23 (d, *J* = 15.4 Hz, 1H), 5.05 (d, *J* = 15.4 Hz, 1H), 4.12 (d, *J* = 10.6 Hz, 1H), 3.18 (d, *J* = 10.5 Hz, 1H), 1.59 (s, 3H).; ^{13}C-NMR (176 MHz, $CDCl_3$) δ 160.6 (J_{C-F} = 244.6 Hz), 151.6, 139.1, 138.3 (J_{C-F} = 8.80 Hz), 133.3, 132.6, 129.7, 129.4, 128.5, 128.1, 127.8, 127.7, 127.3 (J_{C-F} = 1.8 Hz), 127.2, 126.2, 125.9, 125.4 (J_{C-F} = 7.1 Hz), 124.2, 121.5, 116.7, 116.1 (J_{C-F} = 22.9 Hz), 114.2 (J_{C-F} = 22.9 Hz), 98.2, 65.7, 48.0, 47.2, 23.3.

Enantiometric excess of the product was determined by chiral stationary phase HPLC analysis using Daicel AD-H column (*n*-Hexane/*i*-PrOH 95:5 at 0.9 ml/min, λ = 230 nm, t_{minor} = 7.1 min, t_{major} = 10.3 min, 91 % *ee*, $[\alpha]_D^{20}$ = 50.35 (*c* = 0.28 in $CHCl_3$).; HRMS (ESI) *m/z* calcd for $C_{27}H_{21}O_2F$, $[M+Na]^+$: 419.1418, found: 419.1423.

(6a*S*,12*S*,12a*S*)-2-chloro-6a-methyl-12-(naphthalen-2-yl)-5,6a,12,12a-tetrahydroisochromeno[3,4-b]chromene

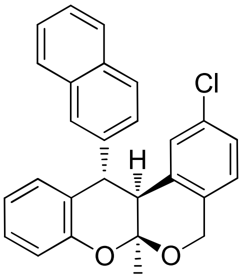

4u

White solid, yield 61 %, **m.p.** 197-200°C, >20:1 d.r., ^{1}H-NMR (400 MHz, $CDCl_3$) δ 7.90 – 7.79 (m, 2H), 7.75 – 7.68 (m, 1H), 7.54 – 7.44 (m, 2H), 7.35 (s, 1H), 7.22 – 7.10 (m, 3H), 7.04 – 6.96 (m, 2H), 6.79 (t, *J* = 7.4 Hz, 1H), 6.67 (d, *J* = 7.6 Hz, 1H), 6.06 (s, 1H), 5.22 (d, *J* = 16.0 Hz, 1H), 5.04 (d, *J* = 16.0 Hz, 1H), 4.10 (d, *J* = 10.4 Hz, 1H), 3.13 (d, *J* = 10.4 Hz, 1H), 1.59 (s, 3H).; ^{13}C-NMR (176 MHz, $CDCl_3$) δ 151.6, 139.0, 137.9, 133.2, 132.6, 131.4, 130.2, 129.7, 129.4, 129.4, 128.5, 128.1, 127.7, 127.7, 127.2, 127.2, 126.2, 125.9, 125.3, 124.0, 121.5, 116.7, 98.2, 65.6, 48.1, 47.1, 23.3.

Enantiometric excess of the product was determined by chiral stationary phase HPLC analysis using Daicel AD-H column (*n*-Hexane/*i*-PrOH 98:2 at 0.5 ml/min, λ = 230 nm, t_{minor} = 16.5 min, t_{major} = 32.4 min, 90 % *ee*, $[\alpha]_D^{20}$ = 48.30 (*c* = 0.31, in $CHCl_3$); HRMS (ESI) *m/z* calcd for $C_{27}H_{21}O_2Cl$, $[M+Na]^+$: 435.1122, found: 435.1124.

(6a*S*,12*S*,12a*S*)-3-chloro-6a-methyl-12-(naphthalen-2-yl)-5,6a,12,12a-tetrahydroisochromeno[3,4-b]chromene

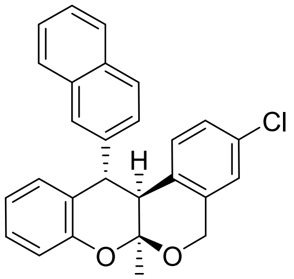

4v

White solid, yield 55 %, **m.p.** 94-98°C, 3:1 d.r., ^{1}H-NMR (400 MHz, $CDCl_3$) δ 7.90 – 7.79 (m, 2H), 7.75 – 7.68 (m, 1H), 7.54 – 7.44 (m, 2H), 7.35 (s, 1H), 7.22 – 7.10 (m, 3H), 7.04 – 6.96 (m, 2H), 6.79 (t, *J* = 7.4 Hz, 1H), 6.67 (d, *J* = 7.6 Hz, 1H), 6.06 (s, 1H), 5.22 (d, *J* = 16.0 Hz, 1H), 5.04 (d, *J* = 16.0 Hz, 1H), 4.10 (d, *J* = 10.4 Hz, 1H), 3.13 (d, *J* = 10.4 Hz, 1H), 1.59 (s, 3H).; ^{13}C-NMR (176 MHz, $CDCl_3$) δ 151.5, 139.1, 134.5, 133.5, 133.2, 132.7, 132.6, 130.9, 129.6, 129.5, 128.5, 128.1, 127.7, 127.7, 127.3, 126.2, 125.9, 125.9, 124.1, 124.0, 121.4, 116.6, 98.3, 65.6, 48.1, 46.6, 23.1.

Enantiometric excess of the product was determined by chiral stationary phase HPLC analysis using Daicel AD-H column (*n*-Hexane/*i*-PrOH 98:2 at 0.5 ml/min, λ = 230 nm, t_{minor} = 17.6 min, t_{major} = 26.3 min, 91 % *ee*, $[\alpha]_D^{20}$ = -29.13 (*c* = 0.23, in $CHCl_3$); HRMS (ESI) *m/z* calcd for $C_{27}H_{21}O_2Cl$, $[M+Na]^+$: 435.1122, found: 435.1117.

(6a*S*,12*S*,12a*S*)-2-bromo-6a-methyl-12-(naphthalen-2-yl)-5,6a,12,12a-tetrahydroisochromeno[3,4-b]chromene

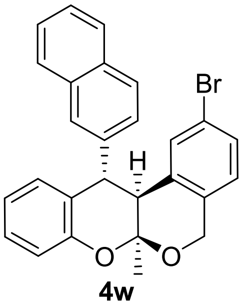

4w

White solid, yield 62 %, **m.p.** 198-202°C, 11:1 d.r., ^{1}H-NMR (700 MHz, $CDCl_3$) δ 7.85 (d, J = 7.2 Hz, 1H), 7.82 (d, J = 8.4 Hz, 1H), 7.72 – 7.68 (m, 1H), 7.50 – 7.45 (m, 2H), 7.34 (s, 1H), 7.27 (dd, J = 8.1, 2.0 Hz, 1H), 7.19 – 7.12 (m, 2H), 7.01 (dd, J = 8.3, 1.0 Hz, 1H), 6.93 (d, J = 8.2 Hz, 1H), 6.79 (td, J = 7.6, 1.1 Hz, 1H), 6.69 (d, J = 7.7 Hz, 1H), 6.18 (d, J = 1.9 Hz, 1H), 5.18 (d, J = 15.9 Hz, 1H), 5.01 (d, J = 15.9 Hz, 1H), 4.08 (s, 1H), 3.11 (d, J = 10.5 Hz, 1H), 1.59 (s, 3H).; ^{13}C-NMR (176 MHz, $CDCl_3$) δ 151.6, 138.9, 138.2, 133.2, 132.7, 132.4, 130.7, 130.0, 129.7, 129.5, 128.5, 128.2, 127.7, 127.2, 126.2, 125.9, 125.6, 124.0, 121.5, 119.3, 116.7, 98.2, 65.7, 48.1, 47.2, 23.3.

Enantiometric excess of the product was determined by chiral stationary phase HPLC analysis using Daicel AD-H column (n-Hexane/i-PrOH 98:2 at 0.5 ml/min, λ = 230 nm, t_{minor} = 17.2 min, t_{major} = 36.4 min, 94 % *ee*, $[\alpha]_D^{20}$ = 32.76 (c = 0.29, in $CHCl_3$); HRMS (ESI) m/z calcd for $C_{27}H_{21}O_2Br$, $[M+Na]^+$: 479.0617, found: 479.0611.

(6a*S*,12*S*,12a*S*)-3-bromo-6a-methyl-12-(naphthalen-2-yl)-5,6a,12,12a-tetrahydroisochromeno[3,4-b]chromene

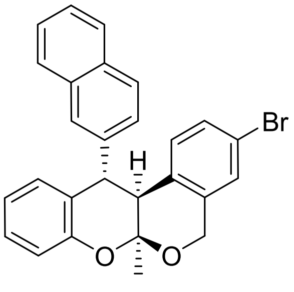

4x

White solid, yield 58 %, **m.p.** 88-90°C, 4:1 d.r., ^{1}H-NMR (700 MHz, $CDCl_3$) δ 7.87 – 7.83 (m, 1H), 7.80 (d, J = 8.4 Hz, 1H), 7.70 (d, J = 8.9 Hz, 1H), 7.51 – 7.44 (m, 2H), 7.35 (s, 1H), 7.21 (s, 1H), 7.19 – 7.14 (m, 2H), 7.00 (dd, J = 8.3, 1.1 Hz, 1H), 6.88 – 6.84 (m, 1H), 6.77 (td, J = 7.6, 1.1 Hz, 1H), 6.62 (d, J = 7.7 Hz, 1H), 5.94 (d, J = 8.2 Hz, 1H), 5.21 (d, J = 16.0 Hz, 1H), 5.04 (d, J = 16.0 Hz, 1H), 4.07 (d, J = 10.7 Hz, 1H), 3.16 (d, J = 10.7 Hz, 1H), 1.59 (s, 3H).; ^{13}C-NMR (176 MHz, $CDCl_3$) δ 151.5, 139.0, 135.0, 133.9, 133.2, 132.6, 131.2, 129.6, 129.5, 128.8, 128.5, 128.1, 127.7, 127.7, 127.3, 127.0, 126.2, 125.9, 124.1, 121.5, 120.7, 116.6, 98.2, 65.5, 48.0, 46.6, 23.1.

Enantiometric excess of the product was determined by chiral stationary phase HPLC analysis using Daicel AD-H column (n-Hexane/i-PrOH 98:2 at 0.5 ml/min, λ = 230 nm, t_{minor} = 18.9 min, t_{major} = 26.0 min, 93 % *ee*, $[\alpha]_D^{20}$ = -20.45 (c = 0.22, in $CHCl_3$).; HRMS (ESI) m/z calcd for $C_{27}H_{21}O_2Br$, $[M+Na]^+$: 479.0617, found: 479.0608.

(6a*S*,12*S*,12a*S*)-6a-methyl-12-(naphthalen-2-yl)-2-(trifluoromethyl)-5,6a,12,12a-tetrahydroisochromeno[3,4-b]chromene

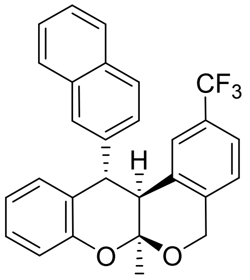

4y

White solid, yield 43 %, **m.p.** 194-198°C, 5:1 d.r., ^{1}H-NMR (700 MHz, $CDCl_3$) δ 7.86-7.80 (m, 2H), 7.63 (d, *J* = 7.9 Hz, 1H), 7.50-7.42 (m, 2H), 7.39 (d, *J* = 7.8 Hz, 1H), 7.25 (s, 1H), 7.23-7.12 (m, 3H), 7.03 (d, *J* = 8.2 Hz, 1H), 6.81 (t, *J* = 7.4 Hz, 1H), 6.72 (d, *J* = 7.6 Hz, 1H), 6.20 (s, 1H), 5.29 (d, *J* = 16.5 Hz, 1H), 5.12 (d, *J* = 16.3 Hz, 1H), 4.07 (d, *J* = 10.6 Hz, 1H), 3.18 (d, *J* = 10.6 Hz, 1H), 1.61 (s, 3H).; ^{13}C-NMR (176 MHz, $CDCl_3$) δ 151.6, 138.4, 136.7, 135.8, 133.2, 132.6, 129.6, 129.6, 128.6, 128.2, 128.0 ($J_{C\text{-}F}$ = 32.5 Hz), 127.6, 127.5, 127.0, 126.7 ($J_{C\text{-}F}$ = 3.5 Hz), 126.2, 125.9, 124.3, 123.8, 123.7 ($J_{C\text{-}F}$ = 3.5 Hz), 123.5 ($J_{C\text{-}F}$ = 271.0 Hz), 121.6, 116.7, 98.2, 65.9, 48.2, 47.3, 29.7, 23.1.

Enantiometric excess of the product was determined by chiral stationary phase HPLC analysis using Daicel AD-H column (*n*-Hexane/*i*-PrOH 98:2 at 0.5 ml/min, λ = 230 nm, t_{minor} = 13.0 min, t_{major} = 17.0 min, 95 % *ee*, $[\alpha]_D^{20}$ = -10.15 (*c* = 0.23, in $CHCl_3$); HRMS (ESI) *m/z* calcd for $C_{28}H_{21}O_2F_3$, $[M+Na]^+$: 469.1386, found: 469.1377

(6*aS*,12*S*,12*aS*)-benzyl 6a-methyl-12-(naphthalen-2-yl)-12,12a-dihydro-5H-chromeno[2,3-c]isoquinoline-6(6aH)-carboxylate

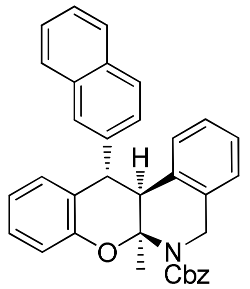

5a

White solid, yield 63 %, **m.p.** 87-90°C, 1.5:1 d.r., ^{1}H-NMR (400 MHz, $CDCl_3$) δ 7.79 (d, *J* = 8.8 Hz, 0.57×1H), 7.72 (d, *J* = 8.4 Hz, 0.57×1H), 7.66 – 7.60 (m, 0.43×2H), 7.50 – 6.70 (m, 16H), 6.72 (d, *J* = 7.6 Hz, 0.57×1H), 6.61 (d, *J* = 7.6 Hz, 0.43×1H), 6.54 (d, *J* = 8.4 Hz, 0.43×1H), 6.40 (d, *J* = 7.6 Hz, 0.57×1H), 5.23 (s, 0.57×1H), 5.14 (s, 0.43×1H), 5.00 (d, *J* = 16.2 Hz, 0.57×1H), 4.77 (d, *J* = 16.2 Hz, 0.57×1H), 4.47 (d, *J* = 6.8 Hz, 0.43×1H), 4.10 (d, *J* = 10.4 Hz, 0.57×1H), 3.93 (d, *J* = 17.2 Hz, 0.43×1H), 3.62 (d, *J* = 6.8 Hz, 0.43×1H), 3.34 (d, *J* = 10.4 Hz, 0.57×1H), 2.73 (d, *J* = 17.2 Hz, 0.43×1H), 1.66 (s, 0.57×3H), 1.66 (s, 0.43×3H). ^{13}C-NMR (101 MHz, $CDCl_3$) δ 155.9, 155.0, 152.7, 151.4, 140.8, 137.7, 137.1, 136.9, 136.7, 134.4, 133.3, 133.2, 132.5, 132.5, 131.8, 131.8, 130.1, 129.6, 129.4, 128.55, 128.5, 128.5, 128.4, 128.4, 128.3, 128.3, 128.1, 127.9, 127.9, 127.9, 127.8, 127.7, 127.7, 127.4, 127.3, 126.9, 126.8, 126.7, 126.1, 126.0, 125.8, 125.8, 125.6, 125.6, 123.8, 121.9, 121.5, 118.1, 117.8, 87.5, 86.7, 67.4, 66.9, 54.4, 49.6, 48.6, 48.0, 46.56, 46.3, 27.9, 27.0.

Enantiometric excess of the product was determined by chiral stationary phase HPLC analysis using Daicel AD-H column (*n*-Hexane/*i*-PrOH 98:2 at 0.4 ml/min, λ = 230 nm, t_{minorA} = 13.7 min, t_{majorB} = 17.6 min, t_{minorB} = 32.8 min, t_{majorA} = 35.3 min, 22 % *ee* for major product, 15 % *ee* for minor product, $[\alpha]_D^{20}$ = -7.25 (*c* = 0.27, in $CHCl_3$); HRMS (ESI) *m/z* calcd for $C_{35}H_{29}NO_3$, $[M+H]^+$: 512.2220, found: 512.2219.

(4*S*)-4-(naphthalen-2-yl)-3'H-spiro[chroman-2,1'-isobenzofuran]

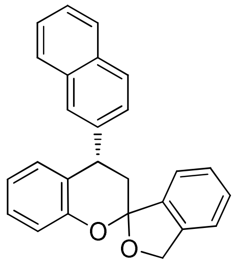

5b

White solid, yield 72 %, **m.p.** 96-100°C, >20:1 d.r., ^{1}H-NMR (400 MHz, $CDCl_3$) δ 7.91 – 7.77 (m, 4H), 7.54 – 7.30 (m, 7H), 7.13 (dt, *J* = 8.6, 4.2 Hz, 1H), 6.87 (d, *J* = 8.2 Hz, 1H), 6.80 (d, *J* = 4.2 Hz, 2H), 5.36 (d, *J* = 12.8 Hz, 1H), 5.14 (d, *J* = 12.8 Hz, 1H), 4.76 (dd, *J* = 13.2, 5.6 Hz, 1H), 2.77 (t, *J* = 13.2 Hz, 1H), 2.42 (dd, *J* = 13.2, 5.6 Hz, 1H); ^{13}C-NMR (101 MHz, $CDCl_3$) δ 153.3, 141.4, 140.1, 139.7, 133.6, 132.6, 129.6, 129.5, 128.6, 128.0, 127.9, 127.9, 127.7, 127.7, 126.7, 126.2, 125.7, 125.4, 122.1, 121.4, 121.1, 117.2, 108.5, 71.9, 39.6, 39.2.
Enantiometric excess of the product was determined by chiral stationary phase HPLC analysis using Daicel AD-H column (*n*-Hexane/*i*-PrOH 97:3 at 0.6 ml/min, λ = 230 nm, t_{minor} = 8.3 min, t_{major} = 13.8 min, 52 % *ee,* $[\alpha]_D^{20}$ = 32.33 (*c* = 0.30, in $CHCl_3$); HRMS (ESI) *m/z* calcd for $C_{26}H_{20}O_2$, $[M+Na]^+$: 367.1536, found: 367.1533.

(4*S*)-5'-methyl-4-(naphthalen-2-yl)-3'H-spiro[chroman-2,1'-isobenzofuran]

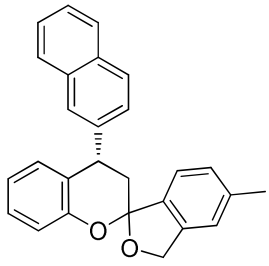

5c

Colorless oil, yield 76 %, >20:1 d.r., ^{1}H-NMR (400 MHz, $CDCl_3$) δ 7.88 – 7.76 (m, 4H), 7.53 – 7.42 (m, 2H), 7.38 (dd, *J* = 8.6, 1.2 Hz, 1H), 7.32 (d, *J* = 7.6 Hz, 1H), 7.19 (d, *J* = 7.8 Hz, 1H), 7.16 – 7.10 (m, 2H), 6.87 (d, *J* = 8.4 Hz, 1H), 6.82 – 6.75(m, 2H), 5.32 (d, *J* = 12.8 Hz, 1H), 5.08 (d, *J* = 12.8 Hz, 1H), 4.75 (dd, *J* = 13.2, 5.6 Hz, 1H), 2.75 (t, *J* = 13.2 Hz, 1H), 2.46 – 2.33 (m, 4H); ^{13}C-NMR (101 MHz, $CDCl_3$) δ 153.4, 141.5, 140.4, 139.8, 137.1, 133.6, 132.6, 129.5, 128.9, 128.5, 127.9, 127.7, 127.7, 126.7, 126.2, 125.7, 125.4, 121.8, 121.8, 121.0, 117.2, 108.4, 71.8, 39.6, 39.2, 21.5.
Enantiometric excess of the product was determined by chiral stationary phase HPLC analysis using Daicel AD-H column (*n*-Hexane/*i*-PrOH 97:3 at 0.6 ml/min, λ = 230 nm, t_{minor} = 9.2 min, t_{major} = 15.6 min, 55 % *ee,* $[\alpha]_D^{20}$ = 45.95 (*c* = 0.42, in $CHCl_3$); HRMS (ESI) *m/z* calcd for $C_{27}H_{22}O_2$, $[M+H]^+$: 379.1693, found: 379.1694.

(4'*S*)-4'-(naphthalen-2-yl)-3H-spiro[benzofuran-2,2'-chroman]

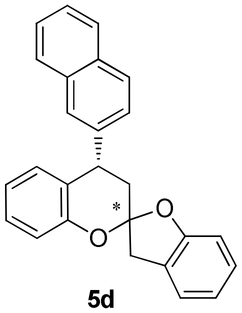

5d

White solid, yield 31 %, **m.p.** 191-194°C, >20:1 d.r., ^{1}H-NMR (700 MHz, $CDCl_3$) δ 7.86 – 7.75 (m, 4H), 7.51 – 7.44 (m, 2H), 7.33 (d, *J* = 8.5 Hz,1H), 7.21 (d, *J* = 7.3 Hz, 1H), 7.18 – 7.14 (m, 2H), 6.95 – 6.88 (m, 2H), 6.84 – 6.82 (d, *J* =

4.6 Hz, 3H), 4.48 (dd, J = 10.2, 6.4 Hz, 1H), 3.44 (d, J = 16.2 Hz, 1H), 3.35 (d, J = 16.2 Hz, 1H), 2.73 (dd, J = 13.4, 10.3 Hz, 1H), 2.60 (dd, J = 13.4, 6.4 Hz, 1H); ^{13}C-NMR (176 MHz, $CDCl_3$) δ 157.9, 152.6, 141.1, 133.5, 132.6, 129.7, 128.5, 128.4, 128.3, 127.7, 127.7, 126.6, 126.2, 125.8, 124.9, 124.9, 124.4, 121.5, 121.4, 117.8, 110.1, 110.0, 41.1, 40.9, 39.7.

Enantiometric excess of the product was determined by chiral stationary phase HPLC analysis using Daicel AD-H column (*n*-Hexane/*i*-PrOH 95:5 at 0.8 ml/min, λ = 230 nm, t_{major} = 14.0 min, t_{minor} = 17.2 min, 37 % *ee,* $[\alpha]_D^{20}$ = 20.00 (c = 0.21, in $CHCl_3$); HRMS (ESI) *m/z* calcd for $C_{26}H_{20}O_2$, $[M+H]^+$: 365.1536, found: 365.1534.

(5a*S*,12a*R*,13*S*)-5a-methyl-13-(naphthalen-2-yl)-7,12,12a,13-tetrahydro-5aH-benzo[5,6]oxepino[2,3-b]chromene

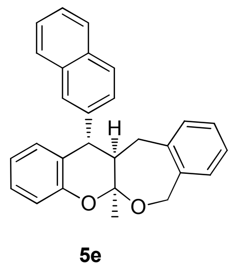

5e

Colorless oil, yield 14 %, ^{1}H-NMR (700 MHz, $CDCl_3$) δ 7.76 – 7.71 (m, 1H), 7.70 – 7.55 (m, 2H), 7.41 – 7.34 (m, 2H), 7.33 – 7.25 (s, 1H), 7.17 (t, J = 7.7 Hz, 1H), 7.11 – 6.96 (m, 4H), 6.80 (t, J = 7.4 Hz, 1H), 6.73 (d, J = 7.5 Hz, 1H), 6.69 (t, J = 7.4 Hz, 1H), 6.38 (d, J = 7.4 Hz, 1H), 4.92 (d, J = 12.4 Hz, 1H), 4.21 (dt, J = 12.7, 3.2 Hz, 1H), 4.04 (t, J = 12.5 Hz, 1H), 3.84 – 3.78 (m, 1H), 3.42 (d, J = 12.4 Hz, 1H), 2.73 (dd, J = 15.7, 3.1 Hz, 1H), 1.56 (s, 3H); ^{13}C-NMR (176 MHz, $CDCl_3$) δ 152.2, 138.3, 137.1, 132.4, 132.3, 130.8, 130.1, 128.0, 127.6, 126.9, 126.4, 125.8, 125.4, 121.2, 116.94, 100.2, 65.3, 43.3, 38.6, 22.9.

Enantiometric excess of the product was determined by chiral stationary phase HPLC analysis using Daicel IA column (*n*-Hexane/*i*-PrOH 98:2 at 0.5 ml/min, λ = 230 nm, t_1 = 10.7 min, t_2 = 12.2 min, 0 % *ee,* HRMS (ESI) *m/z* calcd for $C_{28}H_{24}O_2$, $[M+H]^+$: 393.1849, found: 393.1845.

(*S*,*Z*)-2-((2-methyl-5,6-dihydro-4H-benzo[d]oxocin-1-yl)(naphthalen-2-yl)methyl)phenol

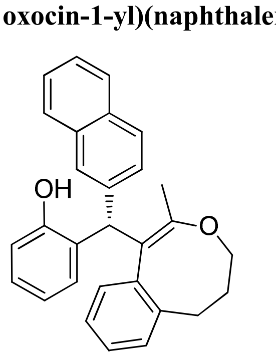

5f

Colorless oil, yield 73 %, ^{1}H-NMR (700 MHz, $CDCl_3$) δ 8.45 (s, 1H), 7.88 – 7.82 (m, 3H), 7.57 – 7.48 (m, 3H), 7.39 (d, J = 7.6 Hz, 1H), 7.25 – 7.19 (m, 1H), 7.19 – 7.09 (m, 3H), 6.99 – 6.91 (m, 2H), 6.85 – 6.79 (m, 1H), 5.75 (s, 1H), 3.72 – 3.59 (m, 2H), 3.03 – 2.84 (m, 2H), 2.12 – 2.05 (m, 2H), 2.01 (s, 3H); ^{13}C-NMR (176 MHz, $CDCl_3$) δ 155.8, 143.2, 137.6, 133.2, 133.1, 132.3, 129.3, 128.9, 128.7, 128.7, 128.2, 127.7, 127.7, 126.4, 126.3, 126.3, 125.9, 125.1, 125.0, 123.5, 119.7, 117.3, 89.7, 85.3, 78.2, 69.3, 31.2, 30.4, 4.4.

Enantiometric excess of the product was determined by chiral stationary phase HPLC analysis using Daicel AD-H column (*n*-Hexane/*i*-PrOH 95:5 at 0.8 ml/min, λ = 230 nm, t_{minor} = 27.9 min, t_{major} = 30.2 min, 28 % *ee,* $[\alpha]_D^{20}$ = -15.48 (c = 0.31, in $CHCl_3$); HRMS (ESI) *m/z* calcd for $C_{29}H_{26}O_2$, $[M+Na]^+$: 429.1825, found: 429.1825.

(6*aS*,12*S*,12*S*)-6a-benzyl-12-(naphthalen-2-yl)-5,6a,12,12a-tetrahydroisochromeno[3,4-b]chromene

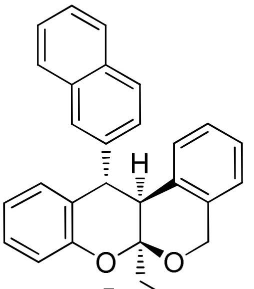

5g

Colorless oil, yield 41 %, >20:1 d.r., ^{1}H-NMR (400 MHz, $CDCl_3$) δ 7.71 (d, *J* = 7.6 Hz, 1H), 7.55 – 7.45 (m, 2H), 7.41 – 7.29 (m, 2H), 7.24 – 6.85 (m, 15H), 4.99 (d, *J* = 14.8 Hz, 1H), 4.66 (d, *J* = 14.8 Hz, 1H), 4.58 (d, *J* = 7.6 Hz, 1H), 3.89 (d, *J* = 7.6 Hz, 1H), 3.06 (d, *J* = 16.8 Hz, 1H), 2.87 (d, *J* = 16.8 Hz, 1H); ^{13}C-NMR (176 MHz, $CDCl_3$) δ 152.2, 139.4, 138.0, 133.2, 132.5, 132.0, 131.2, 131.1, 130.8, 130.5, 130.0, 128.8, 128.0, 127.8, 127.7, 127.3, 126.9, 126.6, 125.8, 125.6, 125.6, 125.3, 125.3, 123.8, 121.4, 117.5, 99.3, 62.2, 53.0, 46.9, 35.8.

Enantiometric excess of the product was determined by chiral stationary phase HPLC analysis using Daicel AD-H column (*n*-Hexane/*i*-PrOH 95:5 at 0.8 ml/min, λ = 230 nm, t_{major} = 6.1 min, t_{minor} = 8.4 min, 55 % *ee,* $[\alpha]_D^{20}$ = -9.54 (*c* = 0.22, in $CHCl_3$); HRMS (ESI) *m/z* calcd for $C_{33}H_{26}O_2$, $[M+Na]^+$: 477.1825, found: 477.1823.

(6*aS*,12*S*,12*S*)-6a-benzyl-12-(naphthalen-2-yl)-5,6a,12,12a-tetrahydroisochromeno[3,4-b]chromene

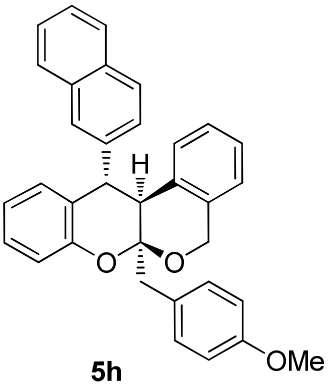

5h

White solid, yield 39 %, **m.p.** 98-100°C, >20:1 d.r., ^{1}H-NMR (700 MHz, $CDCl_3$) δ 7.72 (d, *J* = 7.8 Hz, 1H), 7.53 (d, *J* = 7.9 Hz, 1H), 7.49 (d, *J* = 8.5 Hz, 1H), 7.36 (ddd, *J* = 15.0, 13.4, 6.6 Hz, 2H), 7.20 (dd, *J* = 11.3, 4.1 Hz, 1H), 7.17 – 7.09 (m, 3H), 7.03 – 6.84 (m, 8H), 6.55 (s, 2H), 4.97 (d, *J* = 14.6 Hz, 1H), 4.64 (d, *J* = 14.6 Hz, 1H), 4.54 (d, *J* = 7.6 Hz, 1H), 3.83 (d, *J* = 7.6 Hz, 1H), 3.68 (s, 3H), 3.03 (d, *J* = 16.7 Hz, 1H), 2.85 (d, *J* = 16.7 Hz, 1H); ^{13}C-NMR (176 MHz, $CDCl_3$) δ 158.5, 152.2, 139.5, 133.2, 132.5, 131.9, 131.5, 131.3, 131.1, 130.8, 130.1, 130.1, 128.8, 127.9, 127.8, 127.3, 126.6, 125.8, 125.7, 125.6, 125.3, 125.2, 123.8, 121.4, 117.4, 113.1, 99.4, 62.2, 55.1, 52.2, 46.9, 35.8.

Enantiometric excess of the product was determined by chiral stationary phase HPLC analysis using Daicel AD-H column (*n*-Hexane/*i*-PrOH 97:3 at 0.6 ml/min, λ = 230 nm, t_{major} = 12.0 min, t_{minor} = 18.9 min, 53 % *ee,* $[\alpha]_D^{20}$ = -15.30 (*c* = 0.28, in $CHCl_3$); HRMS (ESI) *m/z* calcd for $C_{34}H_{28}O_3$, $[M+Na]^+$: 507.1931, found: 507.1928.

(4*S*)-4-(naphthalen-2-yl)-3-phenyl-4',5'-dihydro-3'H-spiro[chroman-2,2'-furan]

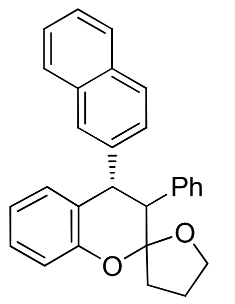

5i

White solid, yield 52 %, **m.p.** 126-144°C,>20:1 d.r., ^{1}H-NMR (400 MHz, $CDCl_3$) δ 7.74 – 7.66 (m, 1H), 7.65 – 7.58 (m, 2H), 7.43 – 7.32 (m, 3H), 7.30 – 7.04 (m, 7H), 6.91 (d, J = 8.0 Hz, 1H), 6.75 (t, J = 7.4 Hz, 1H), 6.68 (d, J = 7.6 Hz, 1H), 4.77 (d, J = 12.4 Hz, 1H), 4.08 (dd, J = 14.6, 7.0 Hz, 1H), 3.97 – 3.89 (m, 1H), 3.56 (d, J = 12.4 Hz, 1H), 2.21 – 2.04 (m, 2H), 1.82 – 1.72 (m, 1H), 1.60 – 1.50 (m, 1H); ^{13}C-NMR (101 MHz, $CDCl_3$) δ 152.5, 140.7, 139.0, 133.2, 132.2, 130.1, 129.5, 128.6, 128.0, 127.9, 127.6, 127.5, 126.8, 126.7, 125.7, 125.3, 120.9, 117.1, 108.8, 68.8, 53.3, 47.1, 35.2, 24.6.

Enantiometric excess of the product was determined by chiral stationary phase HPLC analysis using Daicel AD-H column (*n*-Hexane/*i*-PrOH 97:3 at 0.6 ml/min, λ = 230 nm, t_{minor} = 8.7 min, t_{major} = 11.1 min, 66 % *ee,* $[\alpha]_D^{20}$ = 99.99 (c = 0.47, in $CHCl_3$); HRMS (ESI) *m/z* calcd for $C_{28}H_{24}O_2$, $[M+H]^+$: 393.1849, found: 393.1844.

(2-((2*R*,3*S*,4*S*)-2-methyl-4-(*m*-tolyl)chroman-3-yl)phenyl)methanol

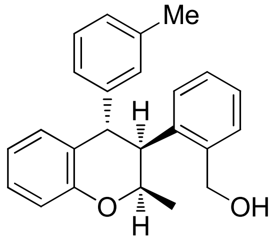

6

White solid, yield 73 %, **m.p.** 110-118°C, 11:1 d.r., ^{1}H-NMR (400 MHz, $CDCl_3$) δ 7.42 (dd, J = 7.6, 1.2 Hz, 1H), 7.13 – 6.88 (m, 5H), 6.88 – 6.73 (m, 5H), 6.46 (d, J = 8.0 Hz, 1H), 4.99 – 4.86 (m, 2H), 4.79 (d, J = 8.8 Hz, 1H), 4.06 (qd, J = 6.6, 2.4 Hz, 1H), 3.68 (dd, J = 8.6, 1.8 Hz, 1H), 2.13 (s, 3H), 1.05 (d, J = 6.8 Hz, 3H). ^{13}C-NMR (101 MHz, $CDCl_3$) δ 153.2, 142.6, 137.3, 134.3, 130.9, 130.4, 130.2, 129.4, 127.7, 127.3, 126.6, 126.4, 126.0, 125.1, 123.5, 120.6, 116.5, 73.6, 67.7, 44.85, 44.3, 21.4, 18.5.

Enantiometric excess of the product was determined by chiral stationary phase HPLC analysis using Daicel AD-H column (*n*-Hexane/*i*-PrOH 90:10 at 1 ml/min, λ = 230 nm, t_{minor} = 6.0 min, t_{major} = 7.4 min, 85 % *ee,* $[\alpha]_D^{20}$ = 76.53 (c = 0.45, in $CHCl_3$); HRMS (ESI) *m/z* calcd for $C_{24}H_{24}O_2$, $[M+H]^+$: 345.1849, found: 345.1851.

2-((*S*)-((3*S*,4*S*)-3-methylisochroman-4-yl)(*m*-tolyl)methyl)phenol

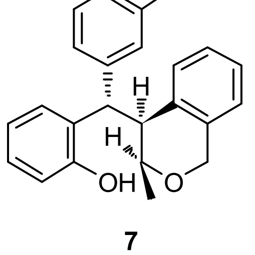

7

White solid, yield 73 %, **m.p.** 120-122°C, 63:37 d.r., ^{1}H-NMR (700 MHz, $CDCl_3$) δ 7.23 – 6.16 (m, 12H), 5.26 (d, *J* = 5.0 Hz, 0.63×1H), 5.17 (d, *J* = 8.1 Hz, 0.37×1H), 4.57 (dq, *J* = 12.7, 6.3 Hz, 0.63×1H), 4.43 (d, *J* = 16.5 Hz, 0.63×1H), 4.29 (dd, *J* = 13.7, 5.6 Hz, 0.37×2H), 4.01 (d, *J* = 15.6 Hz, 0.37×1H), 3.85 (d, *J* = 16.6 Hz, 0.63×1H), 3.55 (dd, *J* = 7.7, 6.6 Hz, 0.37×1H), 3.24 (dd, *J* = 8.4, 5.2 Hz, 0.63×1H), 2.45 (s, 0.37×3H), 2.13 (s, 0.63×3H), 1.09 (d, *J* = 6.3 Hz, 0.63×3H), 1.07 (d, *J* = 6.3 Hz, 0.37×3H). ^{13}C-NMR (176 MHz, $CDCl_3$) δ 153.1, 152.5, 139.5, 138.9, 138.9, 138.9, 138.9, 138.4, 137.7, 136.1, 134.6, 134.5, 133.7, 132.3, 131.9, 131.7, 131.1, 130.6, 130.1, 129.9, 129.0, 128.7, 128.5, 127.3, 126.8, 126.8, 126.7, 126.4, 126.3, 126.0, 120.7, 120.2, 116.1, 115.5, 70.3, 68.7, 58.5, 56.3, 43.4, 43.3, 42.1, 36.2, 22.7, 21.1, 20.9, 20.8.

Enantiometric excess of the product was determined by chiral stationary phase HPLC analysis using Daicel AD-H column (*n*-Hexane/*i*-PrOH 90:10 at 1 ml/min, λ = 230 nm, t_{majorA}= 16.9 min, t_{majorB} = 18.5 min, t_{minorB} = 19.9 min, t_{minorA} = 21.5 min, 83 % *ee* for major product, $[\alpha]_D^{20}$ = -12.12 (c = 0.46, in $CHCl_3$); HRMS (ESI) *m/z* calcd for $C_{24}H_{24}O_2$, $[M+Na]^+$: 367.1669, found: 367.1665

(6*aS*,12*aS*)-9,10-dimethoxy-6a-methyl-6a,12a-dihydro-5H,12H-isochromeno[3,4-b]chromene

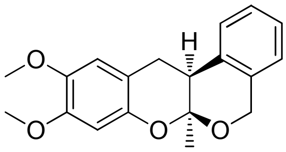

9a

Yellow oil, yield 39 %, >20:1 d.r., ^{1}H-NMR (700 MHz, $CDCl_3$) δ 7.24 (s, 1H), 7.22 – 7.16 (m, 2H), 7.01 (d, *J* = 6.1 Hz, 1H), 6.52 (s, 1H), 6.42 (s, 1H), 5.16 (d, *J* = 15.3 Hz, 1H), 4.90 (d, *J* = 15.3 Hz, 1H), 3.79 (s, 6H), 3.10 (t, *J* = 6.3 Hz, 1H), 3.04 (d, *J* = 6.3 Hz, 2H), 1.60 (s, 3H); ^{13}C-NMR (176 MHz, $CDCl_3$) δ 148.6, 146.5, 143.3, 135.4, 132.8, 127.1, 126.8, 126.5, 124.0, 111.6, 110.1, 100.8, 98.4, 64.5, 56.5, 55.8, 38.3, 29.8, 24.1.

Enantiometric excess of the product was determined by chiral stationary phase HPLC analysis using Daicel AD-H column (*n*-Hexane/*i*-PrOH 90:10 at 0.9 ml/min, λ = 230 nm, t_{major}= 3.7 min, t_{minor} = 8.4 min, 90 % *ee*, $[\alpha]_D^{20}$ = -40.10 (c = 0.46, in $CHCl_3$).; HRMS (ESI) *m/z* calcd for $C_{19}H_{20}O_4$, $[M+H]^+$: 313.1434, found: 313.1424.

6. X-ray crystallographic data of 4l

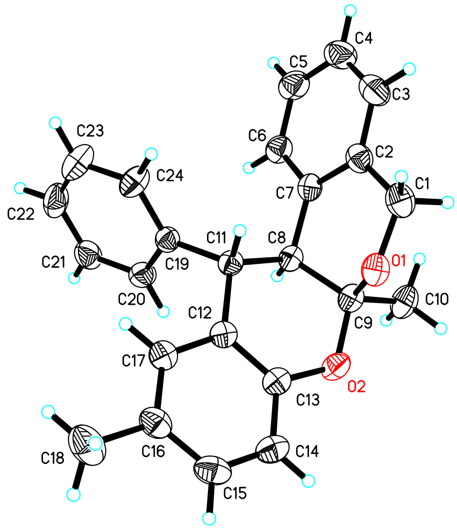

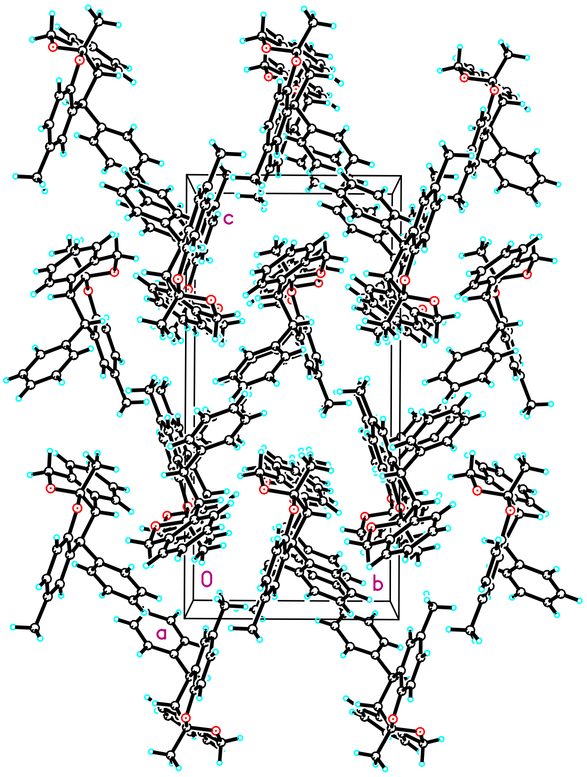

Supplementary Table 3. Crystal data and structure refinement for mo_d8v191072_0m.

Identification code	mo_d8v191072_0m	
Empirical formula	$C_{24}H_{22}O_2$	
Formula weight	342.41	
Temperature	293(2) K	
Wavelength	0.71073 Å	
Crystal system	Orthorhombic	
Space group	P 21 21 21	
Unit cell dimensions	a = 9.5849(5) Å	α= 90°.
	b = 9.7384(4) Å	β= 90°.
	c = 20.1244(9) Å	γ = 90°.
Volume	1878.44(15) $Å^3$	
Z	4	
Density (calculated)	1.211 Mg/m^3	
Absorption coefficient	0.076 mm^{-1}	
F(000)	728	
Crystal size	0.200 x 0.150 x 0.100 mm^3	
Theta range for data collection	2.323 to 25.994°.	
Index ranges	-11<=h<=11, -12<=k<=11, -24<=l<=24	
Reflections collected	17046	
Independent reflections	3674 [R(int) = 0.0475]	
Completeness to theta = 25.242°	99.4 %	
Absorption correction	Semi-empirical from equivalents	
Max. and min. transmission	0.7456 and 0.5496	
Refinement method	Full-matrix least-squares on F^2	
Data / restraints / parameters	3674 / 0 / 238	
Goodness-of-fit on F^2	1.027	
Final R indices [I>2sigma(I)]	R1 = 0.0372, wR2 = 0.0918	
R indices (all data)	R1 = 0.0471, wR2 = 0.0997	
Absolute structure parameter	-1.4(6)	
Extinction coefficient	0.059(7)	
Largest diff. peak and hole	0.106 and -0.105 e.$Å^{-3}$	

Supplementary Table 4. Atomic coordinates (x 10^4) and equivalent isotropic displacement parameters ($Å^2$x 10^3) for mo_d8v191072_0m. U(eq) is defined as one third of the trace of the orthogonalized U^{ij} tensor.

	x	y	z	U(eq)
O(1)	5627(2)	6322(1)	7907(1)	54(1)
O(2)	3964(2)	4919(2)	7498(1)	56(1)
C(1)	6883(3)	6544(3)	8269(1)	66(1)
C(2)	8044(3)	5562(2)	8107(1)	52(1)
C(3)	9363(3)	5764(3)	8384(1)	70(1)
C(4)	10439(3)	4865(3)	8257(1)	74(1)
C(5)	10206(3)	3739(3)	7860(1)	69(1)
C(6)	8907(3)	3522(3)	7582(1)	55(1)
C(7)	7814(2)	4432(2)	7698(1)	45(1)
C(8)	6416(2)	4278(2)	7365(1)	42(1)
C(9)	5288(3)	4906(2)	7803(1)	48(1)
C(10)	5062(3)	4140(3)	8448(1)	64(1)
C(11)	6468(2)	4996(2)	6675(1)	42(1)
C(12)	5023(2)	5345(2)	6422(1)	44(1)
C(13)	3881(2)	5327(2)	6840(1)	48(1)
C(14)	2565(3)	5704(2)	6618(1)	59(1)
C(15)	2399(3)	6153(3)	5975(1)	64(1)
C(16)	3520(3)	6218(3)	5538(1)	57(1)
C(17)	4807(3)	5792(2)	5771(1)	50(1)
C(18)	3346(4)	6790(3)	4847(2)	86(1)
C(19)	7304(2)	4166(2)	6179(1)	45(1)
C(20)	6833(3)	2900(3)	5958(1)	57(1)
C(21)	7583(3)	2156(3)	5498(1)	64(1)
C(22)	8811(3)	2650(3)	5250(1)	73(1)
C(23)	9303(4)	3901(4)	5469(2)	83(1)
C(24)	8550(3)	4644(3)	5932(1)	63(1)

Supplementary Table 5. Bond lengths [Å] and angles [°] for mo_d8v191072_0m.

O(1)-C(1)	1.423(3)
O(1)-C(9)	1.432(3)
O(2)-C(13)	1.385(3)
O(2)-C(9)	1.410(3)
C(1)-C(2)	1.503(3)
C(1)-H(1A)	0.9700
C(1)-H(1B)	0.9700
C(2)-C(7)	1.393(3)
C(2)-C(3)	1.395(4)
C(3)-C(4)	1.378(4)
C(3)-H(3)	0.9300
C(4)-C(5)	1.375(4)
C(4)-H(4)	0.9300
C(5)-C(6)	1.381(4)
C(5)-H(5)	0.9300
C(6)-C(7)	1.392(3)
C(6)-H(6)	0.9300
C(7)-C(8)	1.506(3)
C(8)-C(9)	1.523(3)
C(8)-C(11)	1.555(3)
C(8)-H(8)	0.9800
C(9)-C(10)	1.513(3)
C(10)-H(10A)	0.9600
C(10)-H(10B)	0.9600
C(10)-H(10C)	0.9600
C(11)-C(19)	1.513(3)
C(11)-C(12)	1.514(3)
C(11)-H(11)	0.9800
C(12)-C(13)	1.381(3)
C(12)-C(17)	1.397(3)
C(13)-C(14)	1.388(3)
C(14)-C(15)	1.374(4)
C(14)-H(14)	0.9300
C(15)-C(16)	1.390(4)
C(15)-H(15)	0.9300
C(16)-C(17)	1.382(3)
C(16)-C(18)	1.508(4)

C(17)-H(17)	0.9300
C(18)-H(18A)	0.9600
C(18)-H(18B)	0.9600
C(18)-H(18C)	0.9600
C(19)-C(24)	1.374(3)
C(19)-C(20)	1.387(3)
C(20)-C(21)	1.379(3)
C(20)-H(20)	0.9300
C(21)-C(22)	1.366(4)
C(21)-H(21)	0.9300
C(22)-C(23)	1.379(5)
C(22)-H(22)	0.9300
C(23)-C(24)	1.384(4)
C(23)-H(23)	0.9300
C(24)-H(24)	0.9300

C(1)-O(1)-C(9)	114.39(18)
C(13)-O(2)-C(9)	118.02(16)
O(1)-C(1)-C(2)	114.78(19)
O(1)-C(1)-H(1A)	108.6
C(2)-C(1)-H(1A)	108.6
O(1)-C(1)-H(1B)	108.6
C(2)-C(1)-H(1B)	108.6
H(1A)-C(1)-H(1B)	107.5
C(7)-C(2)-C(3)	119.5(2)
C(7)-C(2)-C(1)	120.9(2)
C(3)-C(2)-C(1)	119.6(2)
C(4)-C(3)-C(2)	121.0(3)
C(4)-C(3)-H(3)	119.5
C(2)-C(3)-H(3)	119.5
C(5)-C(4)-C(3)	119.6(3)
C(5)-C(4)-H(4)	120.2
C(3)-C(4)-H(4)	120.2
C(4)-C(5)-C(6)	120.2(3)
C(4)-C(5)-H(5)	119.9
C(6)-C(5)-H(5)	119.9
C(5)-C(6)-C(7)	120.9(2)
C(5)-C(6)-H(6)	119.5
C(7)-C(6)-H(6)	119.5

C(2)-C(7)-C(6)	118.9(2)
C(2)-C(7)-C(8)	118.9(2)
C(6)-C(7)-C(8)	122.2(2)
C(7)-C(8)-C(9)	109.49(17)
C(7)-C(8)-C(11)	108.88(17)
C(9)-C(8)-C(11)	111.04(17)
C(7)-C(8)-H(8)	109.1
C(9)-C(8)-H(8)	109.1
C(11)-C(8)-H(8)	109.1
O(2)-C(9)-O(1)	105.04(17)
O(2)-C(9)-C(10)	104.37(19)
O(1)-C(9)-C(10)	112.5(2)
O(2)-C(9)-C(8)	113.02(18)
O(1)-C(9)-C(8)	108.10(18)
C(10)-C(9)-C(8)	113.58(18)
C(9)-C(10)-H(10A)	109.5
C(9)-C(10)-H(10B)	109.5
H(10A)-C(10)-H(10B)	109.5
C(9)-C(10)-H(10C)	109.5
H(10A)-C(10)-H(10C)	109.5
H(10B)-C(10)-H(10C)	109.5
C(19)-C(11)-C(12)	112.50(17)
C(19)-C(11)-C(8)	111.42(17)
C(12)-C(11)-C(8)	111.79(17)
C(19)-C(11)-H(11)	106.9
C(12)-C(11)-H(11)	106.9
C(8)-C(11)-H(11)	106.9
C(13)-C(12)-C(17)	117.3(2)
C(13)-C(12)-C(11)	121.15(19)
C(17)-C(12)-C(11)	121.4(2)
C(12)-C(13)-O(2)	122.75(19)
C(12)-C(13)-C(14)	121.4(2)
O(2)-C(13)-C(14)	115.9(2)
C(15)-C(14)-C(13)	119.5(3)
C(15)-C(14)-H(14)	120.2
C(13)-C(14)-H(14)	120.2
C(14)-C(15)-C(16)	121.4(3)
C(14)-C(15)-H(15)	119.3
C(16)-C(15)-H(15)	119.3

C(17)-C(16)-C(15)	117.5(2)
C(17)-C(16)-C(18)	121.5(3)
C(15)-C(16)-C(18)	121.0(3)
C(16)-C(17)-C(12)	122.9(2)
C(16)-C(17)-H(17)	118.6
C(12)-C(17)-H(17)	118.6
C(16)-C(18)-H(18A)	109.5
C(16)-C(18)-H(18B)	109.5
H(18A)-C(18)-H(18B)	109.5
C(16)-C(18)-H(18C)	109.5
H(18A)-C(18)-H(18C)	109.5
H(18B)-C(18)-H(18C)	109.5
C(24)-C(19)-C(20)	117.9(2)
C(24)-C(19)-C(11)	121.2(2)
C(20)-C(19)-C(11)	120.9(2)
C(21)-C(20)-C(19)	120.9(2)
C(21)-C(20)-H(20)	119.6
C(19)-C(20)-H(20)	119.6
C(22)-C(21)-C(20)	120.7(3)
C(22)-C(21)-H(21)	119.7
C(20)-C(21)-H(21)	119.7
C(21)-C(22)-C(23)	119.3(3)
C(21)-C(22)-H(22)	120.4
C(23)-C(22)-H(22)	120.4
C(24)-C(23)-C(22)	119.9(3)
C(24)-C(23)-H(23)	120.0
C(22)-C(23)-H(23)	120.0
C(19)-C(24)-C(23)	121.4(3)
C(19)-C(24)-H(24)	119.3
C(23)-C(24)-H(24)	119.3

Symmetry transformations used to generate equivalent atoms:

Supplementary Table 6. Anisotropic displacement parameters ($Å^2$x 10^3) for mo_d8v191072_0m. The anisotropic displacement factor exponent takes the form: $-2\pi^2$[h^2 $a^{*2}U^{11}$ + ... + 2 h k a* b* U^{12}]

	U^{11}	U^{22}	U^{33}	U^{23}	U^{13}	U^{12}
O(1)	66(1)	41(1)	55(1)	-1(1)	6(1)	7(1)
O(2)	46(1)	64(1)	59(1)	9(1)	11(1)	3(1)
C(1)	80(2)	53(1)	63(2)	-13(1)	-1(2)	1(1)
C(2)	61(2)	49(1)	47(1)	2(1)	-2(1)	-4(1)
C(3)	79(2)	71(2)	59(2)	-1(1)	-13(1)	-16(2)
C(4)	61(2)	97(2)	65(2)	13(2)	-16(1)	-5(2)
C(5)	55(2)	85(2)	68(2)	15(2)	1(1)	13(1)
C(6)	54(2)	56(1)	54(1)	4(1)	3(1)	6(1)
C(7)	53(1)	40(1)	41(1)	5(1)	4(1)	1(1)
C(8)	47(1)	33(1)	46(1)	-1(1)	6(1)	-2(1)
C(9)	53(1)	41(1)	50(1)	2(1)	7(1)	3(1)
C(10)	74(2)	61(1)	58(2)	11(1)	18(1)	5(1)
C(11)	43(1)	38(1)	45(1)	-1(1)	4(1)	-4(1)
C(12)	46(1)	33(1)	50(1)	-2(1)	0(1)	0(1)
C(13)	49(1)	39(1)	56(1)	1(1)	4(1)	-1(1)
C(14)	46(1)	56(1)	76(2)	-1(1)	4(1)	4(1)
C(15)	56(2)	56(1)	82(2)	-2(1)	-16(2)	5(1)
C(16)	63(2)	48(1)	60(2)	-5(1)	-13(1)	4(1)
C(17)	55(1)	42(1)	52(1)	-2(1)	-1(1)	1(1)
C(18)	101(3)	87(2)	68(2)	9(2)	-22(2)	15(2)
C(19)	45(1)	48(1)	41(1)	2(1)	0(1)	3(1)
C(20)	47(1)	64(1)	60(2)	-14(1)	1(1)	0(1)
C(21)	63(2)	69(2)	60(2)	-19(1)	-8(1)	14(1)
C(22)	76(2)	89(2)	53(2)	-5(2)	14(1)	29(2)
C(23)	76(2)	87(2)	86(2)	5(2)	38(2)	2(2)
C(24)	61(2)	59(1)	69(2)	7(1)	20(1)	-3(1)

Supplementary Table 7. Hydrogen coordinates (x 10^4) and isotropic displacement parameters (Å^{2}x 10 ^{3}) for mo_d8v191072_0m.

	x	y	z	U(eq)
H(1A)	6678	6477	8740	79
H(1B)	7205	7472	8182	79
H(3)	9517	6517	8659	83
H(4)	11318	5018	8439	89
H(5)	10926	3121	7778	83
H(6)	8760	2757	7315	65
H(8)	6217	3300	7301	50
H(10A)	5905	4160	8706	97
H(10B)	4816	3204	8353	97
H(10C)	4323	4569	8694	97
H(11)	6962	5868	6737	50
H(14)	1801	5653	6901	71
H(15)	1519	6419	5830	77
H(17)	5562	5804	5480	60
H(18A)	3310	7775	4868	128
H(18B)	2495	6448	4657	128
H(18C)	4121	6513	4577	128
H(20)	5999	2548	6123	68
H(21)	7250	1309	5355	77
H(22)	9310	2148	4936	87
H(23)	10140	4246	5305	100
H(24)	8893	5483	6080	76

Supplementary Table 8. Torsion angles [°] for mo_d8v191072_0m.

C(9)-O(1)-C(1)-C(2)	38.3(3)
O(1)-C(1)-C(2)-C(7)	-7.8(3)
O(1)-C(1)-C(2)-C(3)	173.9(2)
C(7)-C(2)-C(3)-C(4)	0.2(4)
C(1)-C(2)-C(3)-C(4)	178.6(2)
C(2)-C(3)-C(4)-C(5)	-1.1(4)
C(3)-C(4)-C(5)-C(6)	1.0(4)
C(4)-C(5)-C(6)-C(7)	0.0(4)
C(3)-C(2)-C(7)-C(6)	0.8(3)
C(1)-C(2)-C(7)-C(6)	-177.6(2)
C(3)-C(2)-C(7)-C(8)	-175.9(2)
C(1)-C(2)-C(7)-C(8)	5.7(3)
C(5)-C(6)-C(7)-C(2)	-0.9(3)
C(5)-C(6)-C(7)-C(8)	175.7(2)
C(2)-C(7)-C(8)-C(9)	-30.8(3)
C(6)-C(7)-C(8)-C(9)	152.65(19)
C(2)-C(7)-C(8)-C(11)	90.8(2)
C(6)-C(7)-C(8)-C(11)	-85.8(2)
C(13)-O(2)-C(9)-O(1)	73.3(2)
C(13)-O(2)-C(9)-C(10)	-168.20(19)
C(13)-O(2)-C(9)-C(8)	-44.3(3)
C(1)-O(1)-C(9)-O(2)	174.48(17)
C(1)-O(1)-C(9)-C(10)	61.6(3)
C(1)-O(1)-C(9)-C(8)	-64.6(2)
C(7)-C(8)-C(9)-O(2)	174.43(17)
C(11)-C(8)-C(9)-O(2)	54.2(2)
C(7)-C(8)-C(9)-O(1)	58.6(2)
C(11)-C(8)-C(9)-O(1)	-61.7(2)
C(7)-C(8)-C(9)-C(10)	-66.9(2)
C(11)-C(8)-C(9)-C(10)	172.8(2)
C(7)-C(8)-C(11)-C(19)	74.0(2)
C(9)-C(8)-C(11)-C(19)	-165.38(18)
C(7)-C(8)-C(11)-C(12)	-159.17(17)
C(9)-C(8)-C(11)-C(12)	-38.5(2)
C(19)-C(11)-C(12)-C(13)	141.3(2)
C(8)-C(11)-C(12)-C(13)	15.0(3)
C(19)-C(11)-C(12)-C(17)	-43.4(3)

C(8)-C(11)-C(12)-C(17)	-169.66(18)
C(17)-C(12)-C(13)-O(2)	-179.31(19)
C(11)-C(12)-C(13)-O(2)	-3.8(3)
C(17)-C(12)-C(13)-C(14)	1.6(3)
C(11)-C(12)-C(13)-C(14)	177.2(2)
C(9)-O(2)-C(13)-C(12)	18.7(3)
C(9)-O(2)-C(13)-C(14)	-162.1(2)
C(12)-C(13)-C(14)-C(15)	-2.4(4)
O(2)-C(13)-C(14)-C(15)	178.4(2)
C(13)-C(14)-C(15)-C(16)	1.0(4)
C(14)-C(15)-C(16)-C(17)	1.0(4)
C(14)-C(15)-C(16)-C(18)	-176.3(3)
C(15)-C(16)-C(17)-C(12)	-1.9(3)
C(18)-C(16)-C(17)-C(12)	175.5(2)
C(13)-C(12)-C(17)-C(16)	0.6(3)
C(11)-C(12)-C(17)-C(16)	-174.9(2)
C(12)-C(11)-C(19)-C(24)	120.3(2)
C(8)-C(11)-C(19)-C(24)	-113.2(2)
C(12)-C(11)-C(19)-C(20)	-59.4(3)
C(8)-C(11)-C(19)-C(20)	67.0(3)
C(24)-C(19)-C(20)-C(21)	-0.9(4)
C(11)-C(19)-C(20)-C(21)	178.8(2)
C(19)-C(20)-C(21)-C(22)	0.1(4)
C(20)-C(21)-C(22)-C(23)	0.6(4)
C(21)-C(22)-C(23)-C(24)	-0.4(5)
C(20)-C(19)-C(24)-C(23)	1.1(4)
C(11)-C(19)-C(24)-C(23)	-178.6(2)
C(22)-C(23)-C(24)-C(19)	-0.5(5)

Symmetry transformations used to generate equivalent atoms:

Supplementary Table 9. Hydrogen bonds for mo_d8v191072_0m [Å and °].

D-H...A	d(D-H)	d(H...A)	d(D...A)	<(DHA)
C(8)-H(8)...O(1)#1	0.98	2.65	3.524(3)	149.1

Symmetry transformations used to generate equivalent atoms:

#1 -x+1,y-1/2,-z+3/2

7. 1H NMR and 13C NMR spectra

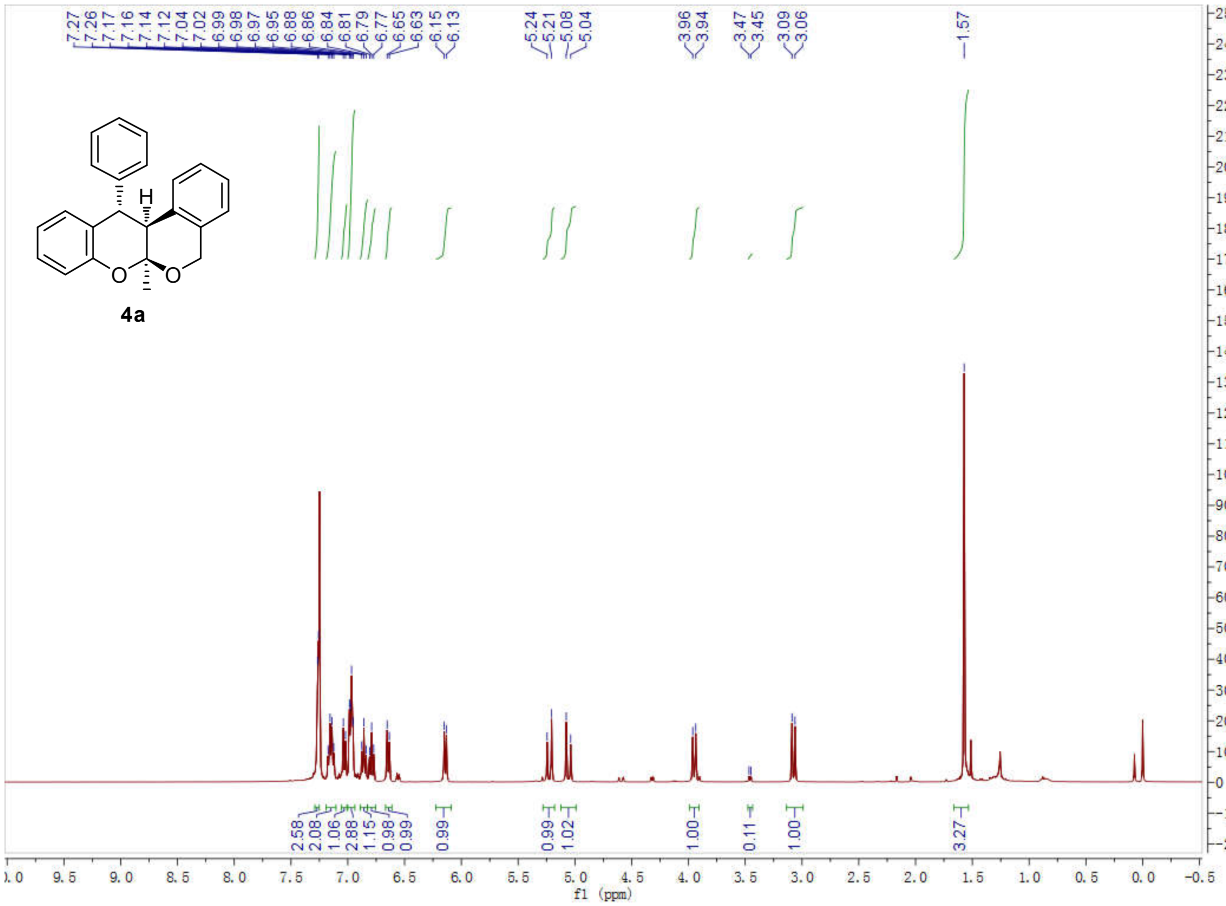

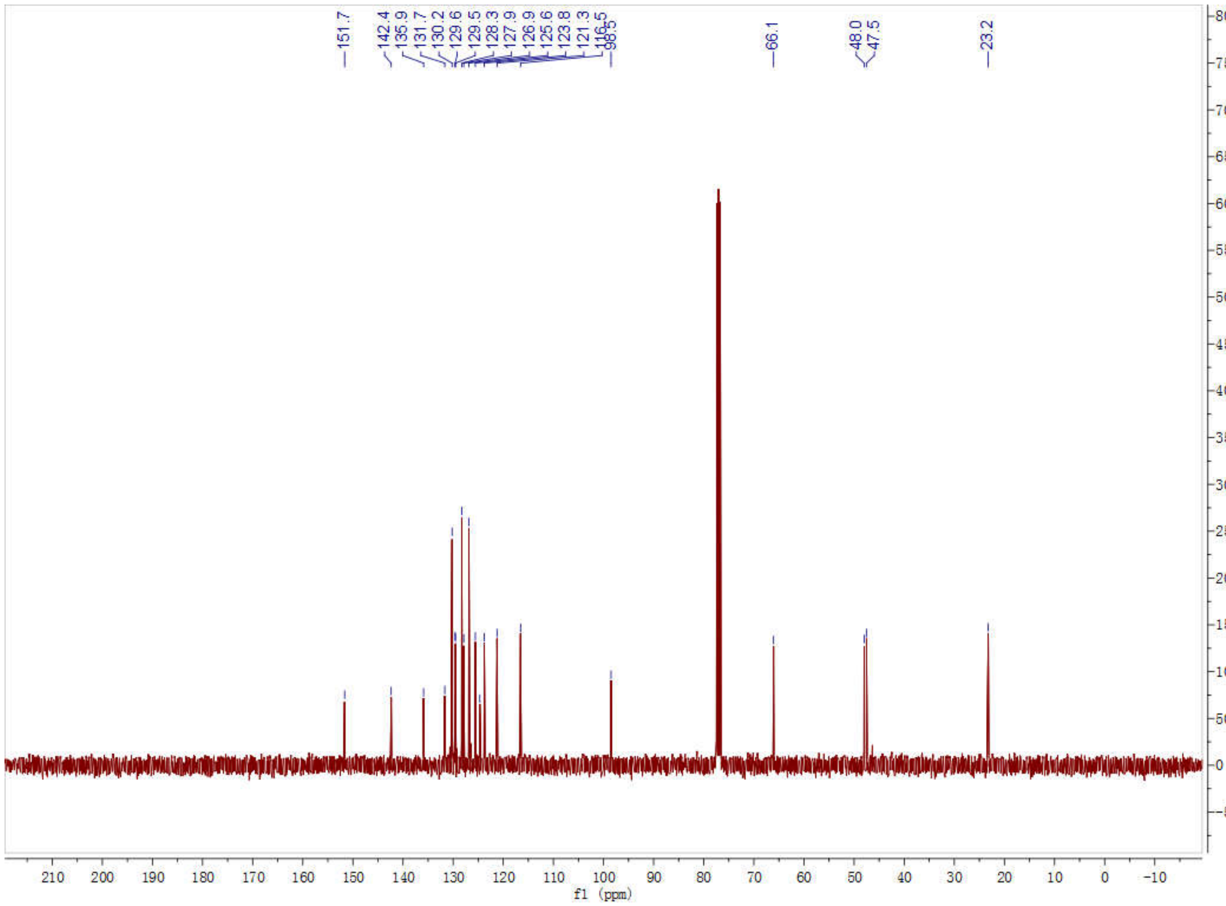

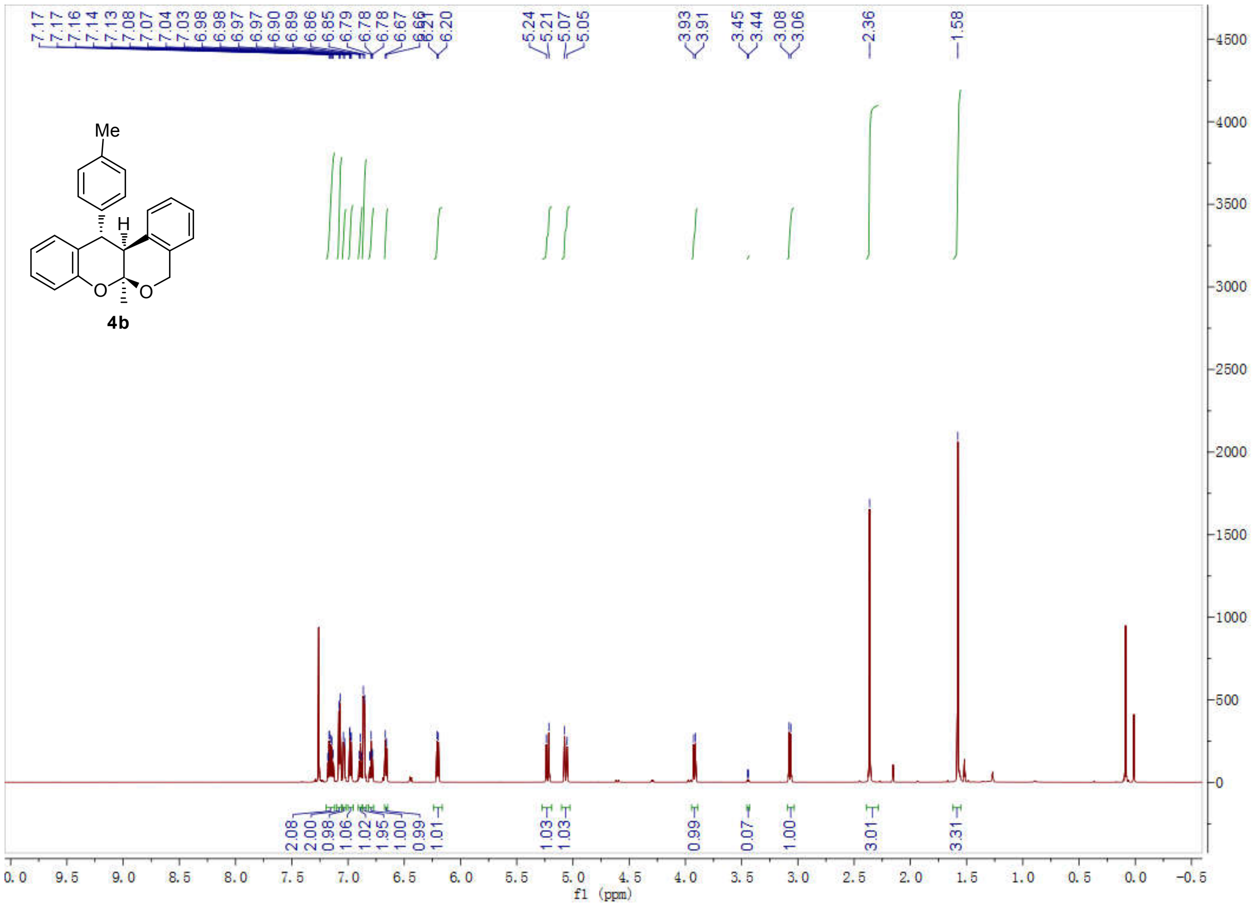
7.17 7.17 7.16 7.14 7.13 7.08 7.07 7.04 7.03 6.98 6.98 6.97 6.97 6.90 6.89 6.86 6.85 6.79 6.78 6.78 6.67 6.66 6.21 6.20
5.24 5.21 5.07 5.05
3.93 3.91 3.45 3.44 3.08 3.06
2.36
1.58
Me
H
O
O
4b
2.08 2.00 0.98 1.06 1.02 1.95 1.00 0.99 1.01
1.03 1.03
0.99 0.07 1.00 3.01 3.31
f1 (ppm)

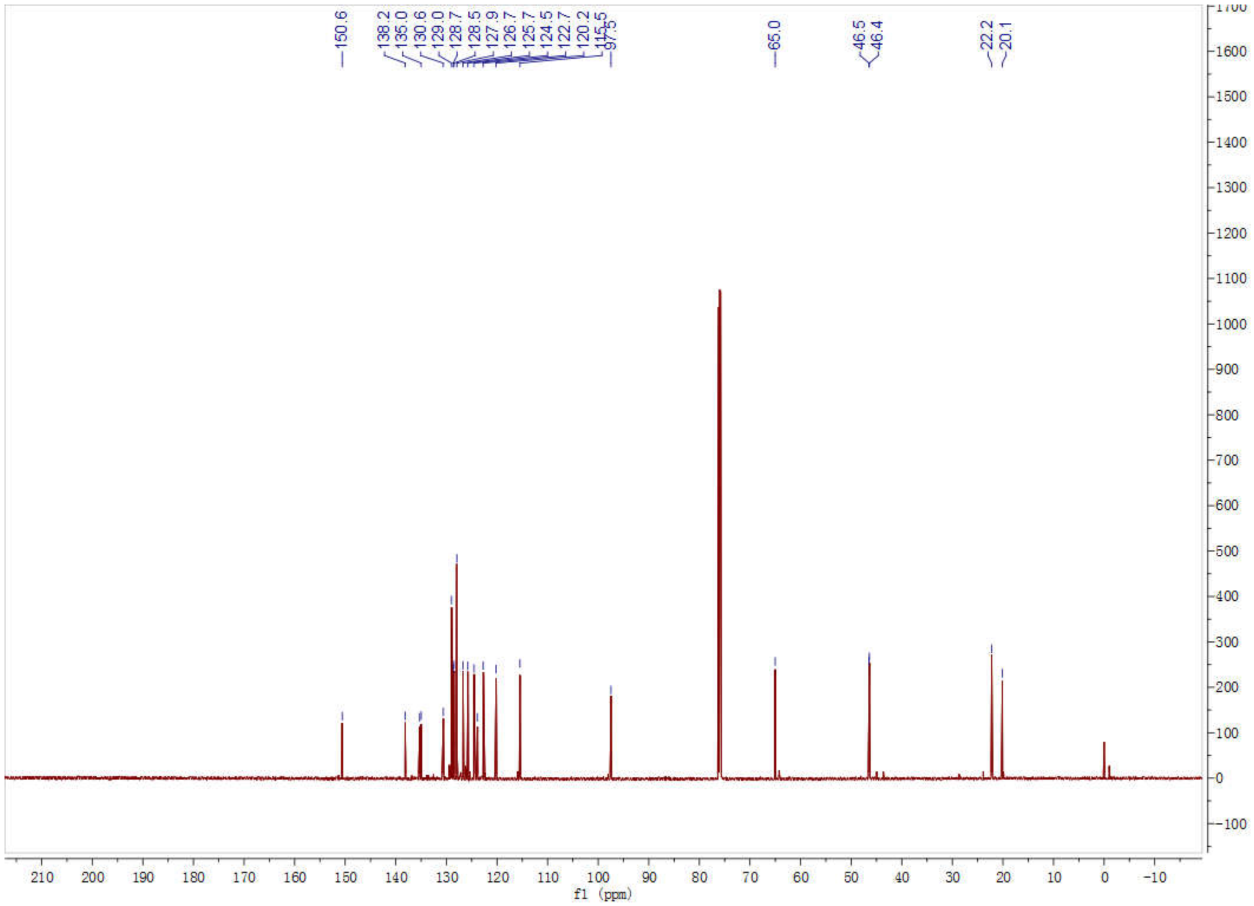
150.6
138.2 135.0 130.6 129.0 128.7 128.5 127.9 126.7 125.7 124.5 122.7 120.2 115.5 97.5
65.0
46.5 46.4
22.2 20.1
f1 (ppm)

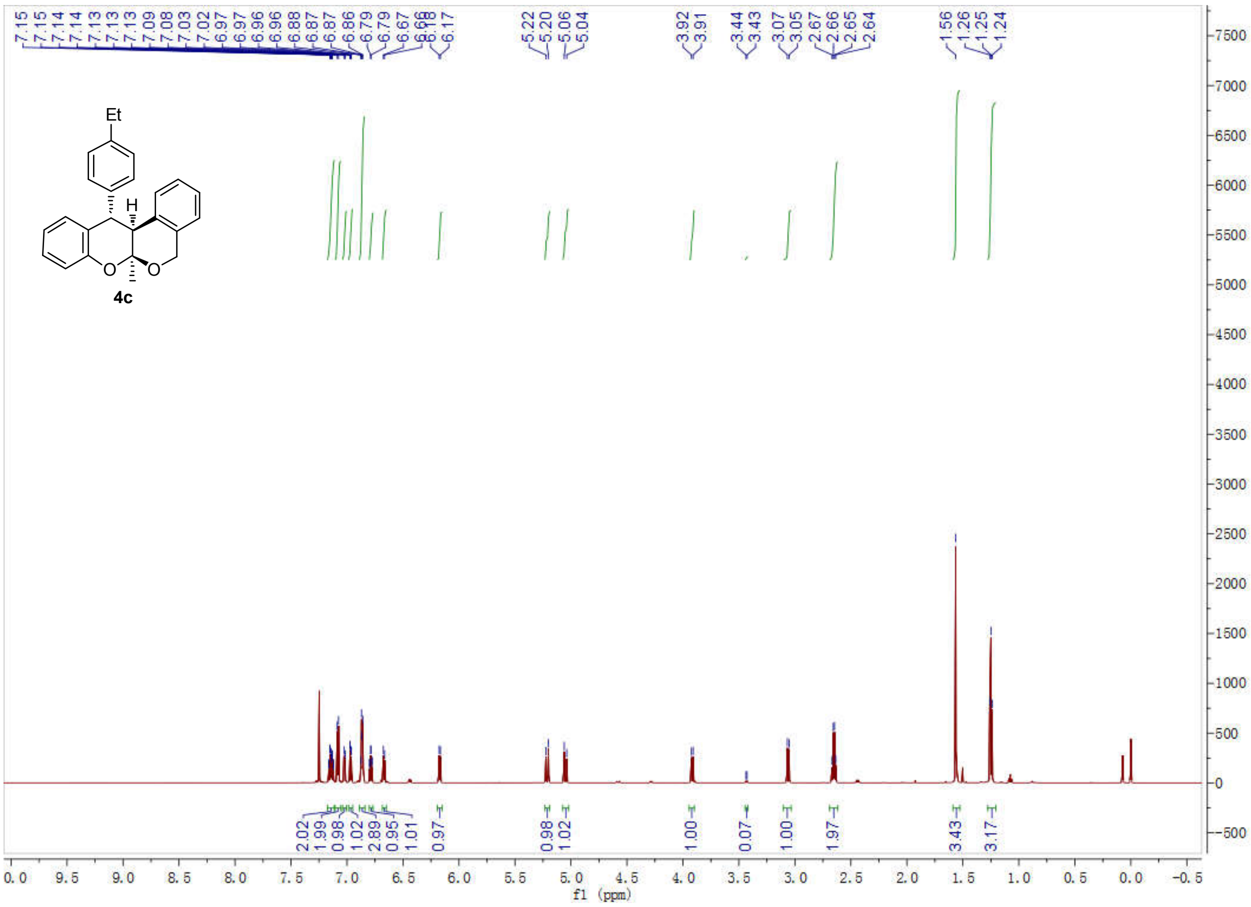
7.15 7.15 7.14 7.14 7.13 7.13 7.13 7.09 7.08 7.03 7.02 6.97 6.97 6.96 6.96 6.88 6.87 6.87 6.86 6.79 6.79 6.67 6.66 6.18 6.17
5.22 5.20 5.06 5.04
3.92 3.91 3.44 3.43 3.07 3.05 2.67 2.66 2.65 2.64
1.56 1.26 1.25 1.24
Et
H
O
O
4c
2.02 1.99 0.98 1.02 2.89 0.95 1.01 0.97 0.98 1.02 1.00 0.07 1.00 1.97 3.43 3.17
f1 (ppm)

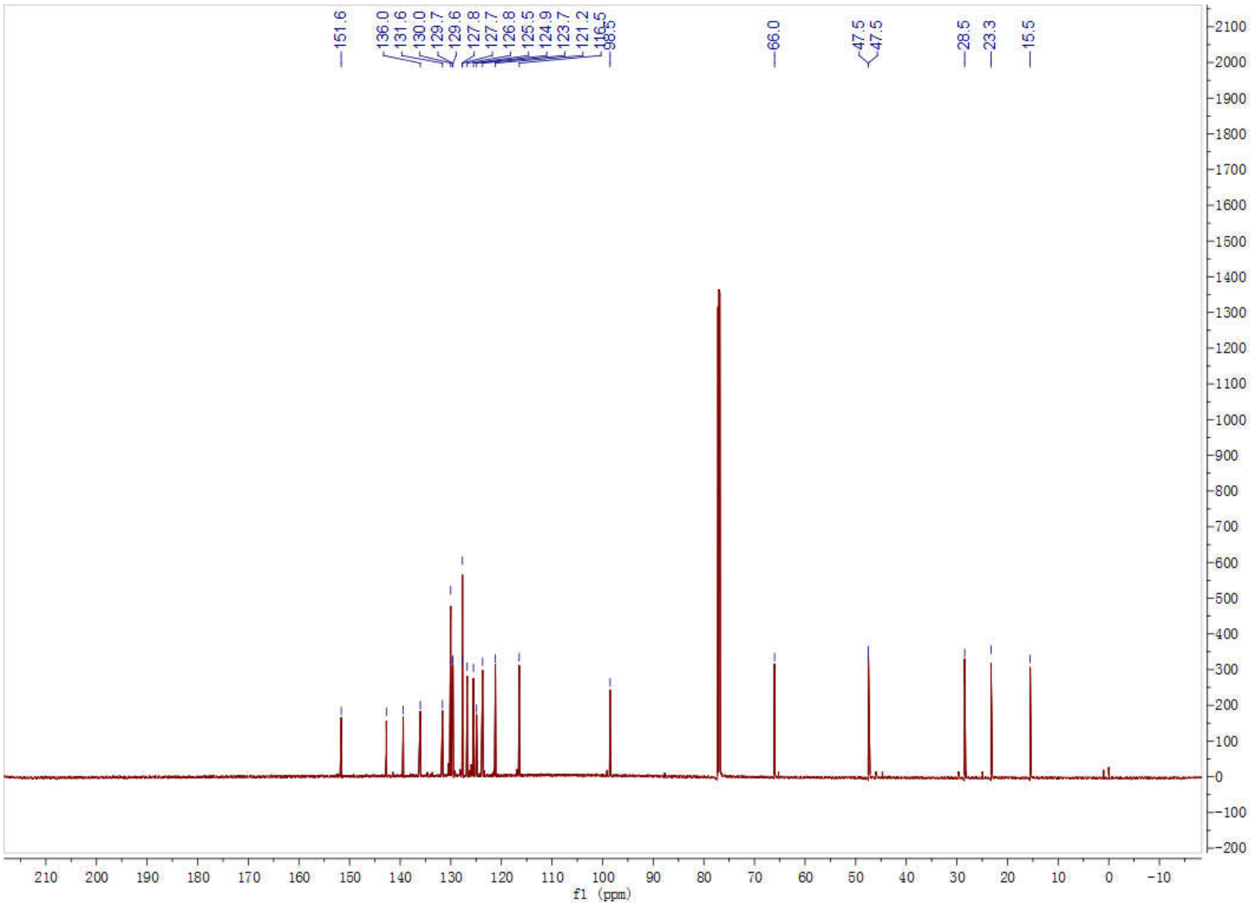
151.6
136.0 131.6 130.0 129.7 129.6 127.8 127.7 126.8 125.5 124.9 123.7 121.2 116.5 98.5
66.0
47.5 47.5
28.5 23.3 15.5
f1 (ppm)

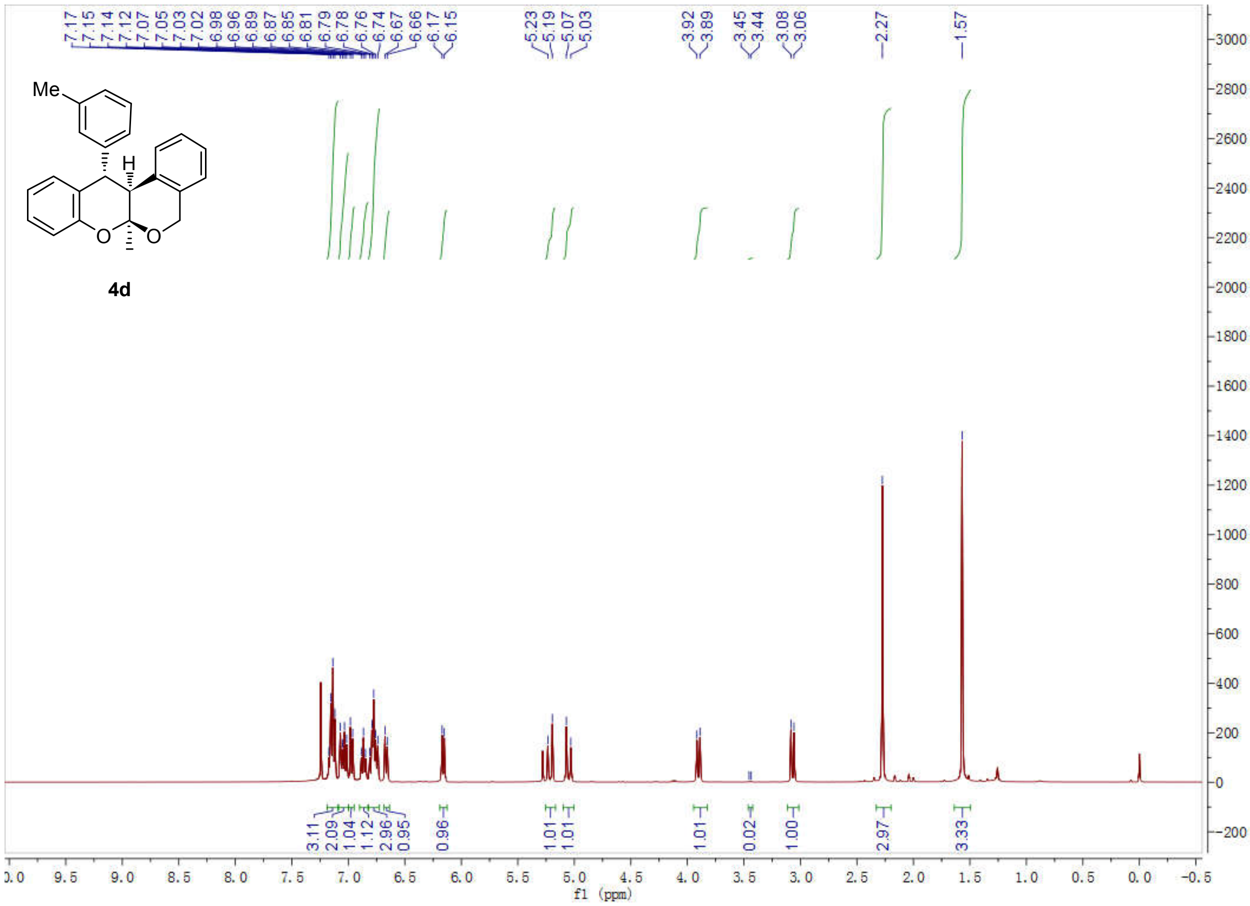
Me
H
O
O
4d
7.17
7.15
7.14
7.12
7.07
7.05
7.03
7.02
6.98
6.96
6.89
6.87
6.85
6.81
6.79
6.78
6.76
6.74
6.67
6.66
6.17
6.15
5.23
5.19
5.07
5.03
3.92
3.89
3.45
3.44
3.08
3.06
2.27
1.57
3.11
2.09
1.04
1.12
2.96
0.95
0.96
1.01
1.01
1.01
0.02
1.00
2.97
3.33
f1 (ppm)

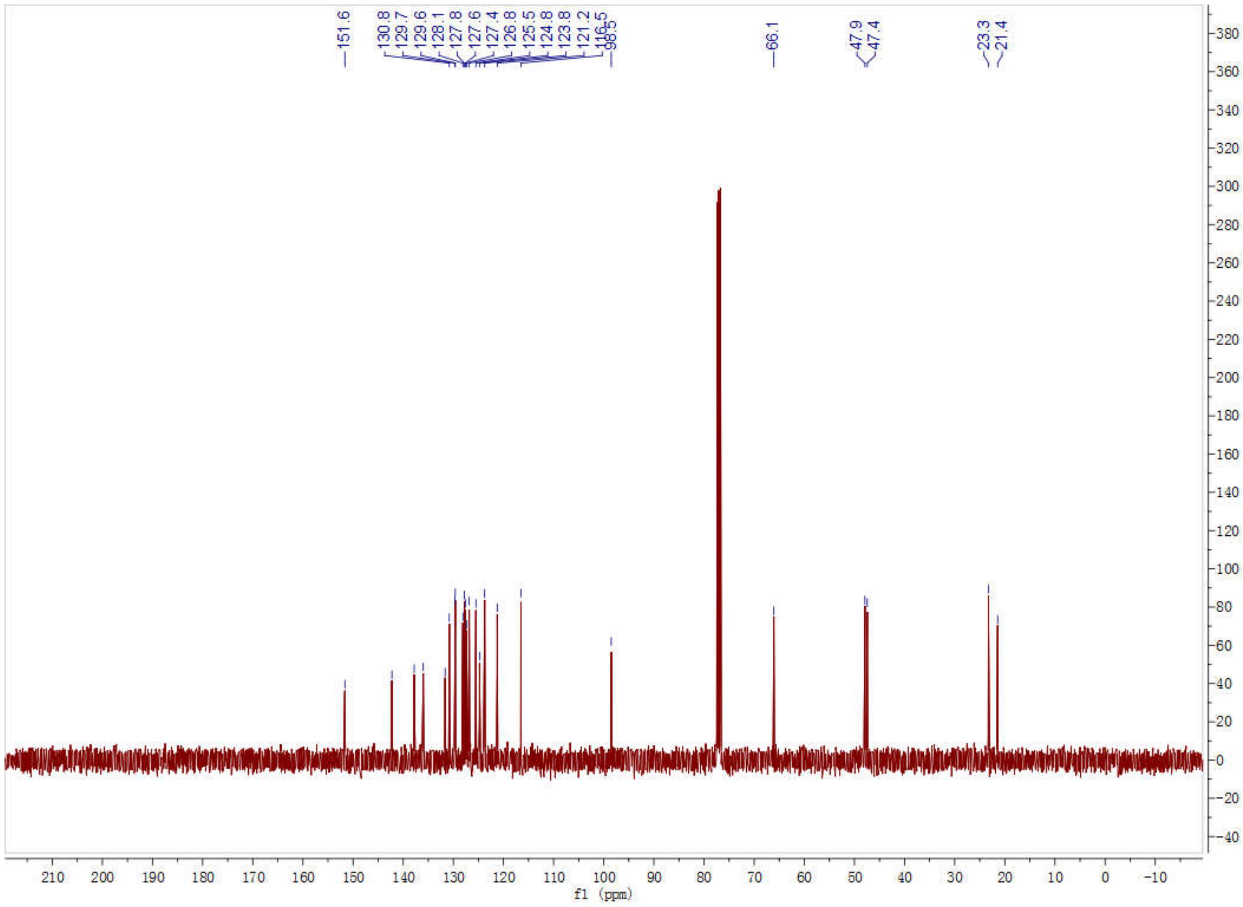
151.6
130.8
129.7
129.6
128.1
127.8
127.6
127.4
126.8
125.5
124.8
123.8
121.2
116.5
98.5
66.1
47.9
47.4
23.3
21.4
f1 (ppm)

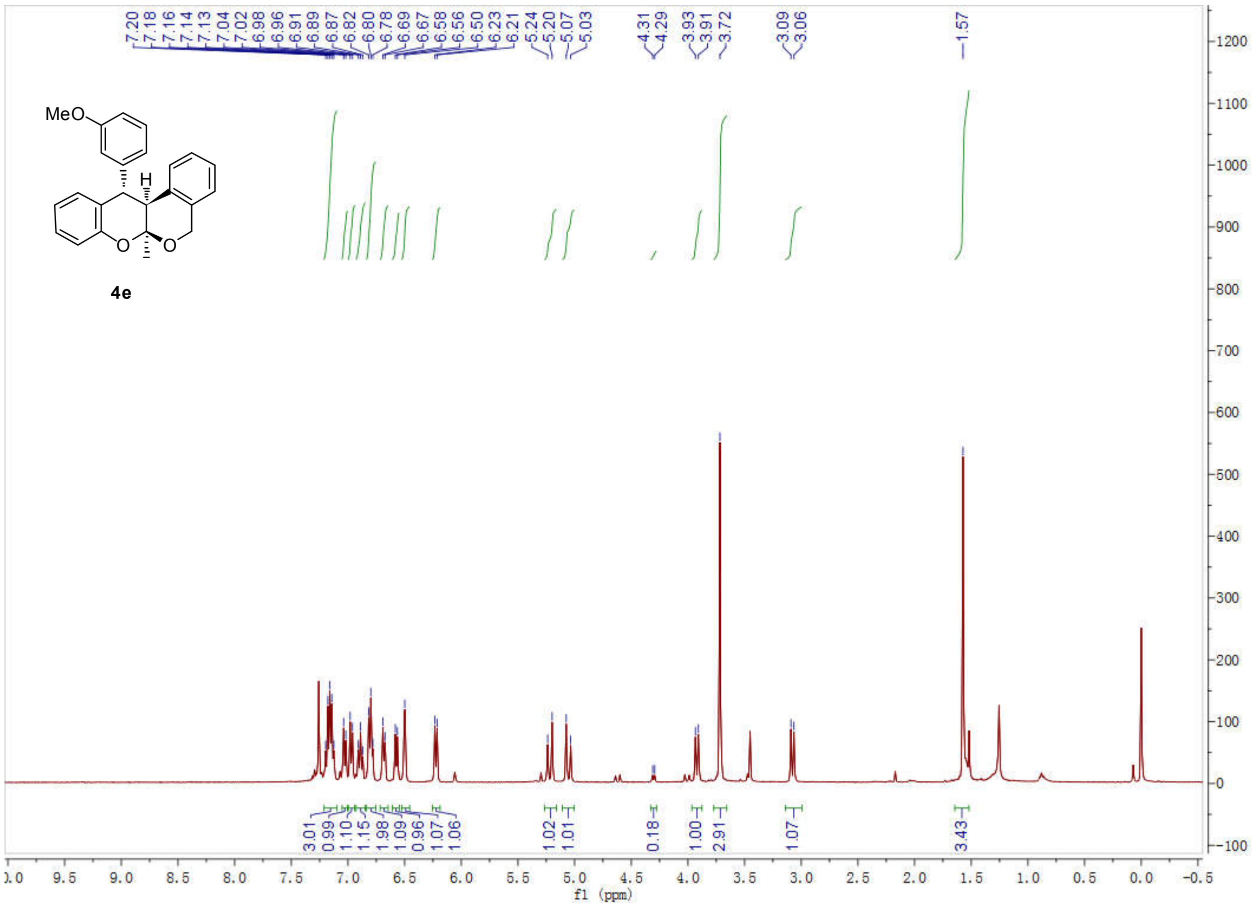
7.20 7.18 7.16 7.14 7.13 7.04 7.02 6.98 6.96 6.91 6.89 6.87 6.82 6.80 6.78 6.69 6.67 6.58 6.56 6.50 6.23 6.21 5.24 5.20 5.07 5.03 4.31 4.29 3.93 3.91 3.72 3.09 3.06 1.57
MeO
H
O
O
4e
3.01 0.99 1.10 1.15 1.98 1.09 0.96 1.07 1.06 1.02 1.01 0.18 1.00 2.91 1.07 3.43
f1 (ppm)

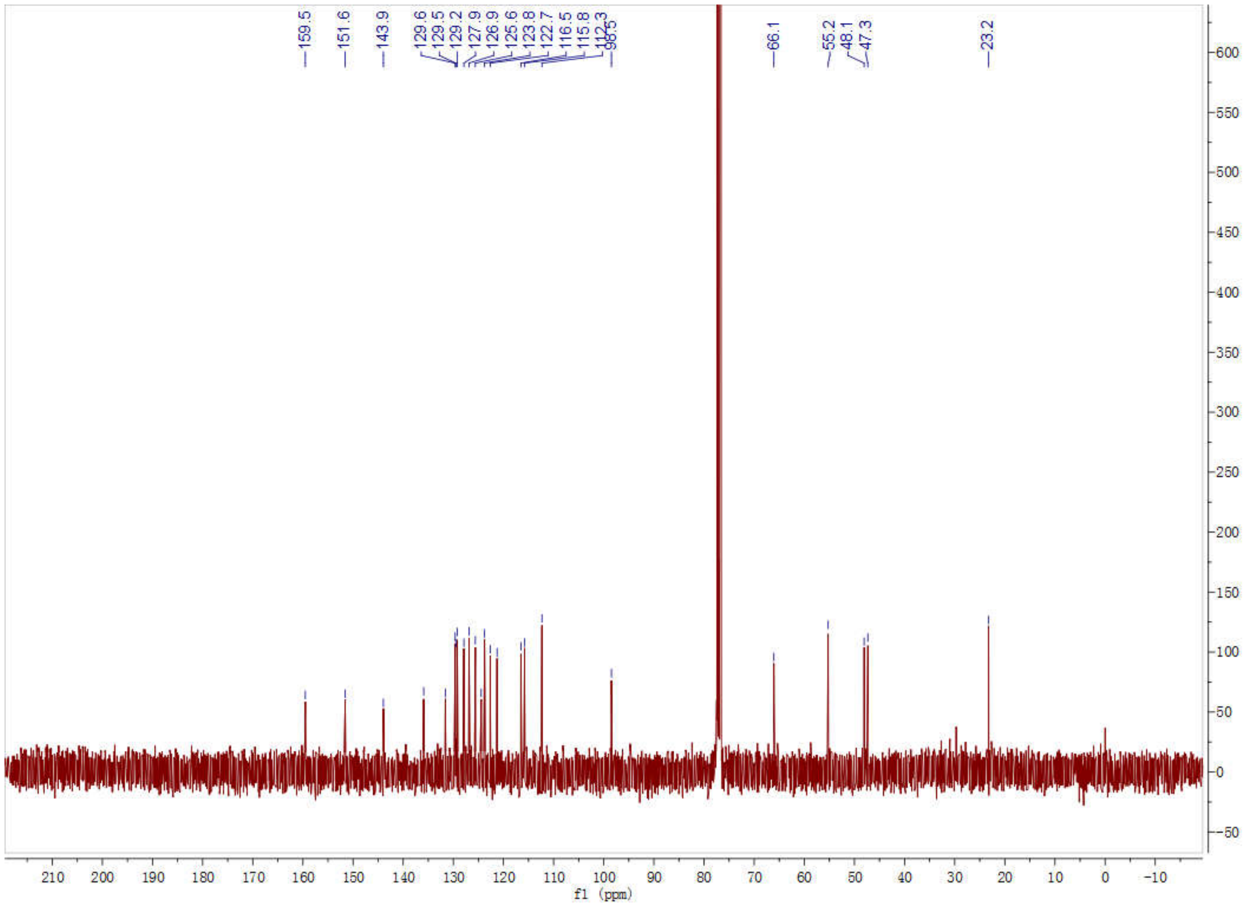
159.5 151.6 143.9 129.6 129.5 129.2 127.9 126.9 125.6 123.8 122.7 116.5 115.8 112.3 98.5 66.1 55.2 48.1 47.3 23.2
f1 (ppm)

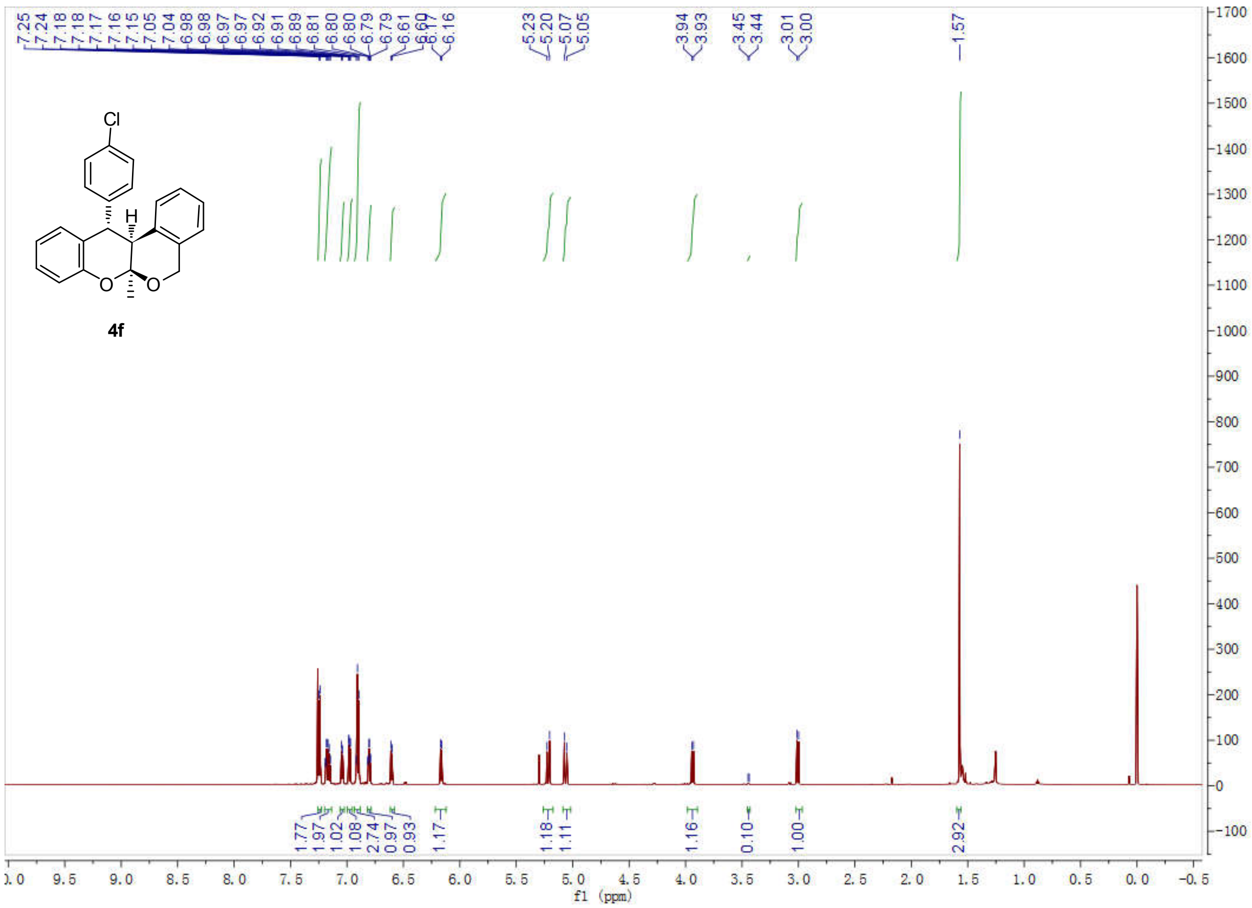

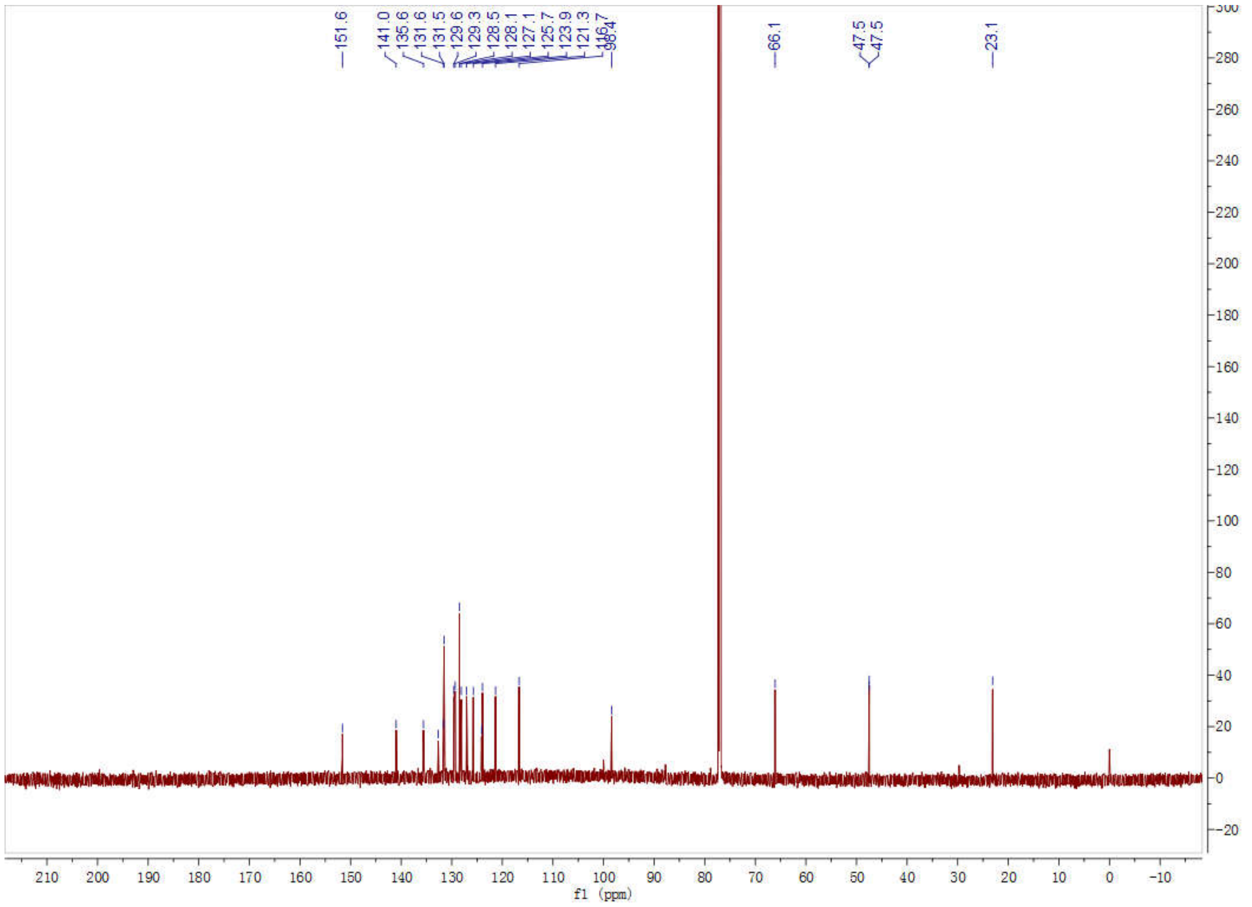

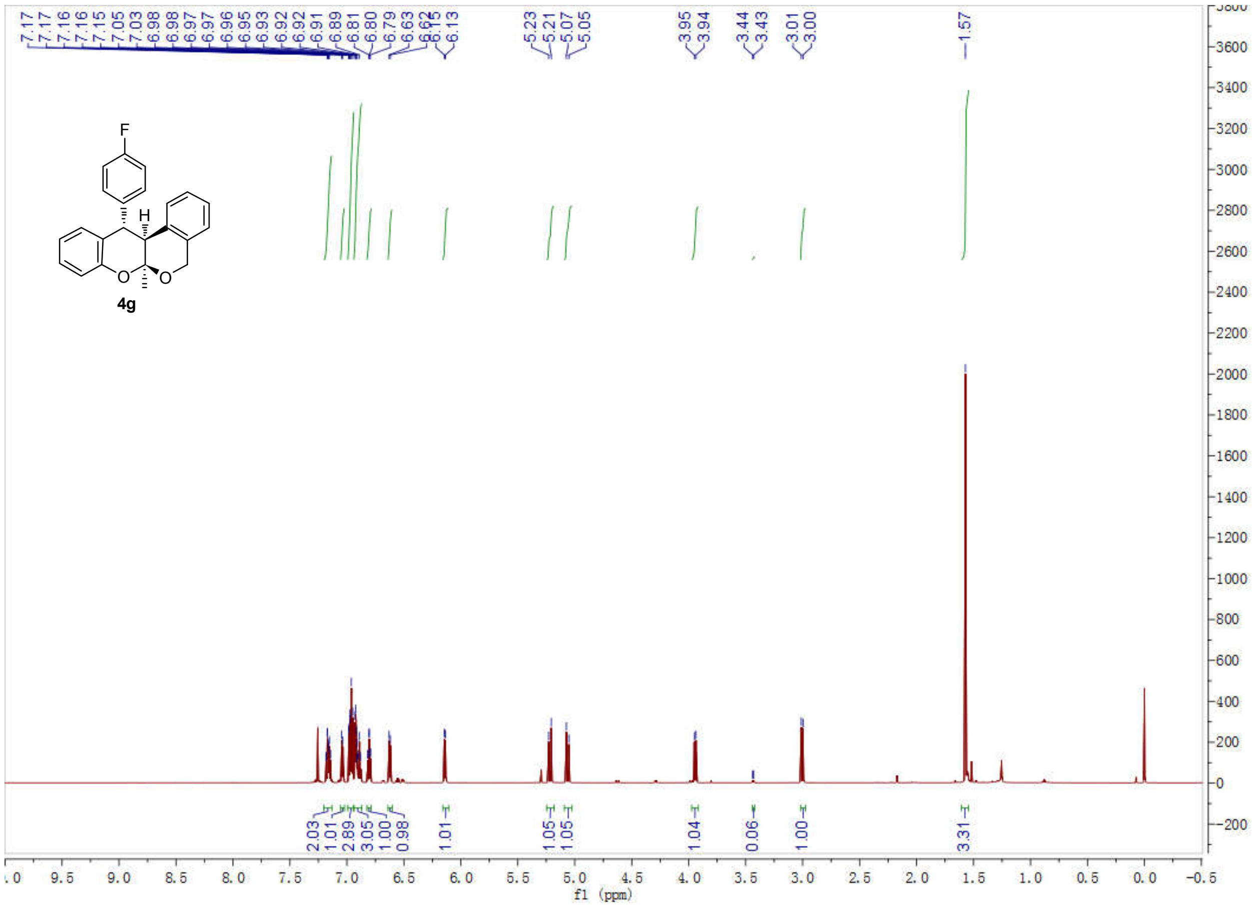
7.17 7.17 7.16 7.15 7.05 7.03 6.98 6.98 6.97 6.97 6.96 6.95 6.93 6.92 6.92 6.91 6.89 6.81 6.80 6.79 6.63 6.62 6.15 6.13
5.23 5.21 5.07 5.05
3.95 3.94
3.44 3.43
3.01 3.00
1.57
F
H
O
O
4g
2.03 1.01 2.89 3.05 1.00 0.98 1.01 1.05 1.05 1.04 0.06 1.00 3.31
f1 (ppm)

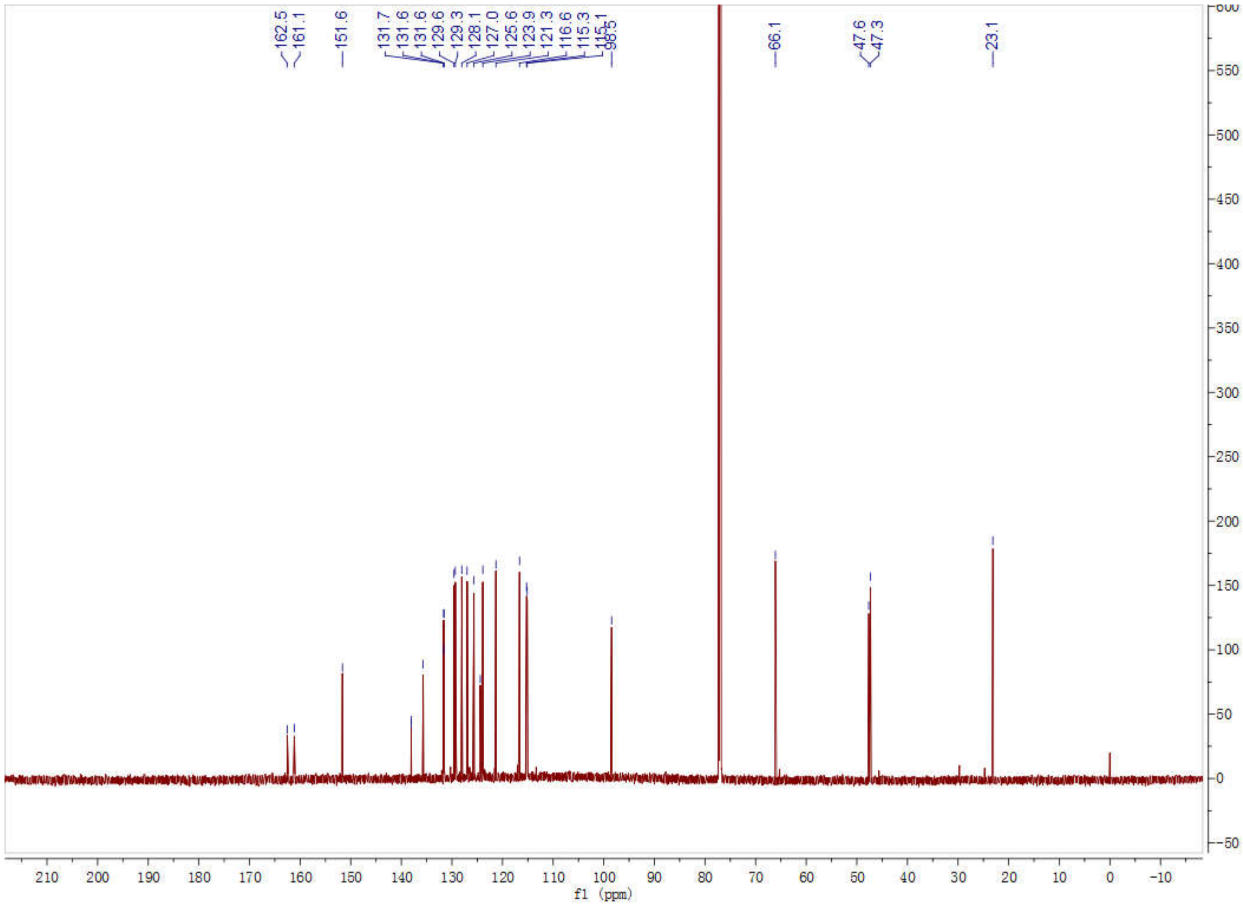
162.5 161.1
151.6
131.7 131.6 131.6 129.6 129.3 128.1 127.0 125.6 123.9 121.3 116.6 115.3 115.1 98.5
66.1
47.6 47.3
23.1
f1 (ppm)

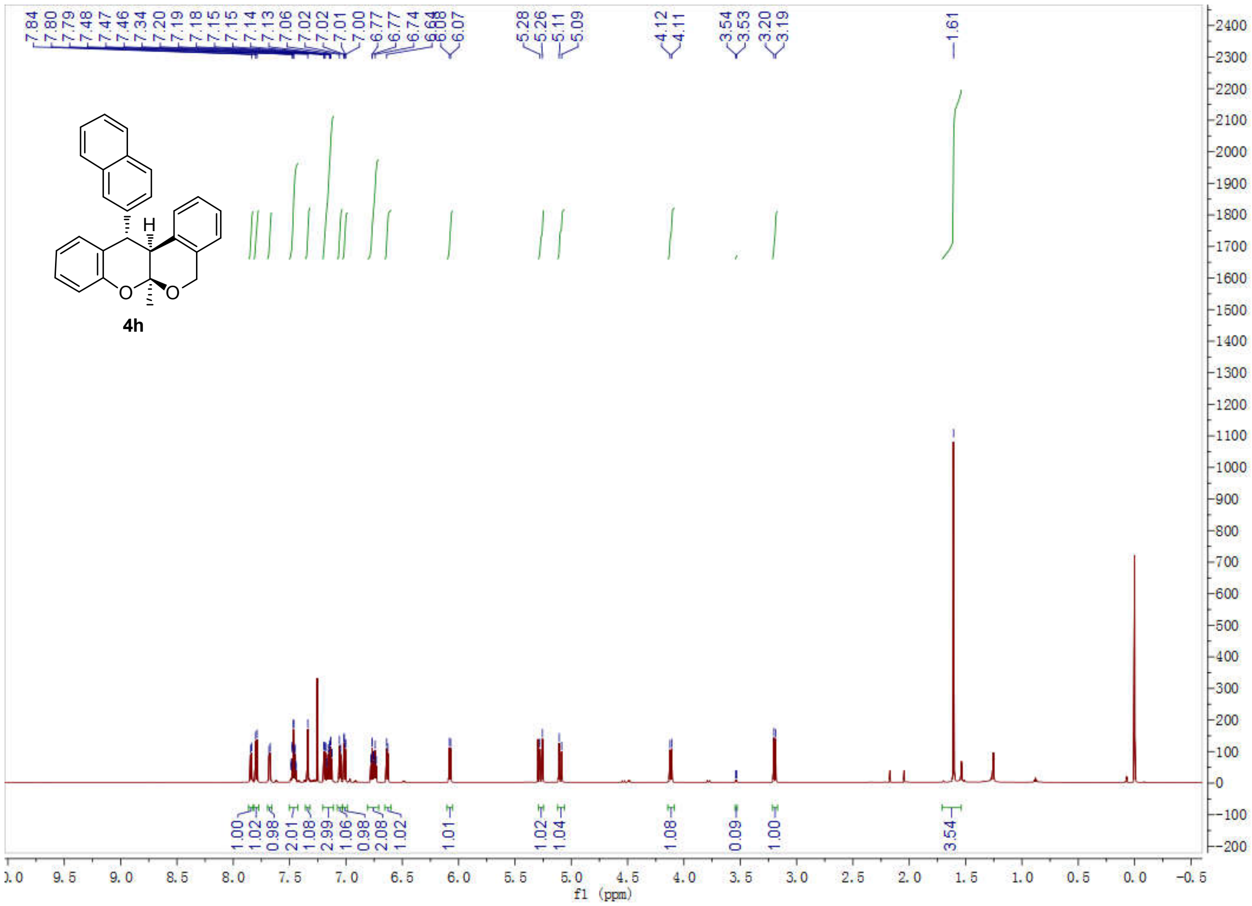
7.84 7.80 7.79 7.48 7.47 7.46 7.34 7.20 7.19 7.18 7.15 7.14 7.13 7.06 7.02 7.02 7.01 7.00 6.77 6.77 6.74 6.07
5.28 5.26 5.11 5.09
4.12 4.11
3.54 3.53 3.20 3.19
1.61
4h
1.00 1.02 0.98 2.01 1.08 2.99 1.06 0.98 2.08 1.02 1.01 1.02 1.04 1.08 0.09 1.00 3.54
f1 (ppm)

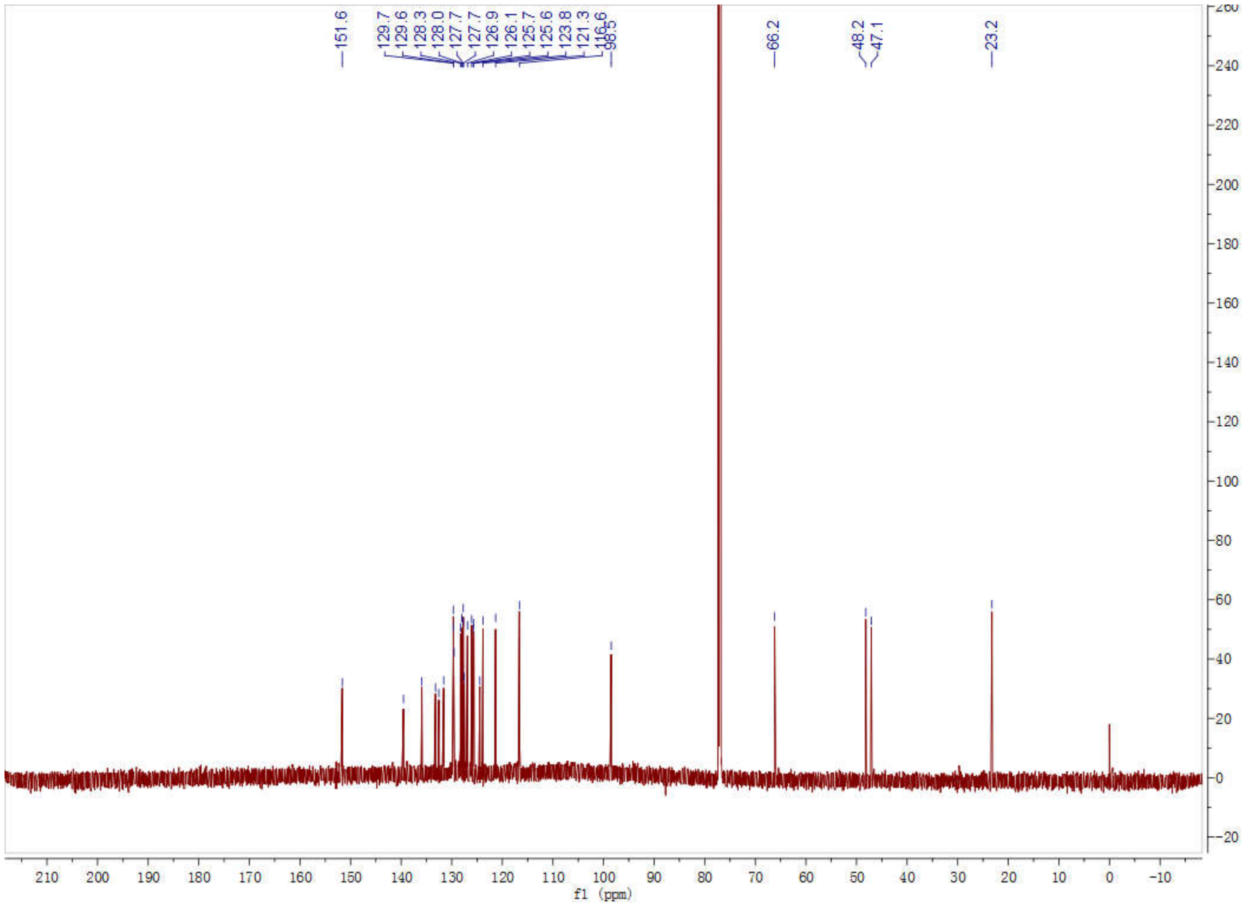
151.6
129.7 129.6 128.3 128.0 127.7 127.7 126.9 126.1 125.7 125.6 123.8 121.3 116.6 98.5
66.2
48.2 47.1
23.2
f1 (ppm)

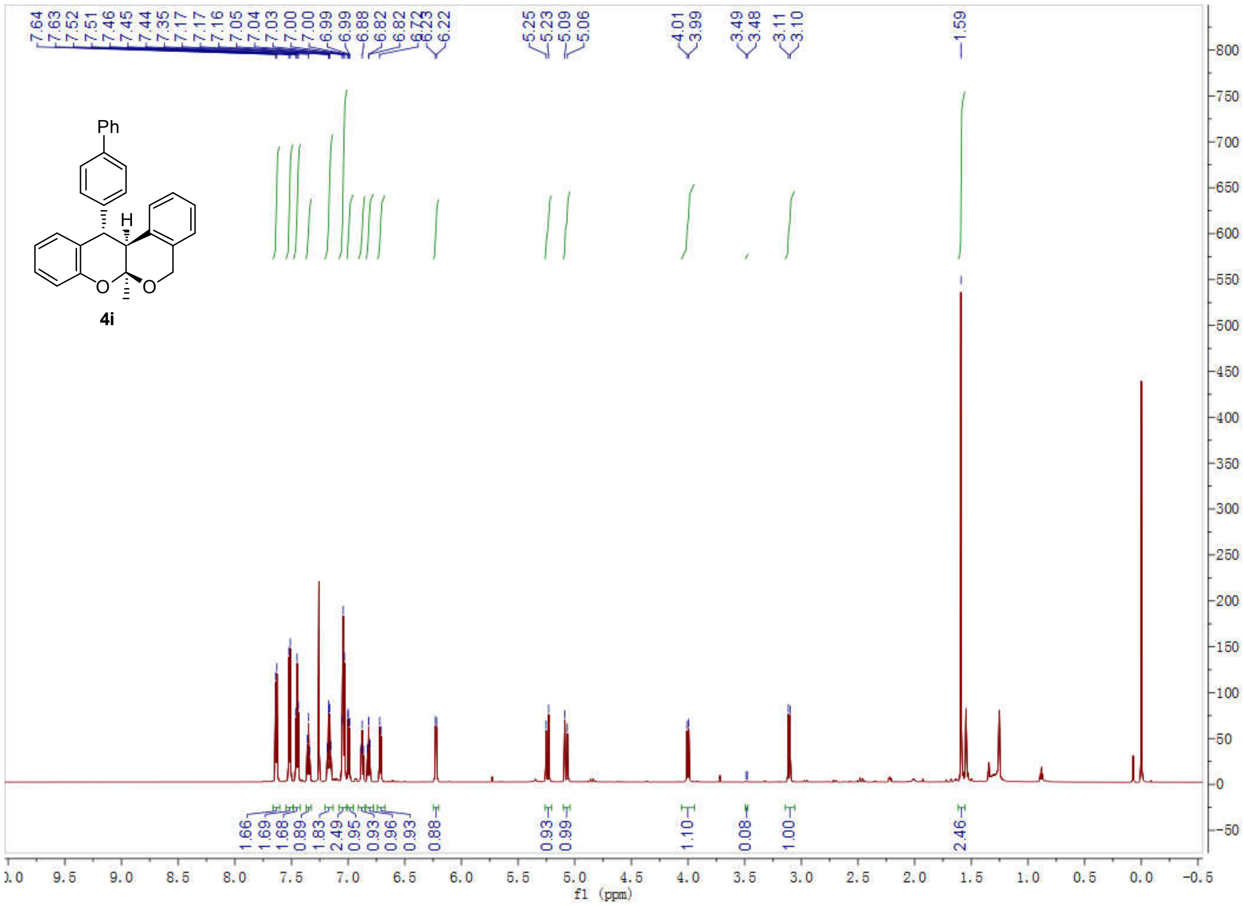

7.64
7.63
7.51
7.46
7.45
7.44
7.35
7.17
7.16
7.05
7.04
7.03
7.00
6.99
6.99
6.88
6.82
6.82
6.73
6.22
5.25
5.23
5.09
5.06
4.01
3.99
3.49
3.48
3.11
3.10
1.59
Ph
H
O
O
4i
1.66
1.69
1.68
0.89
1.83
2.49
0.95
0.93
0.96
0.93
0.88
0.93
0.99
1.10
0.08
1.00
2.46
f1 (ppm)

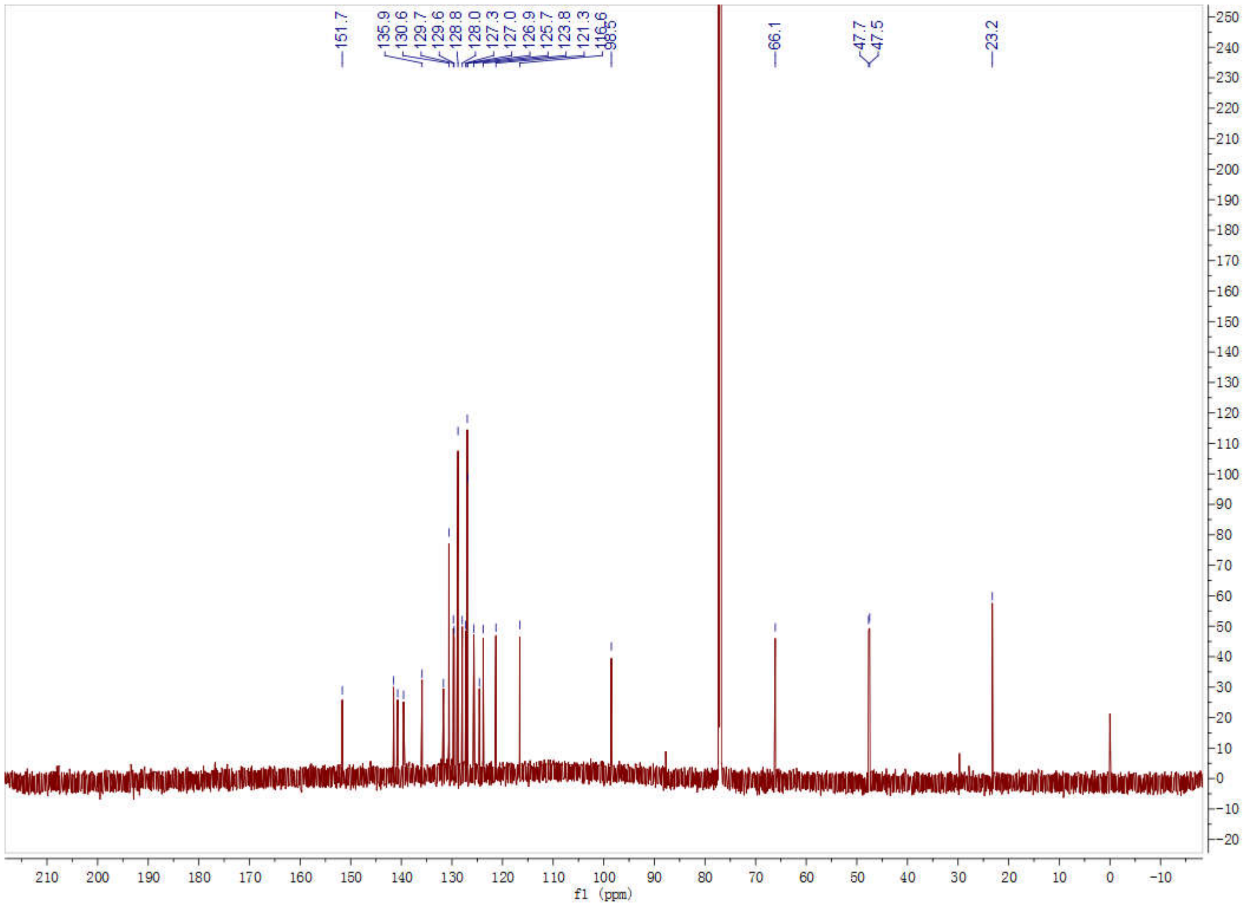

151.7
135.9
130.6
129.7
129.6
128.8
128.0
127.3
127.0
126.9
125.7
123.8
121.3
116.6
98.5
66.1
47.7
47.5
23.2
f1 (ppm)

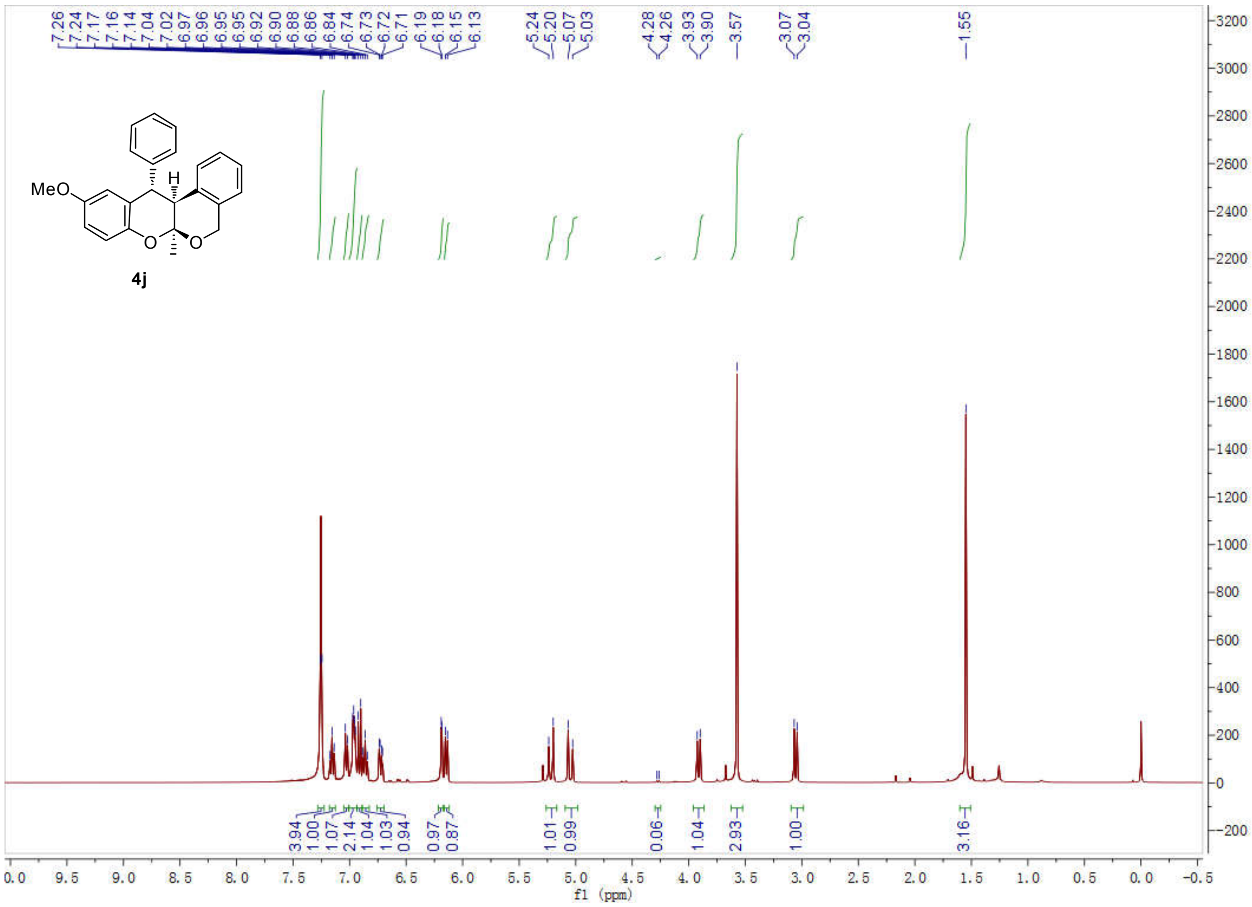
4j
MeO
f1 (ppm)

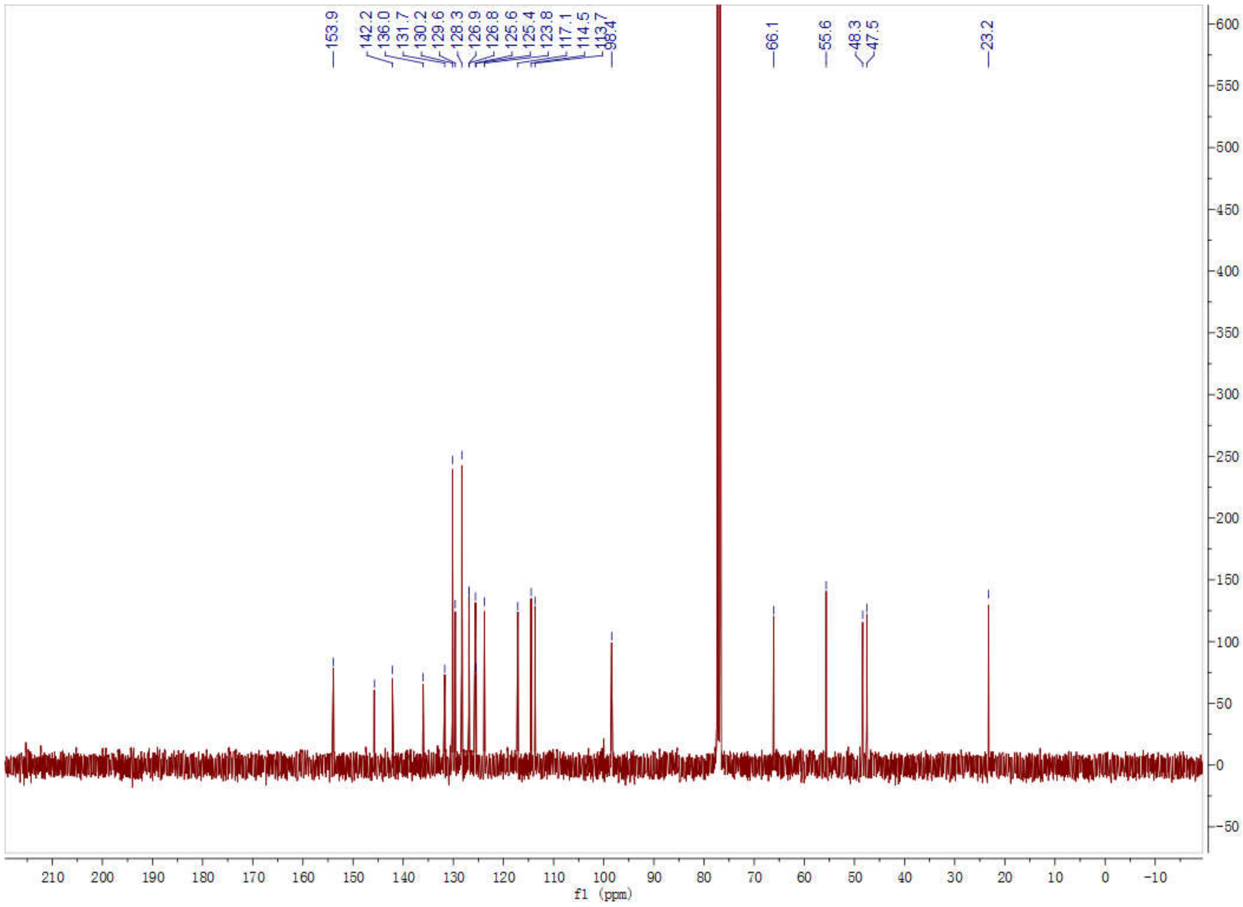
f1 (ppm)

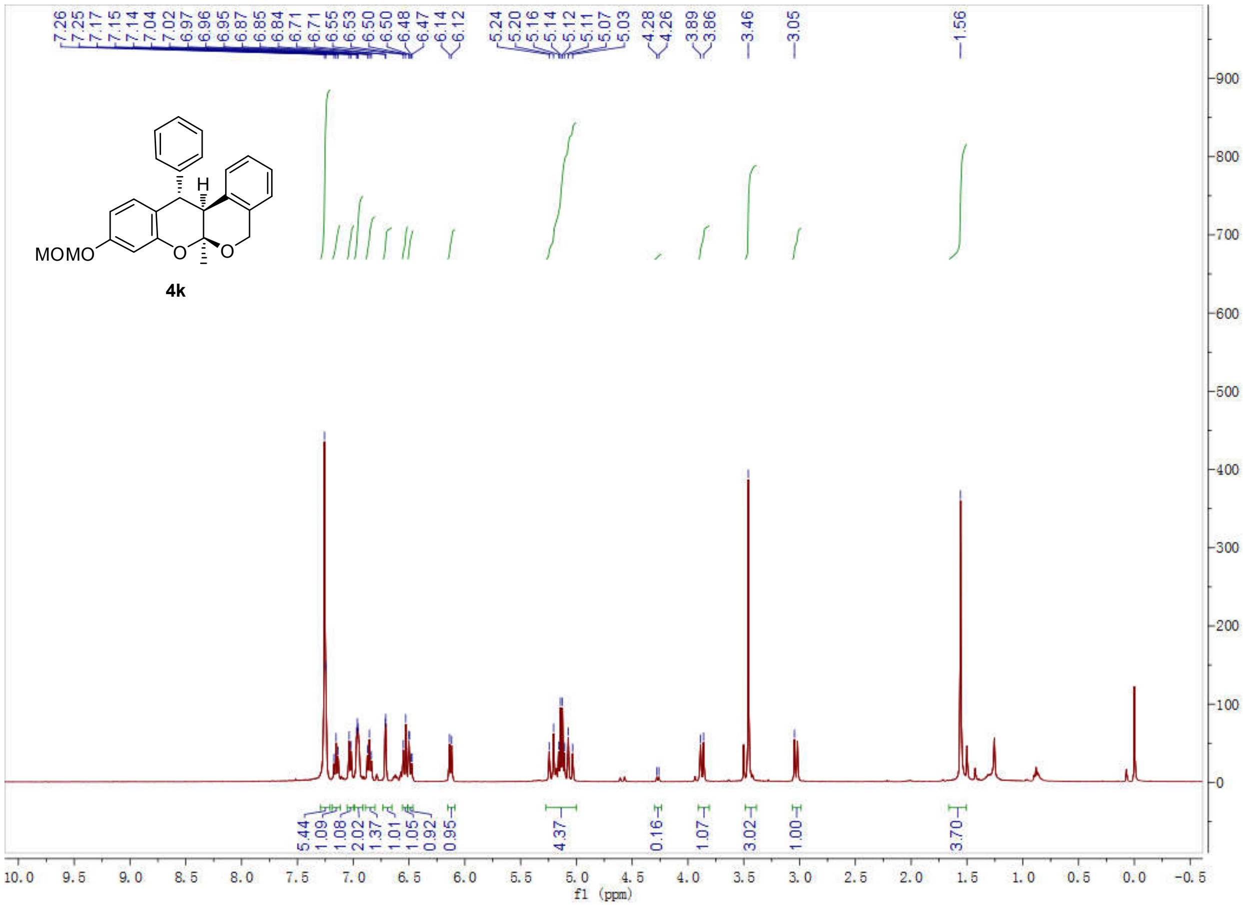
7.26 7.25 7.17 7.15 7.14 7.04 7.02 6.97 6.96 6.95 6.87 6.85 6.84 6.71 6.55 6.53 6.50 6.50 6.48 6.47 6.14 6.12 5.24 5.20 5.16 5.14 5.12 5.11 5.07 5.03 4.28 4.26 3.89 3.86 3.46 3.05 1.56
MOMO
4k
5.44 1.09 1.08 2.02 1.37 1.01 1.05 0.92 0.95 4.37 0.16 1.07 3.02 1.00 3.70
f1 (ppm)

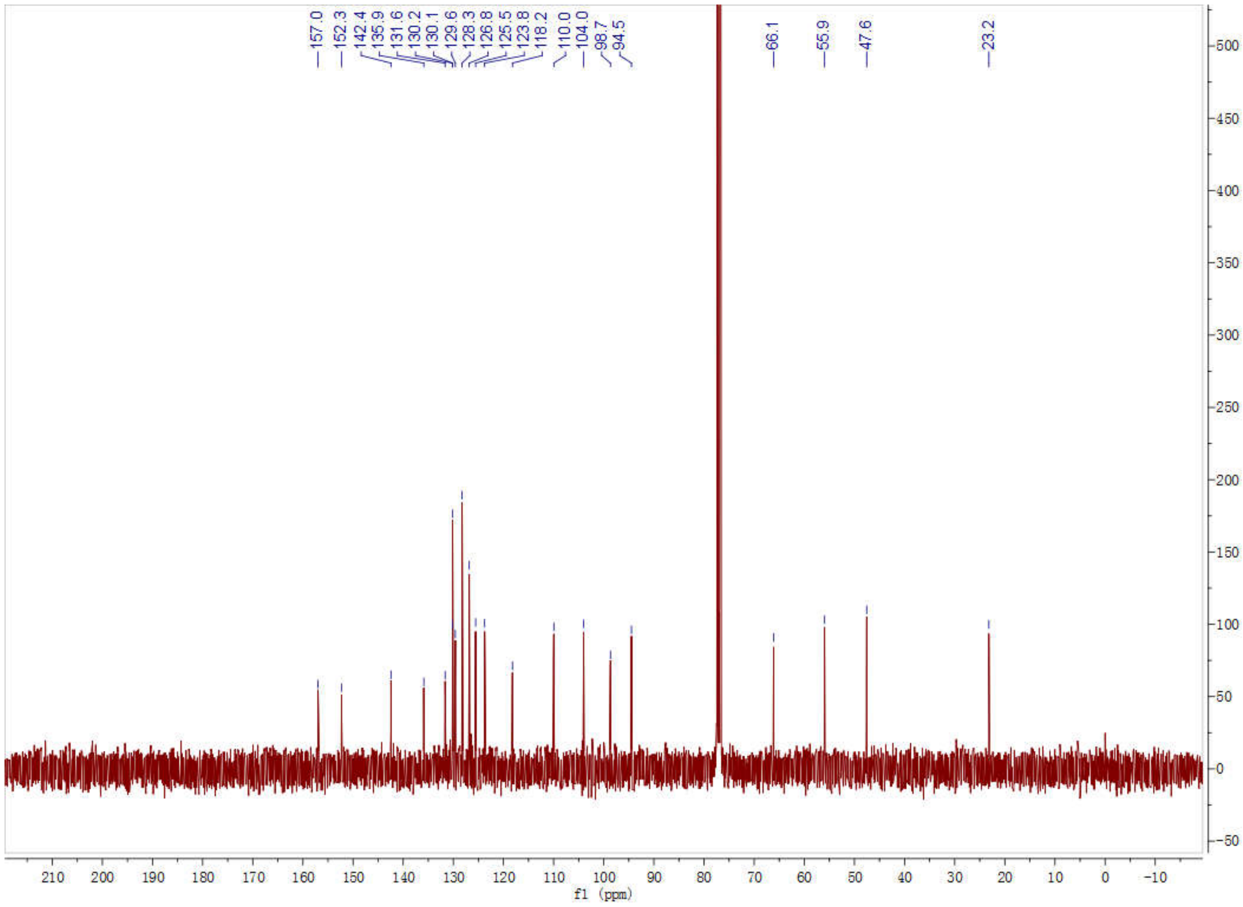
157.0 152.3 142.4 135.9 131.6 130.2 130.1 129.6 128.3 126.8 125.5 123.8 118.2 110.0 104.0 98.7 94.5 66.1 55.9 47.6 23.2
f1 (ppm)

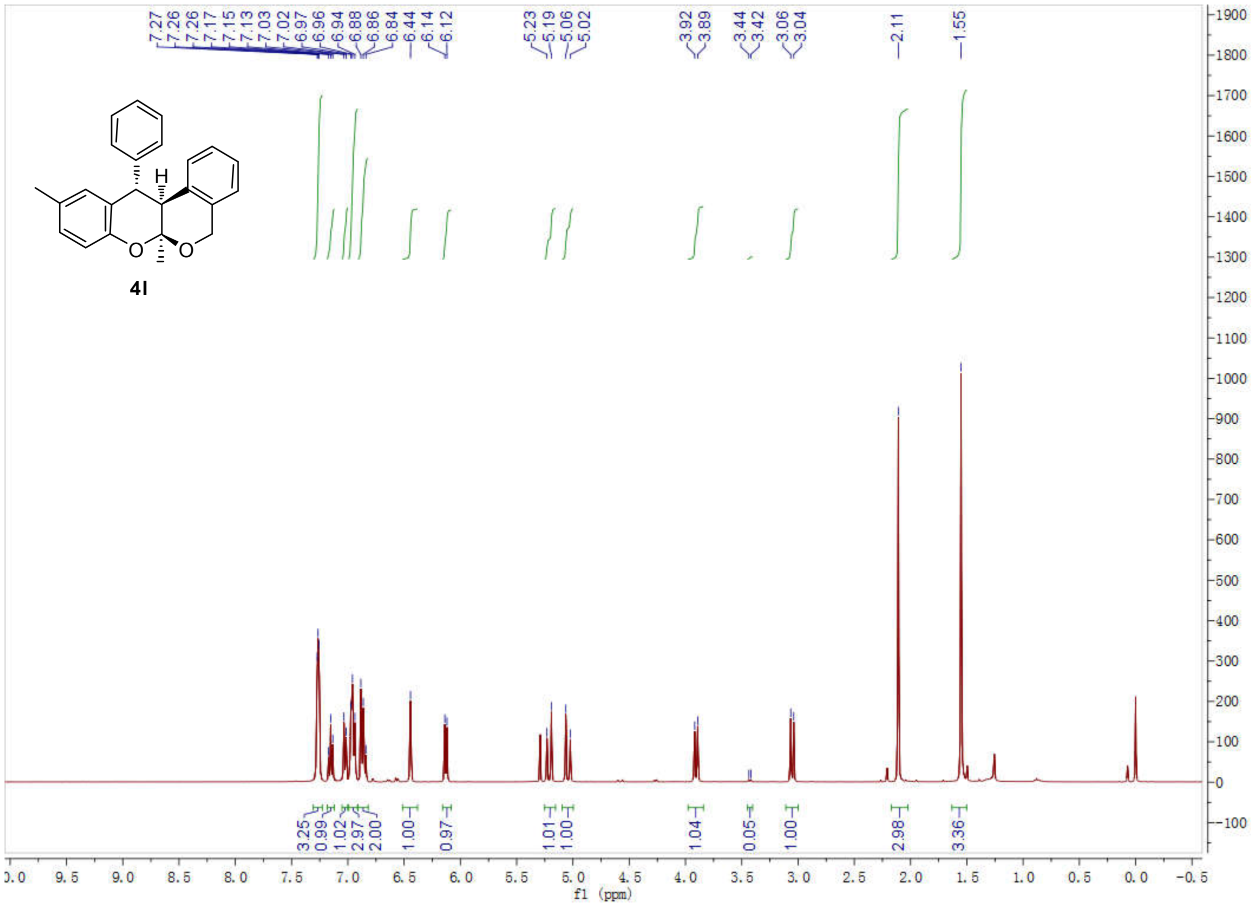
7.27
7.26
7.26
7.17
7.15
7.13
7.03
7.02
6.97
6.96
6.94
6.88
6.86
6.84
6.44
6.14
6.12
5.23
5.19
5.06
5.02
3.92
3.89
3.44
3.42
3.06
3.04
2.11
1.55
H
O
O
4l
3.25
0.99
1.02
2.97
2.00
1.00
0.97
1.01
1.00
1.04
0.05
1.00
2.98
3.36
f1 (ppm)

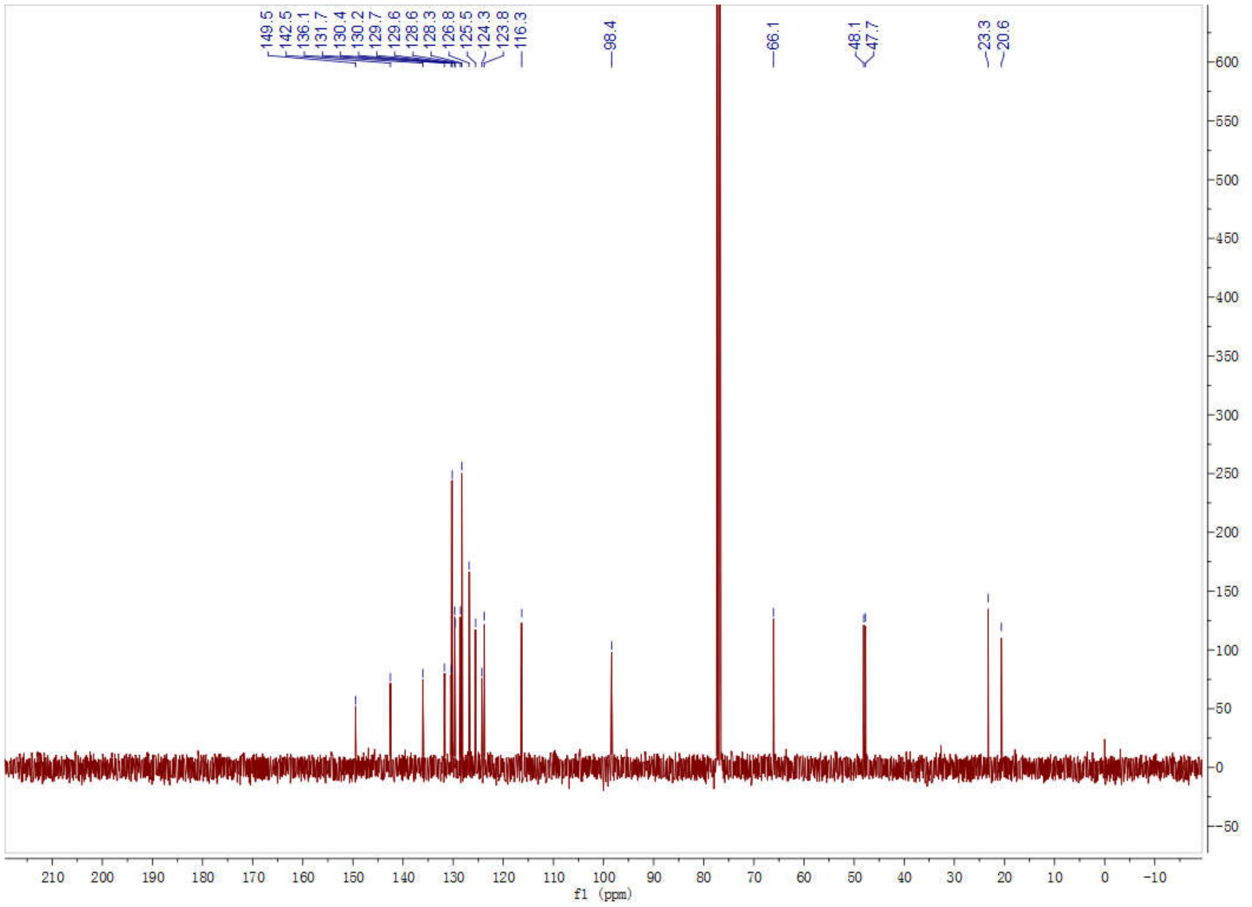
149.5
142.5
136.1
131.7
130.4
130.2
129.7
129.6
128.6
128.3
126.8
125.5
124.3
123.8
116.3
98.4
66.1
48.1
47.7
23.3
20.6
f1 (ppm)

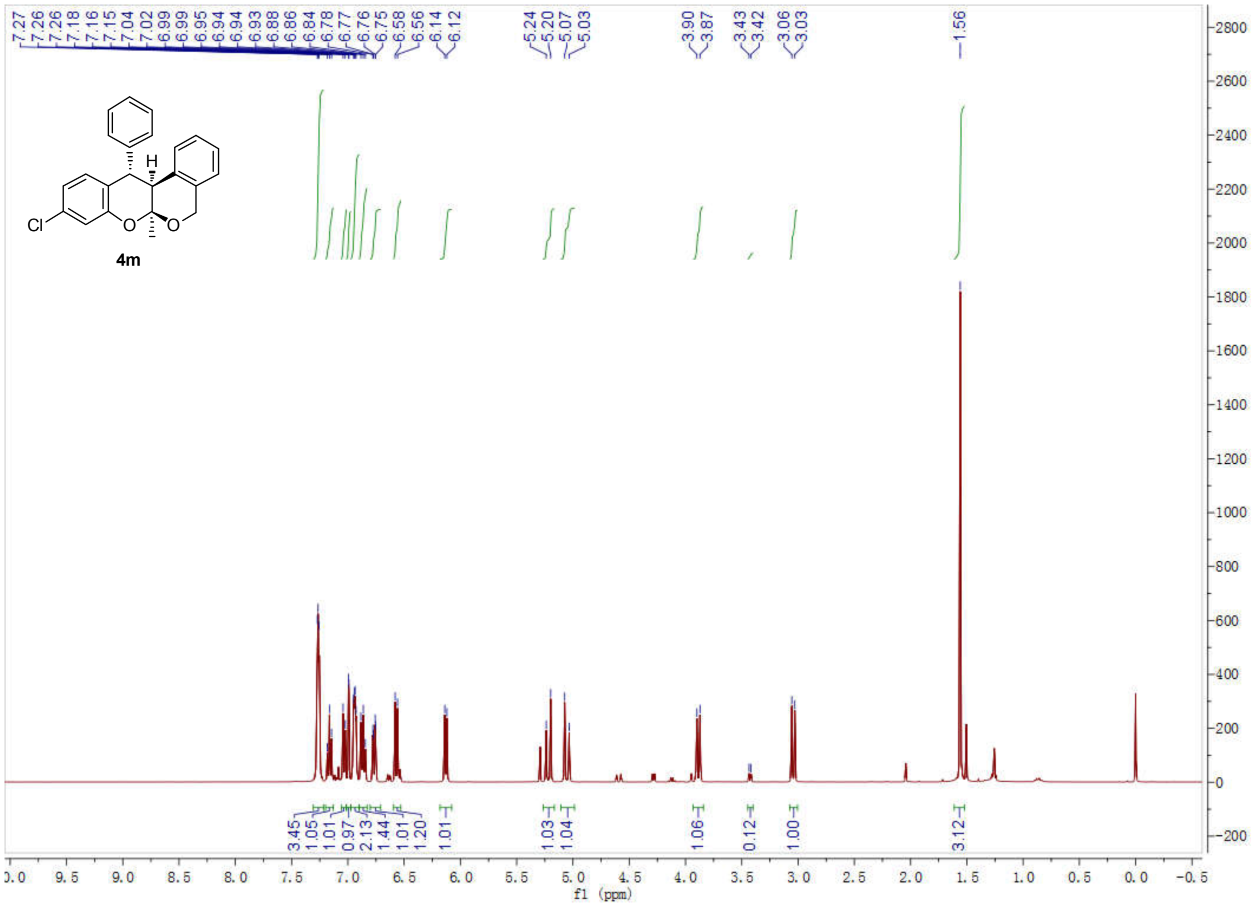
7.27
7.26
7.26
7.18
7.16
7.15
7.04
7.02
6.99
6.99
6.95
6.94
6.94
6.93
6.88
6.86
6.84
6.78
6.77
6.76
6.75
6.58
6.56
6.14
6.12
5.24
5.20
5.07
5.03
3.90
3.87
3.43
3.42
3.06
3.03
1.56
H
Cl
O
O
4m
3.45
1.05
1.01
0.97
2.13
1.44
1.01
1.20
1.01
1.03
1.04
1.06
0.12
1.00
3.12
f1 (ppm)

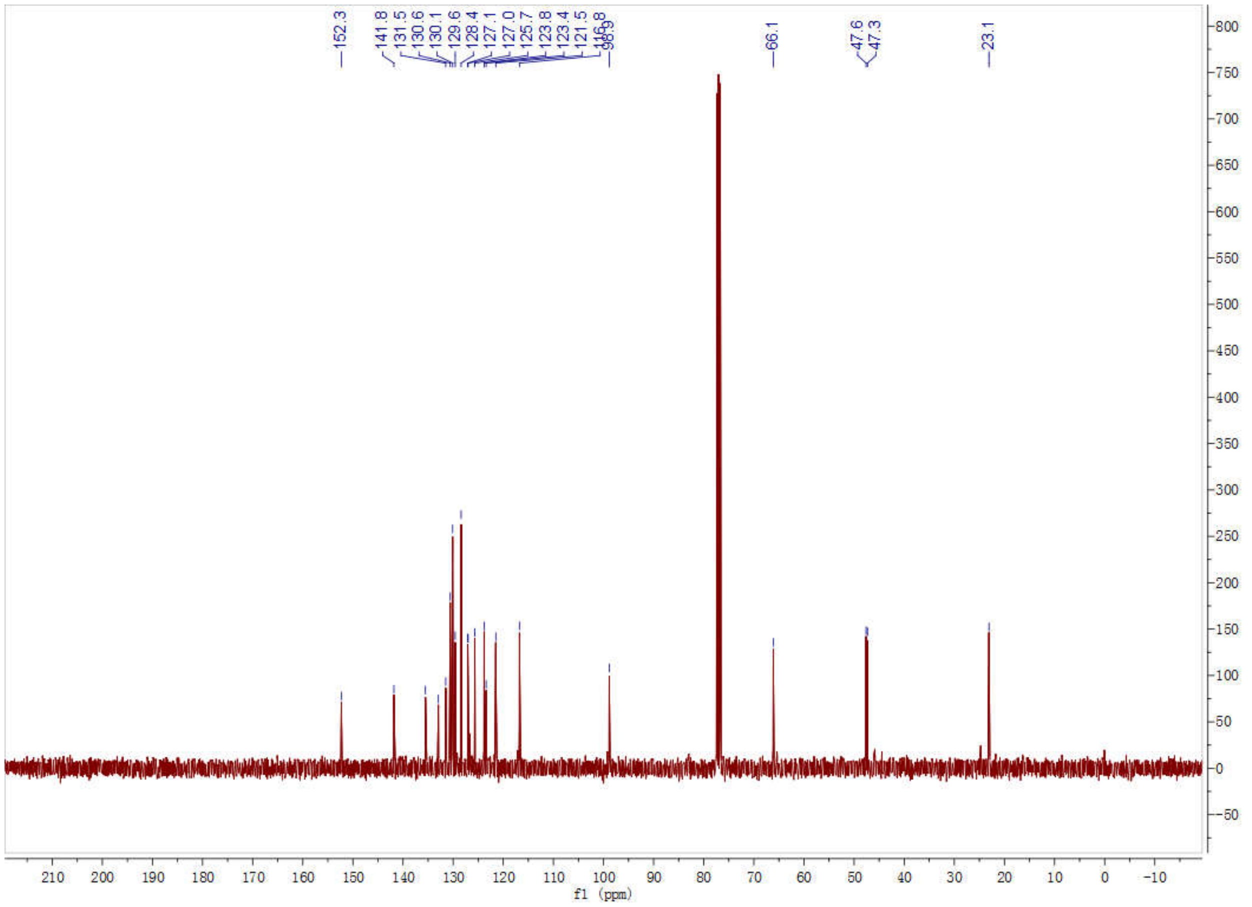
152.3
141.8
131.5
130.6
130.1
129.6
128.4
127.1
127.0
125.7
123.8
123.4
121.5
116.8
98.9
66.1
47.6
47.3
23.1
f1 (ppm)

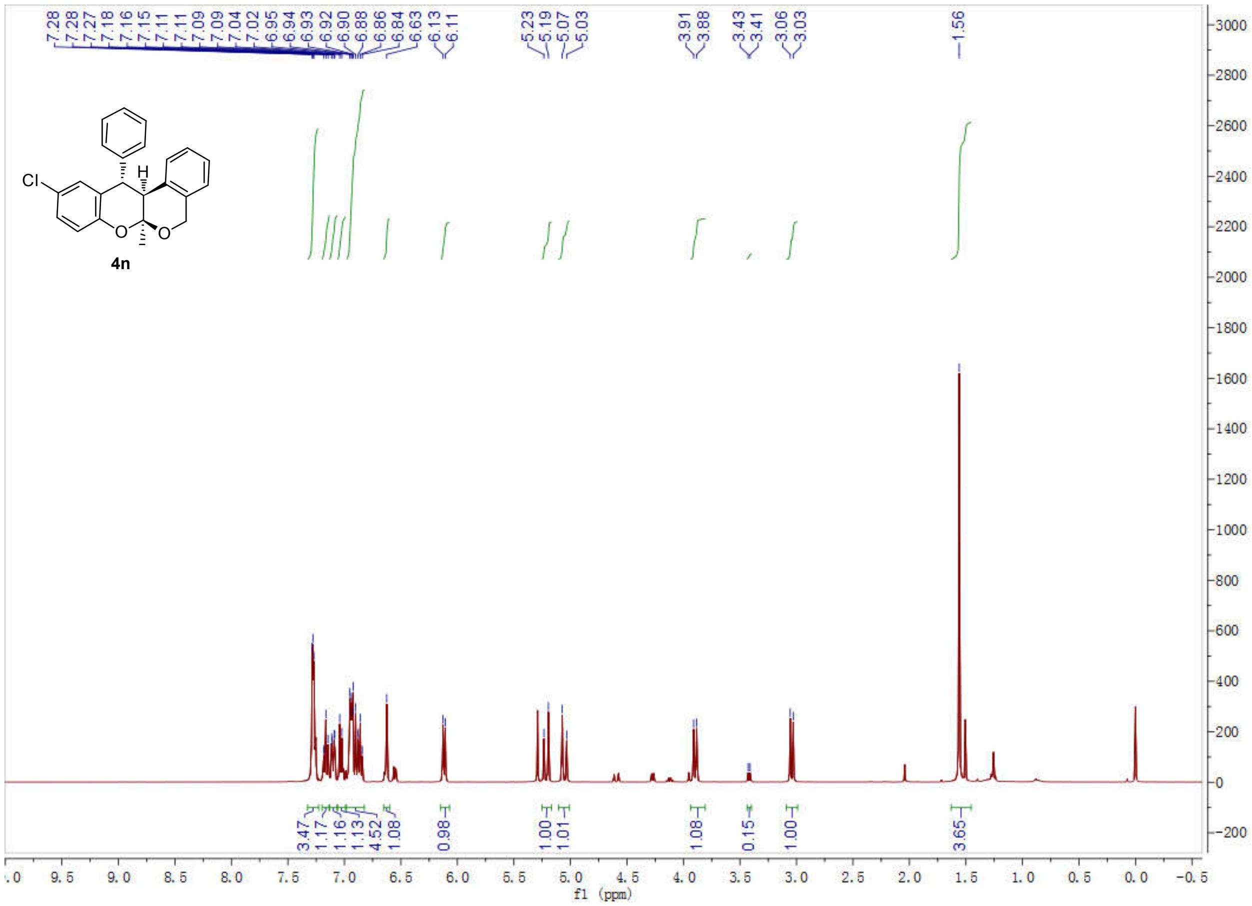
7.28
7.28
7.27
7.18
7.16
7.15
7.11
7.11
7.09
7.09
7.04
7.02
6.95
6.94
6.93
6.92
6.90
6.88
6.86
6.84
6.63
6.13
6.11
5.23
5.19
5.07
5.03
3.91
3.88
3.43
3.41
3.06
3.03
1.56
Cl
H
O
O
4n
3.47
1.17
1.16
1.13
4.52
1.08
0.98
1.00
1.01
1.08
0.15
1.00
3.65
f1 (ppm)

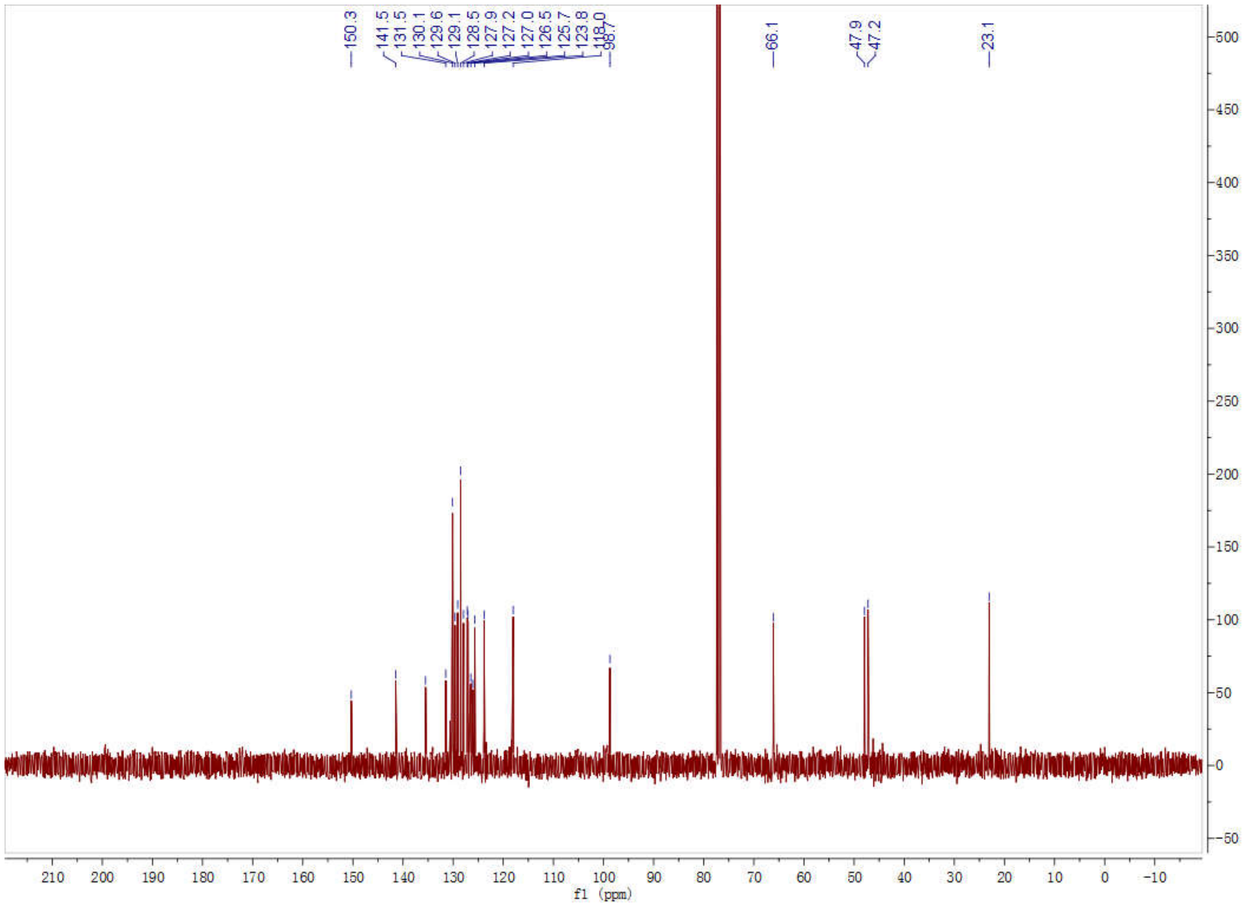
150.3
141.5
131.5
130.1
129.6
129.1
128.5
127.9
127.2
127.0
126.5
125.7
123.8
118.0
98.7
66.1
47.9
47.2
23.1
f1 (ppm)

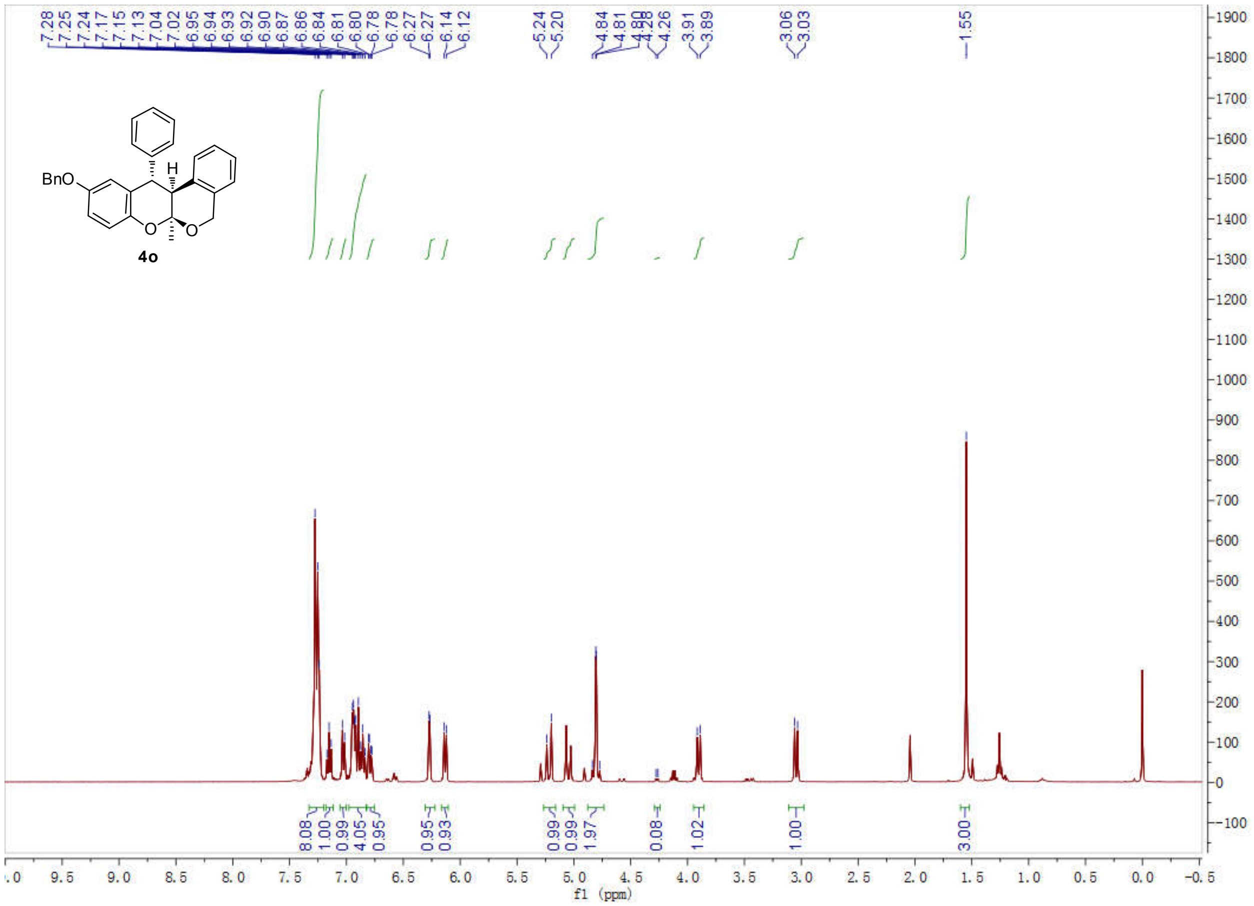
7.28
7.25
7.24
7.17
7.15
7.13
7.04
7.02
6.95
6.94
6.93
6.92
6.90
6.87
6.86
6.84
6.81
6.80
6.78
6.27
6.27
6.14
6.12
5.24
5.20
4.84
4.81
4.28
4.26
3.91
3.89
3.06
3.03
1.55
BnO
H
O
O
4o
8.08
1.00
0.99
4.05
0.95
0.95
0.93
0.99
0.99
1.97
0.08
1.02
1.00
3.00
f1 (ppm)

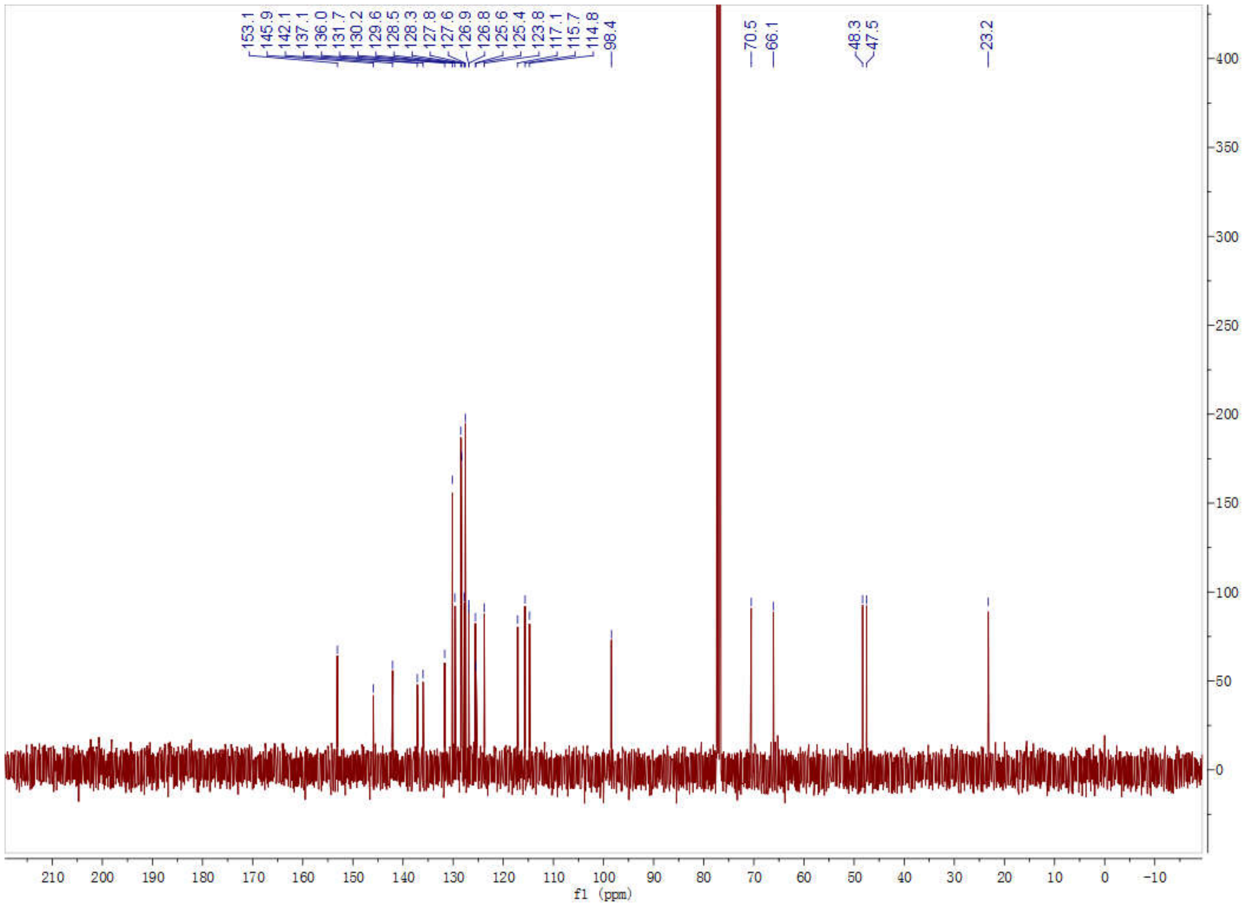
153.1
145.9
142.1
137.1
136.0
131.7
130.2
129.6
128.5
128.3
127.8
127.6
126.9
126.8
125.6
125.4
123.8
117.1
115.7
114.8
98.4
70.5
66.1
48.3
47.5
23.2
f1 (ppm)

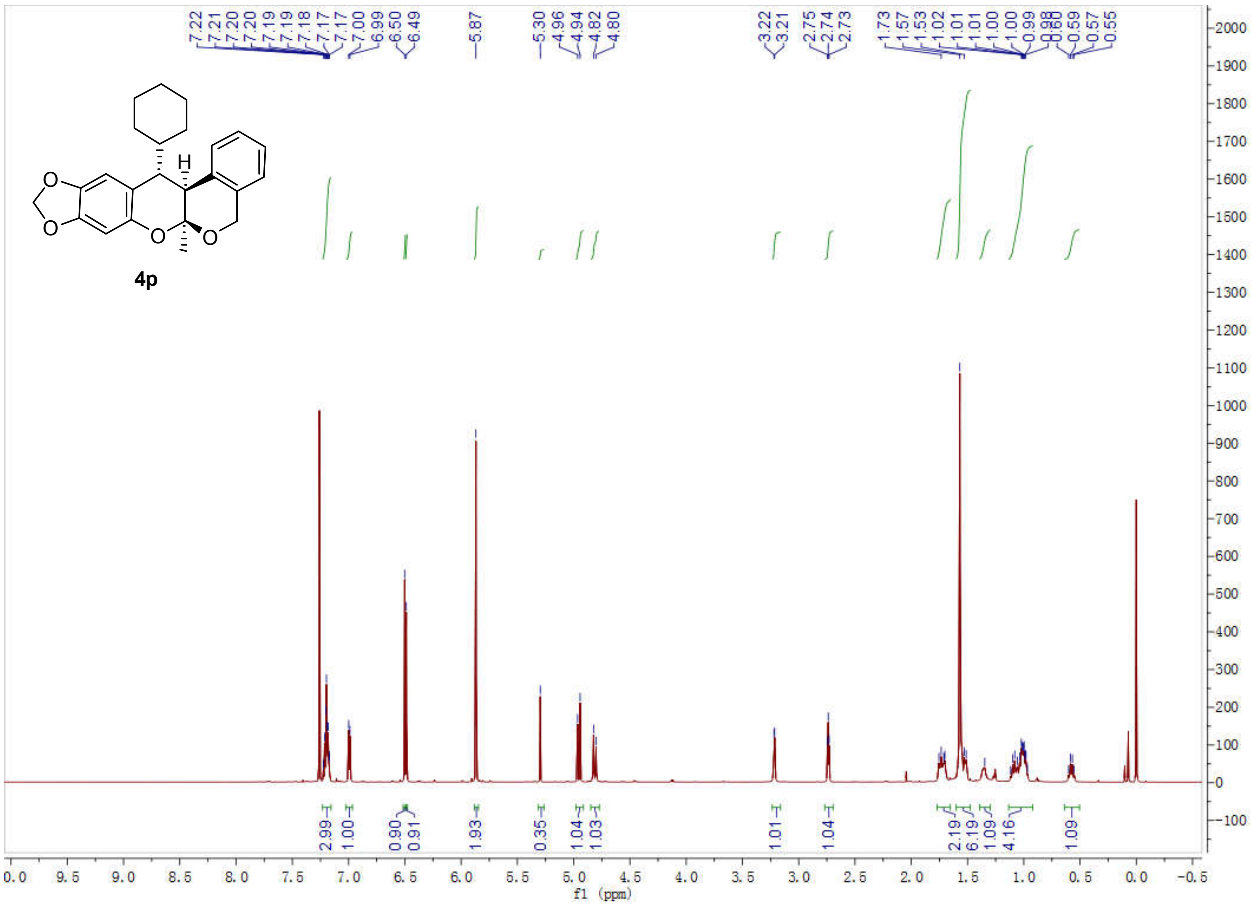
4p
f1 (ppm)

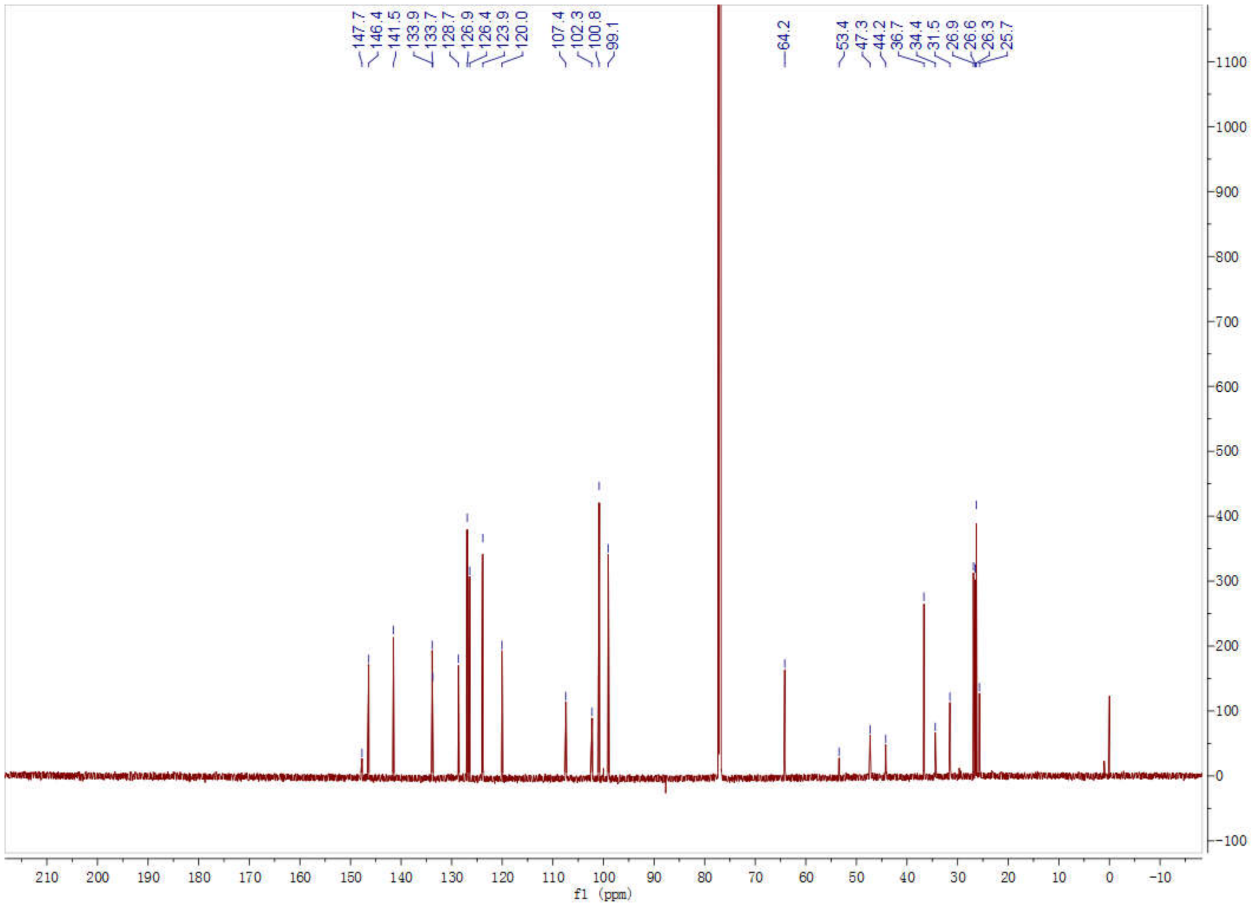
f1 (ppm)

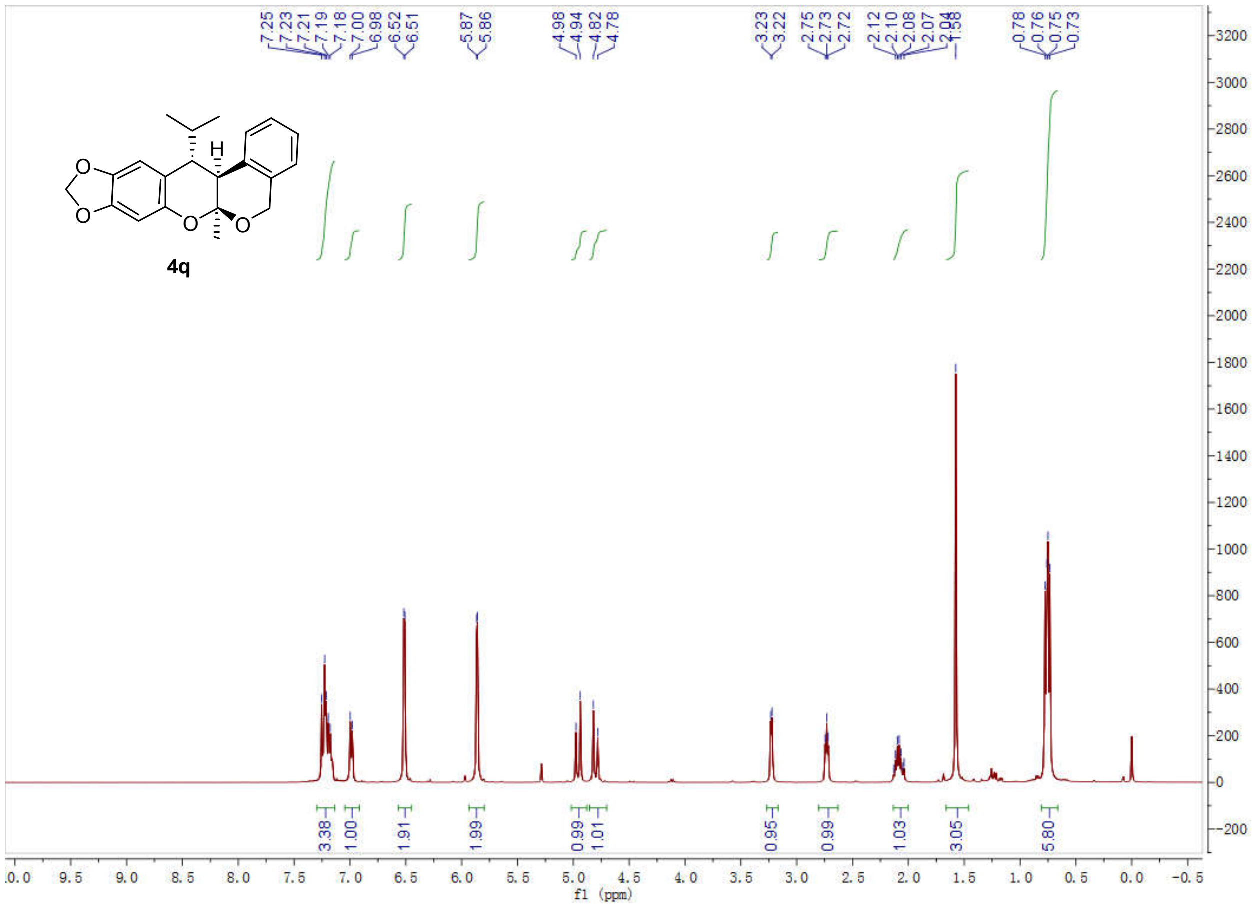
4q
7.25 7.23 7.21 7.19 7.18 7.00 6.98 6.52 6.51 5.87 5.86 4.98 4.94 4.82 4.78 3.23 3.22 2.75 2.73 2.72 2.12 2.10 2.08 2.07 2.04 1.58 0.78 0.76 0.75 0.73
3.38 1.00 1.91 1.99 0.99 1.01 0.95 0.99 1.03 3.05 5.80
f1 (ppm)

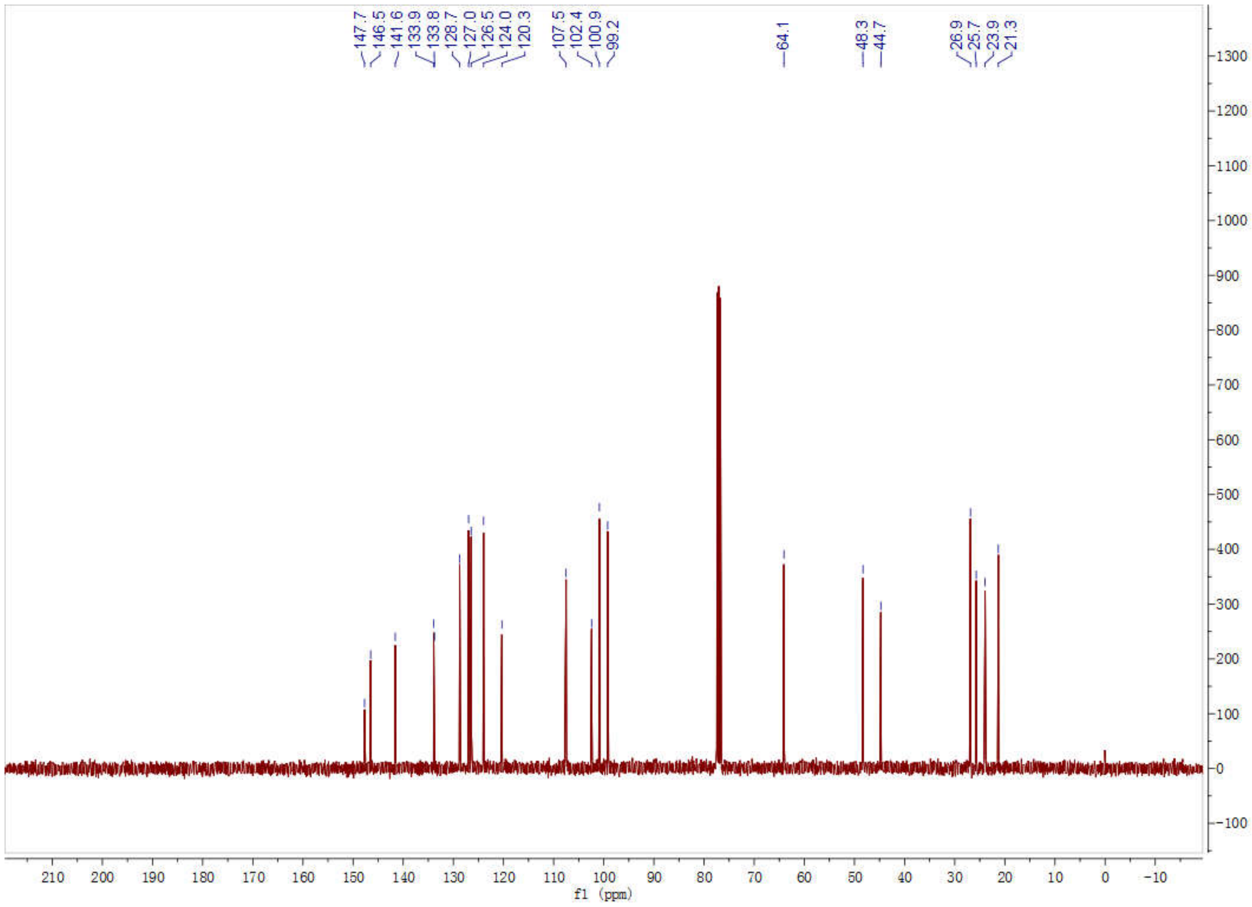
147.7 146.5 141.6 133.9 133.8 128.7 127.0 126.5 124.0 120.3 107.5 102.4 100.9 99.2 64.1 48.3 44.7 26.9 25.7 23.9 21.3
f1 (ppm)

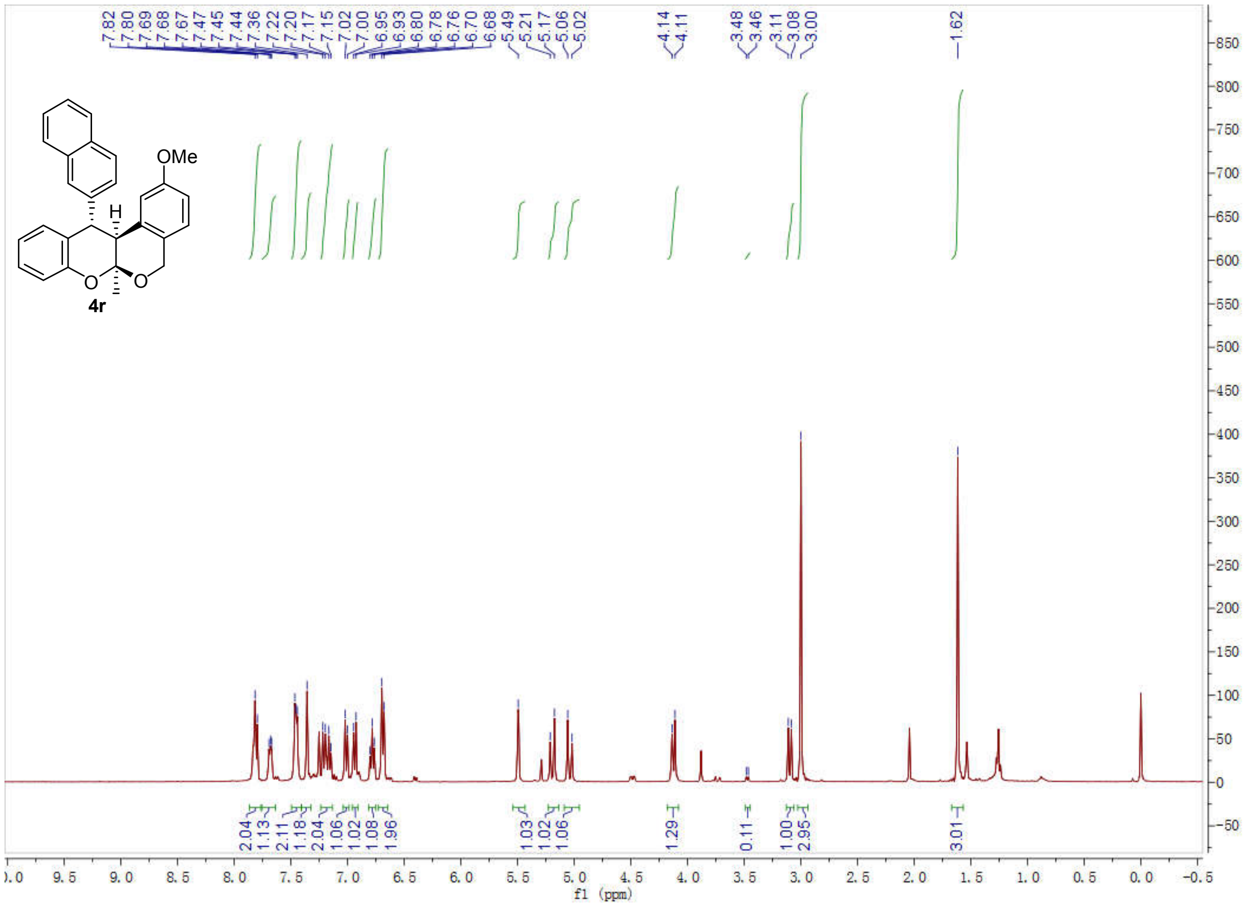

7.82
7.80
7.69
7.68
7.67
7.47
7.45
7.44
7.36
7.22
7.20
7.17
7.15
7.02
7.00
6.95
6.93
6.80
6.78
6.76
6.70
6.68
5.49
5.21
5.17
5.06
5.02
4.14
4.11
3.48
3.46
3.11
3.08
3.00
1.62
OMe
H
O
O
4r
2.04
1.13
2.11
1.18
2.04
1.06
1.02
1.08
1.96
1.03
1.02
1.06
1.29
0.11
1.00
2.95
3.01
f1 (ppm)

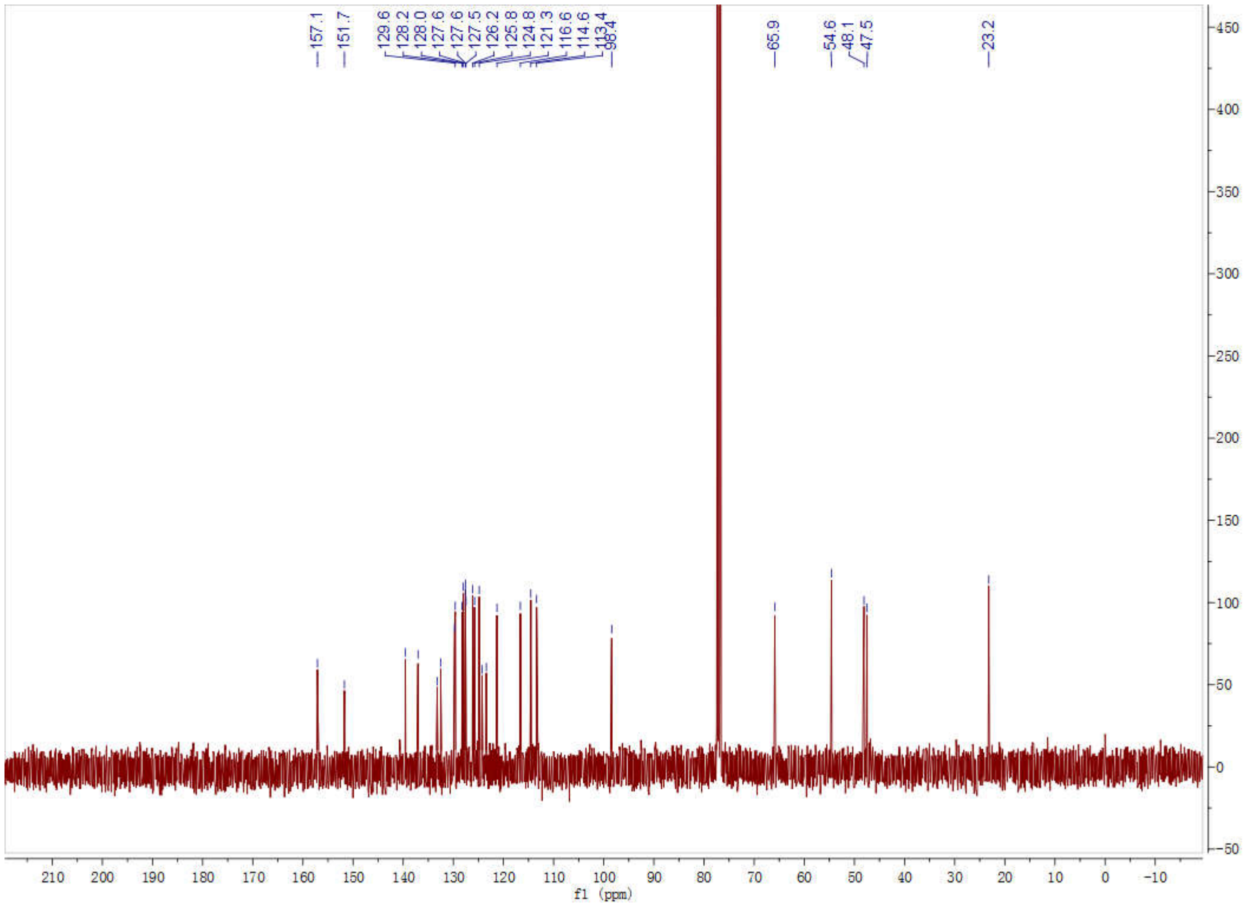

157.1
151.7
129.6
128.2
128.0
127.6
127.6
127.5
126.2
125.8
124.8
121.3
116.6
114.6
113.4
98.4
65.9
54.6
48.1
47.5
23.2
f1 (ppm)

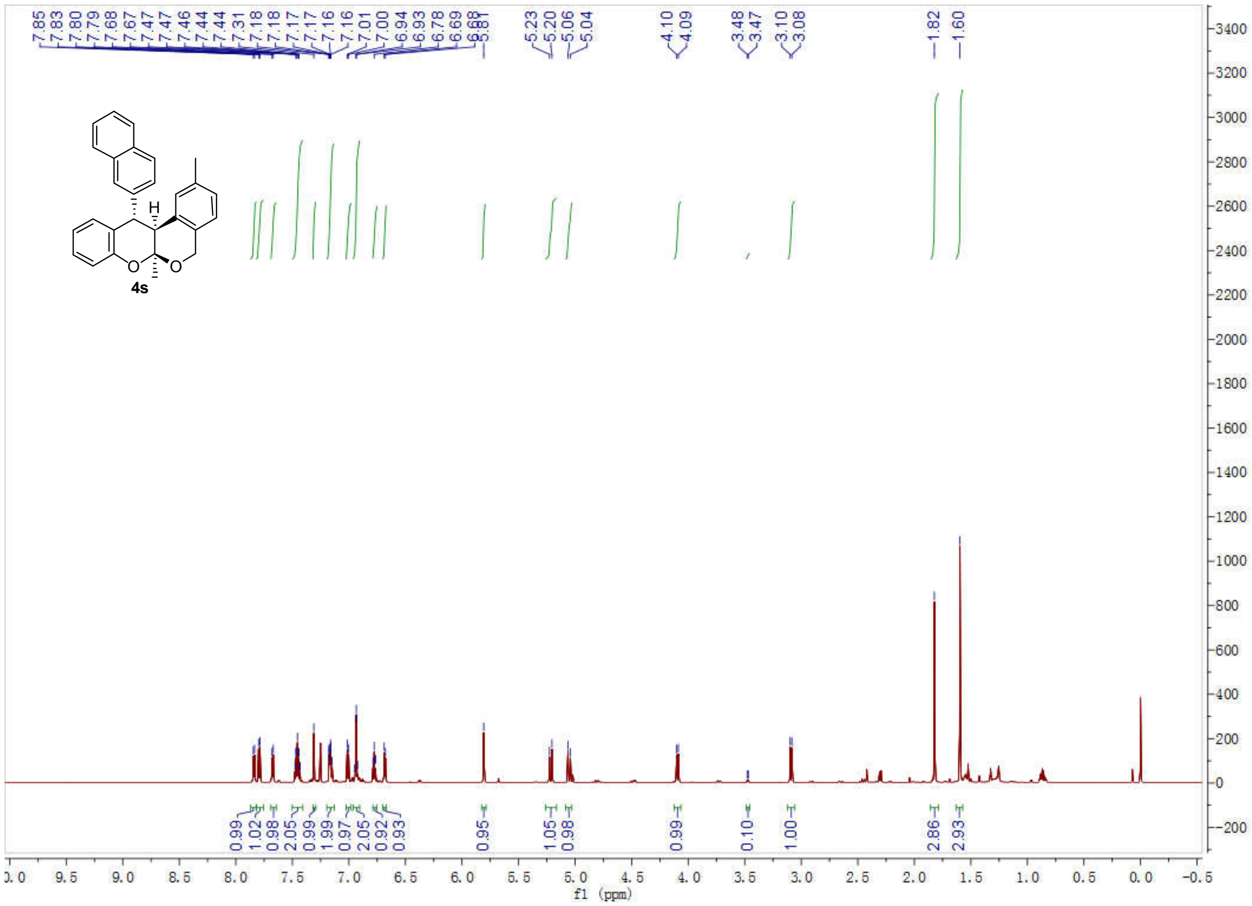
7.85 7.83 7.80 7.79 7.68 7.67 7.47 7.47 7.46 7.44 7.44 7.31 7.18 7.18 7.17 7.16 7.16 7.01 7.00 6.94 6.93 6.78 6.69 6.68 5.81
5.23 5.20 5.06 5.04
4.10 4.09
3.48 3.47 3.10 3.08
1.82 1.60
4s
0.99 1.02 0.98 2.05 0.99 1.99 0.97 2.05 0.92 0.93 0.95 1.05 0.98 0.99 0.10 1.00 2.86 2.93
f1 (ppm)

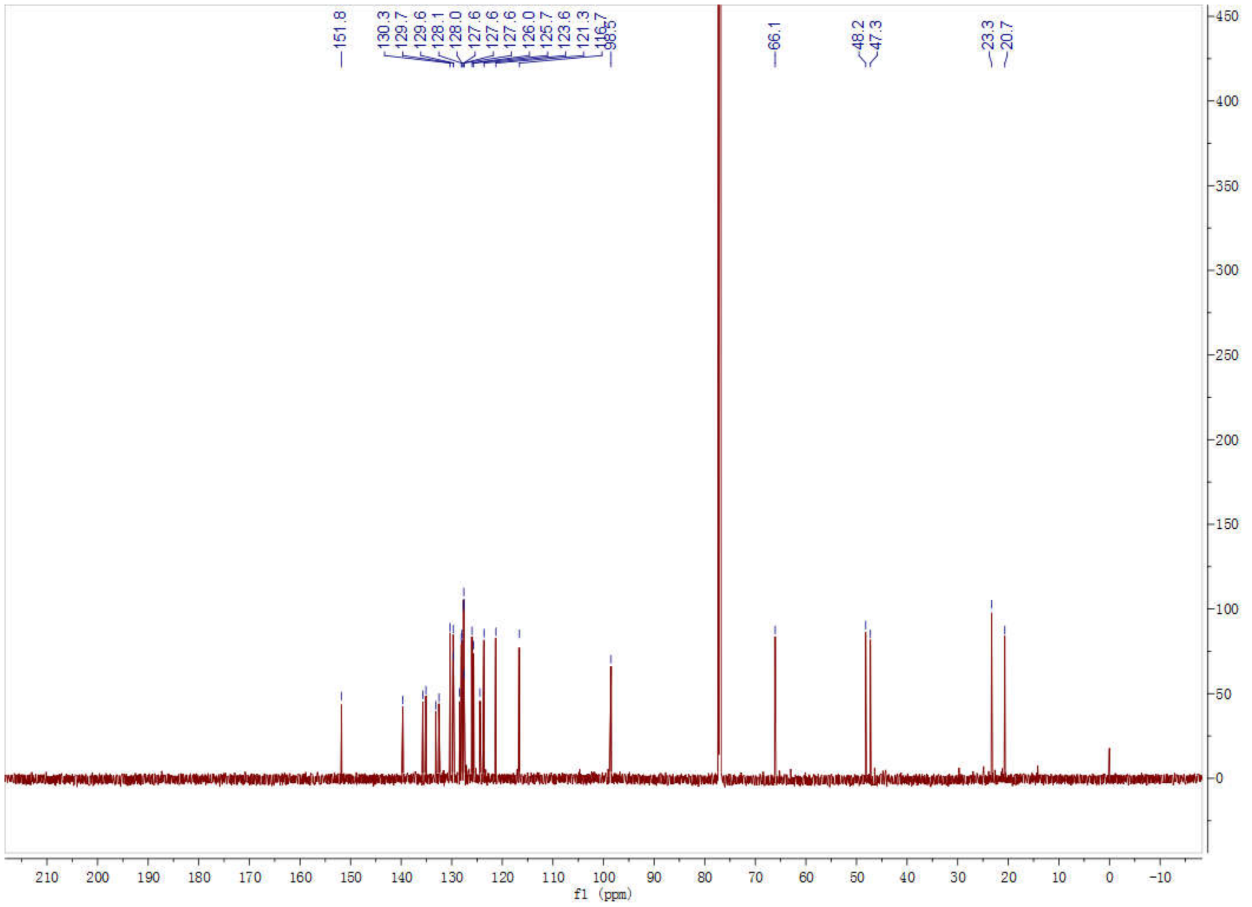
151.8
130.3 129.7 129.6 128.1 128.0 127.6 127.6 127.6 126.0 125.7 123.6 121.3 116.7 98.5
66.1
48.2 47.3
23.3 20.7
f1 (ppm)

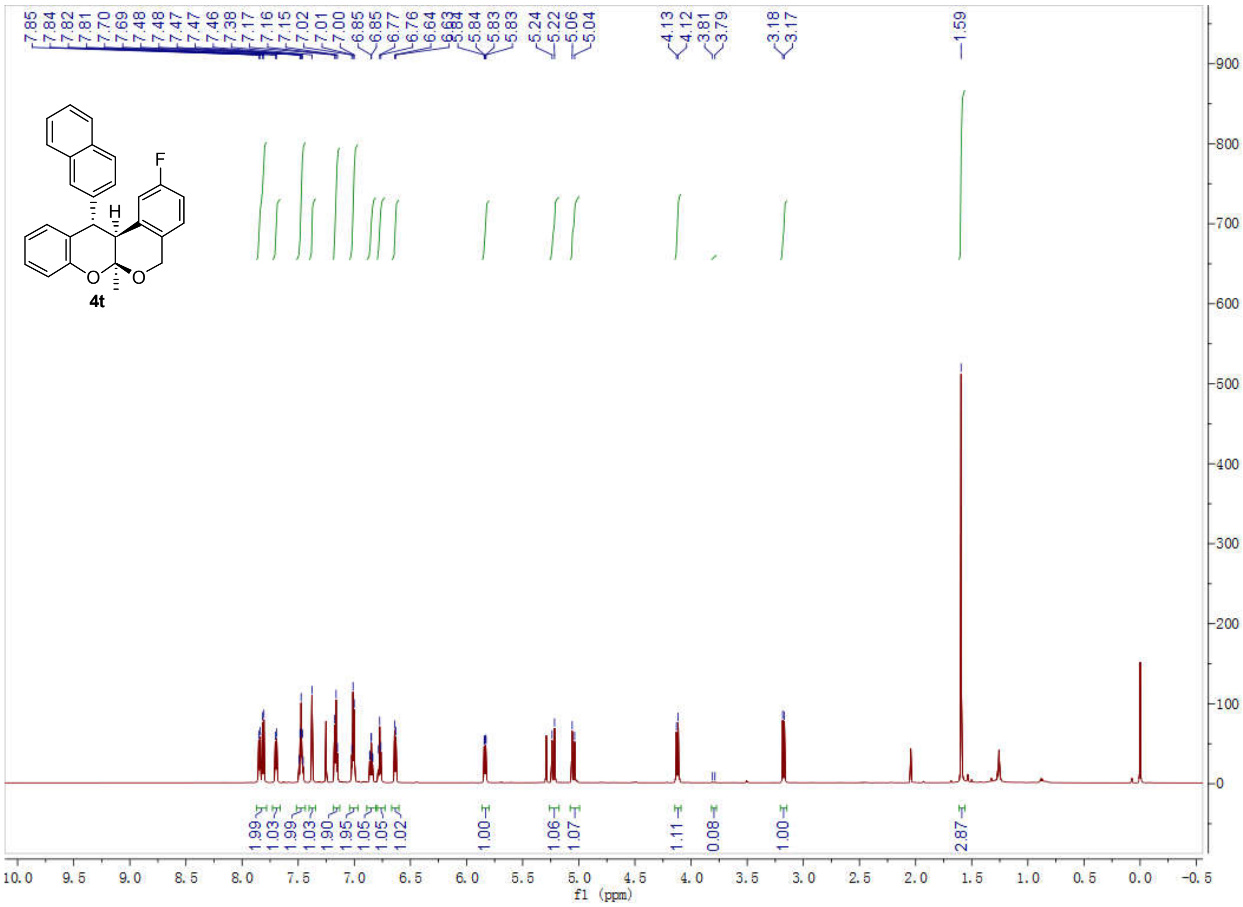
7.85
7.84
7.82
7.81
7.70
7.69
7.48
7.48
7.47
7.47
7.46
7.38
7.17
7.16
7.15
7.02
7.01
7.00
6.85
6.85
6.77
6.76
6.64
6.63
5.84
5.84
5.83
5.83
5.24
5.22
5.06
5.04
4.13
4.12
3.81
3.79
3.18
3.17
1.59
F
H
O
O
4t
1.99
1.03
1.99
1.03
1.90
1.95
1.05
1.05
1.02
1.00
1.06
1.07
1.11
0.08
1.00
2.87
f1 (ppm)

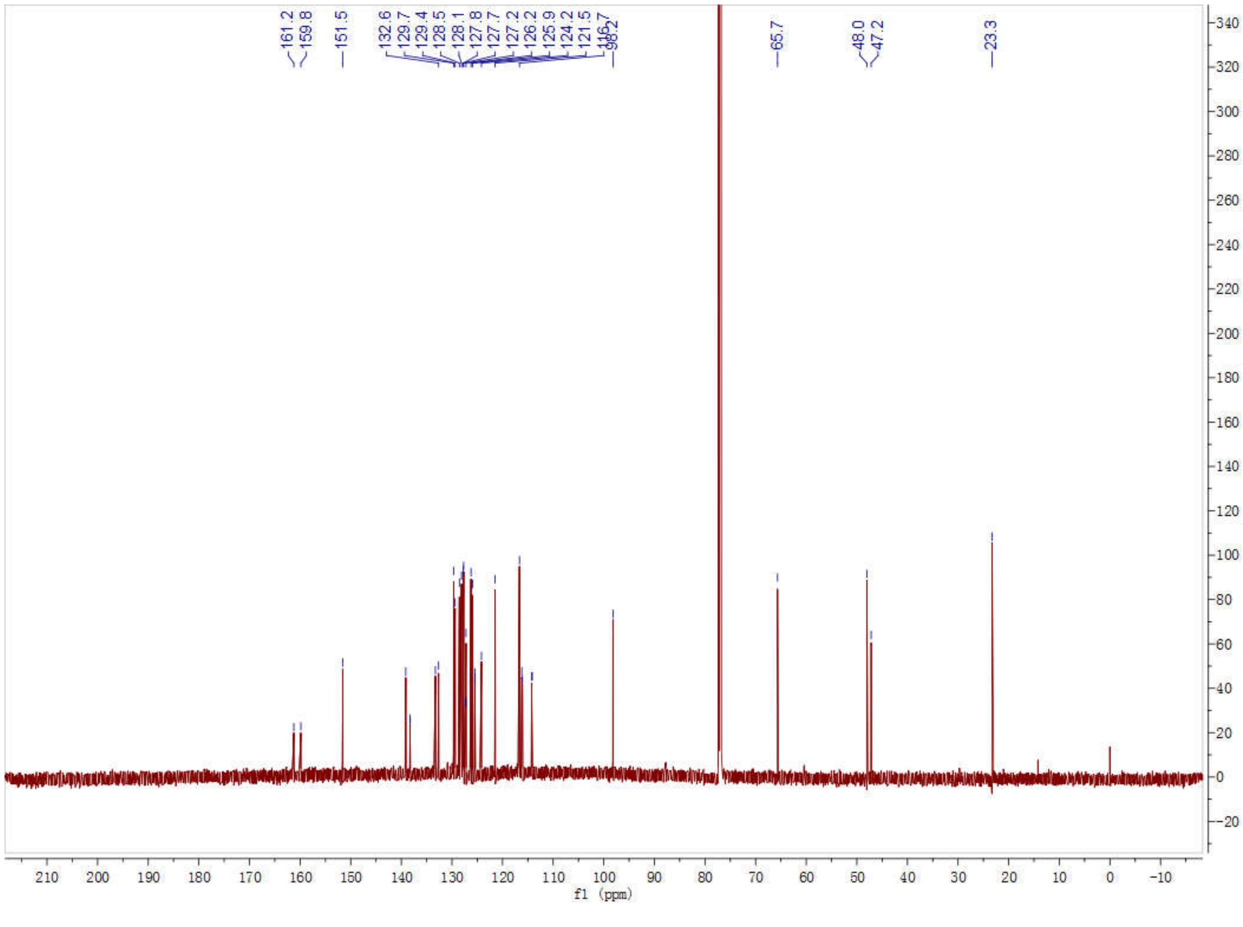
161.2
159.8
151.5
132.6
129.7
129.4
128.5
128.1
127.8
127.7
127.2
126.2
125.9
124.2
121.5
116.7
98.2
65.7
48.0
47.2
23.3
f1 (ppm)

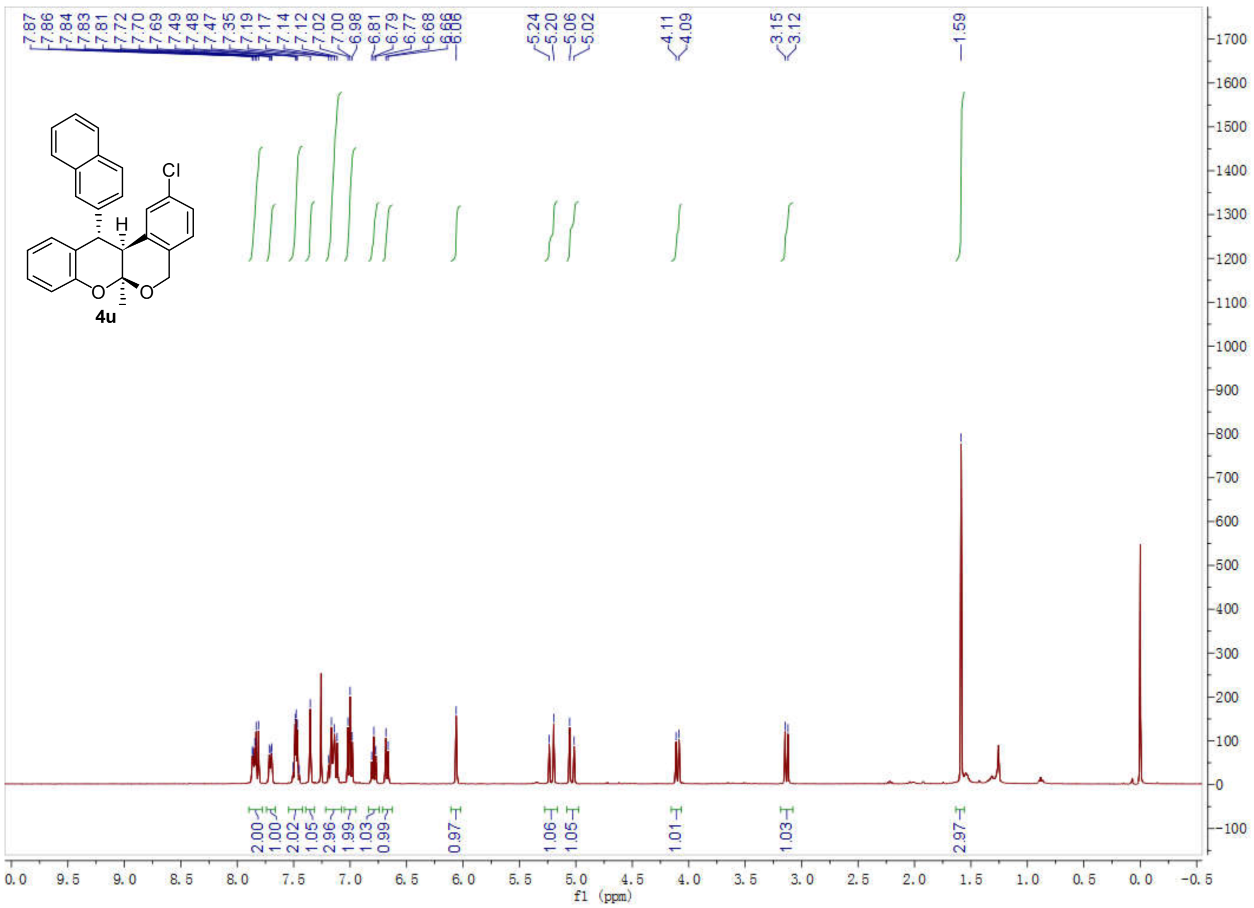
7.87 7.86 7.84 7.83 7.81 7.72 7.70 7.69 7.49 7.48 7.47 7.35 7.19 7.17 7.14 7.12 7.02 7.00 6.98 6.81 6.79 6.77 6.68 6.66 6.06
5.24 5.20 5.06 5.02
4.11 4.09
3.15 3.12
1.59
Cl
H
O
4u
2.00 1.00 2.02 1.05 2.96 1.99 1.03 0.99 0.97 1.06 1.05 1.01 1.03 2.97
f1 (ppm)

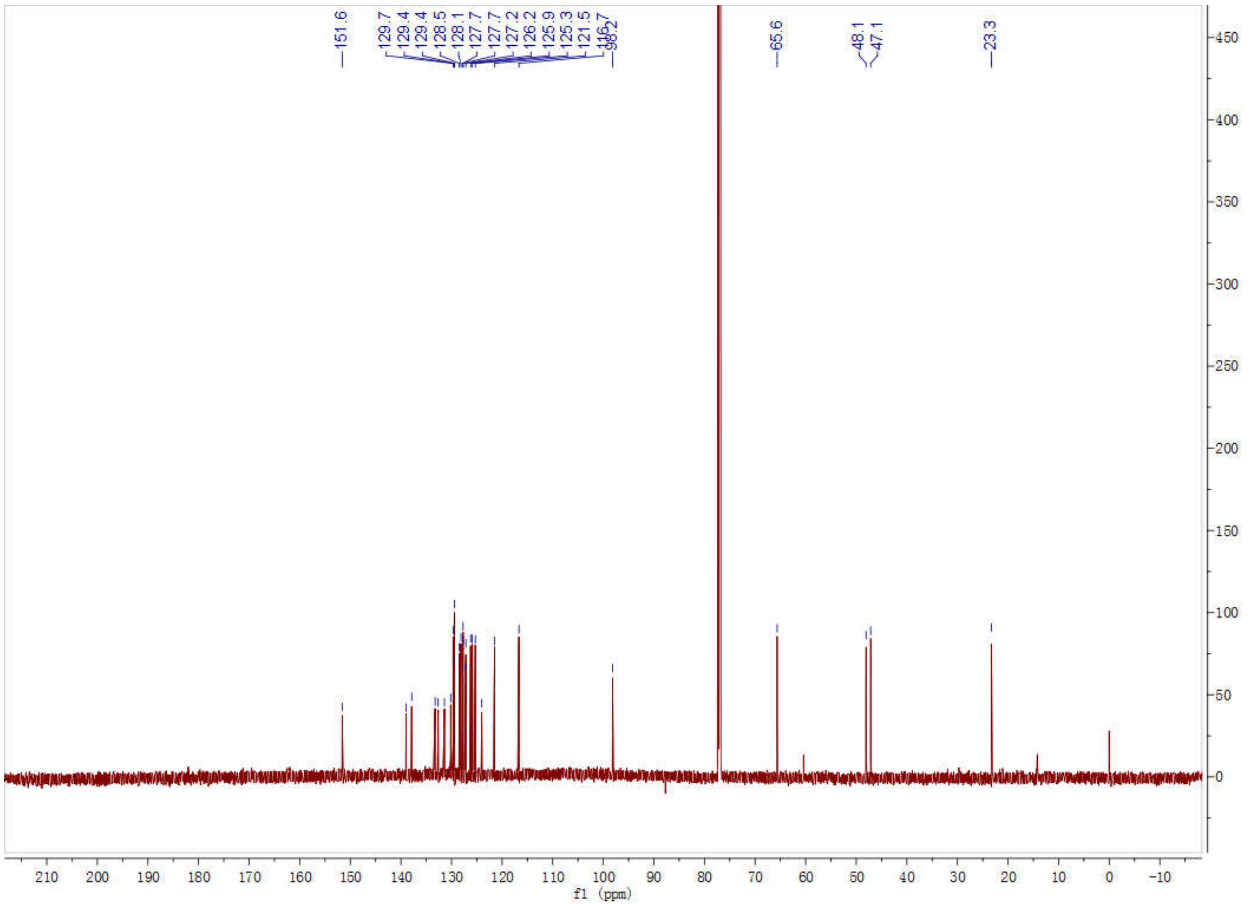
151.6
129.7 129.4 129.4 128.5 128.1 127.7 127.7 127.2 126.2 125.9 125.3 121.5 116.7 98.2
65.6
48.1 47.1
23.3
f1 (ppm)

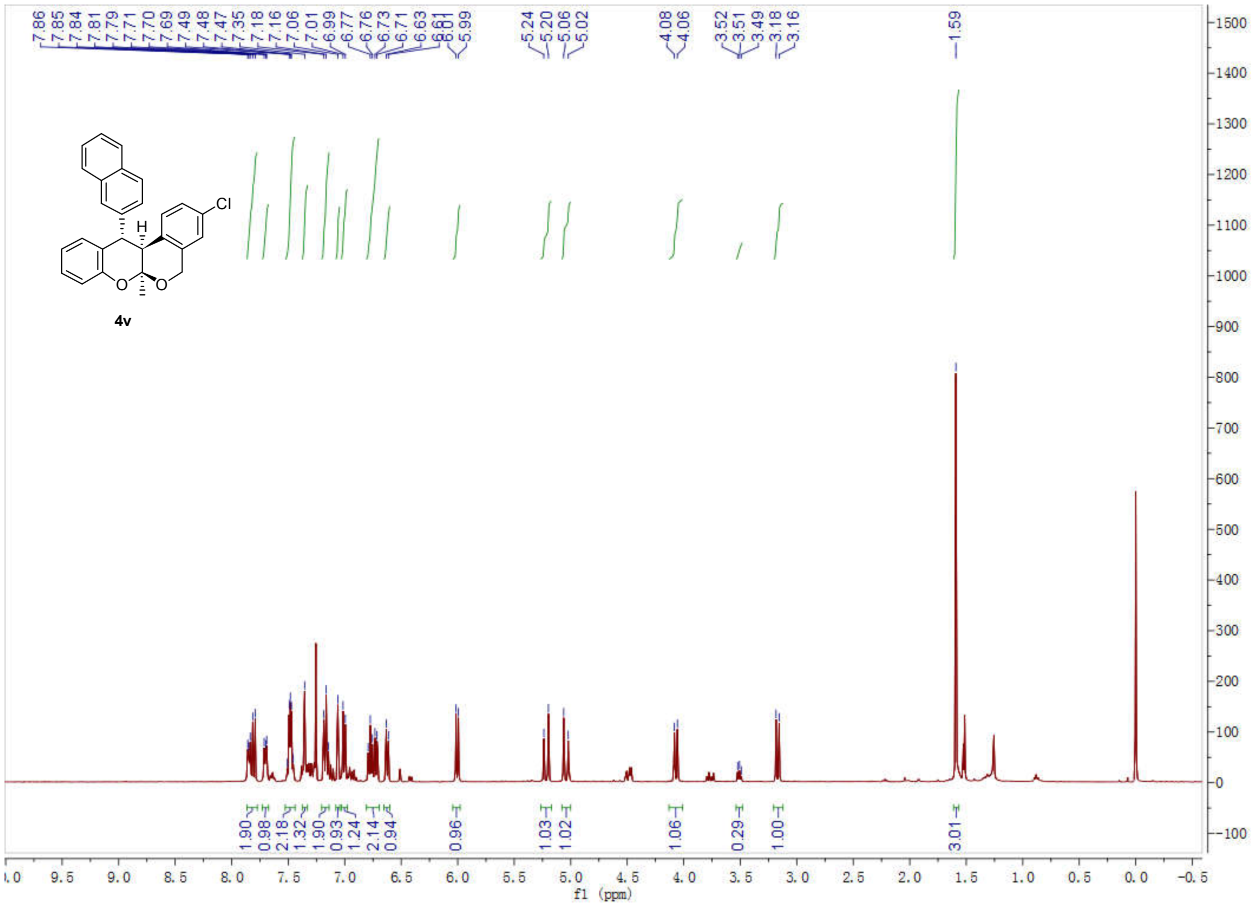

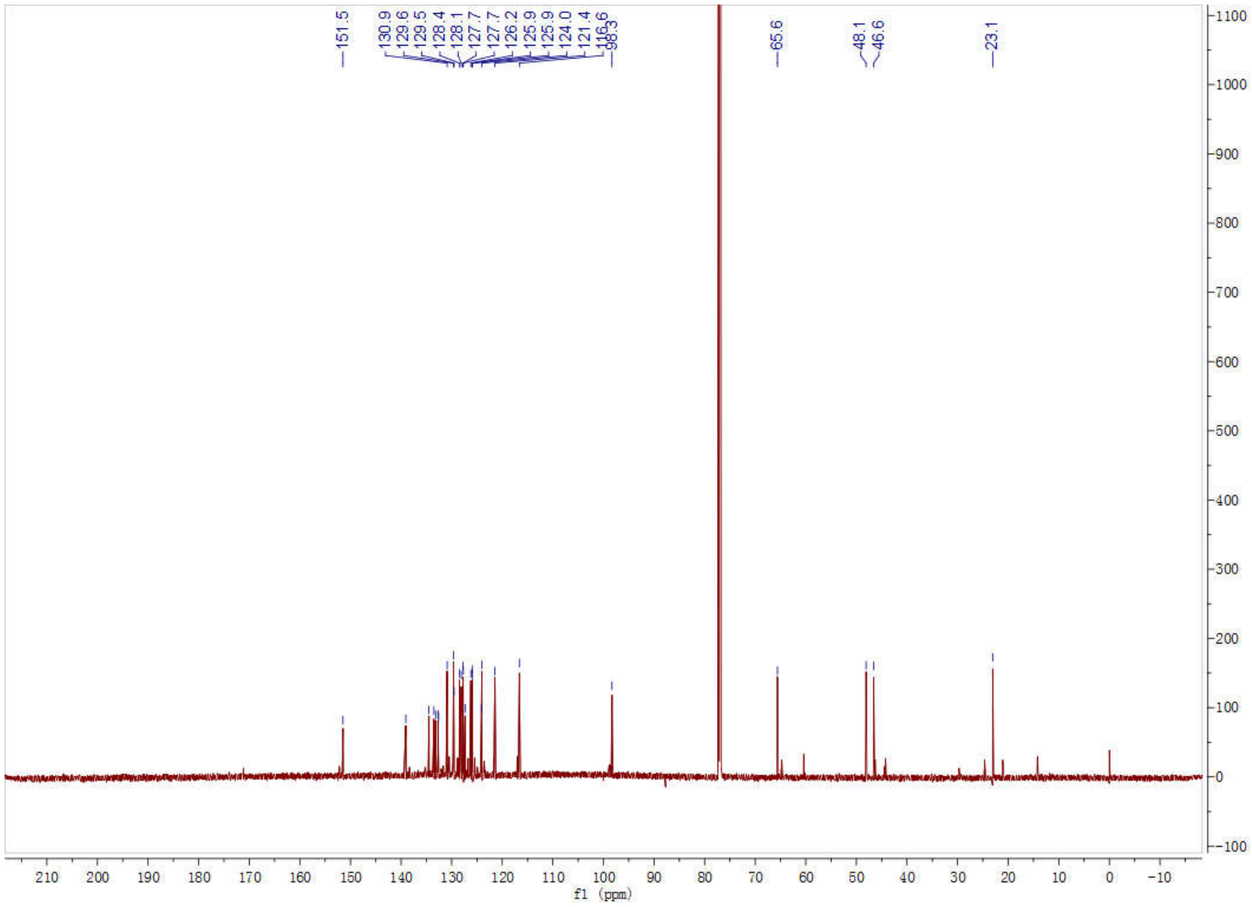

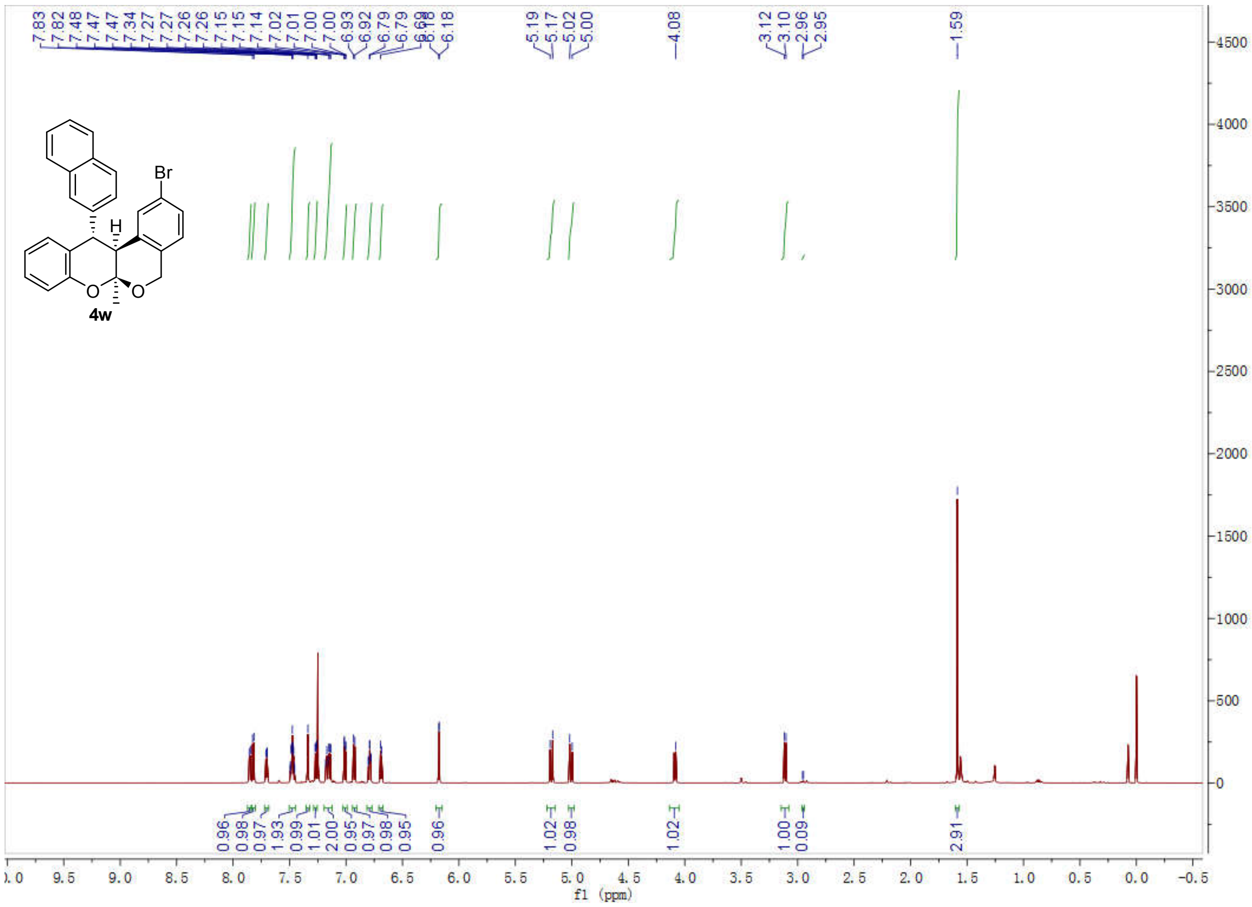
7.83 7.82 7.48 7.47 7.47 7.34 7.27 7.27 7.26 7.26 7.15 7.15 7.14 7.02 7.01 7.00 7.00 6.93 6.92 6.79 6.79 6.69 6.68 6.18
5.19 5.17 5.02 5.00
4.08
3.12 3.10 2.96 2.95
1.59
Br
H
O
O
4w
0.96 0.98 0.97 1.93 0.99 1.01 2.00 0.95 0.97 0.98 0.95 0.96 1.02 0.98 1.02 1.00 0.09 2.91
f1 (ppm)

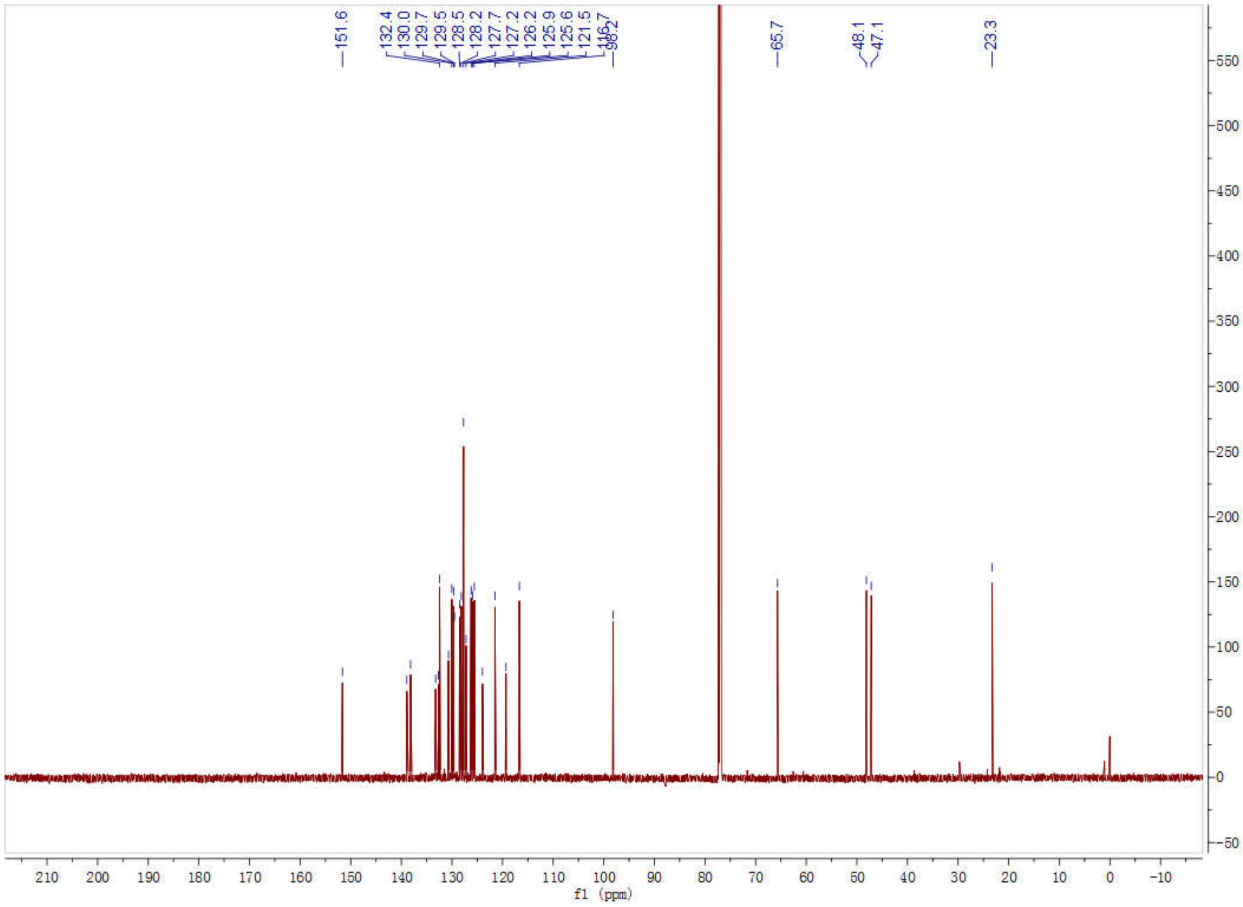
151.6
132.4 130.0 129.7 129.5 128.5 128.2 127.7 127.2 126.2 125.9 125.6 121.5 116.7 98.2
65.7
48.1 47.1
23.3
f1 (ppm)

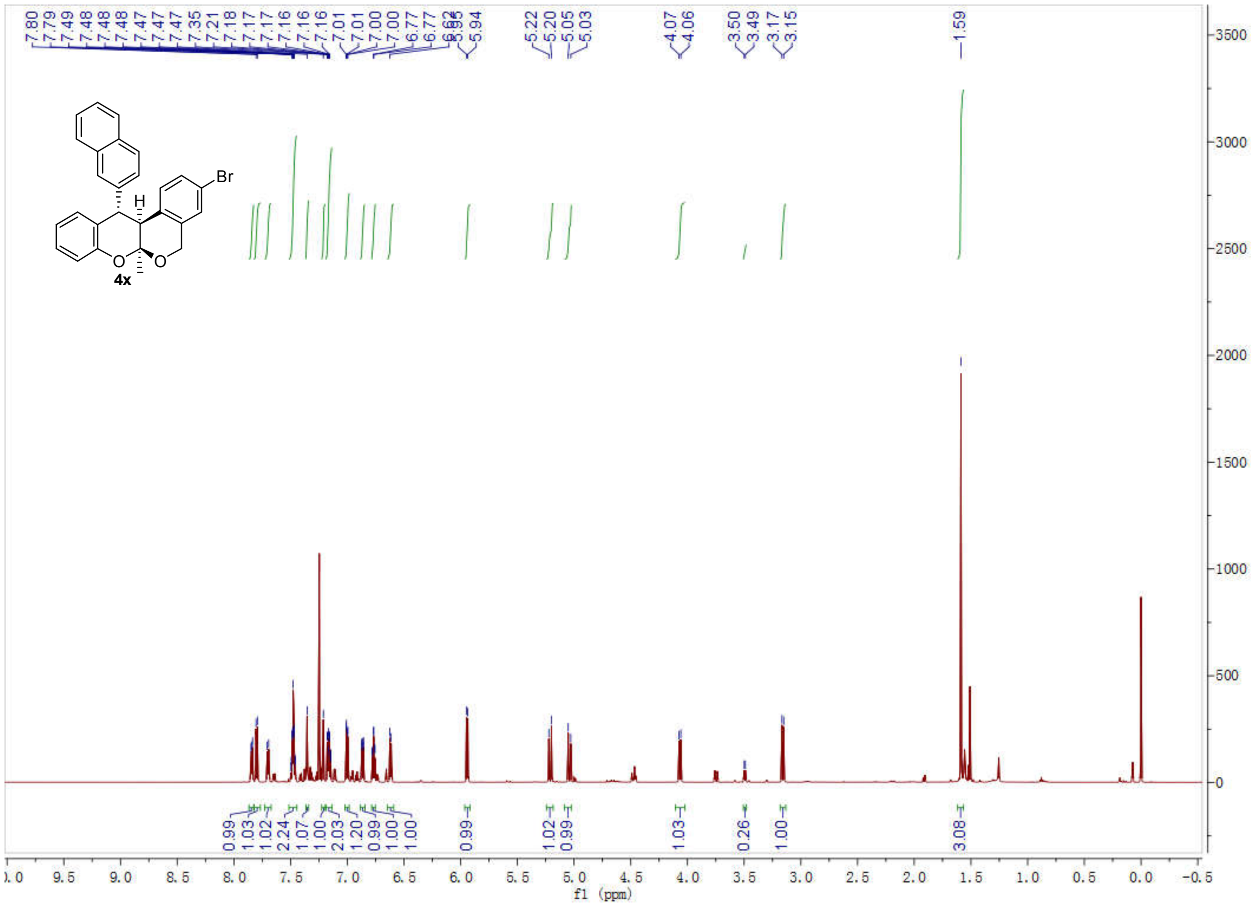

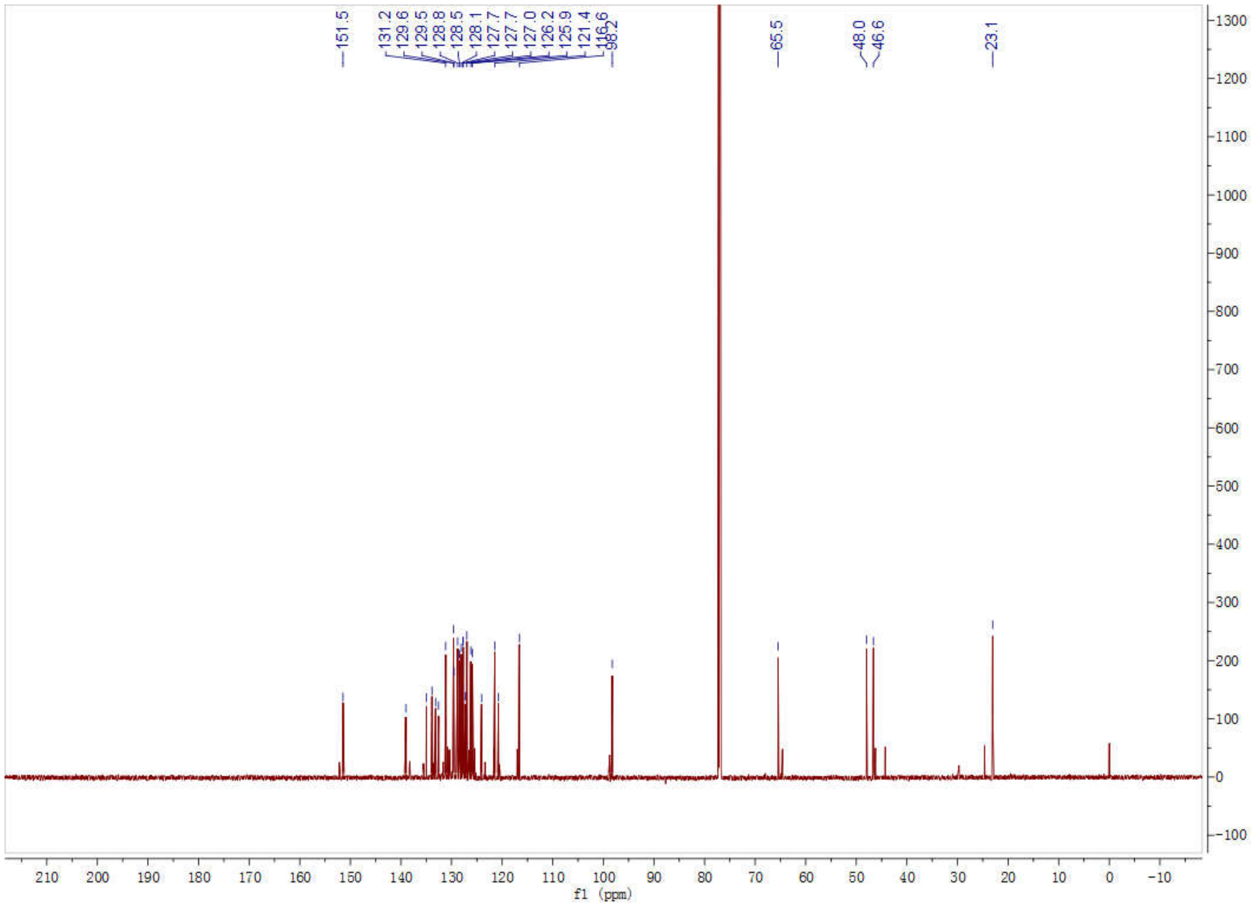

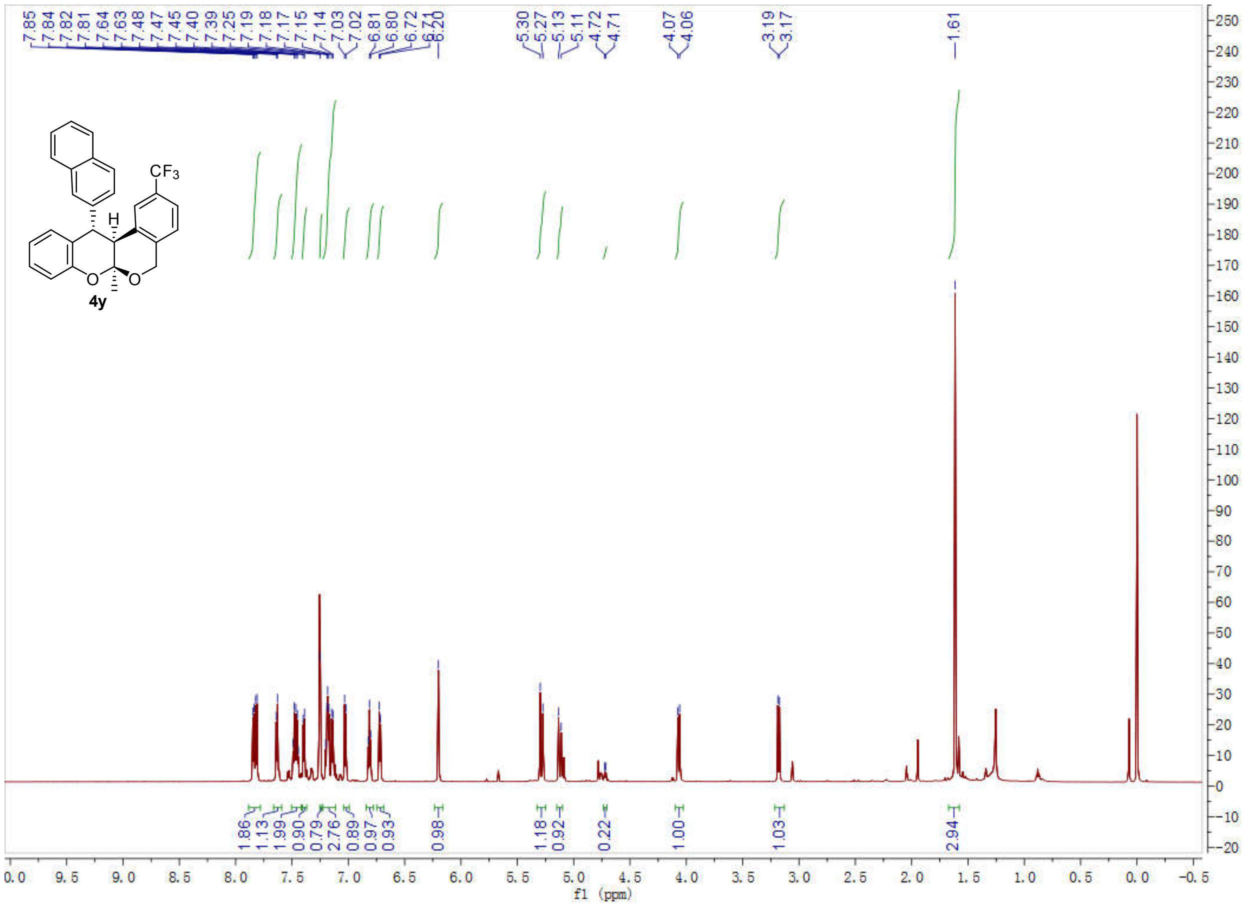
7.85 7.84 7.82 7.81 7.64 7.63 7.48 7.47 7.45 7.40 7.39 7.25 7.19 7.18 7.17 7.15 7.14 7.03 7.02 6.81 6.80 6.72 6.71 6.20
5.30 5.27 5.13 5.11 4.72 4.71
4.07 4.06
3.19 3.17
1.61
CF3
H
O
O
4y
1.86 1.13 1.99 0.90 0.79 2.76 0.89 0.97 0.93 0.98 1.18 0.92 0.22 1.00 1.03 2.94
f1 (ppm)

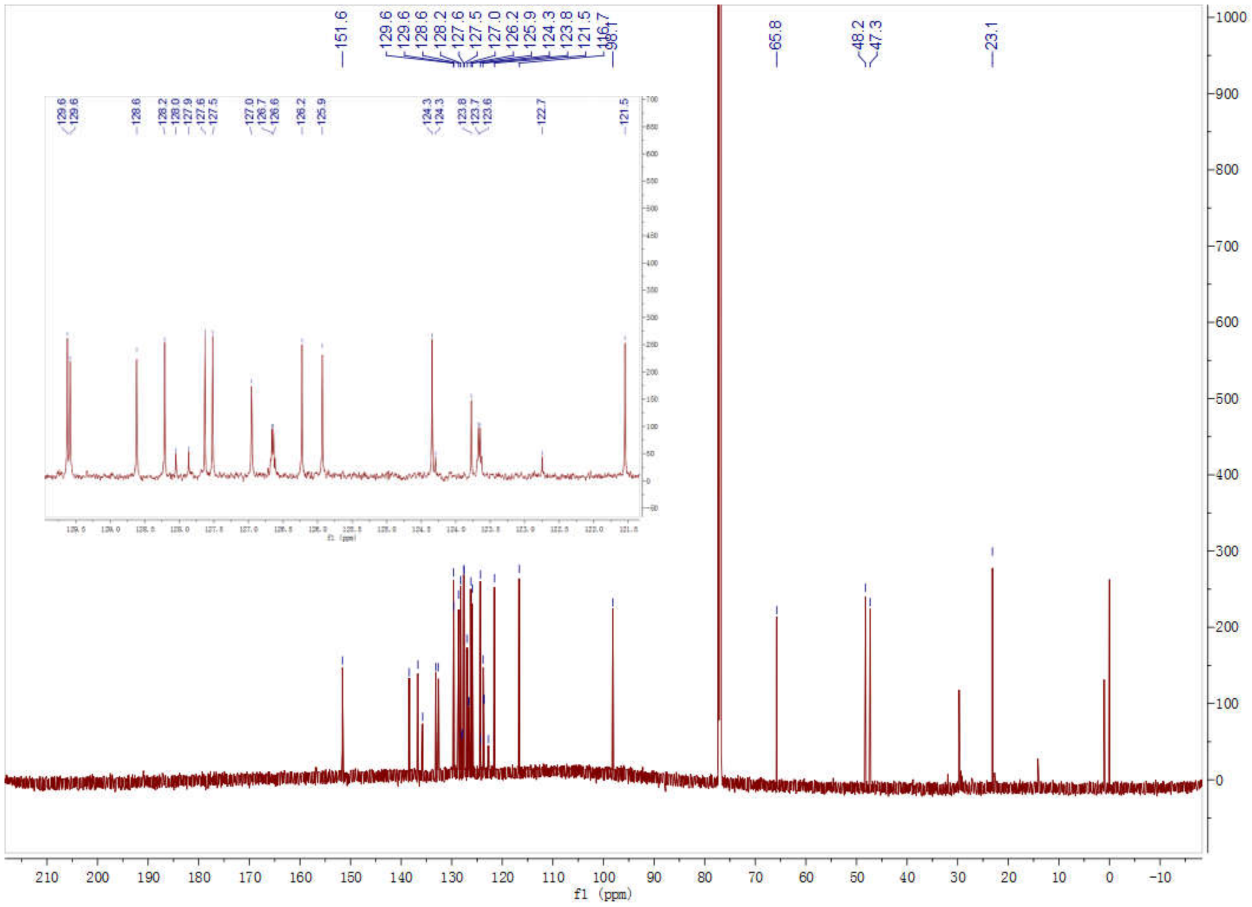
151.6
129.6 129.6 128.6 128.2 127.6 127.5 127.0 126.2 125.9 124.3 123.8 123.7 121.5 116.7 98.1
65.8
48.2 47.3
23.1
129.6 129.6 128.6 128.2 128.0 127.9 127.6 127.5 127.0 126.7 126.6 126.2 125.9 124.3 124.3 123.8 123.7 123.6 122.7 121.5
f1 (ppm)

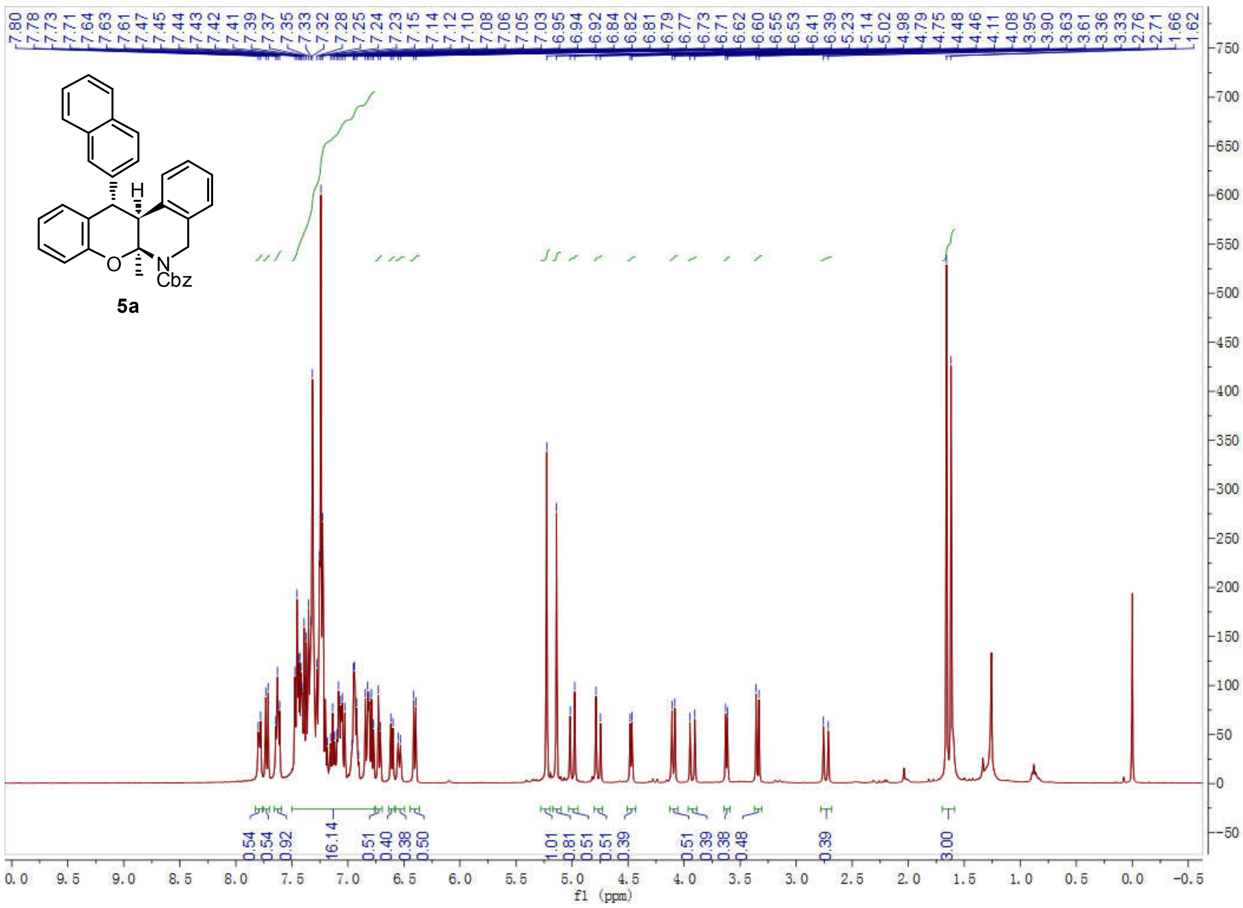
5a

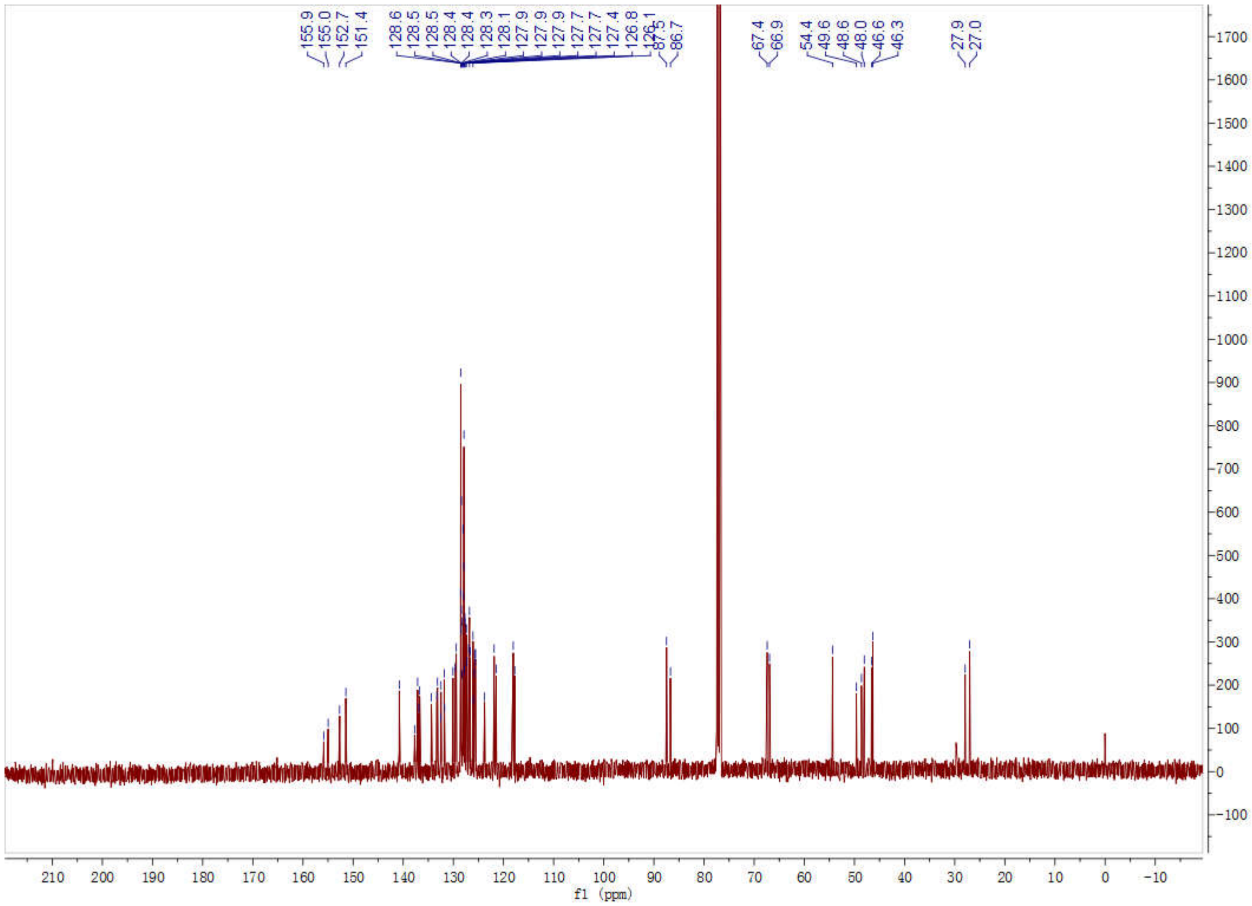

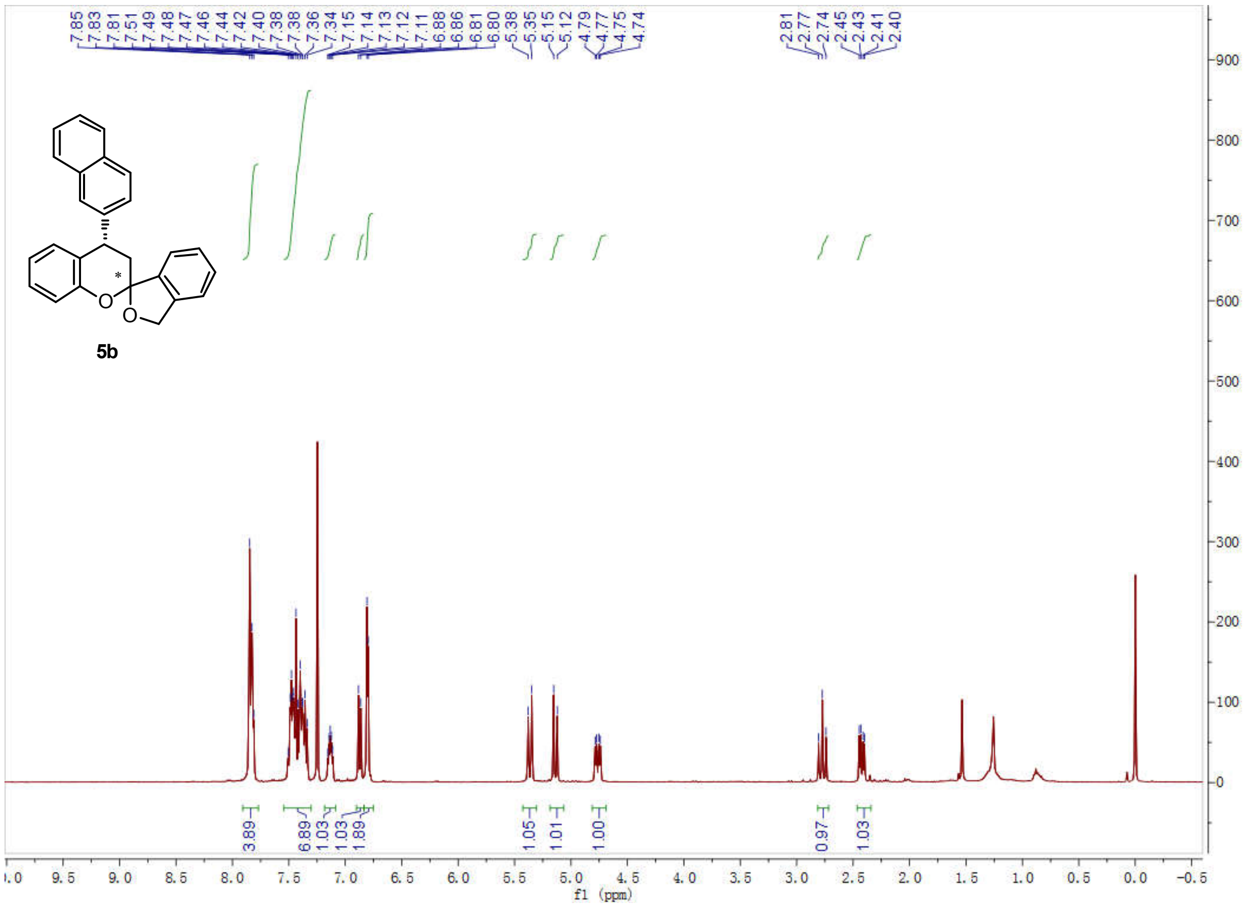
7.85
7.83
7.81
7.51
7.49
7.48
7.47
7.46
7.44
7.42
7.40
7.38
7.38
7.36
7.34
7.15
7.14
7.13
7.12
7.11
6.88
6.86
6.81
6.80
5.38
5.35
5.15
5.12
4.79
4.77
4.75
4.74
2.81
2.77
2.74
2.45
2.43
2.41
2.40
5b
3.89
6.89
1.03
1.03
1.89
1.05
1.01
1.00
0.97
1.03
f1 (ppm)

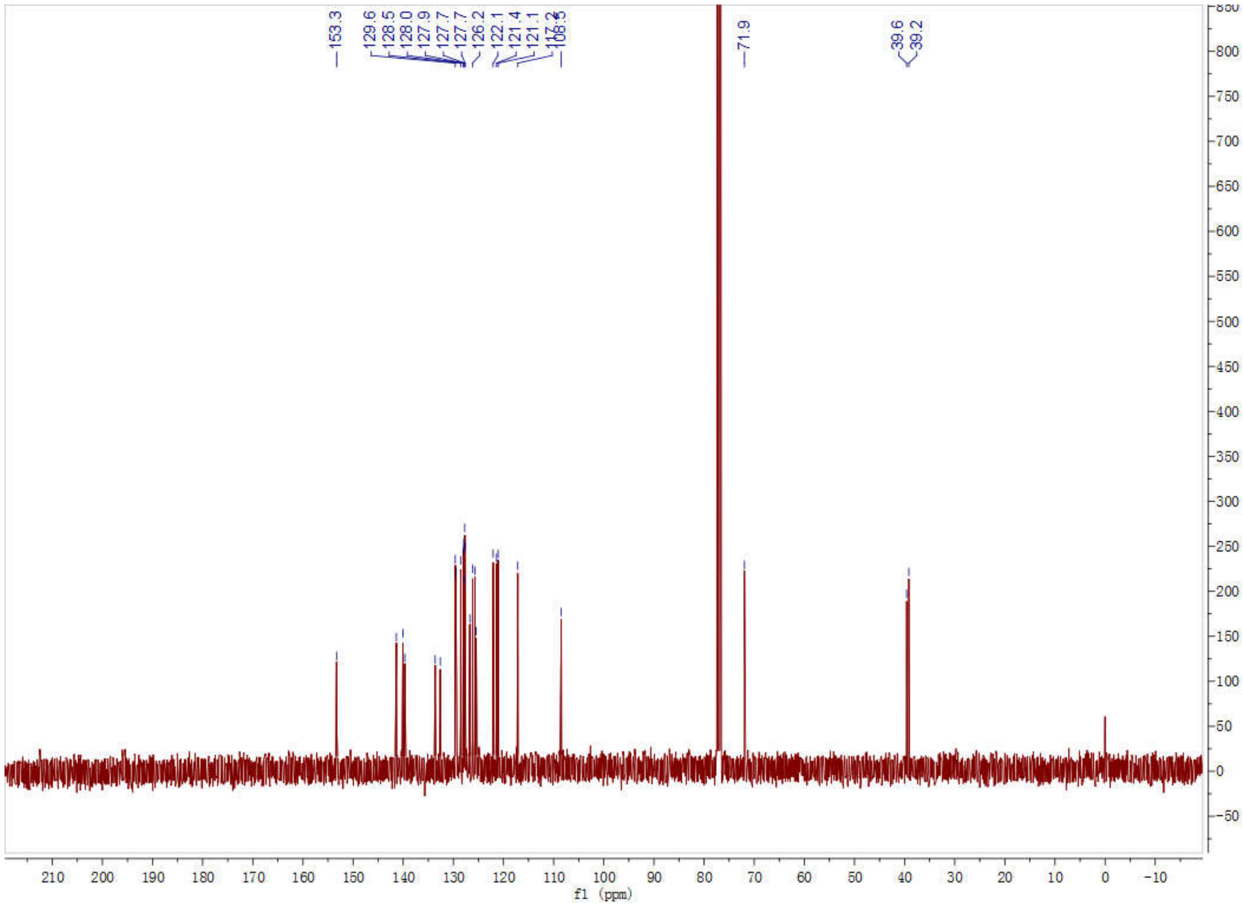
153.3
129.6
128.5
128.0
127.9
127.7
127.7
126.2
122.1
121.4
121.1
108.5
71.9
39.6
39.2
f1 (ppm)

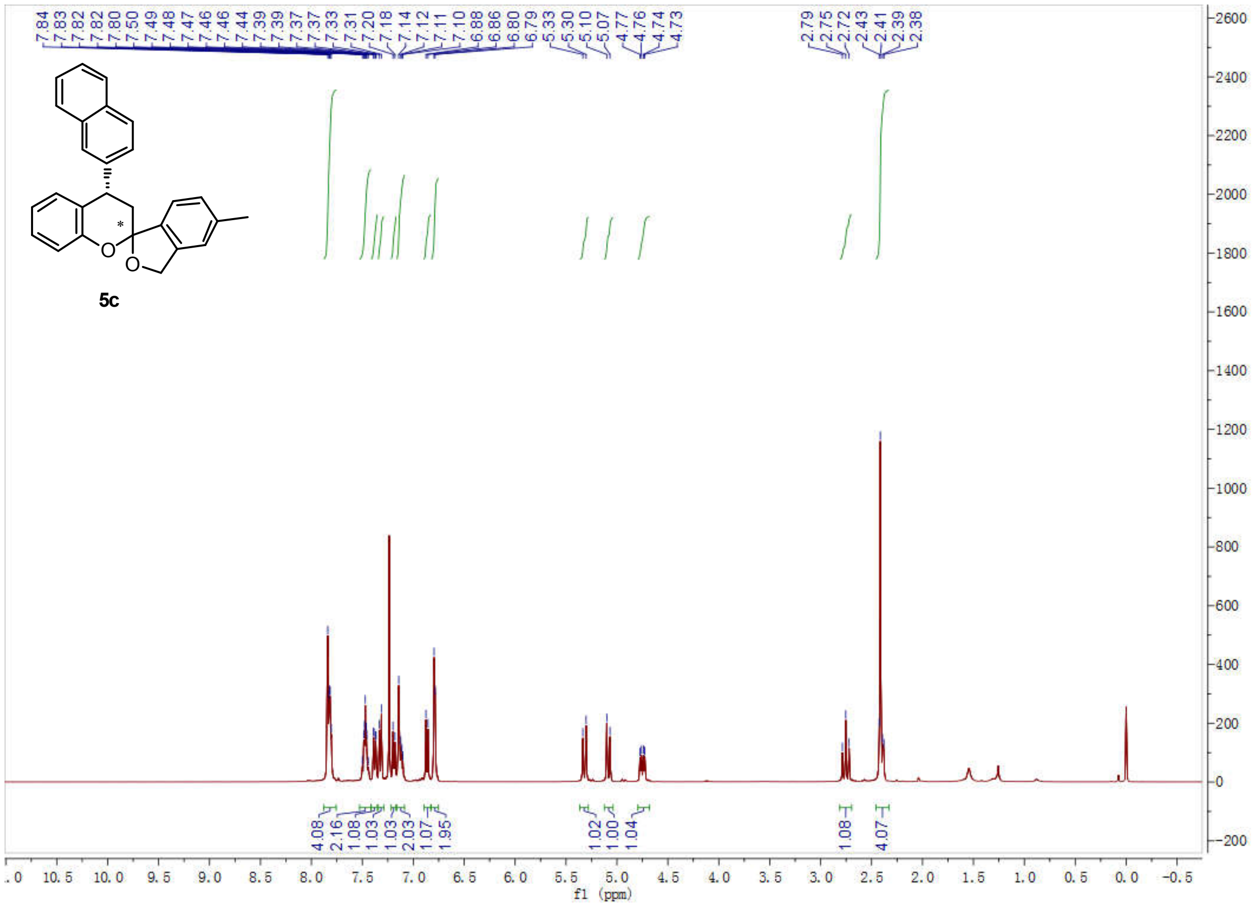

5c

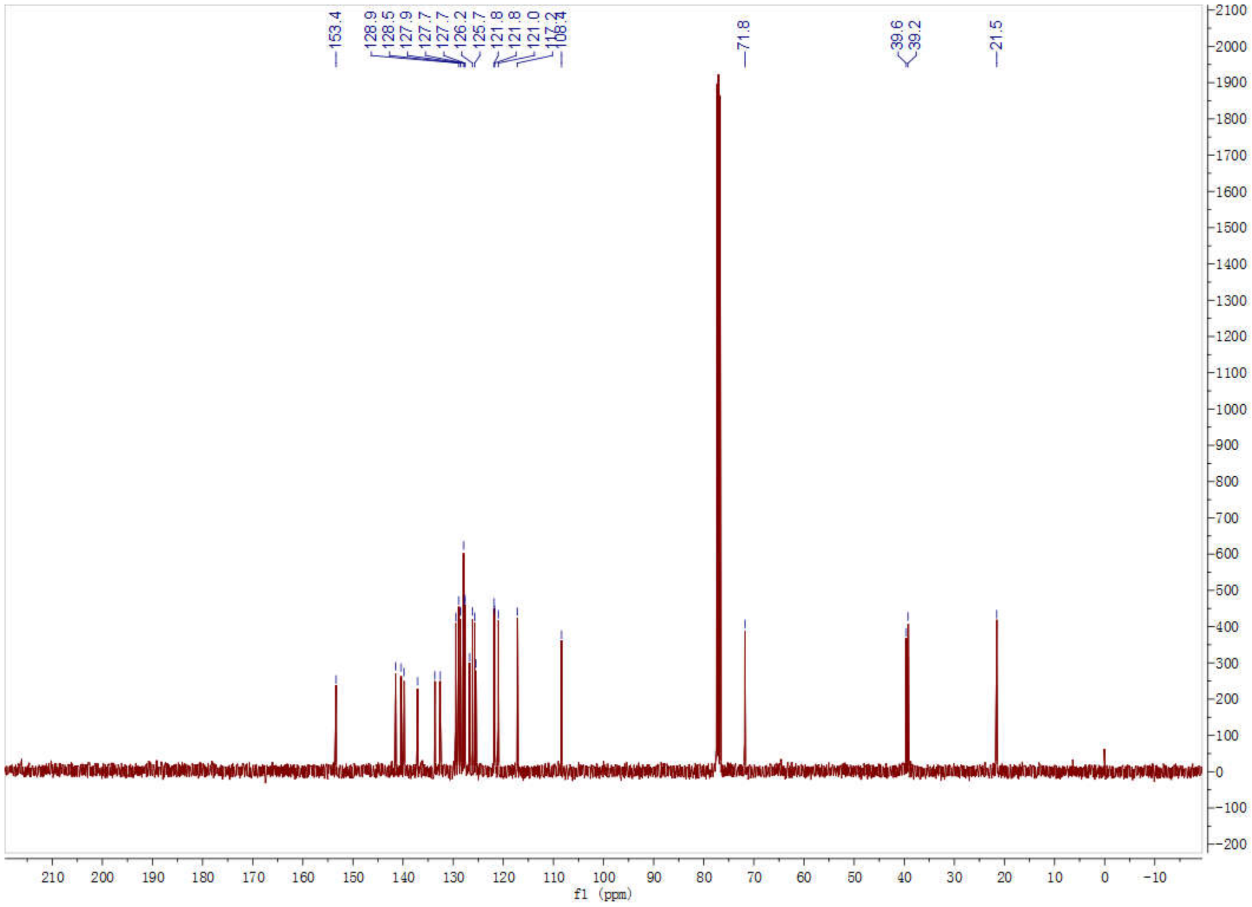

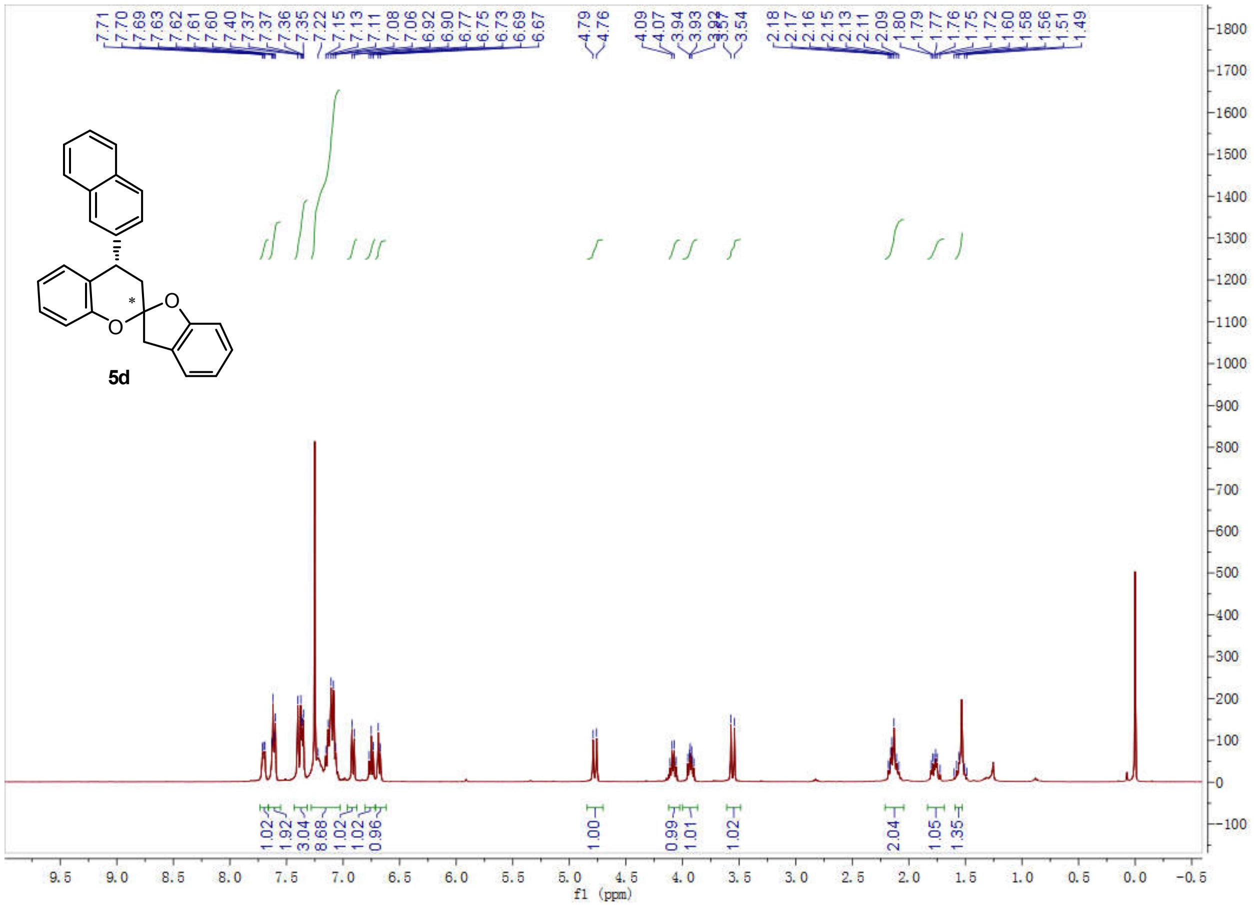
7.71 7.70 7.69 7.63 7.62 7.61 7.60 7.40 7.37 7.37 7.36 7.35 7.22 7.15 7.13 7.11 7.08 7.06 6.92 6.90 6.77 6.75 6.73 6.69 6.67
4.79 4.76
4.09 4.07 3.94 3.93 3.92 3.57 3.54
2.18 2.17 2.16 2.15 2.13 2.11 2.09 1.80 1.79 1.77 1.76 1.75 1.72 1.60 1.58 1.56 1.51 1.49
5d
1.02 1.92 3.04 8.68 1.02 1.02 0.96 1.00 0.99 1.01 1.02 2.04 1.05 1.35
f1 (ppm)

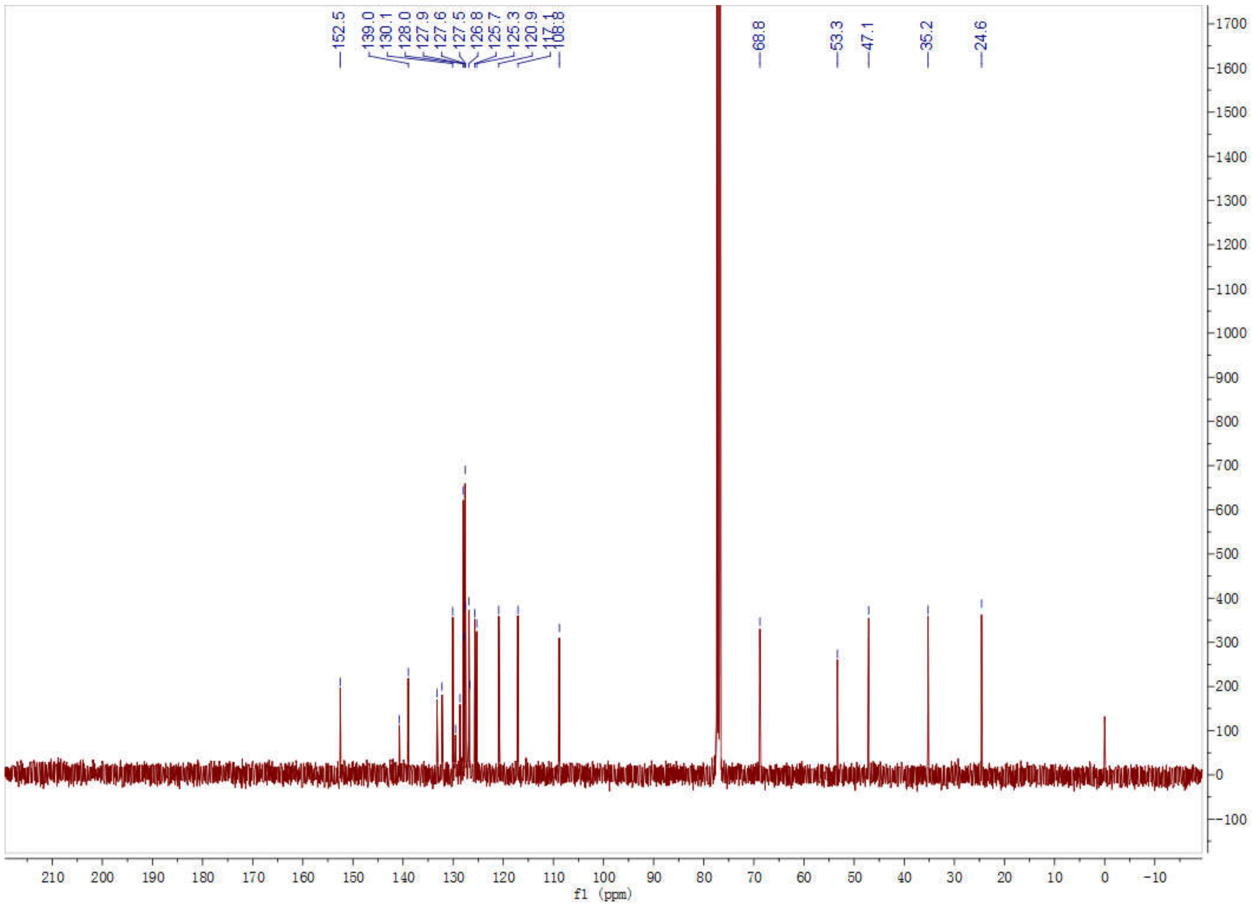
152.5 139.0 130.1 128.0 127.9 127.6 127.5 126.8 125.7 125.3 120.9 117.1 108.8
68.8 53.3 47.1 35.2 24.6
f1 (ppm)

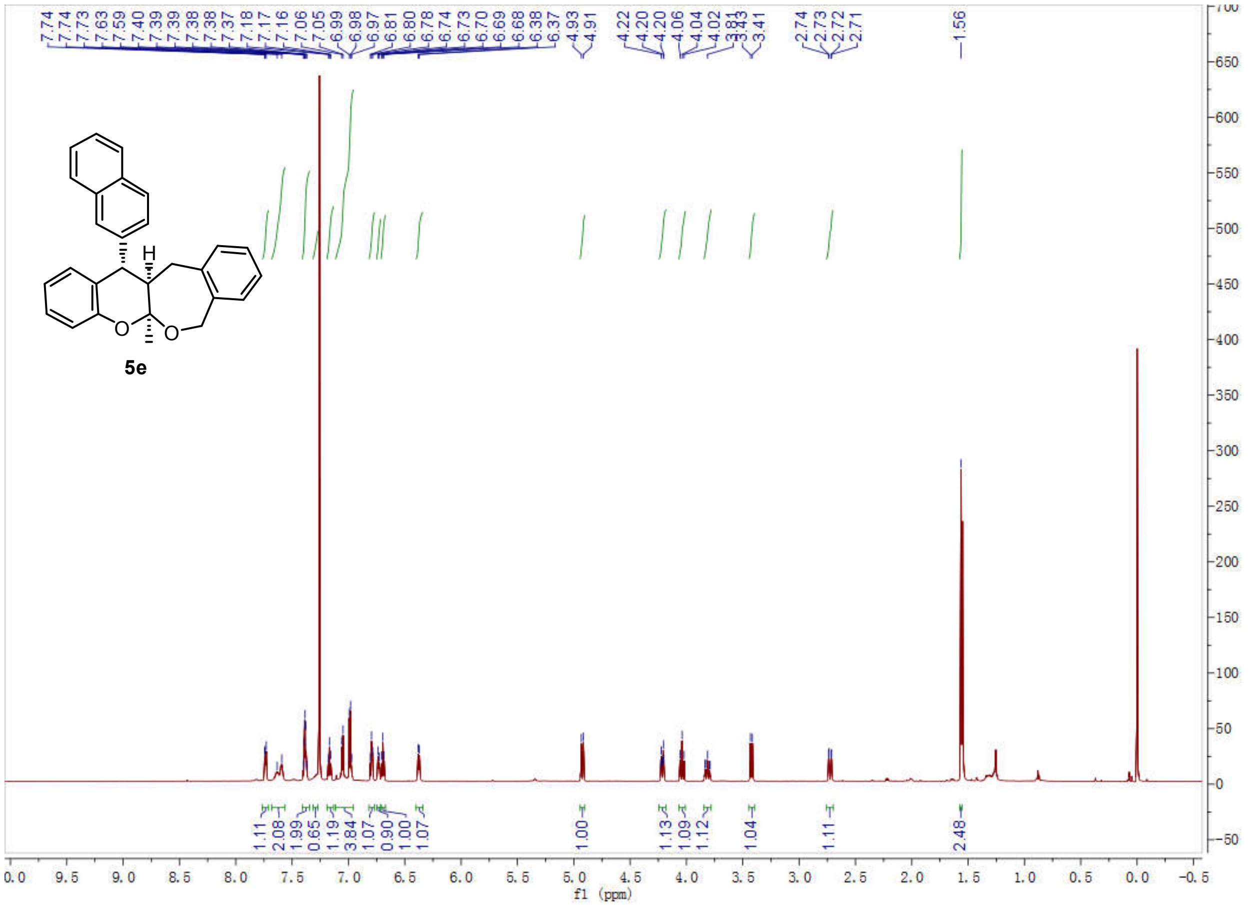
7.74
7.74
7.73
7.63
7.59
7.40
7.39
7.39
7.38
7.38
7.37
7.18
7.17
7.16
7.06
7.05
6.99
6.98
6.97
6.81
6.80
6.78
6.74
6.73
6.70
6.69
6.68
6.38
6.37
4.93
4.91
4.22
4.20
4.20
4.06
4.04
4.02
3.81
3.43
3.41
2.74
2.73
2.72
2.71
1.56
5e
1.11
2.08
1.99
0.65
1.19
3.84
1.07
0.90
1.00
1.07
1.00
1.13
1.09
1.12
1.04
1.11
2.48
f1 (ppm)

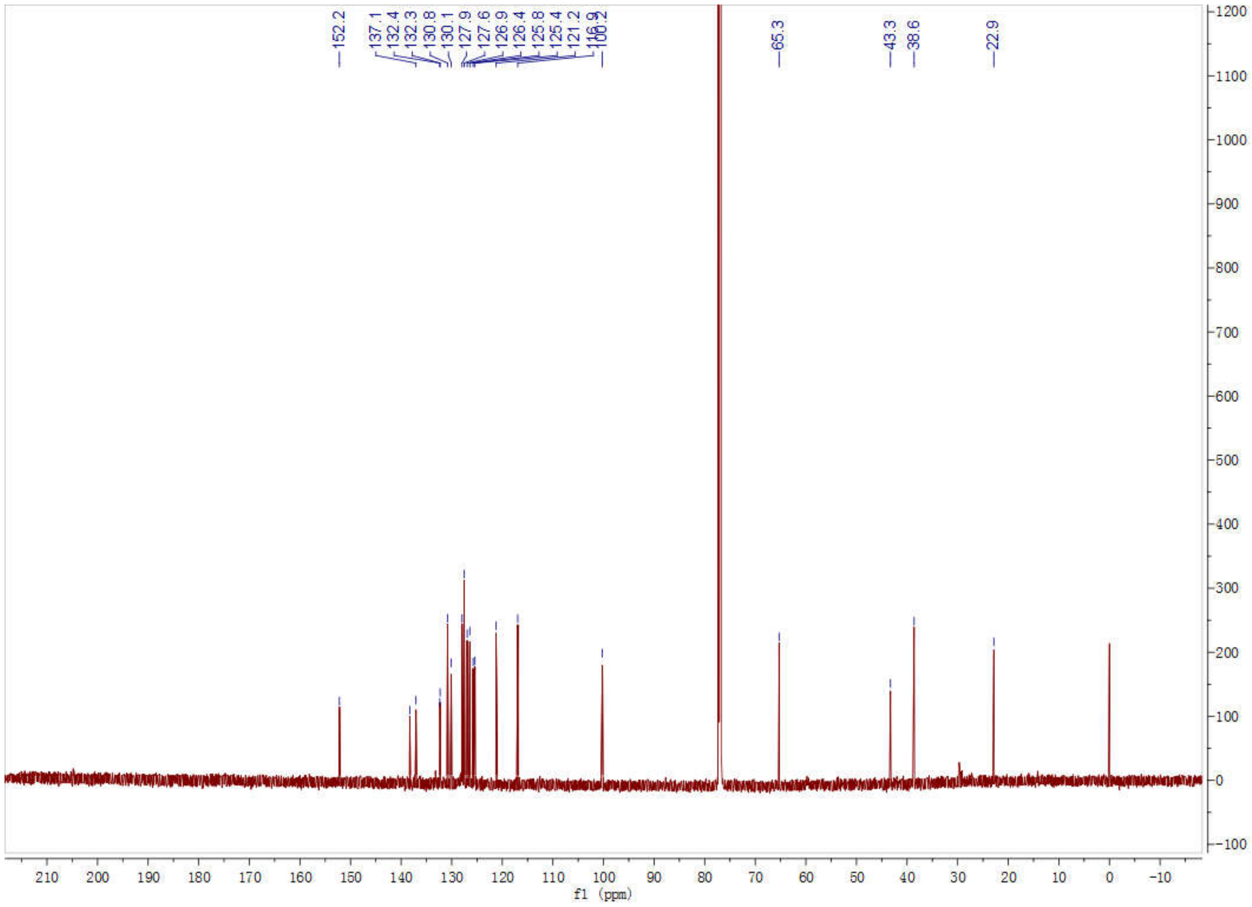
152.2
137.1
132.4
132.3
130.8
130.1
127.9
127.6
126.9
126.4
125.8
125.4
121.2
116.9
100.2
65.3
43.3
38.6
22.9
f1 (ppm)

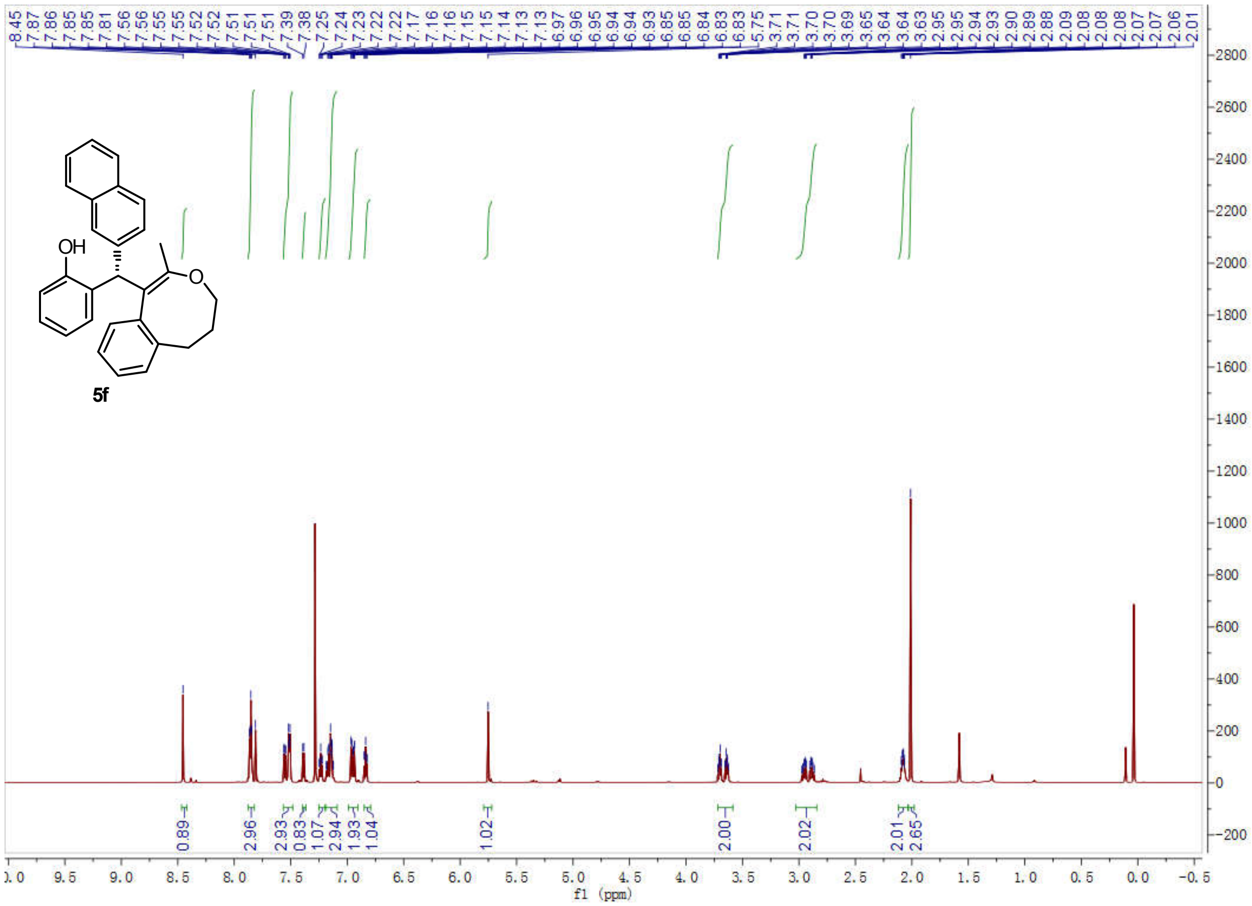
8.45 7.87 7.86 7.85 7.81 7.56 7.56 7.55 7.55 7.52 7.52 7.51 7.51 7.51 7.39 7.38 7.25 7.24 7.23 7.22 7.22 7.17 7.16 7.16 7.15 7.15 7.14 7.13 7.13 6.97 6.96 6.95 6.94 6.94 6.93 6.85 6.85 6.84 6.83 6.83 5.75 3.71 3.71 3.70 3.70 3.69 3.65 3.64 3.64 3.63 2.95 2.95 2.94 2.93 2.90 2.89 2.88 2.09 2.08 2.08 2.08 2.07 2.06 2.01
OH
O
5f
0.89 2.96 2.93 0.83 1.07 2.94 1.93 1.04 1.02 2.00 2.02 2.01 2.65
f1 (ppm)

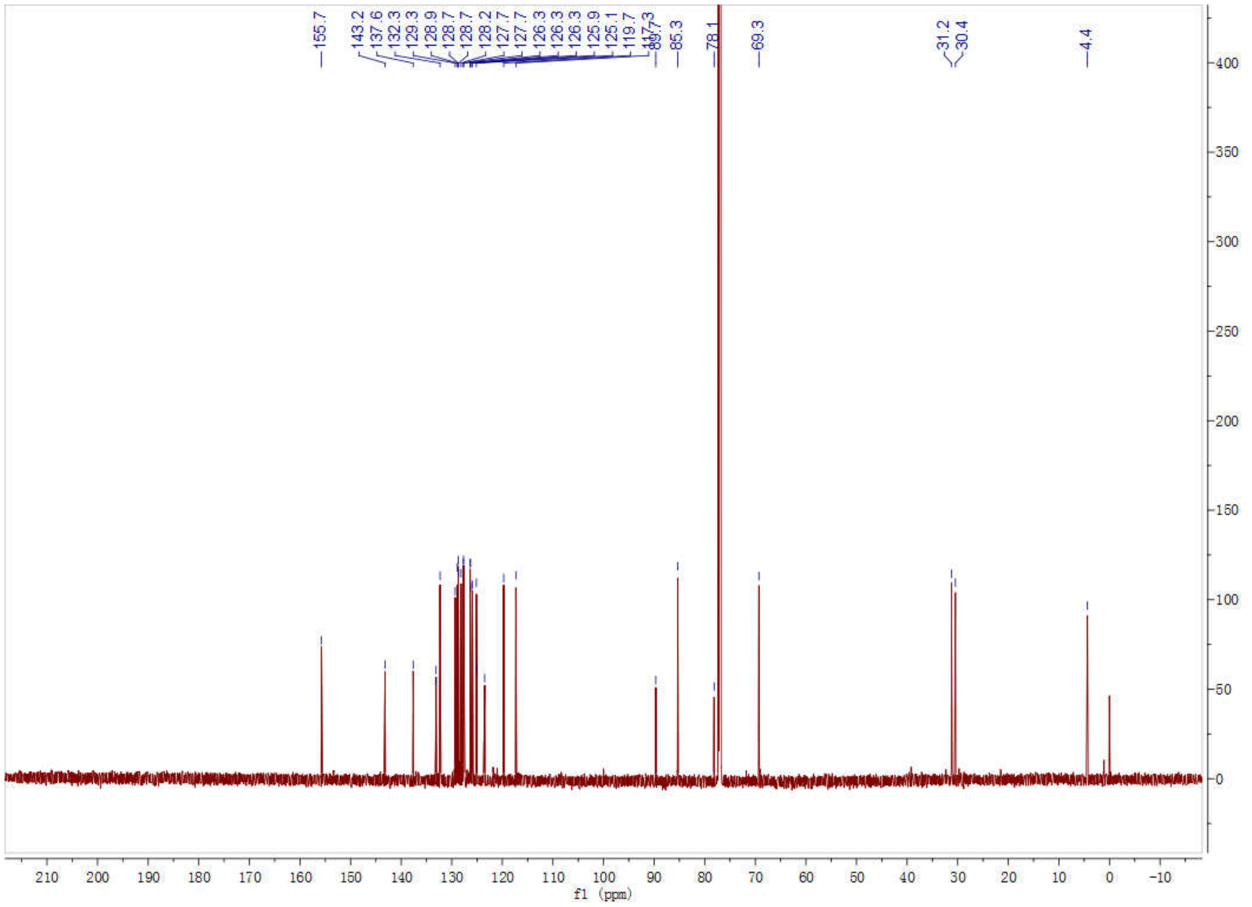
155.7 143.2 137.6 132.3 129.3 128.9 128.7 128.7 128.2 127.7 126.3 126.3 126.3 125.9 125.1 119.7 117.3 89.7 85.3 78.1 69.3 31.2 30.4 4.4
f1 (ppm)

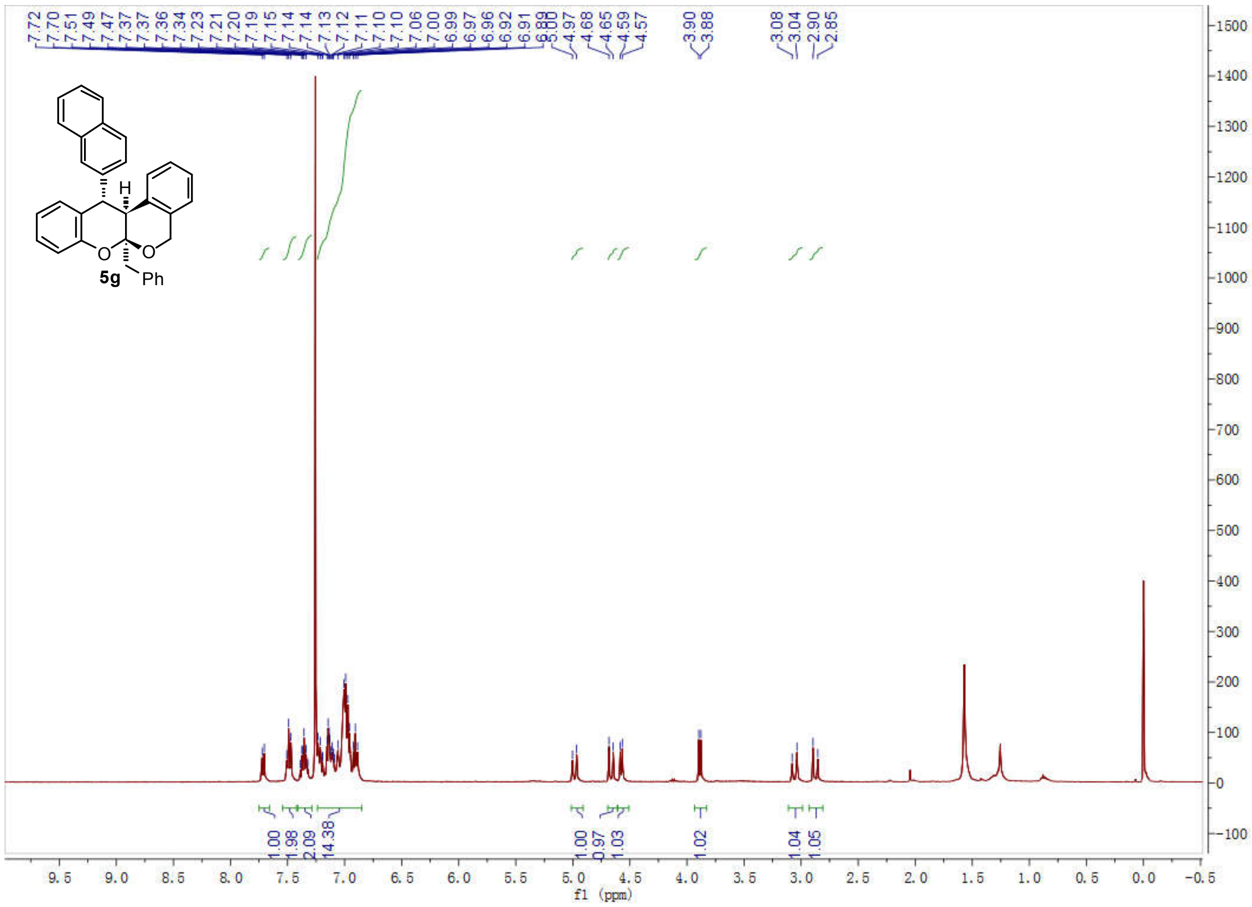
7.72 7.70 7.51 7.49 7.47 7.37 7.36 7.34 7.23 7.21 7.20 7.19 7.15 7.14 7.13 7.12 7.11 7.10 7.10 7.06 7.00 6.99 6.97 6.96 6.92 6.91 6.89 5.00 4.97 4.68 4.65 4.59 4.57
3.90 3.88
3.08 3.04 2.90 2.85
5g
Ph
H
O
1.00 1.98 2.09 14.38 1.00 0.97 1.03 1.02 1.04 1.05
f1 (ppm)

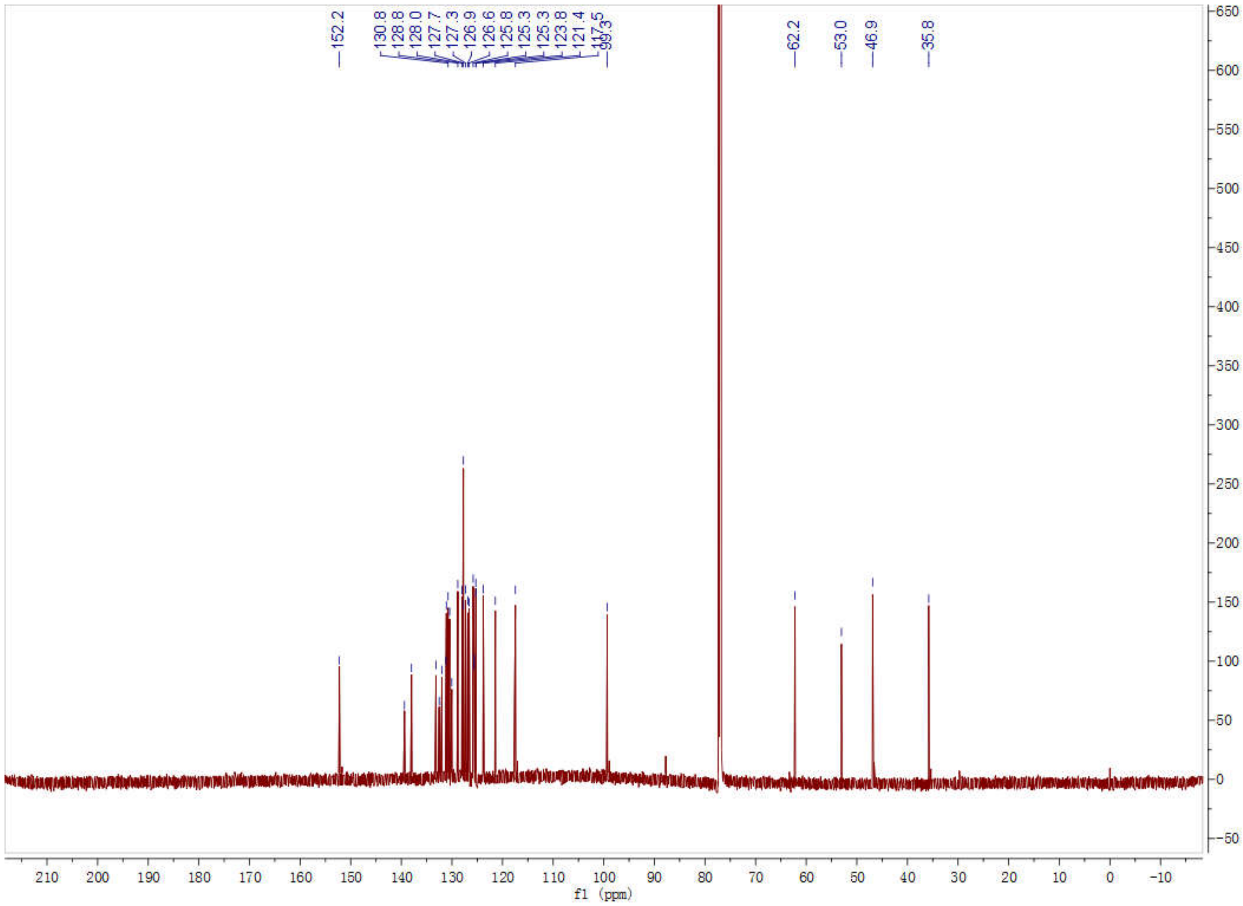
152.2
130.8 128.8 128.0 127.7 127.3 126.9 126.6 125.8 125.3 125.3 123.8 121.4 117.5 99.3
62.2
53.0
46.9
35.8
f1 (ppm)

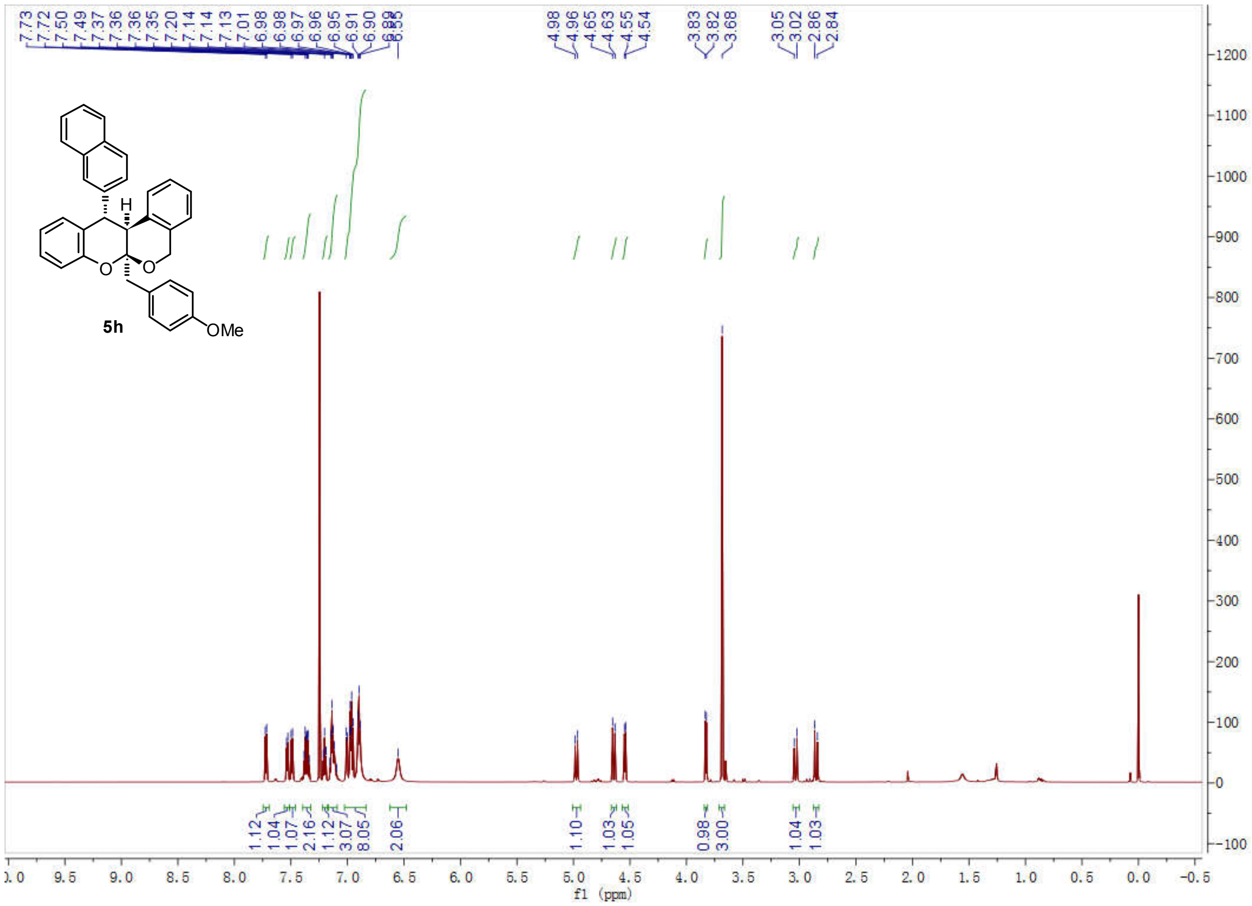
7.73
7.72
7.50
7.49
7.37
7.36
7.36
7.35
7.20
7.14
7.14
7.13
7.01
6.98
6.98
6.97
6.96
6.95
6.91
6.90
4.98
4.96
4.65
4.63
4.55
4.54
3.83
3.82
3.68
3.05
3.02
2.86
2.84
5h
OMe
1.12
1.04
1.07
2.16
1.12
3.07
8.05
2.06
1.10
1.03
1.05
0.98
3.00
1.04
1.03
f1 (ppm)

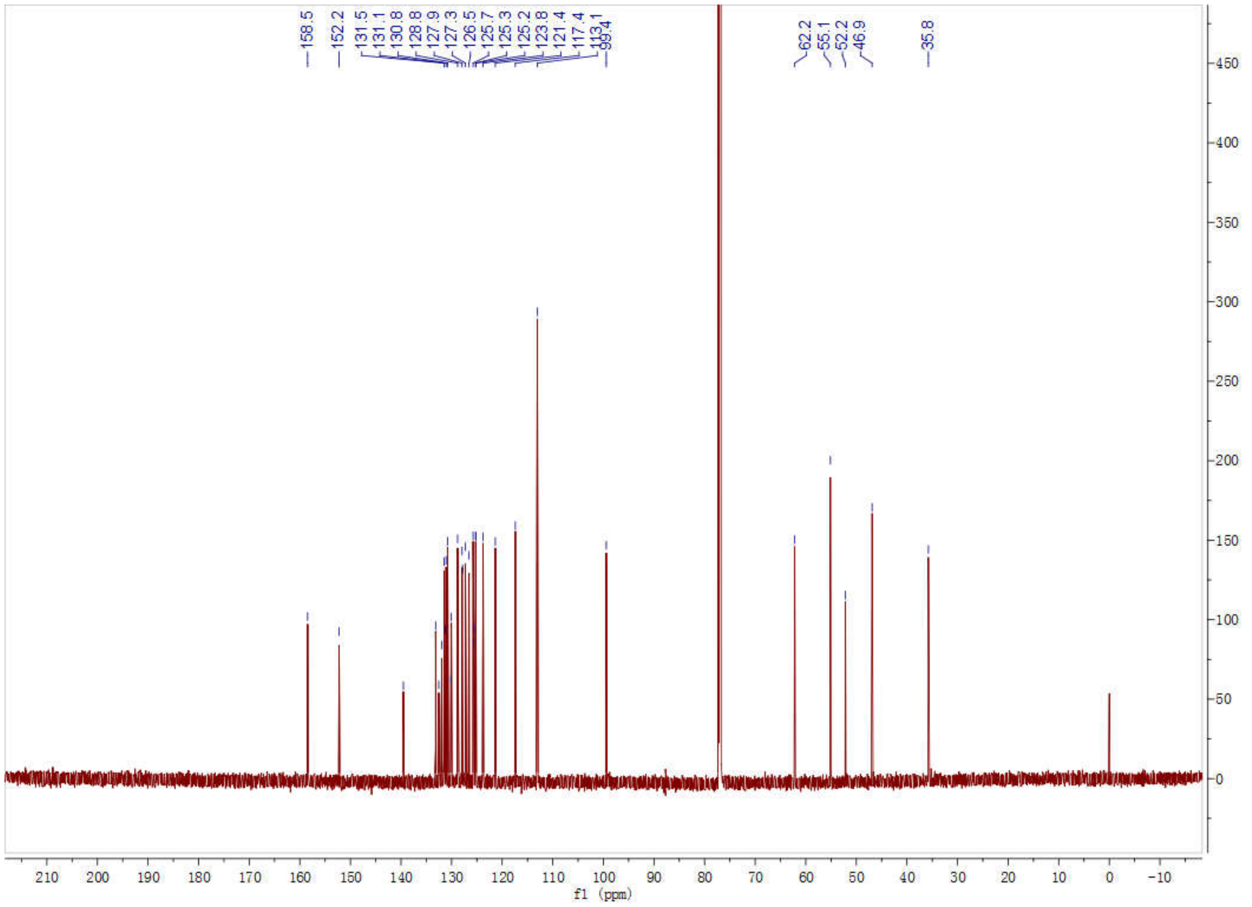
158.5
152.2
131.5
131.1
130.8
128.8
127.9
127.3
126.5
125.7
125.3
125.2
123.8
121.4
117.4
113.1
99.4
62.2
55.1
52.2
46.9
35.8
f1 (ppm)

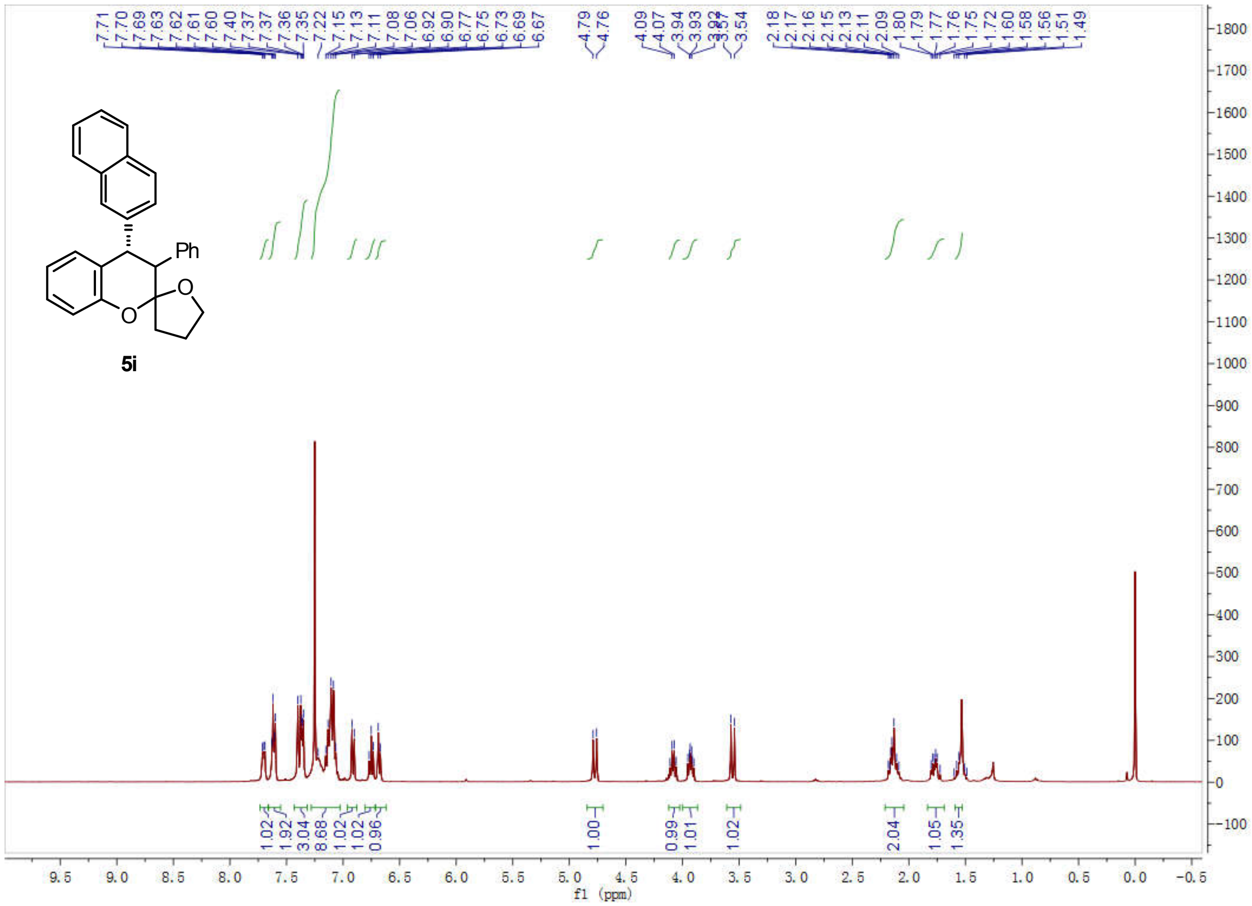
7.71 7.70 7.69 7.63 7.62 7.61 7.60 7.40 7.37 7.37 7.36 7.35 7.22 7.15 7.13 7.11 7.08 7.06 6.92 6.90 6.77 6.75 6.73 6.69 6.67
4.79 4.76
4.09 4.07 3.94 3.93 3.92 3.57 3.54
2.18 2.17 2.16 2.15 2.13 2.11 2.09 1.80 1.79 1.77 1.76 1.75 1.72 1.60 1.58 1.56 1.51 1.49
Ph
O
O
5i
1.02 1.92 3.04 8.68 1.02 1.02 0.96 1.00 0.99 1.01 1.02 2.04 1.05 1.35
f1 (ppm)

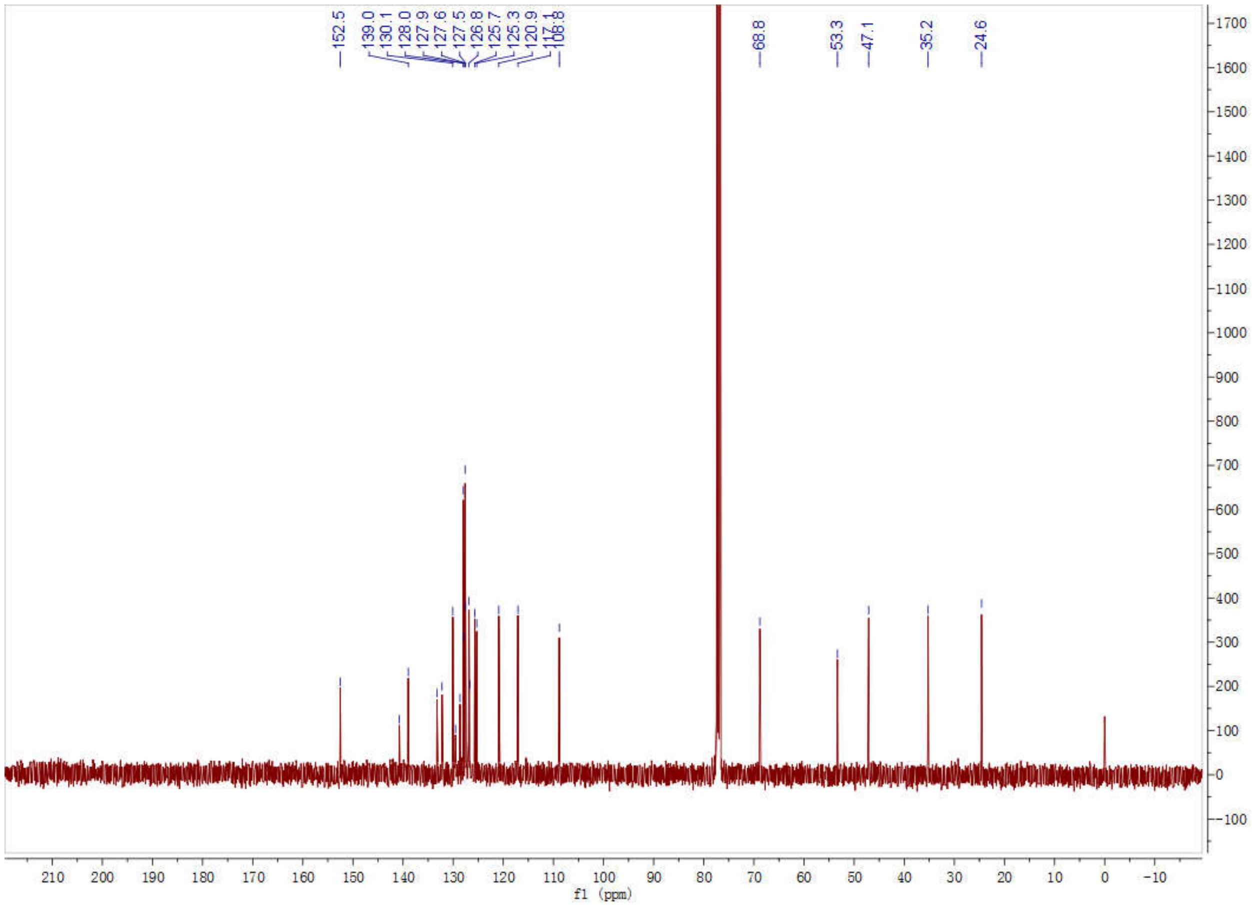
152.5
139.0 130.1 128.0 127.9 127.6 127.5 126.8 125.7 125.3 120.9 117.1 108.8
68.8
53.3
47.1
35.2
24.6
f1 (ppm)

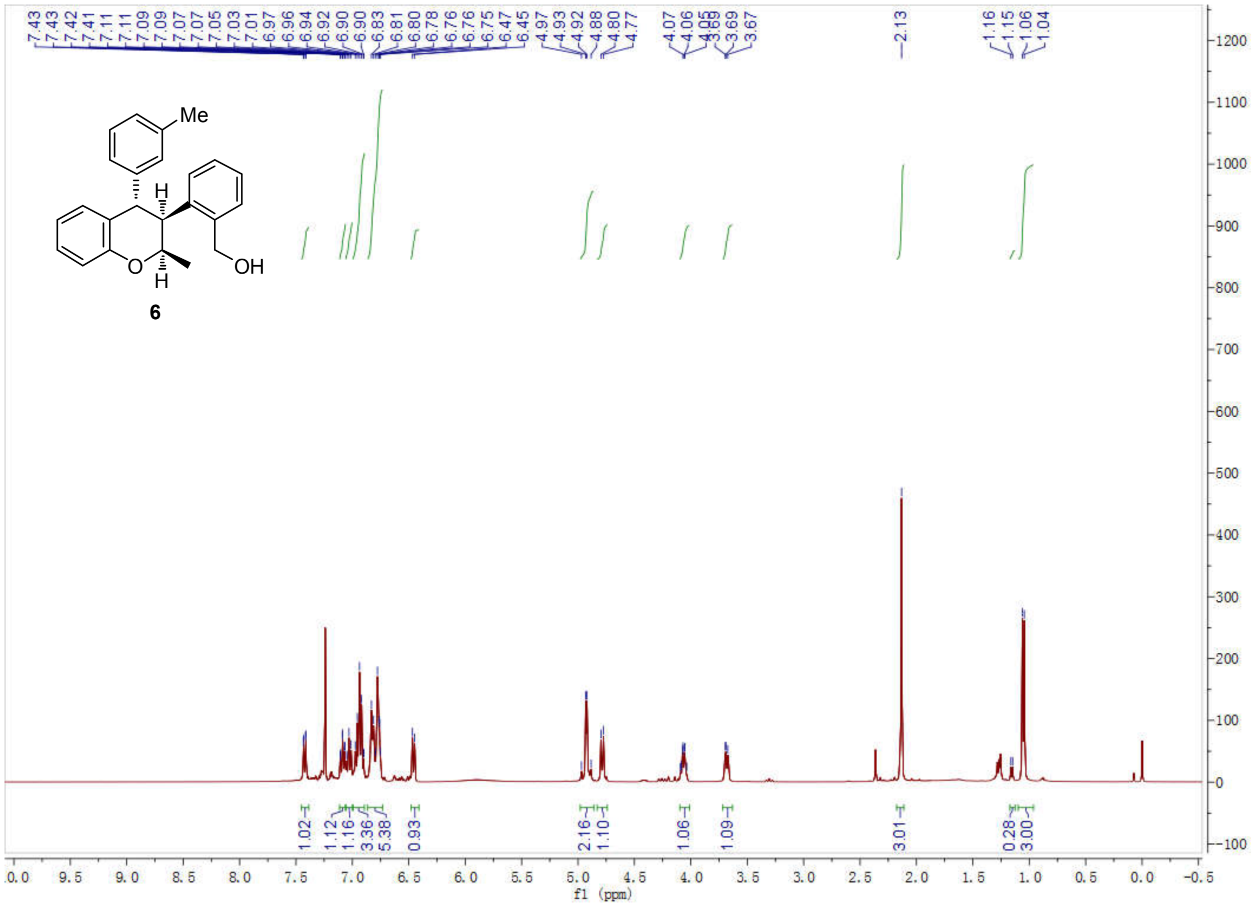
7.43 7.43 7.42 7.41 7.11 7.09 7.09 7.07 7.07 7.05 7.03 7.01 6.97 6.96 6.94 6.92 6.90 6.90 6.83 6.81 6.80 6.78 6.76 6.76 6.75 6.47 6.45 4.97 4.93 4.92 4.88 4.80 4.77 4.07 4.06 3.69 3.67 2.13 1.16 1.15 1.06 1.04
Me
H
O
H
OH
6
1.02 1.12 1.16 3.36 5.38 0.93 2.16 1.10 1.06 1.09 3.01 0.28 3.00
f1 (ppm)

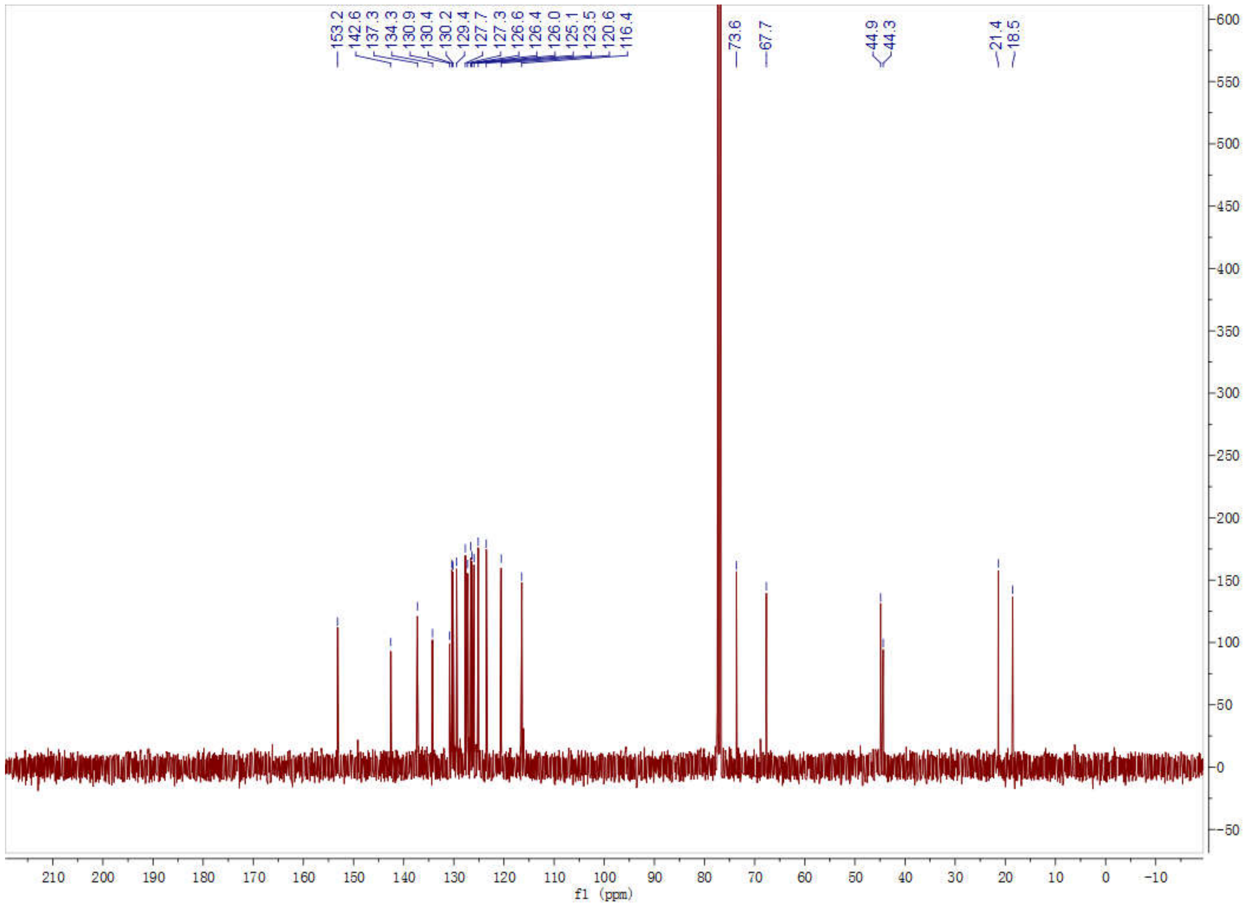
153.2 142.6 137.3 134.3 130.9 130.4 130.2 129.4 127.7 127.3 126.6 126.4 126.0 125.1 123.5 120.6 116.4 73.6 67.7 44.9 44.3 21.4 18.5
f1 (ppm)

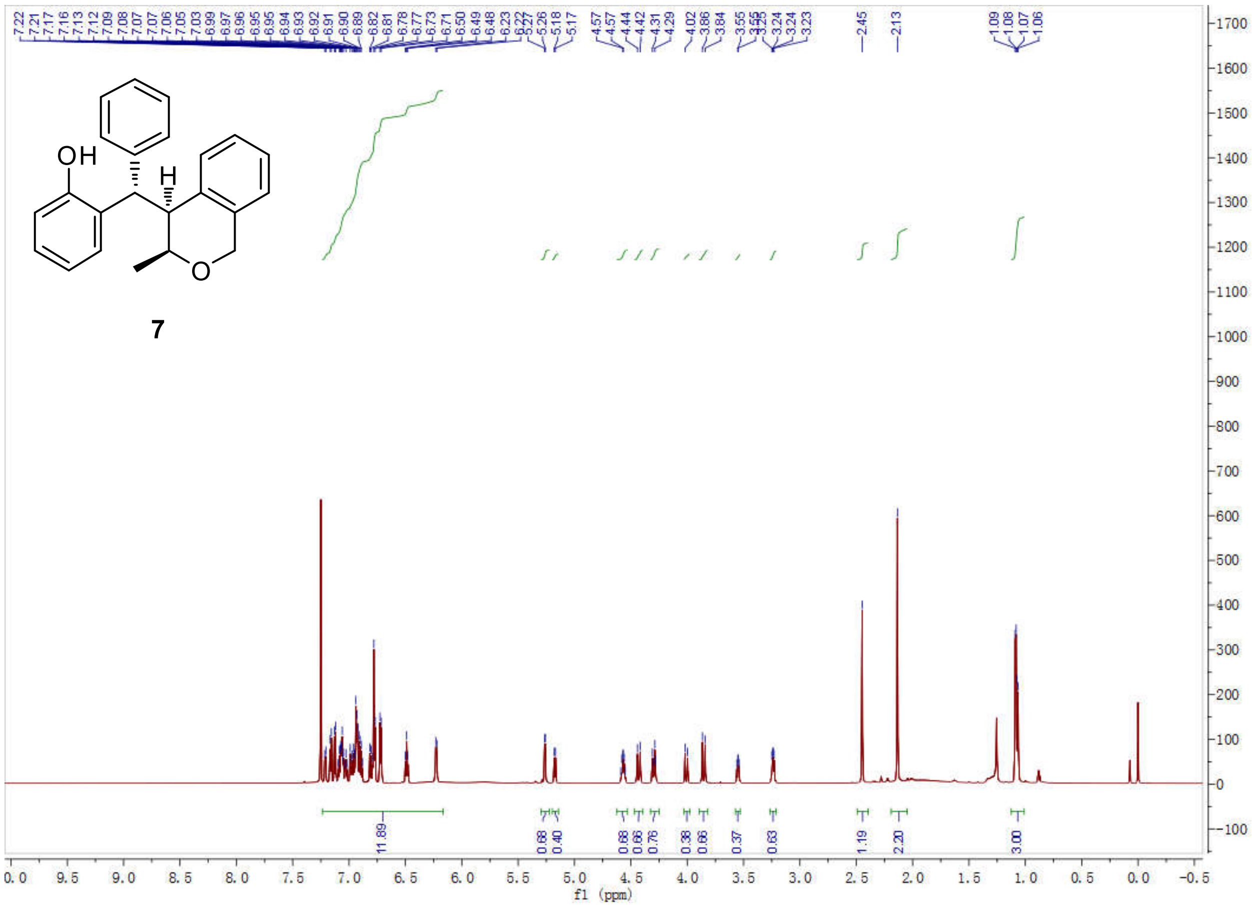
OH
H
O
7
f1 (ppm)

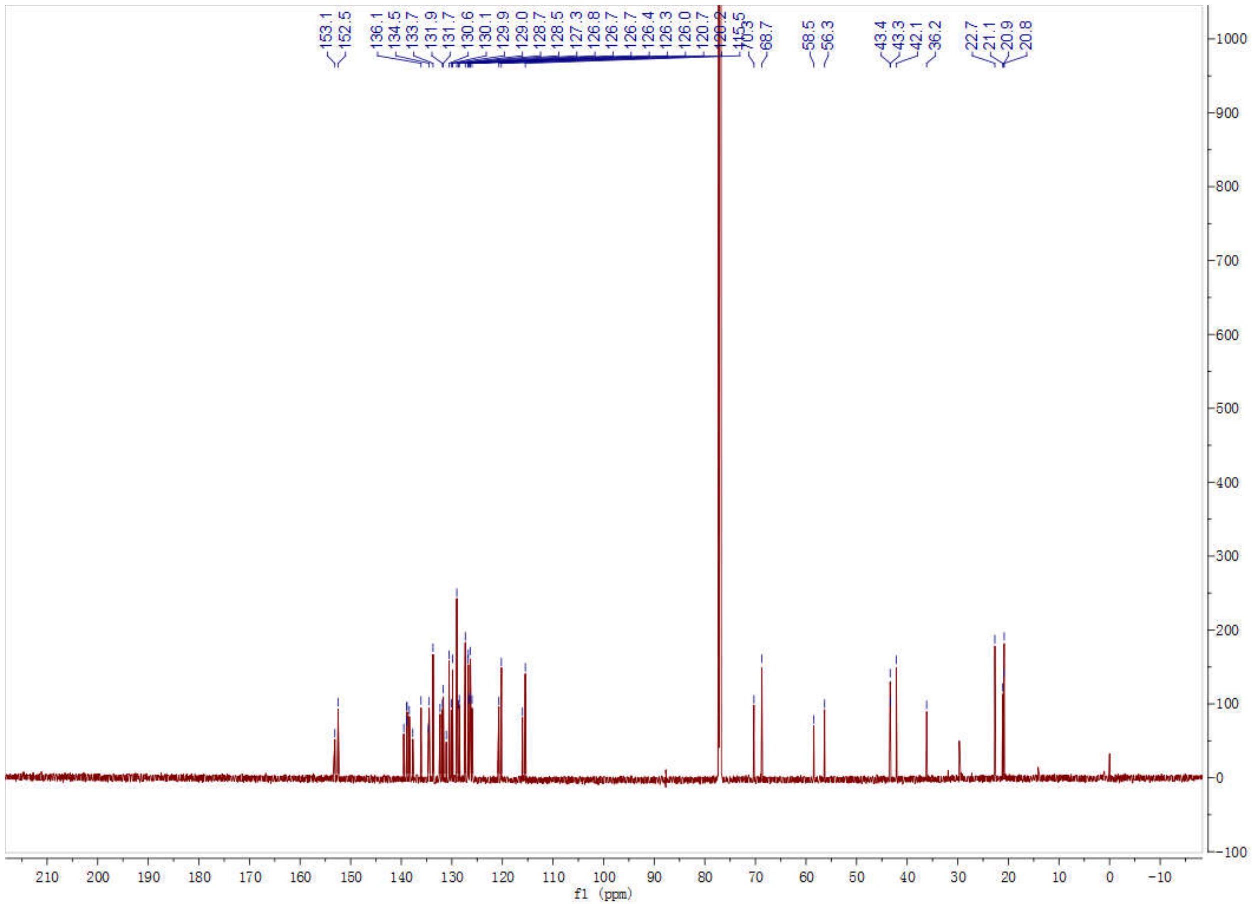
f1 (ppm)

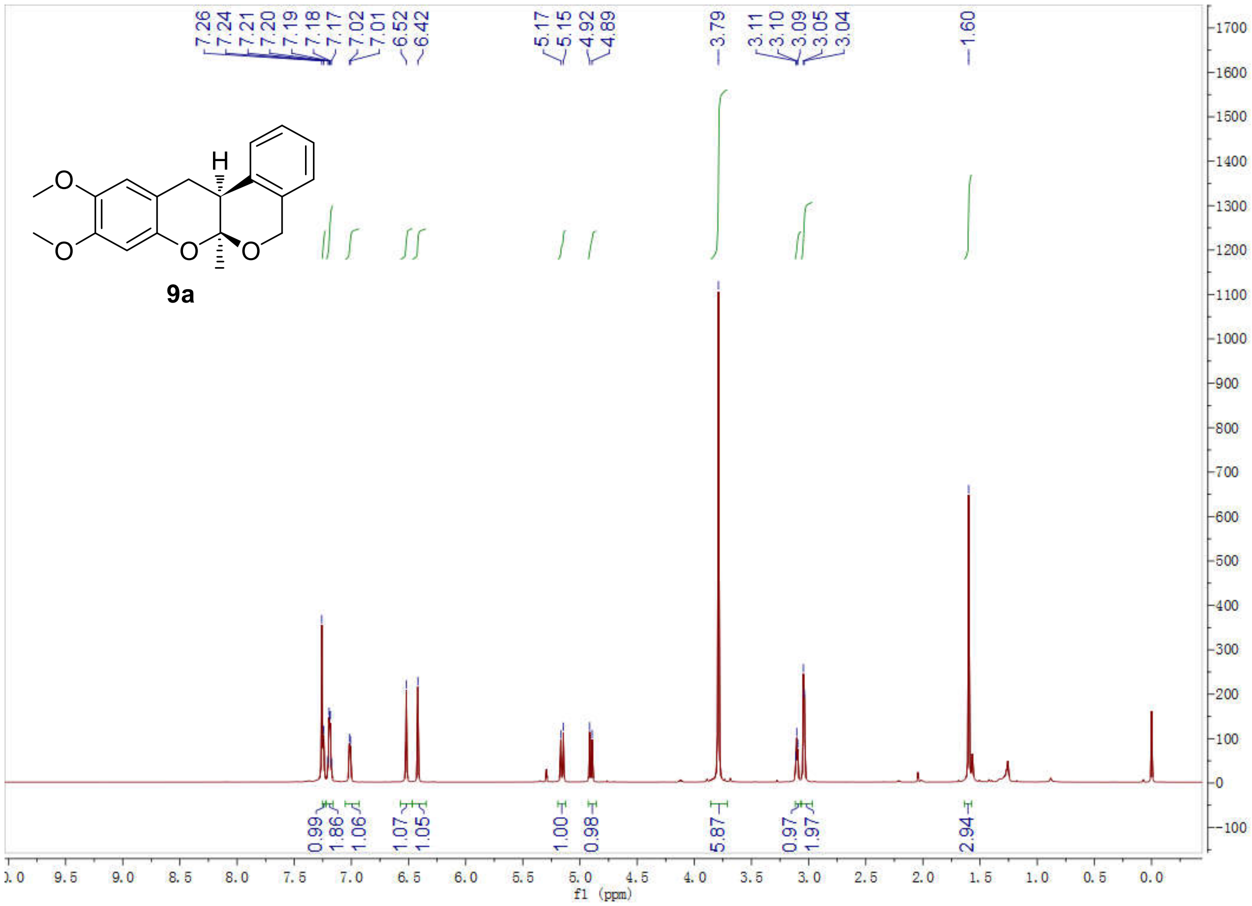
7.26 7.24 7.21 7.20 7.19 7.18 7.17 7.02 7.01 6.52 6.42
5.17 5.15 4.92 4.89
3.79
3.11 3.10 3.09 3.05 3.04
1.60
9a
0.99 1.86 1.06 1.07 1.05 1.00 0.98 5.87 0.97 1.97 2.94
f1 (ppm)

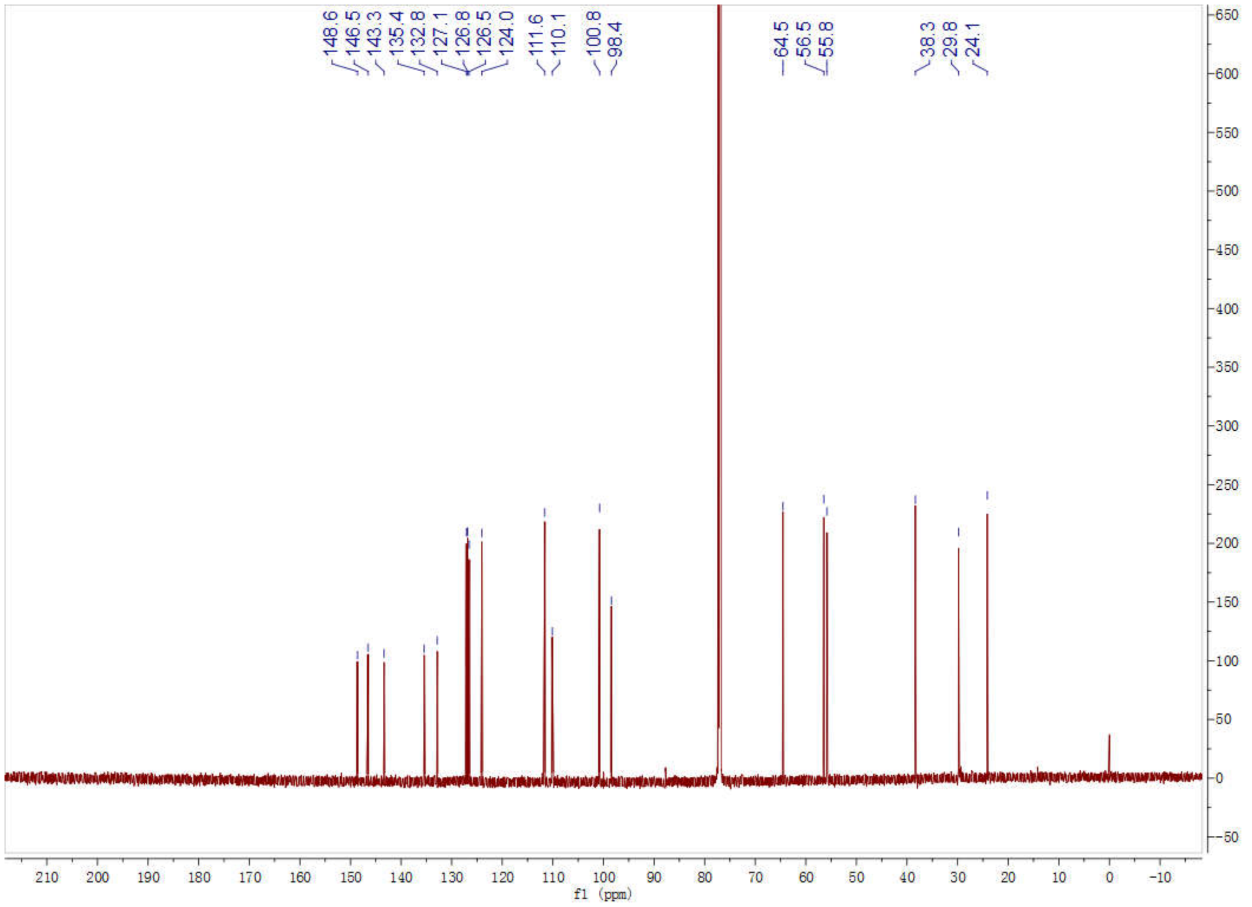
148.6 146.5 143.3 135.4 132.8 127.1 126.8 126.5 124.0 111.6 110.1 100.8 98.4
64.5 56.5 55.8
38.3 29.8 24.1
f1 (ppm)

8. HPLC spectra

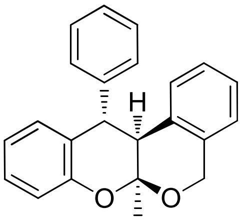

4a

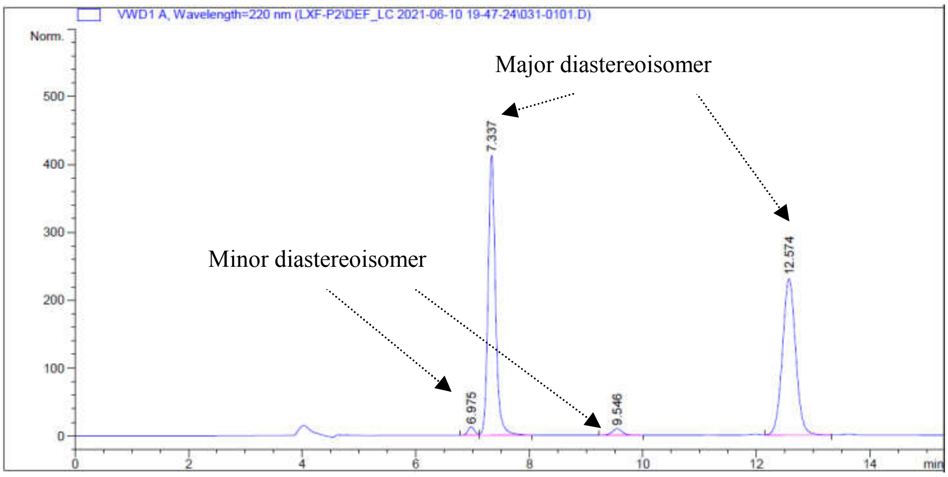

Peak #	RetTime [min]	Type	Width [min]	Area mAU *s	Height [mAU]	Area %
1	6.975	BV	0.1284	104.96179	12.51700	1.3648
2	7.337	VB	0.1376	3730.66675	412.46317	48.5086
3	9.546	BB	0.1866	119.01563	9.61663	1.5475
4	12.574	VB	0.2503	3736.09497	230.42128	48.5791

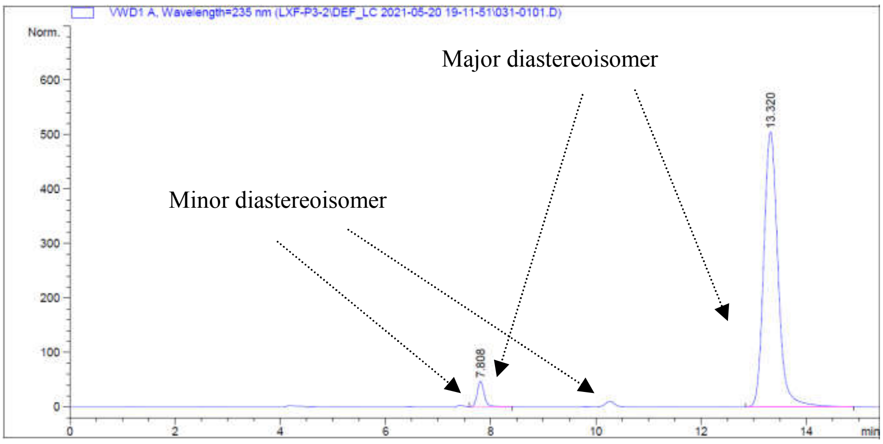

Peak #	RetTime [min]	Type	Width [min]	Area [mAU*s]	Height [mAU]	Area %
1	7.808	VB	0.1437	445.45343	47.17842	4.4871
2	13.320	BB	0.2918	9482.05176	505.28506	95.5129

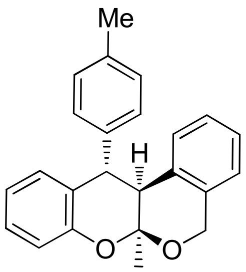

4b

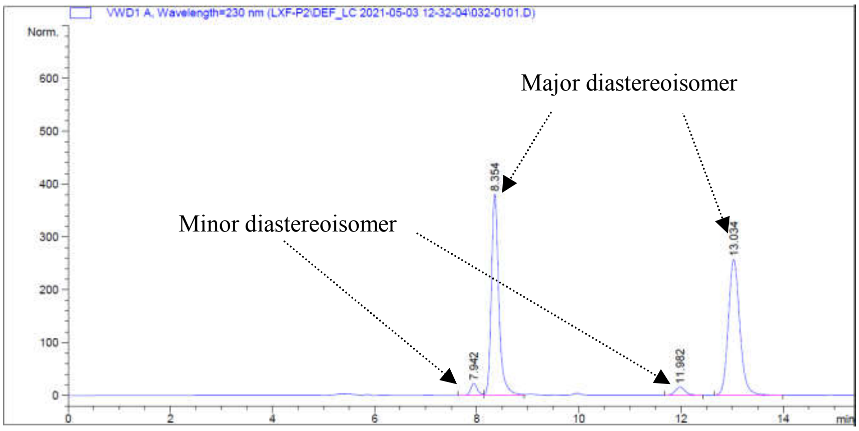

Peak #	RetTime [min]	Type	Width [min]	Area mAU *s	Height [mAU]	Area %
1	7.942	MM	0.1435	191.01791	22.19328	2.2974
2	8.354	MM	0.1711	3914.95190	381.43973	47.0860
3	11.982	MM	0.2229	205.37683	15.35980	2.4701
4	13.034	MM	0.2600	4003.11914	256.65497	48.1464

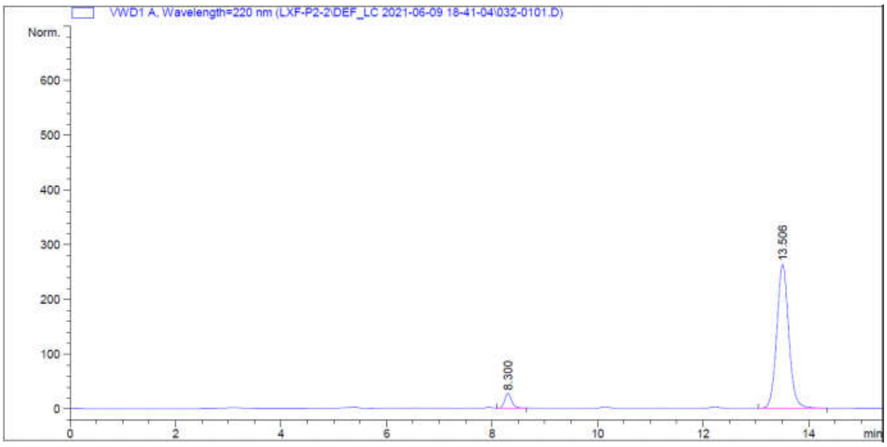

Peak #	RetTime [min]	Type	Width [min]	Area mAU *s	Height [mAU]	Area %
1	8.300	VB	0.1438	255.42485	27.00824	5.8205
2	13.506	BB	0.2404	4132.91406	262.66440	94.1795

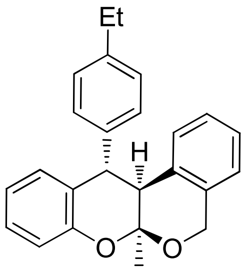

4c

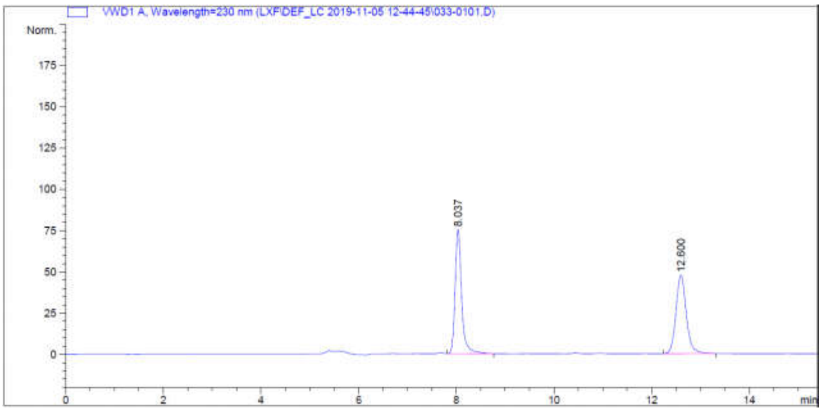

Peak #	RetTime [min]	Type	Width [min]	Area mAU *s	Height [mAU]	Area %
1	8.037	VB	0.1420	711.22052	75.47575	50.4083
2	12.600	BB	0.2253	699.69830	47.62982	49.5917

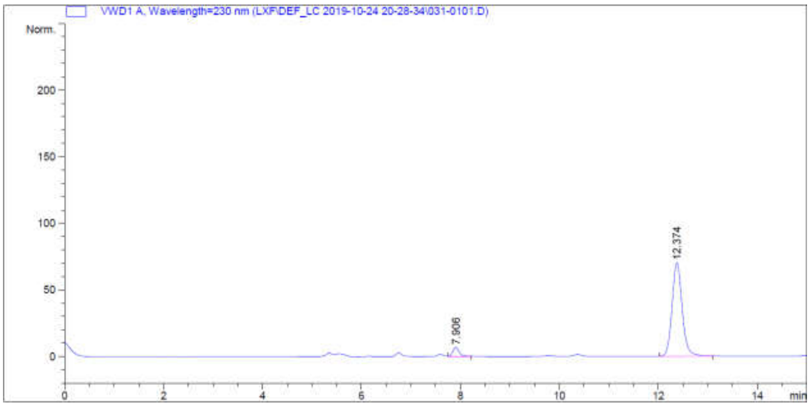

Peak #	RetTime [min]	Type	Width [min]	Area mAU *s	Height [mAU]	Area %
1	7.906	MM	0.1403	55.92403	6.64462	5.2727
2	12.374	MM	0.2387	1004.71588	70.15888	94.7273

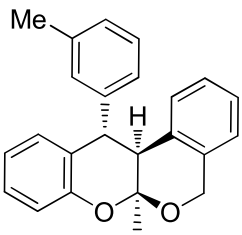

4d

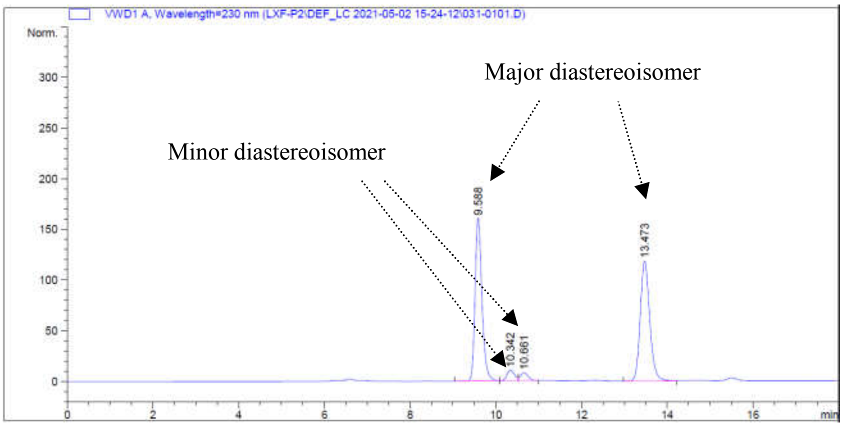

Peak #	RetTime [min]	Type	Width [min]	Area mAU *s	Height [mAU]	Area %
1	9.588	BV	0.1690	1783.85022	160.51265	46.6540
2	10.342	VV	0.1907	129.82603	10.51310	3.3954
3	10.661	VB	0.1833	95.81369	7.84126	2.5059
4	13.473	BB	0.2356	1814.08423	118.38957	47.4447

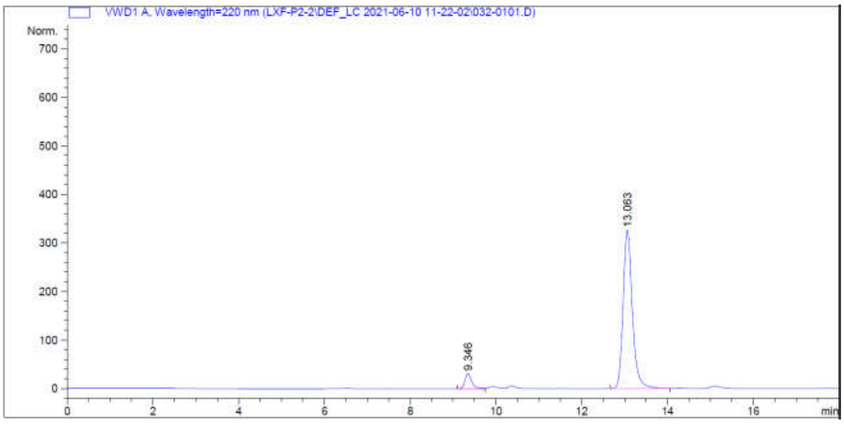

Peak #	RetTime [min]	Type	Width [min]	Area mAU *s	Height [mAU]	Area %
1	9.346	BV	0.1636	338.67657	31.05403	6.5078
2	13.063	BB	0.2263	4865.47266	326.50931	93.4922

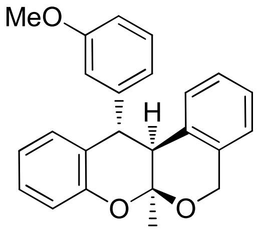

4e

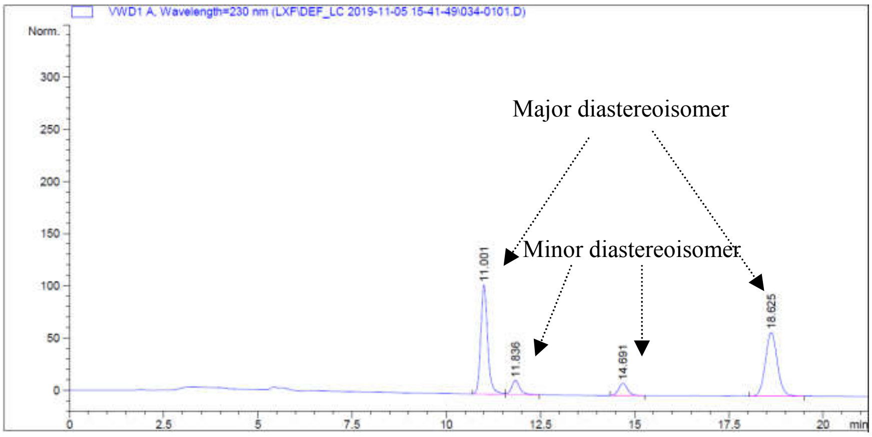

Peak #	RetTime [min]	Type	Width [min]	Area mAU *s	Height [mAU]	Area %
1	11.001	BV	0.1947	1328.09766	104.59319	43.2780
2	11.836	VB	0.2265	200.55299	13.43675	6.5353
3	14.691	BB	0.2539	193.01024	11.68397	6.2895
4	18.625	VB	0.3404	1347.09814	61.05577	43.8972

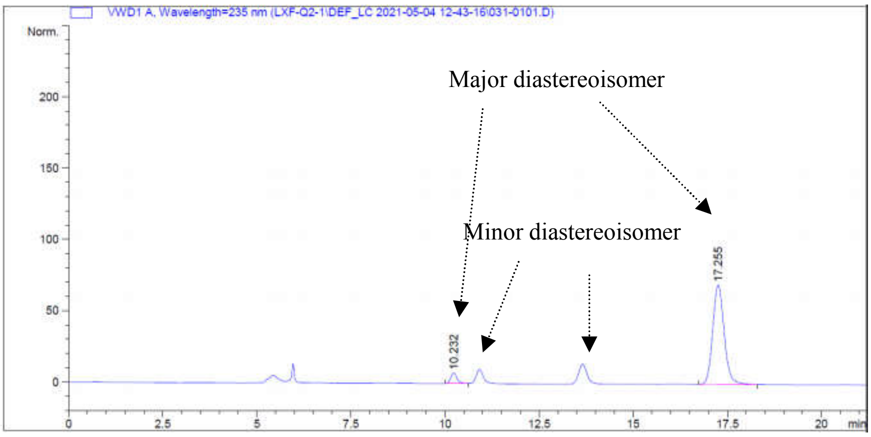

Peak #	RetTime [min]	Type	Width [min]	Area [mAU*s]	Height [mAU]	Area %
1	10.232	BB	0.1781	87.39174	7.42412	5.6000
2	17.255	BB	0.3256	1473.16968	69.62006	94.4000

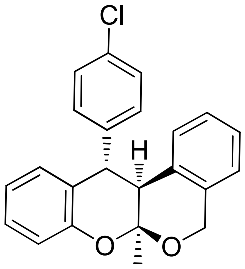

4f

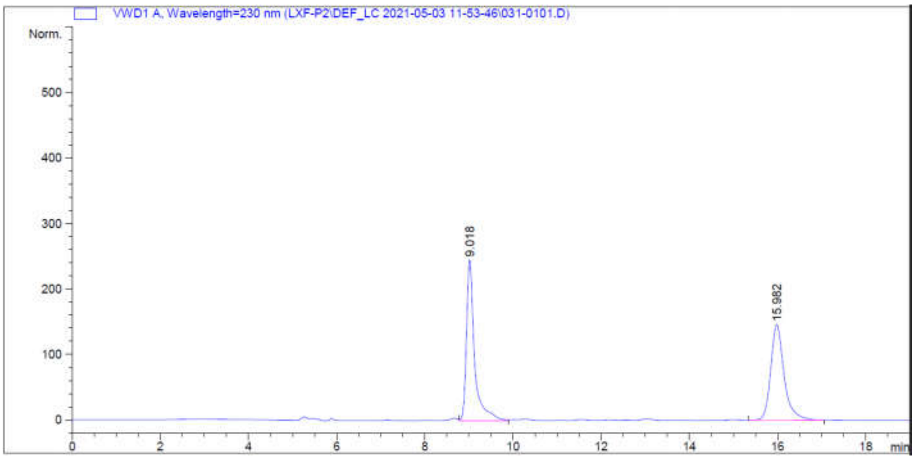

Peak #	RetTime [min]	Type	Width [min]	Area mAU *s	Height [mAU]	Area %
1	9.018	VV	0.1859	3113.78076	245.39366	50.2731
2	15.982	VB	0.3195	3079.94702	145.65443	49.7269

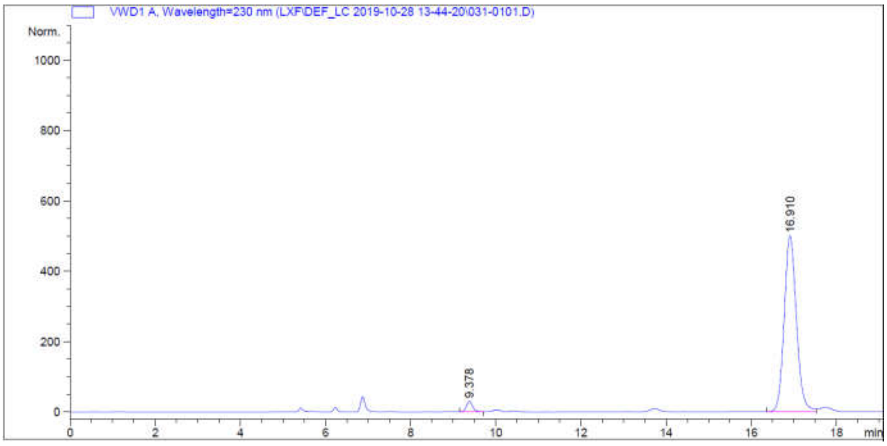

Peak #	RetTime [min]	Type	Width [min]	Area mAU *s	Height [mAU]	Area %
1	9.378	VV	0.1643	324.54688	30.30326	3.1415
2	16.910	VV	0.3089	1.00065e4	500.65811	96.8585

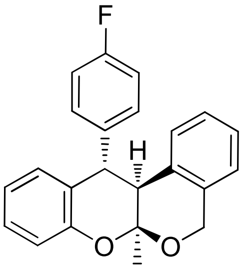

4g

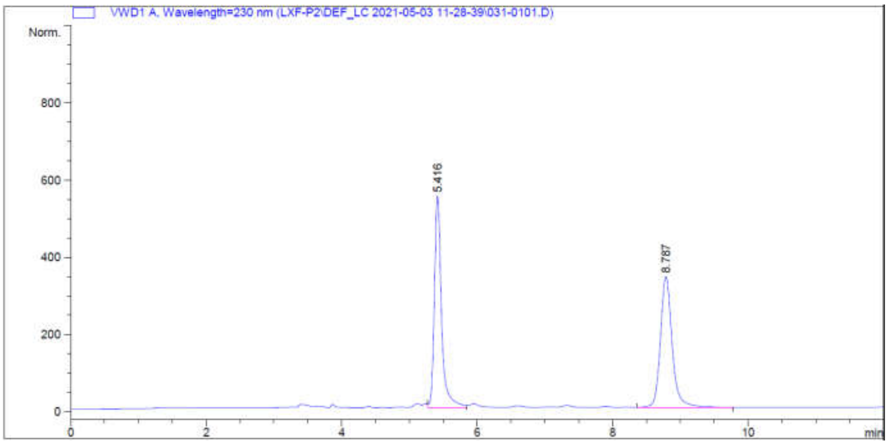

Peak #	RetTime [min]	Type	Width [min]	Area mAU *s	Height [mAU]	Area %
1	5.416	VV	0.1099	4000.84595	548.36096	49.4717
2	8.787	VB	0.1841	4086.30054	339.43478	50.5283

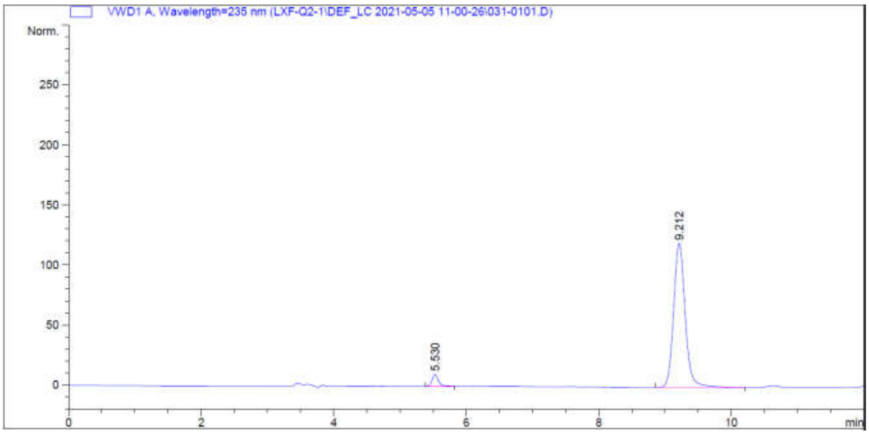

Peak #	RetTime [min]	Type	Width [min]	Area [mAU*s]	Height [mAU]	Area %
1	5.530	BB	0.1019	65.58376	9.74126	4.3076
2	9.212	BB	0.1853	1456.93115	120.02774	95.6924

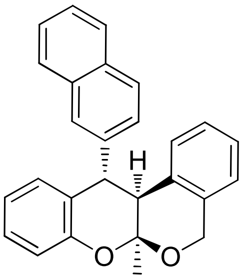

4h

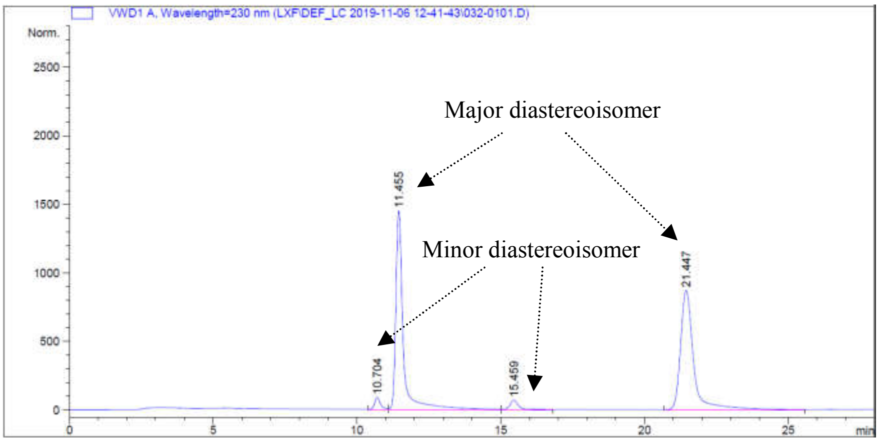

Peak #	RetTime [min]	Type	Width [min]	Area mAU *s	Height [mAU]	Area %
1	10.704	BV	0.2251	1372.41223	90.41931	2.4372
2	11.455	VV	0.2699	2.64970e4	1450.65027	47.0556
3	15.459	VB	0.3191	1472.76257	68.54536	2.6155
4	21.447	MM	0.5162	2.69678e4	870.66559	47.8917

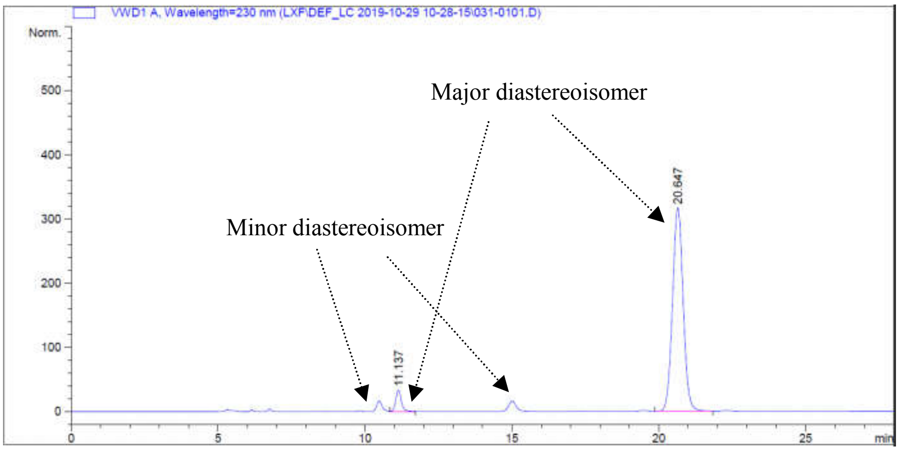

Peak #	RetTime [min]	Type	Width [min]	Area mAU *s	Height [mAU]	Area %
1	11.137	VB	0.1990	434.31207	33.25431	5.1574
2	20.647	VB	0.3901	7986.78369	316.92902	94.8426

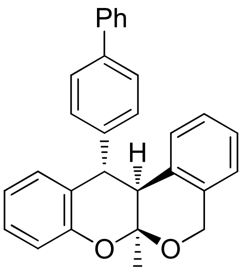

4i

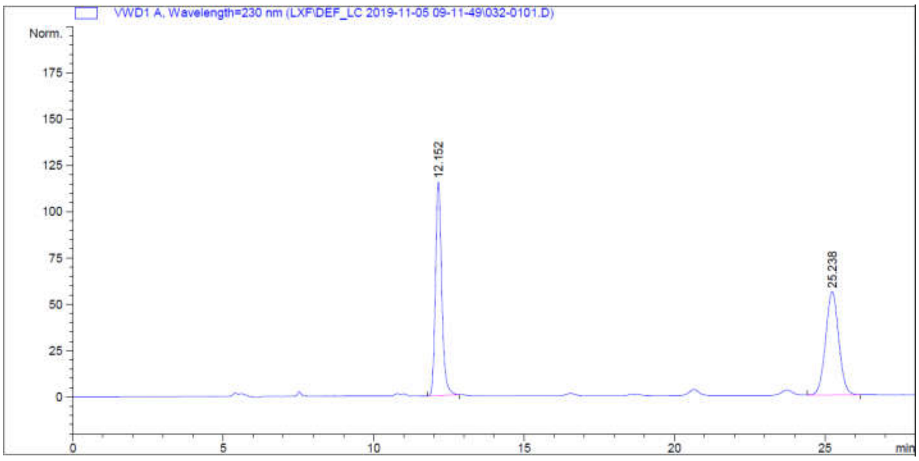

Peak #	RetTime [min]	Type	Width [min]	Area mAU *s	Height [mAU]	Area %
1	12.153	BB	0.2227	1685.92871	115.53764	49.7711
2	25.238	VB	0.4720	1701.43640	55.83889	50.2289

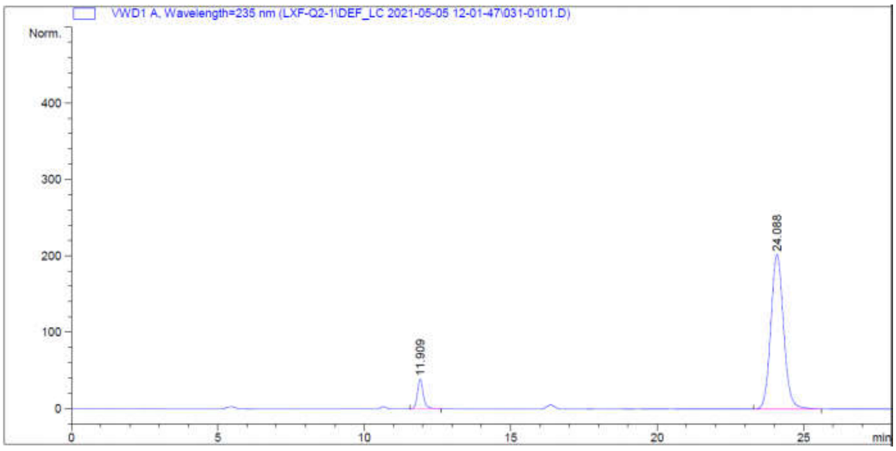

Peak #	RetTime [min]	Type	Width [min]	Area [mAU*s]	Height [mAU]	Area %
1	11.909	BB	0.2119	537.57935	38.63700	8.0124
2	24.088	BB	0.4689	6171.78223	202.72780	91.9876

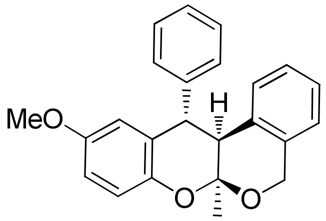

4j

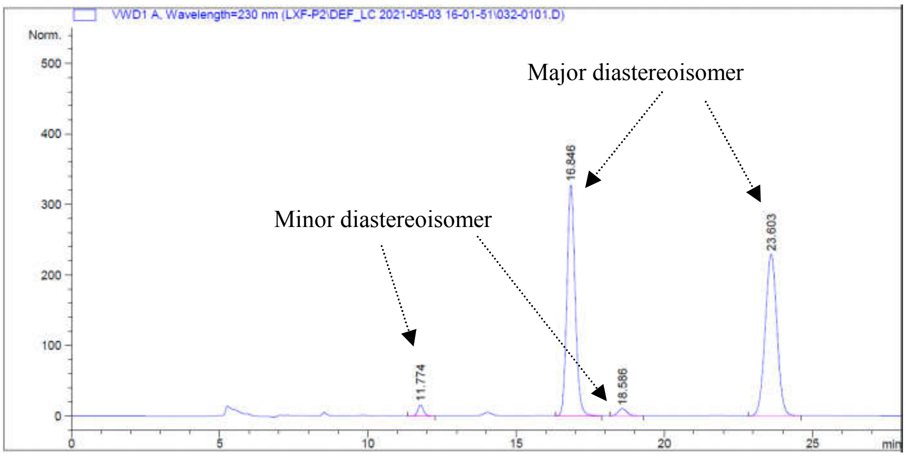

Peak #	RetTime [min]	Type	Width [min]	Area mAU *s	Height [mAU]	Area %
1	11.774	VB	0.2004	206.08344	15.77620	1.5815
2	16.846	BB	0.2970	6289.85449	327.25217	48.2677
3	18.586	BB	0.3226	215.15778	10.22571	1.6511
4	23.603	BB	0.4290	6320.08740	229.51579	48.4997

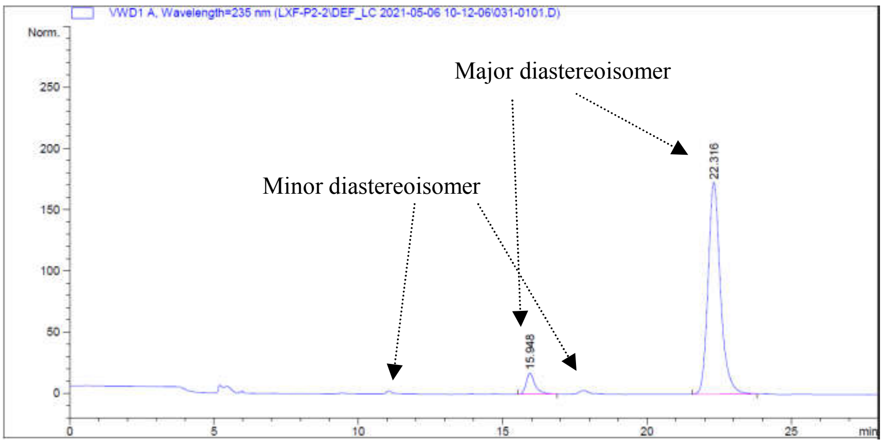

Peak #	RetTime [min]	Type	Width [min]	Area [mAU*s]	Height [mAU]	Area %
1	15.948	BB	0.3283	376.24640	17.18903	6.8041
2	22.316	BB	0.4523	5153.49414	173.09384	93.1959

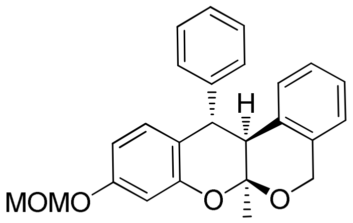

4k

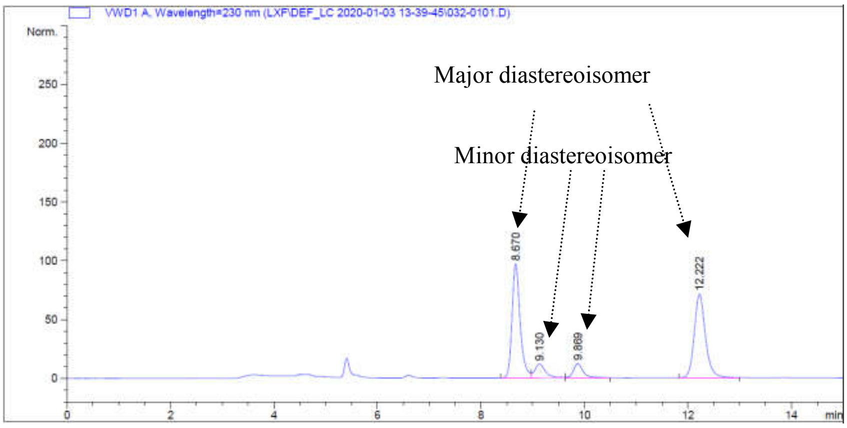

Peak #	RetTime [min]	Type	Width [min]	Area mAU *s	Height [mAU]	Area %
1	8.670	BV	0.1628	1049.25562	96.80769	43.0789
2	9.130	VV	0.2095	167.45633	11.77915	6.8752
3	9.869	VB	0.1931	155.63423	12.03253	6.3898
4	12.222	BB	0.2284	1063.31152	71.08721	43.6560

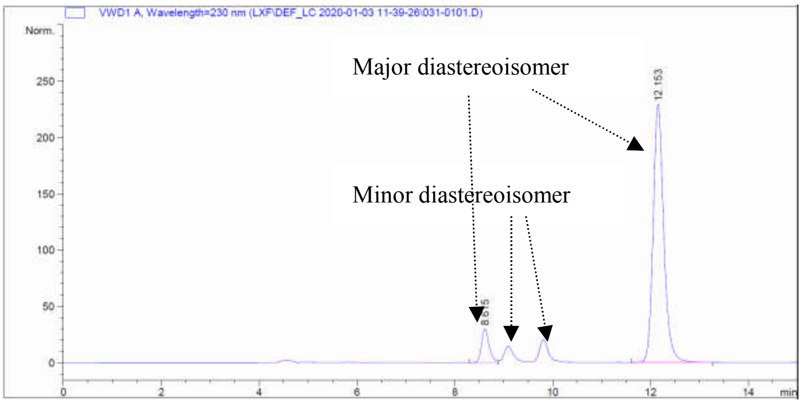

Peak #	RetTime [min]	Type	Width [min]	Area mAU *s	Height [mAU]	Area %
1	8.615	VV	0.1641	323.38467	29.89355	8.5691
2	12.153	VB	0.2298	3450.45361	228.82332	91.4309

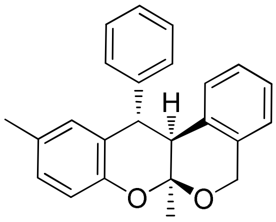

4l

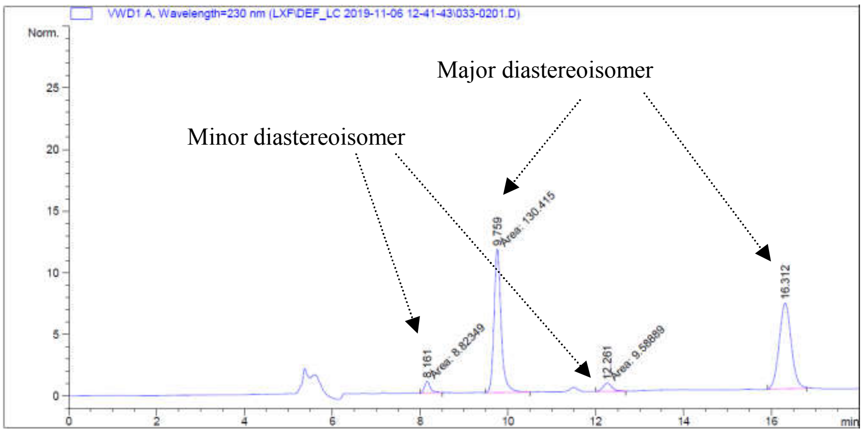

Peak #	RetTime [min]	Type	Width [min]	Area mAU *s	Height [mAU]	Area %
1	8.161	MM	0.1544	8.82349	9.52171e-1	3.1406
2	9.759	MM	0.1868	130.41501	11.63821	46.4187
3	12.261	MM	0.2394	9.58889	6.67661e-1	3.4130
4	16.312	BB	0.2943	132.12628	6.95829	47.0278

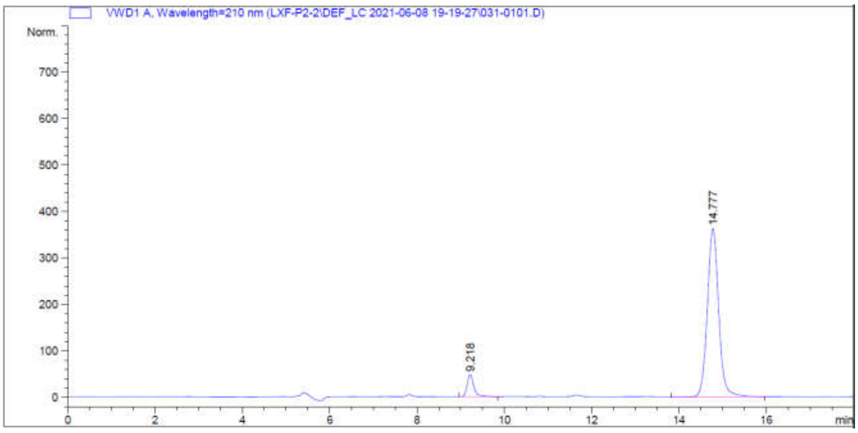

Peak #	RetTime [min]	Type	Width [min]	Area [mAU*s]	Height [mAU]	Area %
1	9.218	MM	0.1443	369.61432	42.70393	5.3284
2	14.777	BB	0.2784	6567.06104	362.58774	94.6716

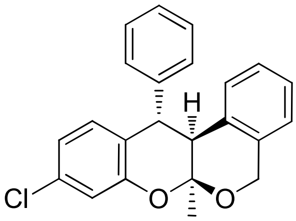

4m

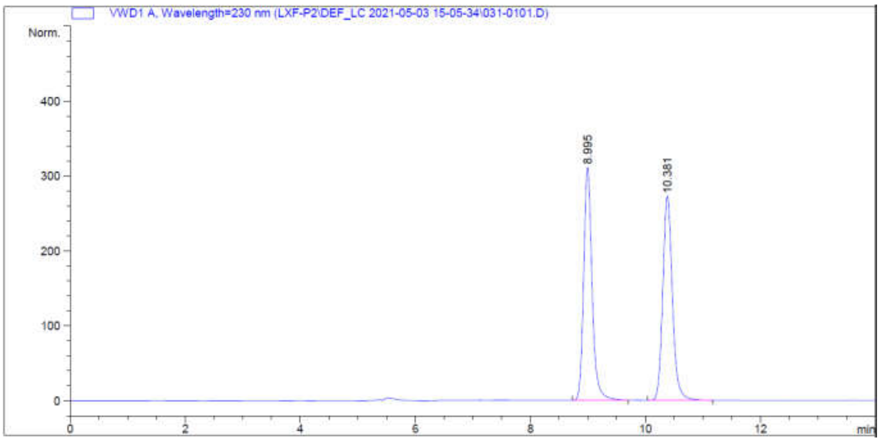

Peak #	RetTime [min]	Type	Width [min]	Area mAU *s	Height [mAU]	Area %
1	8.995	BB	0.1551	3163.10986	310.83575	50.0032
2	10.381	VB	0.1776	3162.70557	272.47183	49.9968

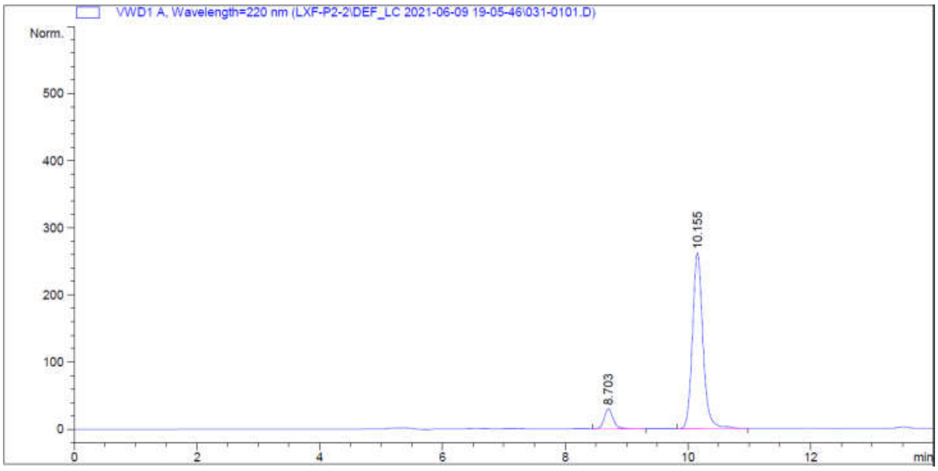

Peak #	RetTime [min]	Type	Width [min]	Area mAU *s	Height [mAU]	Area %
1	8.703	VB	0.1555	306.65982	30.03414	8.9347
2	10.155	BB	0.1815	3125.56689	261.75769	91.0653

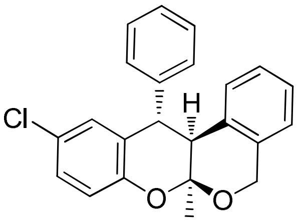

4n

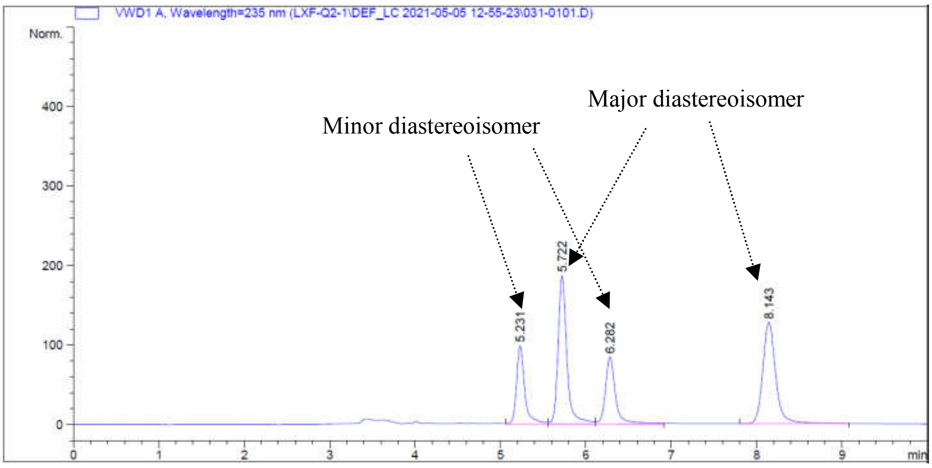

Peak #	RetTime [min]	Type	Width [min]	Area [mAU*s]	Height [mAU]	Area %
1	5.231	VV	0.1002	662.43481	98.62775	16.3936
2	5.722	VV	0.1072	1342.40088	186.62717	33.2211
3	6.282	VV	0.1240	695.88416	84.25954	17.2214
4	8.143	VB	0.1604	1340.08887	127.58478	33.1639

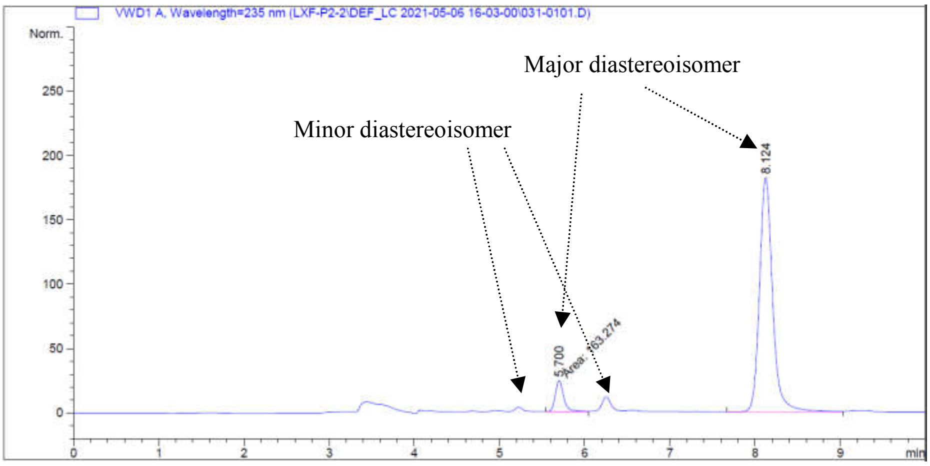

Peak #	RetTime [min]	Type	Width [min]	Area mAU *s	Height [mAU]	Area %
1	5.662	MM	0.1113	184.44144	27.61401	7.7839
2	8.054	VB	0.1578	2185.08057	212.45975	92.2161

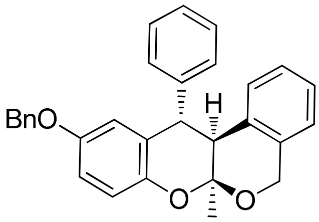

4o

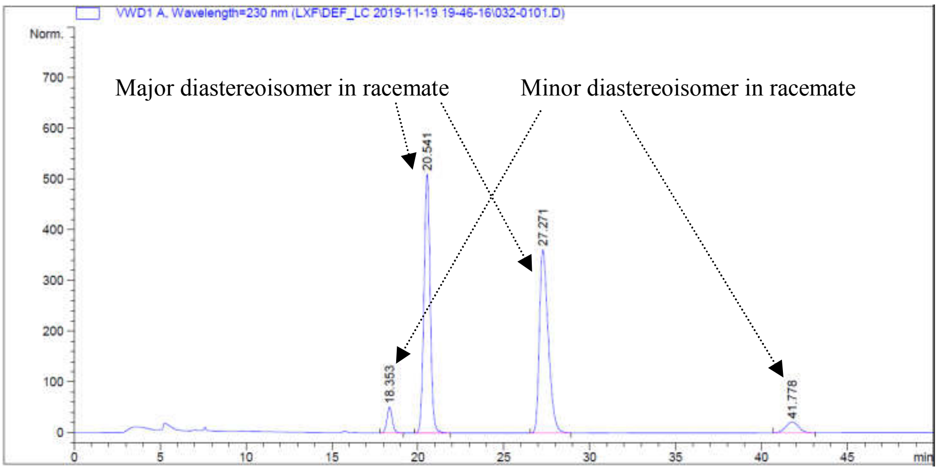

Peak #	RetTime [min]	Type	Width [min]	Area mAU *s	Height [mAU]	Area %
1	18.354	BB	0.3325	1096.84680	50.98329	3.9281
2	20.541	BB	0.3889	1.28066e4	510.35083	45.8645
3	27.272	BB	0.5426	1.29305e4	361.44617	46.3082
4	41.778	BB	0.7905	1088.75134	21.12609	3.8992

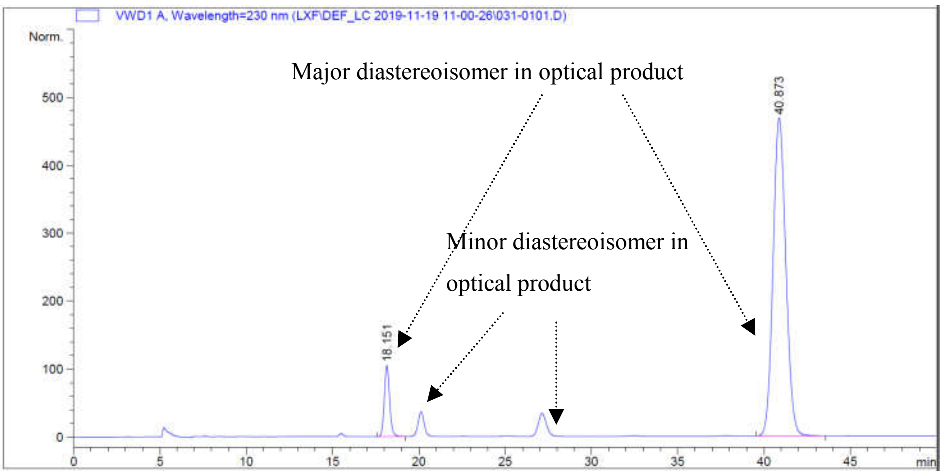

Peak #	RetTime [min]	Type	Width [min]	Area mAU *s	Height [mAU]	Area %
1	18.151	BB	0.3303	2235.13330	104.21784	8.4950
2	40.873	BB	0.8009	2.40760e4	468.17508	91.5050

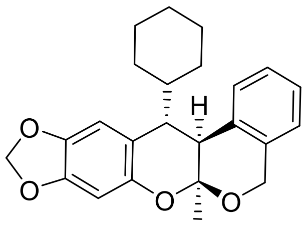

4p

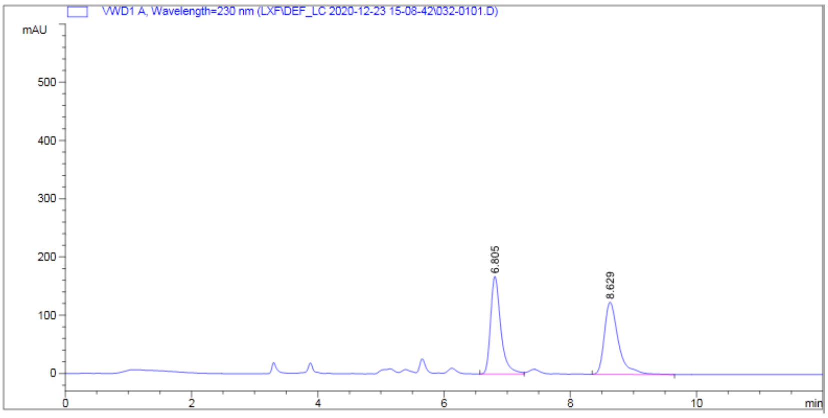

Peak #	RetTime [min]	Type	Width [min]	Area mAU *s	Height [mAU]	Area %
1	6.805	VV	0.1694	1892.17090	167.83118	49.8327
2	8.629	BB	0.2319	1904.87231	123.81264	50.1673

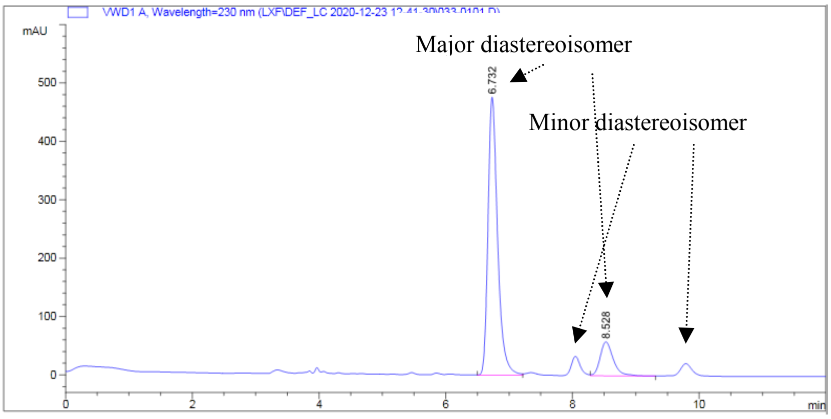

Peak #	RetTime [min]	Type	Width [min]	Area mAU *s	Height [mAU]	Area %
1	6.732	VV	0.1591	5008.06055	476.04922	85.7246
2	8.528	VB	0.2184	833.97430	58.10251	14.2754

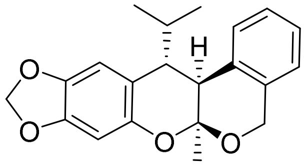

4q

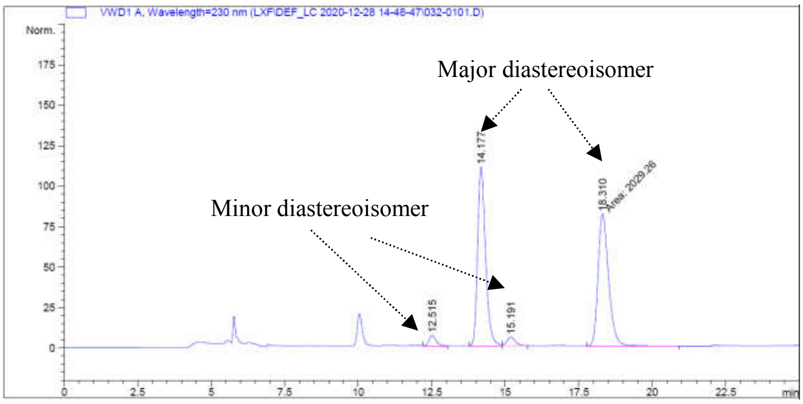

Peak #	RetTime [min]	Type	Width [min]	Area mAU *s	Height [mAU]	Area %
1	12.515	VB	0.2488	105.94632	6.48487	2.4596
2	14.177	BV	0.2836	2064.84106	110.48618	47.9366
3	15.191	VB	0.3115	107.38678	5.24791	2.4931
4	18.310	MM	0.4152	2029.26379	81.46393	47.1107

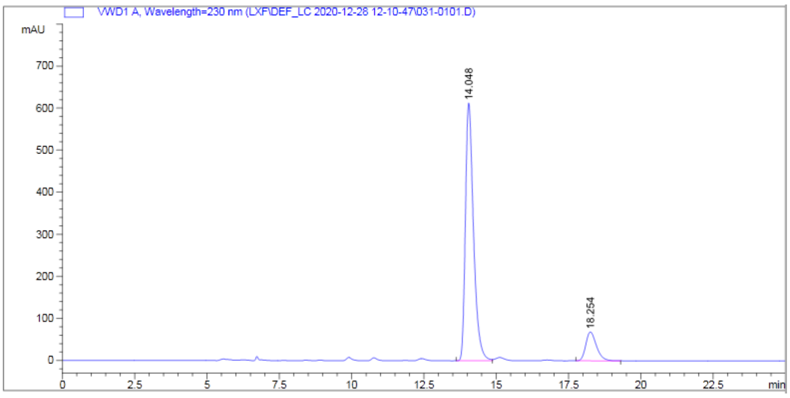

Peak #	RetTime [min]	Type	Width [min]	Area mAU *s	Height [mAU]	Area %
1	14.048	BV	0.2969	1.19306e4	613.12134	87.1463
2	18.254	BB	0.3928	1759.71924	68.51810	12.8537

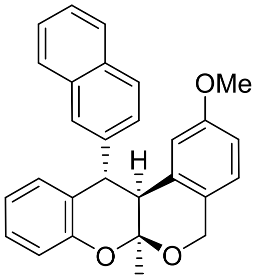

4r

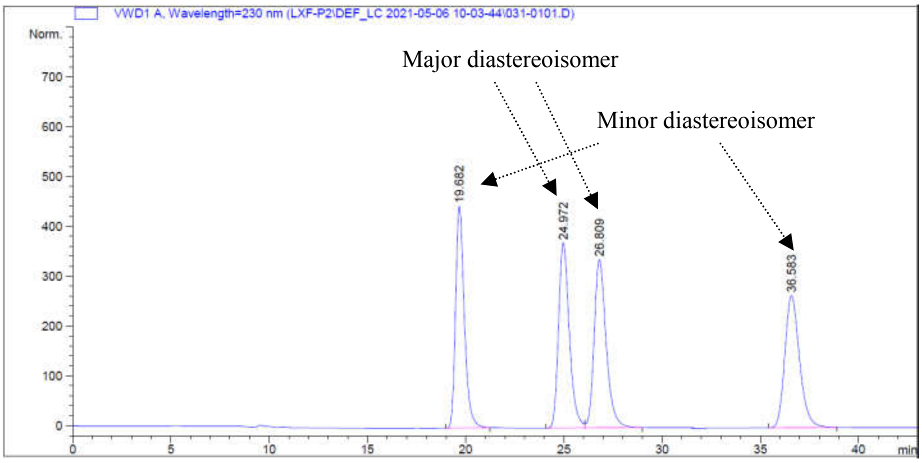

Peak #	RetTime [min]	Type	Width [min]	Area mAU *s	Height [mAU]	Area %
1	19.682	BB	0.4804	1.37697e4	444.97046	24.5313
2	24.972	BV	0.5819	1.41831e4	371.83600	25.2677
3	26.809	VB	0.6419	1.43660e4	337.72906	25.5936
4	36.583	BB	0.8209	1.38125e4	264.88657	24.6075

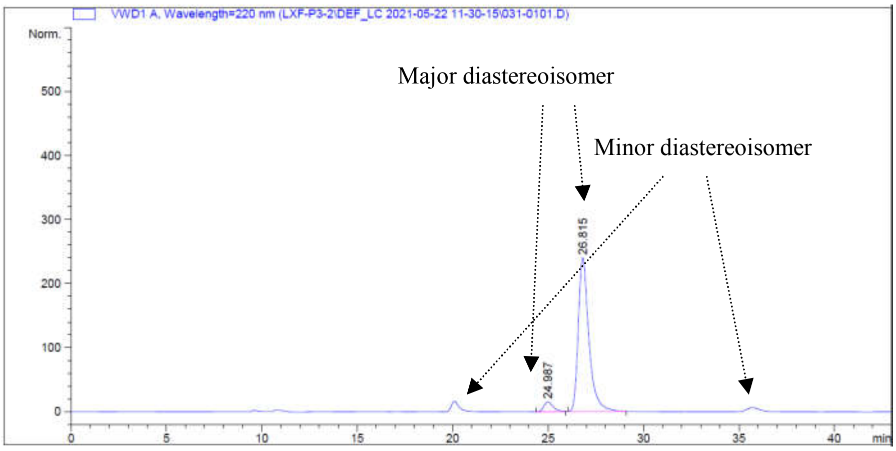

Peak #	RetTime [min]	Type	Width [min]	Area [mAU*s]	Height [mAU]	Area %
1	24.987	BB	0.5207	521.11969	15.37711	5.3107
2	26.815	BB	0.5856	9291.49121	240.09822	94.6893

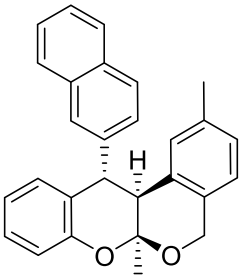

4s

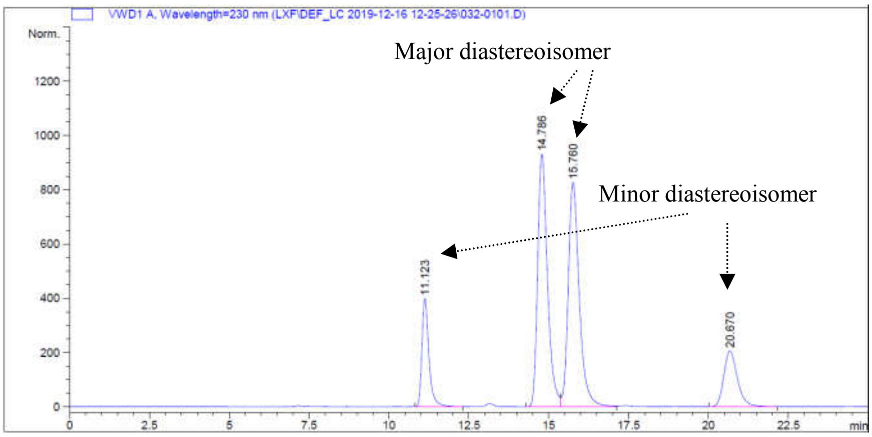

Peak #	RetTime [min]	Type	Width [min]	Area mAU *s	Height [mAU]	Area %
1	11.123	VB	0.2302	6078.06934	398.88419	11.7333
2	14.786	BV	0.3270	1.98275e4	931.22632	38.2759
3	15.760	VV	0.3629	1.98466e4	827.14270	38.3126
4	20.670	BB	0.4481	6049.47705	205.61946	11.6781

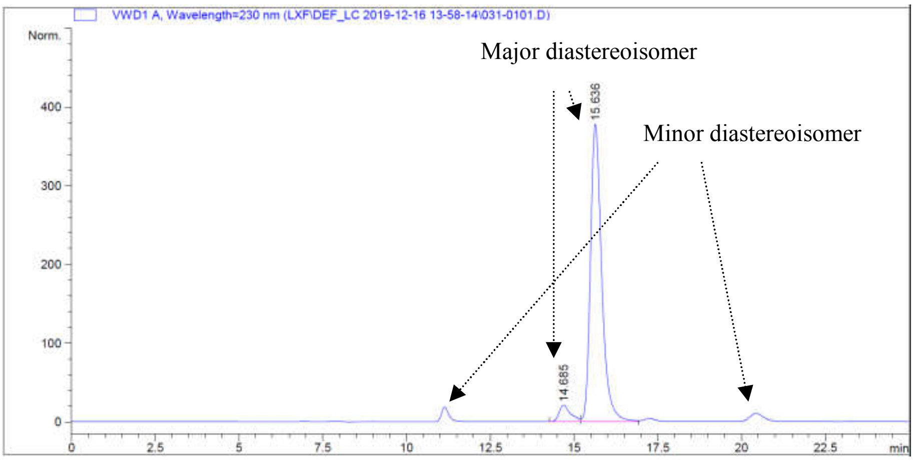

Peak #	RetTime [min]	Type	Width [min]	Area mAU *s	Height [mAU]	Area %
1	14.685	BV	0.3464	498.26010	21.02593	5.3693
2	15.636	VV	0.3526	8781.49121	377.96524	94.6307

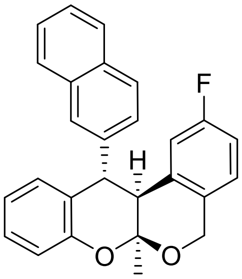

4t

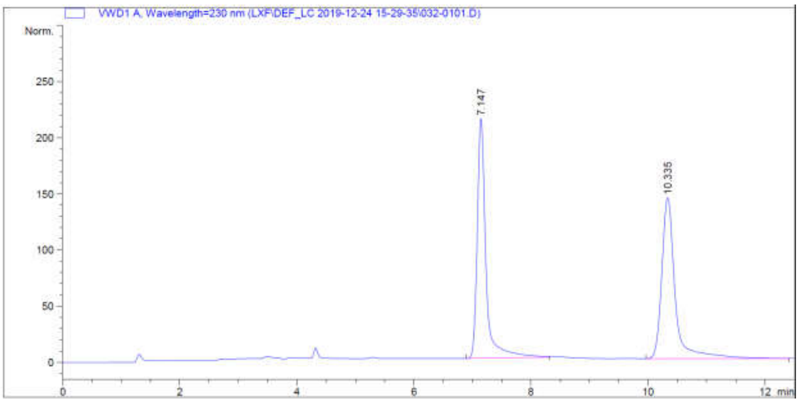

Peak #	RetTime [min]	Type	Width [min]	Area mAU *s	Height [mAU]	Area %
1	7.147	BB	0.1536	2195.07275	213.17220	49.1005
2	10.335	BB	0.2363	2275.49707	143.27444	50.8995

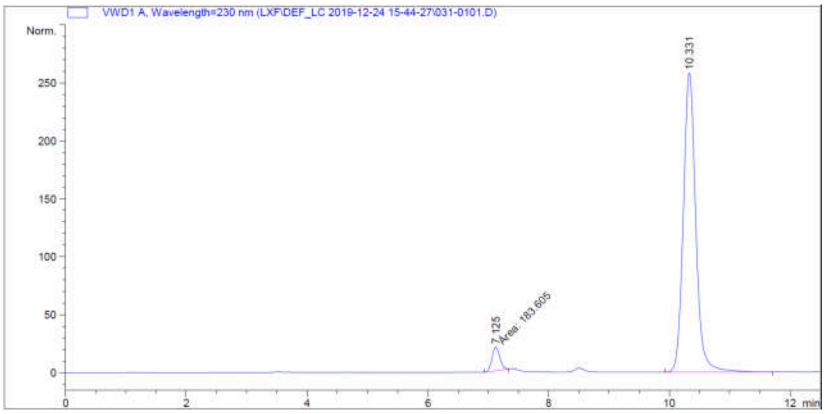

Peak #	RetTime [min]	Type	Width [min]	Area mAU *s	Height [mAU]	Area %
1	7.125	MM	0.1486	183.60461	20.59922	4.7454
2	10.331	BB	0.2192	3685.53296	257.74890	95.2546

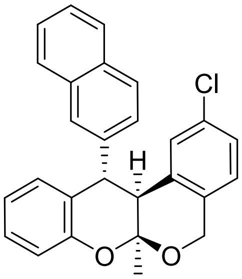

4u

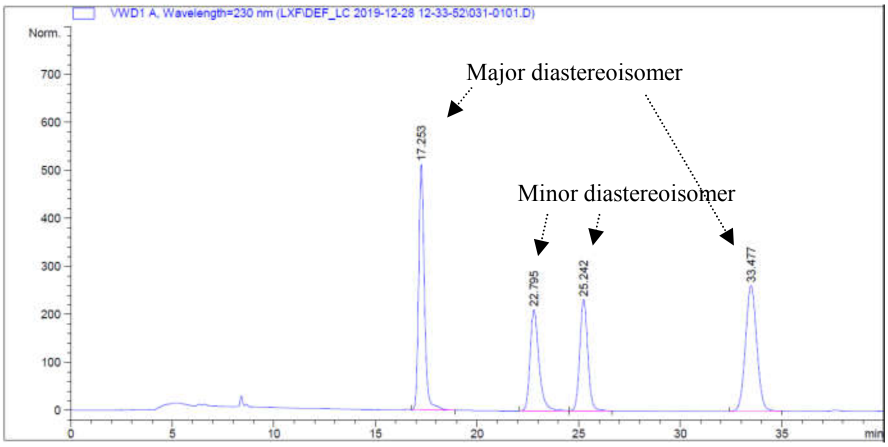

Peak #	RetTime [min]	Type	Width [min]	Area mAU *s	Height [mAU]	Area %
1	17.253	VB	0.2995	1.00715e4	511.69238	30.6571
2	22.795	BB	0.4582	6367.73730	210.24443	19.3830
3	25.242	BB	0.4211	6345.67480	231.92856	19.3159
4	33.477	BB	0.6028	1.00672e4	261.03922	30.6440

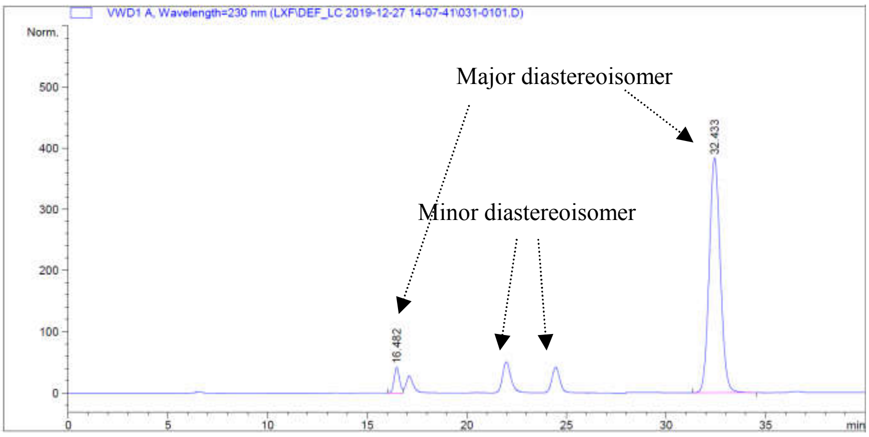

Peak #	RetTime [min]	Type	Width [min]	Area mAU *s	Height [mAU]	Area %
1	16.482	BV	0.2868	780.29205	41.96260	5.0135
2	32.433	BB	0.5972	1.47835e4	383.12686	94.9865

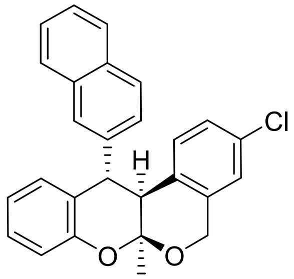

4v

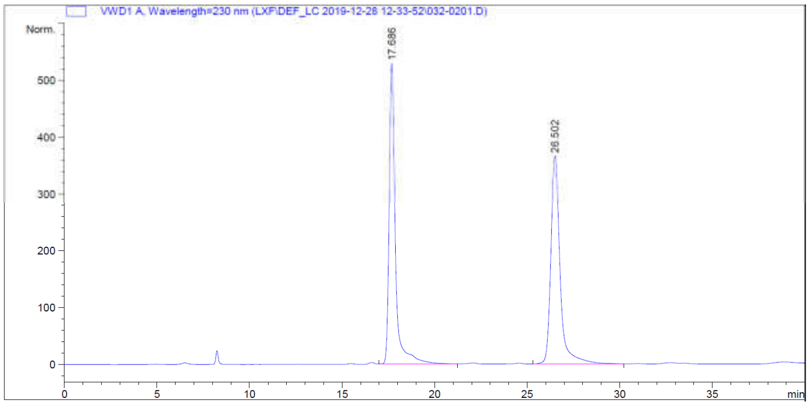

Peak #	RetTime [min]	Type	Width [min]	Area mAU *s	Height [mAU]	Area %
1	17.686	VB	0.3508	1.24659e4	528.70471	49.3982
2	26.502	BB	0.5231	1.27696e4	366.36612	50.6018

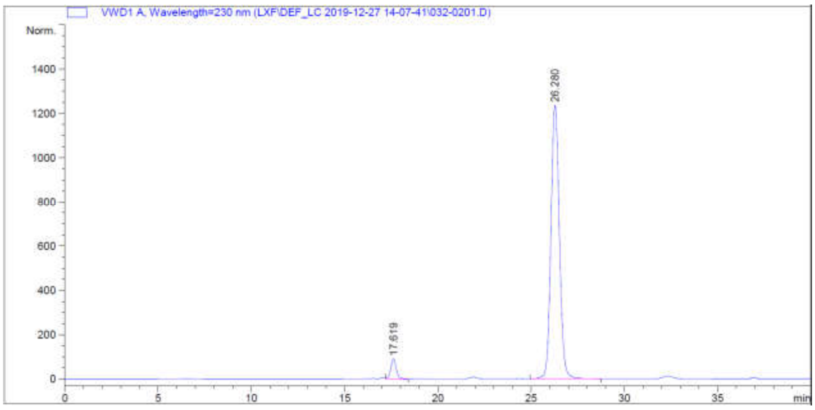

Peak #	RetTime [min]	Type	Width [min]	Area mAU *s	Height [mAU]	Area %
1	17.619	VV	0.3132	1865.25183	90.51764	4.5712
2	26.280	VB	0.4912	3.89392e4	1236.24414	95.4288

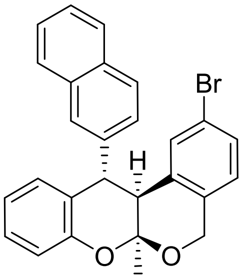

4w

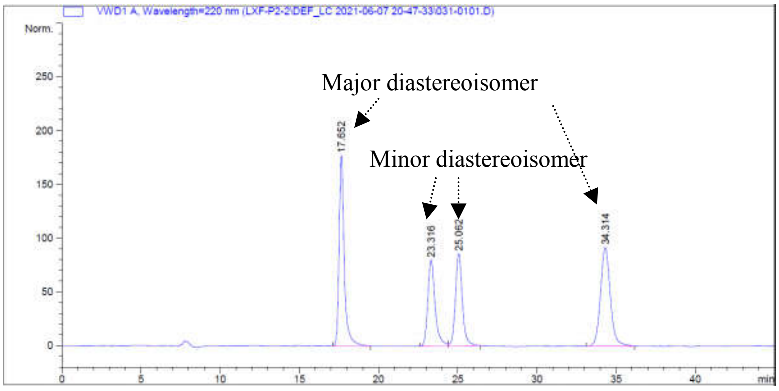

Peak #	RetTime [min]	Type	Width [min]	Area [mAU*s]	Height [mAU]	Area %
1	17.652	BB	0.3377	4013.16675	176.77943	30.5653
2	23.316	BV	0.4787	2557.15894	80.12579	19.4760
3	25.062	VB	0.4574	2589.67163	85.71968	19.7236
4	34.314	BB	0.6506	3969.81104	91.73879	30.2351

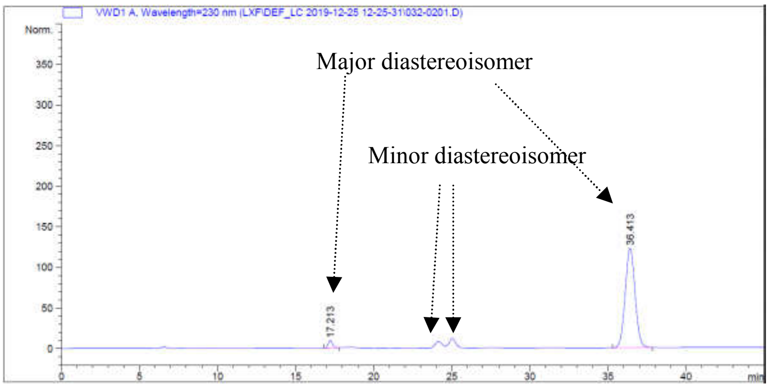

Peak #	RetTime [min]	Type	Width [min]	Area mAU *s	Height [mAU]	Area %
1	17.213	BB	0.2996	178.20328	9.16667	3.2245
2	36.413	BB	0.6835	5348.39355	122.30095	96.7755

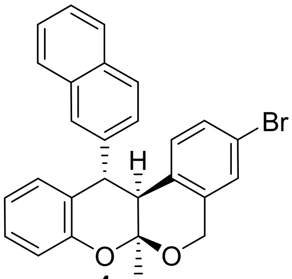

4x

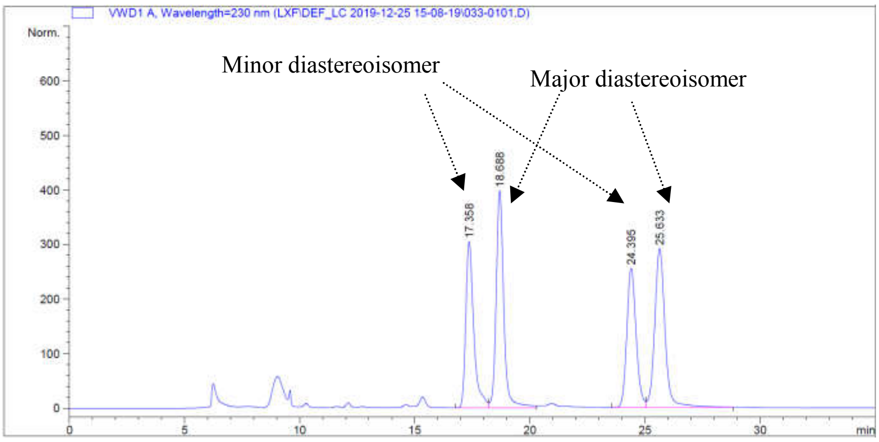

Peak #	RetTime [min]	Type	Width [min]	Area mAU *s	Height [mAU]	Area %
1	17.358	VV	0.3643	7351.30518	303.32181	22.5304
2	18.688	VV	0.3474	9062.12402	397.61047	27.7737
3	24.395	VV	0.4322	7148.37451	254.74095	21.9084
4	25.633	VB	0.4747	9066.63672	290.53235	27.7875

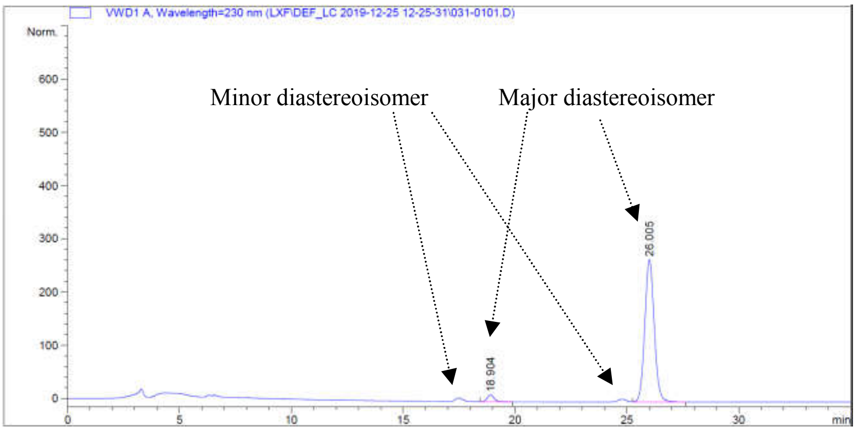

Peak #	RetTime [min]	Type	Width [min]	Area mAU *s	Height [mAU]	Area %
1	18.904	BB	0.3298	269.24561	12.57668	3.3171
2	26.005	VB	0.4521	7847.62451	267.03418	96.6829

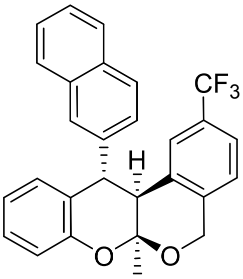

4y

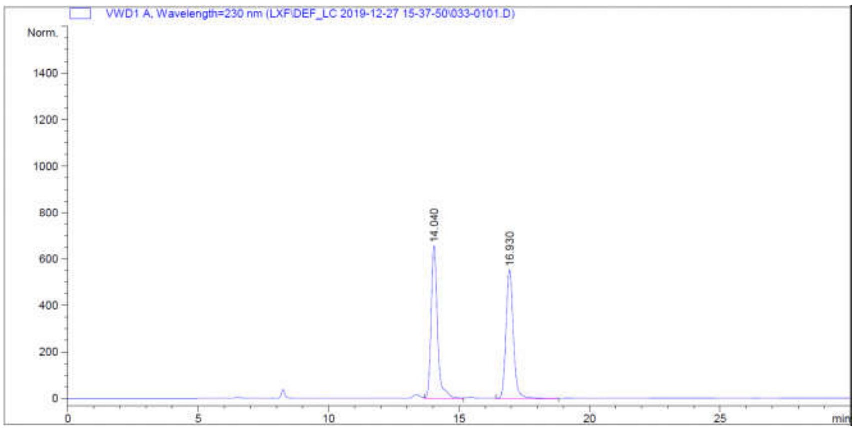

Peak #	RetTime [min]	Type	Width [min]	Area mAU *s	Height [mAU]	Area %
1	14.040	VV	0.2578	1.11442e4	656.16199	50.9036
2	16.930	BB	0.2995	1.07486e4	553.17395	49.0964

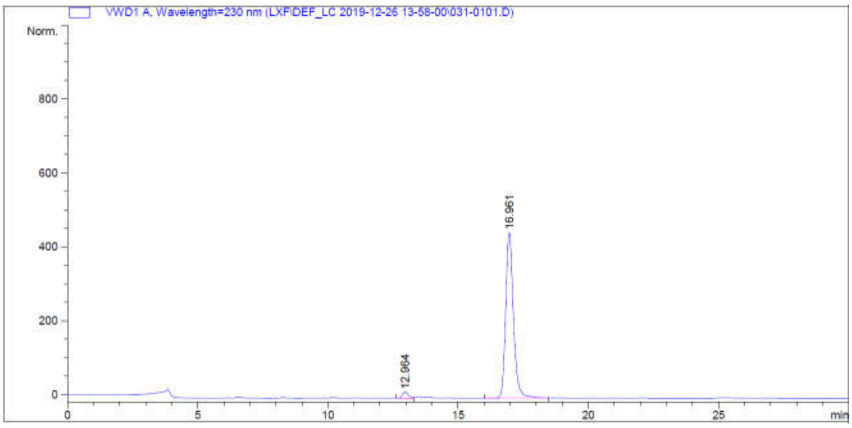

Peak #	RetTime [min]	Type	Width [min]	Area mAU *s	Height [mAU]	Area %
1	12.964	VV	0.2359	262.90540	16.84879	2.7707
2	16.961	VB	0.3130	9225.92188	448.01700	97.2293

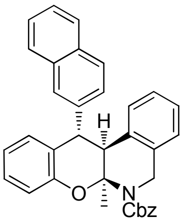

5a

(Only one diastereoisomer was obtained in racemate)

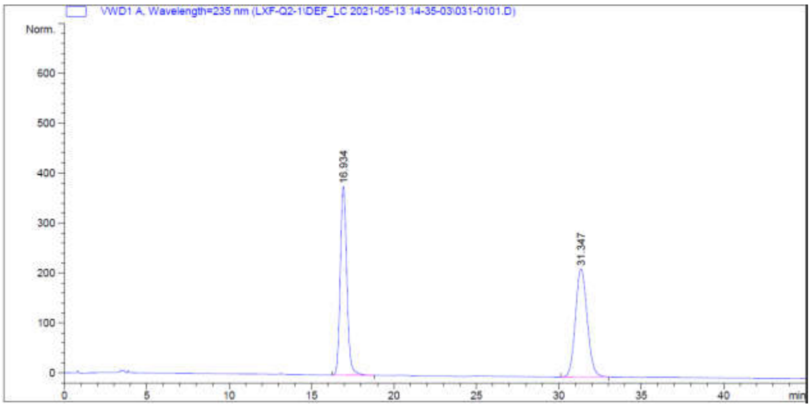

Peak #	RetTime [min]	Type	Width [min]	Area [mAU*s]	Height [mAU]	Area %
1	16.934	BB	0.4270	1.03843e4	377.88153	49.1951
2	31.347	BB	0.7718	1.07241e4	216.49045	50.8049

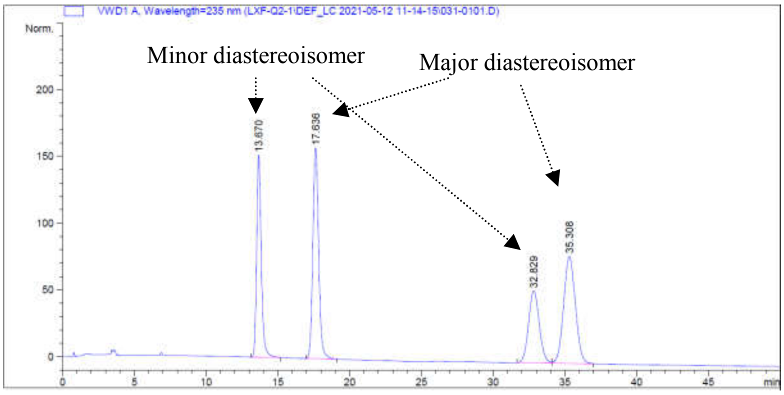

Peak #	RetTime [min]	Type	Width [min]	Area [mAU*s]	Height [mAU]	Area %
1	13.670	BB	0.3389	3375.78149	151.37819	22.3705
2	17.636	BB	0.4307	4384.54688	157.73486	29.0554
3	32.829	BV	0.8008	2791.85449	53.92163	18.5010
4	35.308	VB	0.8979	4538.12305	80.18983	30.0731

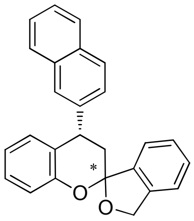

5b

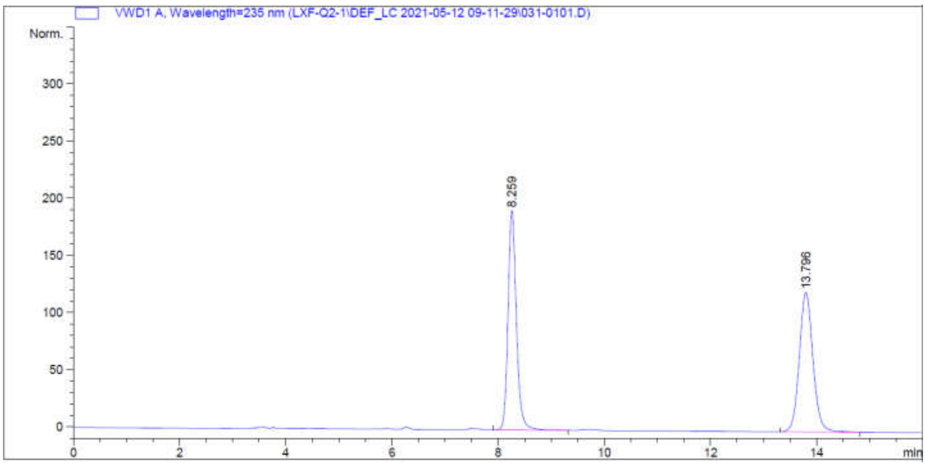

Peak #	RetTime [min]	Type	Width [min]	Area [mAU*s]	Height [mAU]	Area %
1	8.259	BB	0.1676	2110.54492	192.02049	49.1842
2	13.796	BB	0.2742	2180.55713	121.95117	50.8158

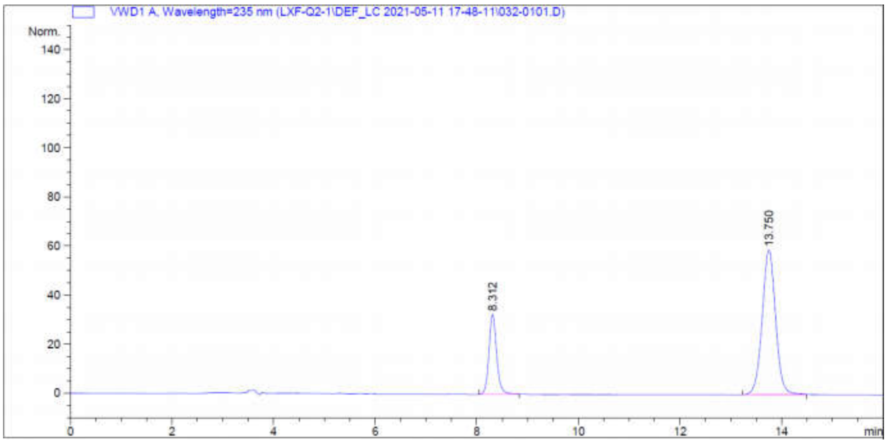

Peak #	RetTime [min]	Type	Width [min]	Area [mAU*s]	Height [mAU]	Area %
1	8.312	BB	0.1614	343.91788	32.47711	24.1259
2	13.750	BB	0.2825	1081.59619	58.97790	75.8741

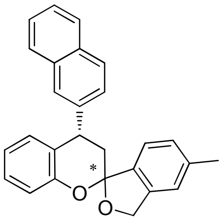

5c

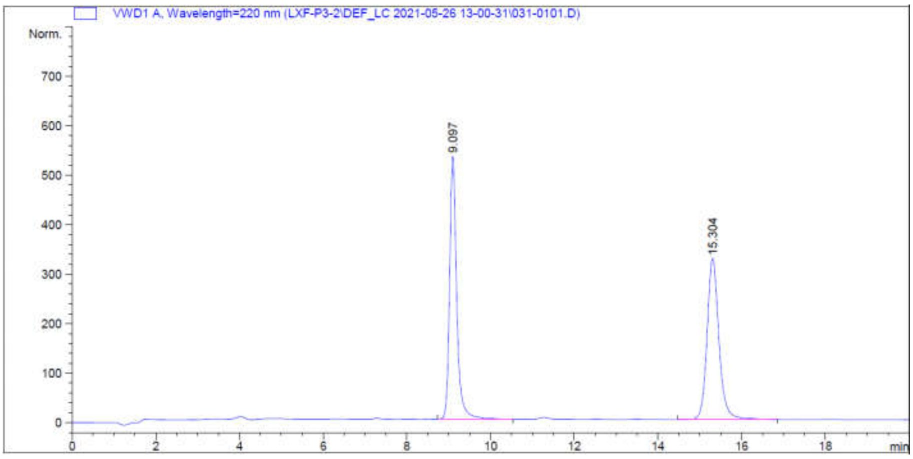

Peak #	RetTime [min]	Type	Width [min]	Area [mAU*s]	Height [mAU]	Area %
1	9.097	VB	0.1772	6282.65479	531.72754	49.4899
2	15.304	VB	0.2997	6412.16504	325.69818	50.5101

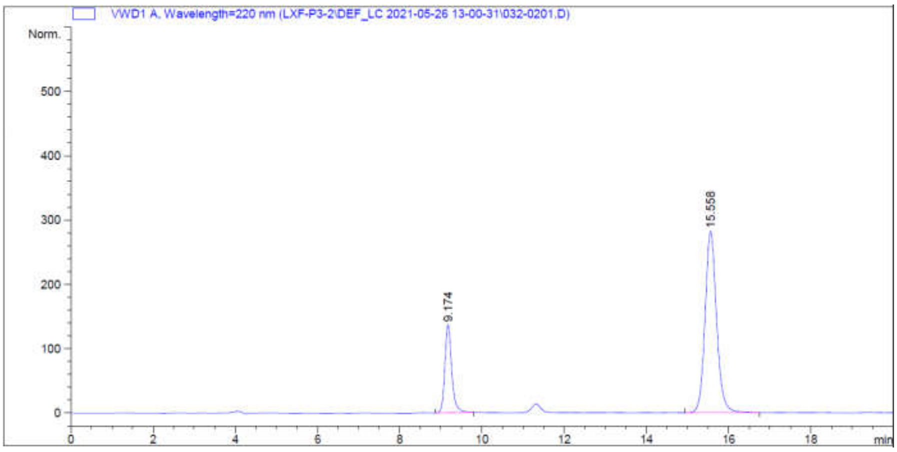

Peak #	RetTime [min]	Type	Width [min]	Area [mAU*s]	Height [mAU]	Area %
1	9.174	VB	0.1739	1577.66907	136.78520	22.3130
2	15.558	VB	0.2971	5492.95117	282.15552	77.6870

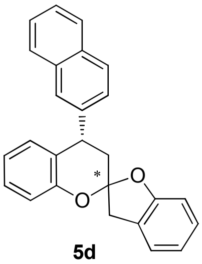

5d

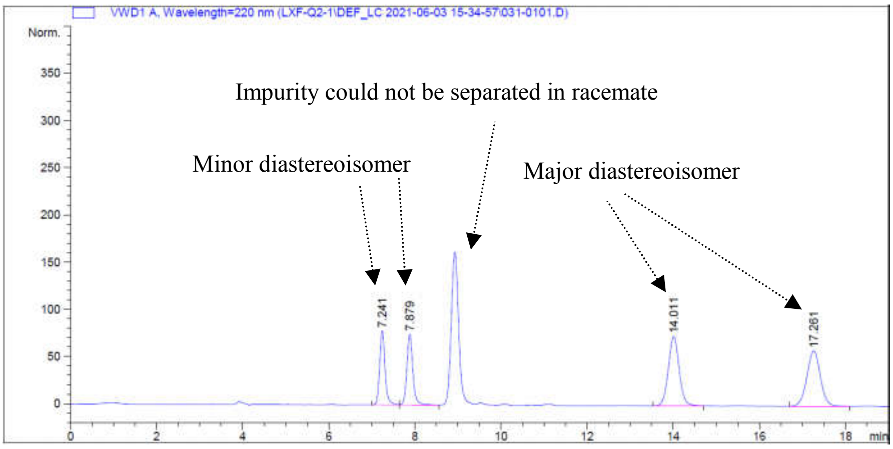

Peak #	RetTime [min]	Type	Width [min]	Area [mAU*s]	Height [mAU]	Area %
1	7.241	BV	0.1321	684.15692	78.64342	17.2632
2	7.879	VB	0.1476	723.68616	74.97568	18.2606
3	14.011	BB	0.2693	1283.20984	73.51001	32.3790
4	17.261	BB	0.3346	1272.04260	58.67724	32.0972

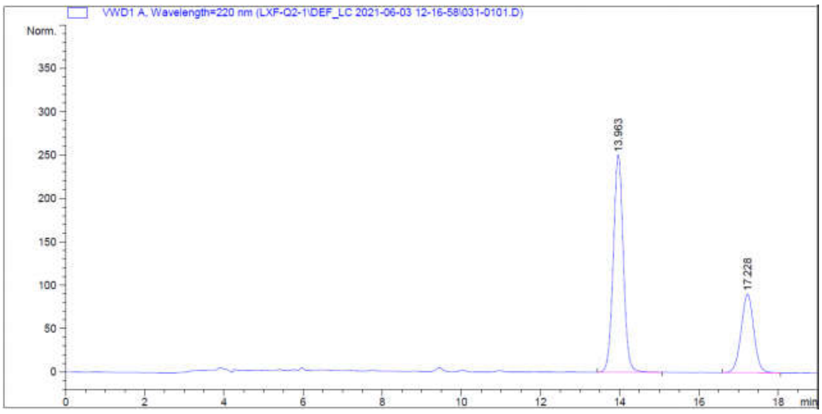

Peak #	RetTime [min]	Type	Width [min]	Area [mAU*s]	Height [mAU]	Area %
1	13.963	BB	0.2718	4397.60645	250.57170	68.6822
2	17.228	BB	0.3415	2005.22900	90.52785	31.3178

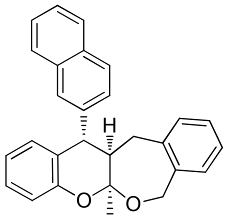

5e

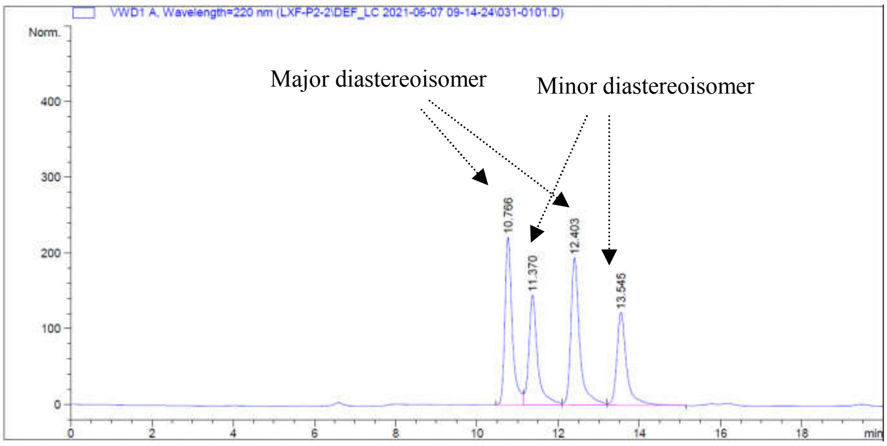

Peak #	RetTime [min]	Type	Width [min]	Area [mAU*s]	Height [mAU]	Area %
1	10.766	BV	0.1866	2768.17822	221.53572	28.1816
2	11.370	VV	0.2191	2181.34009	145.09938	22.2073
3	12.403	MM	0.2431	2806.16748	192.38393	28.5684
4	13.545	VB	0.2491	2066.94678	122.65643	21.0427

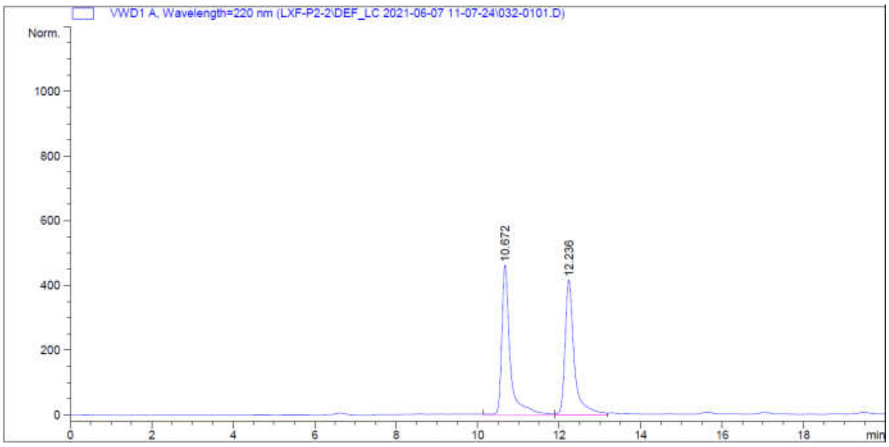

Peak #	RetTime [min]	Type	Width [min]	Area [mAU*s]	Height [mAU]	Area %
1	10.672	VV	0.2100	6664.66455	463.49390	50.2949
2	12.236	VV	0.2320	6586.51270	417.83911	49.7051

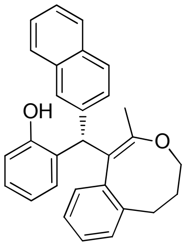

5f

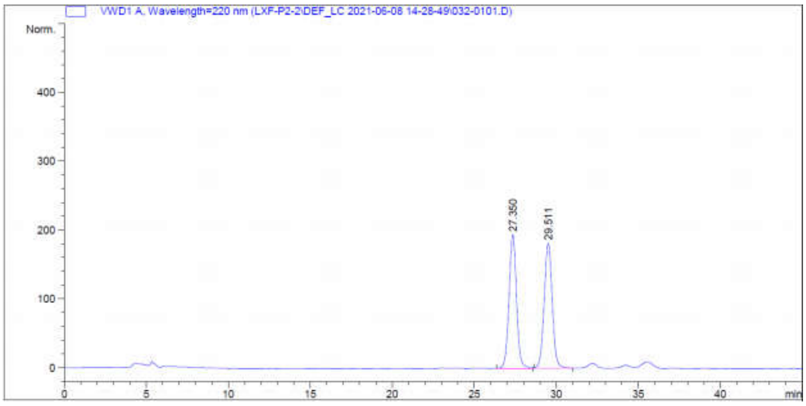

Peak #	RetTime [min]	Type	Width [min]	Area [mAU*s]	Height [mAU]	Area %
1	27.350	BB	0.4875	6168.33447	194.01776	49.8724
2	29.511	BB	0.5268	6199.89160	181.51270	50.1276

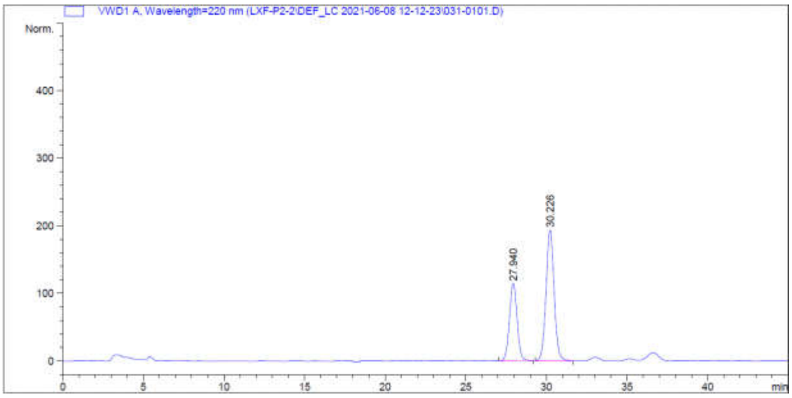

Peak #	RetTime [min]	Type	Width [min]	Area [mAU*s]	Height [mAU]	Area %
1	27.940	BB	0.5181	3812.18945	114.52061	36.0605
2	30.226	BB	0.5392	6759.44971	193.26466	63.9395

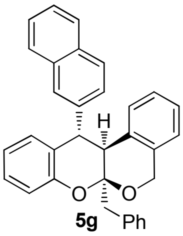

5g

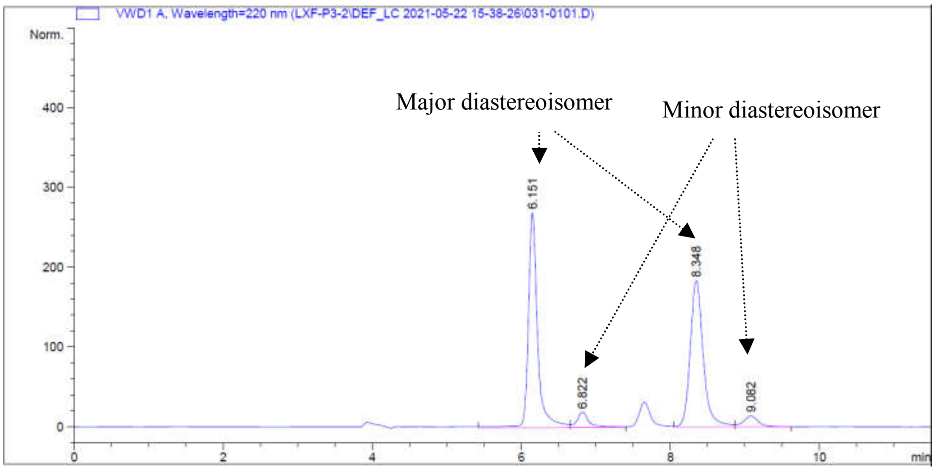

Peak #	RetTime [min]	Type	Width [min]	Area [mAU*s]	Height [mAU]	Area %
1	6.151	VV	0.1281	2318.25098	269.26834	45.8893
2	6.822	VV	0.1663	223.82826	19.24521	4.4306
3	8.348	MM	0.2082	2317.47412	185.50798	45.8739
4	9.082	VB	0.2099	192.28297	13.73788	3.8062

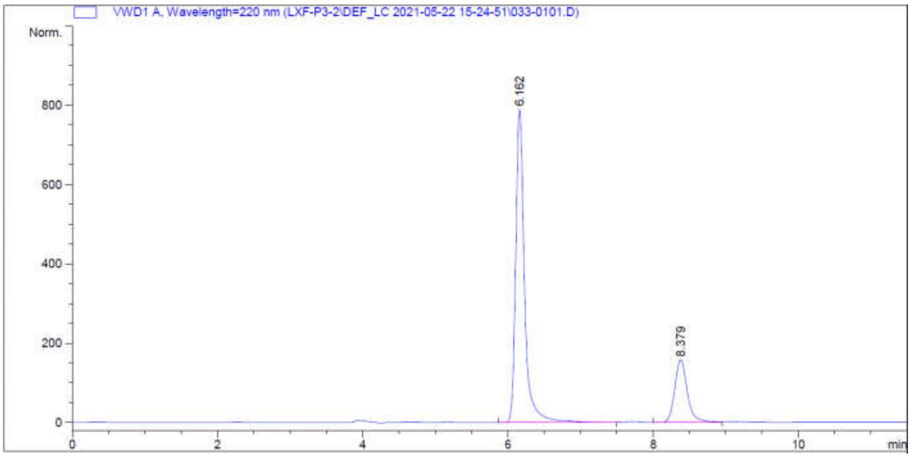

Peak #	RetTime [min]	Type	Width [min]	Area [mAU*s]	Height [mAU]	Area %
1	6.162	BV	0.1261	6654.10596	788.75781	77.6053
2	8.379	VV	0.1838	1920.18530	158.34149	22.3947

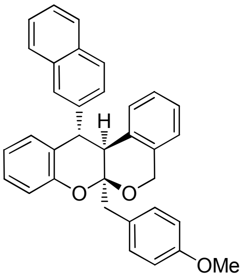

5h

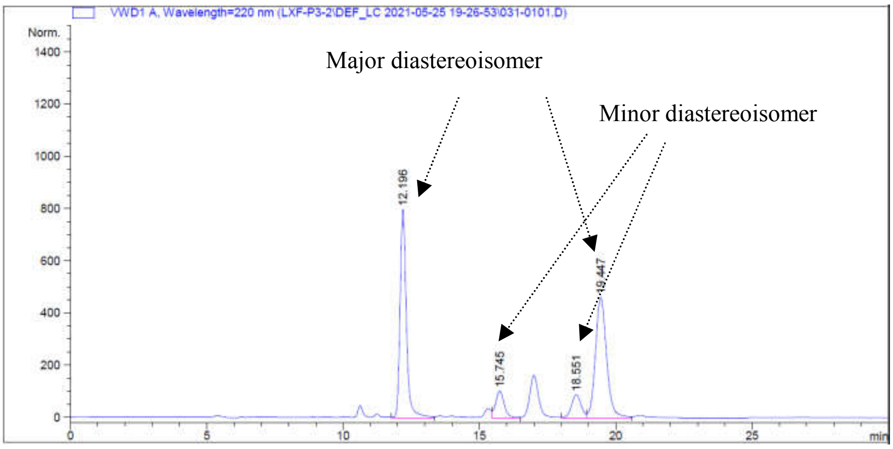

Peak #	RetTime [min]	Type	Width [min]	Area [mAU*s]	Height [mAU]	Area %
1	12.196	VV	0.2441	1.28411e4	800.00159	42.2082
2	15.745	VV	0.3328	2277.13281	103.37984	7.4848
3	18.551	VV	0.3784	2188.06958	89.52778	7.1921
4	19.447	VV	0.4336	1.31170e4	461.49274	43.1150

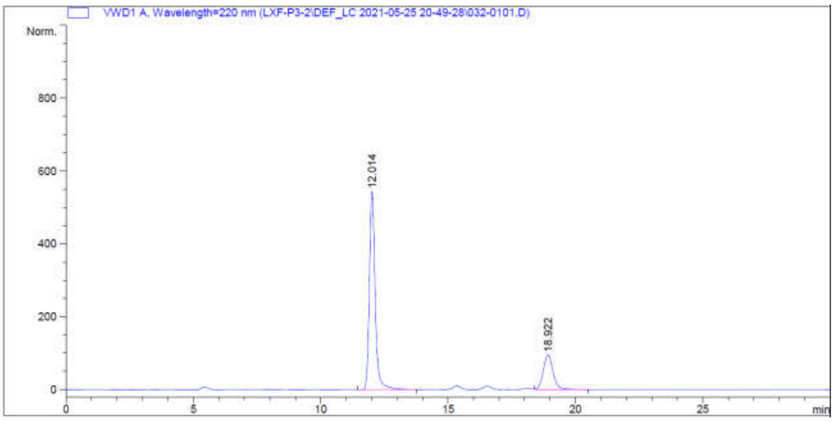

Peak #	RetTime [min]	Type	Width [min]	Area [mAU*s]	Height [mAU]	Area %
1	12.014	VB	0.2373	8553.76563	544.30469	76.6426
2	18.922	VB	0.4151	2606.82080	95.81834	23.3574

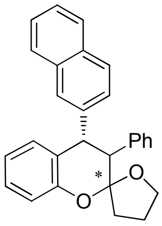

5i

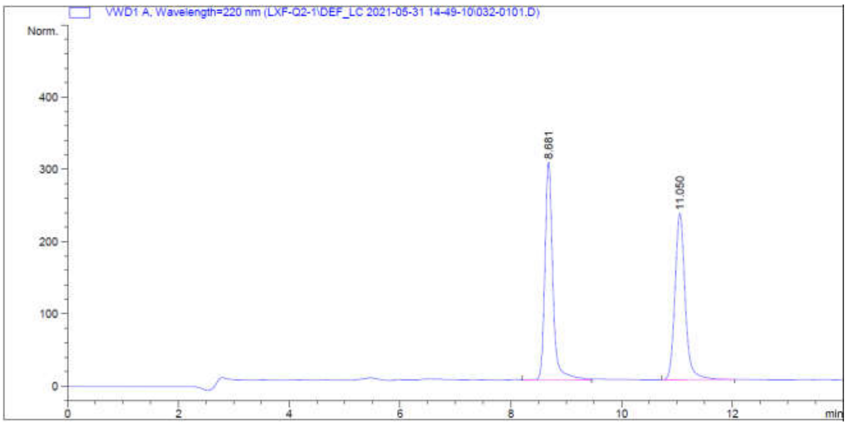

Peak #	RetTime [min]	Type	Width [min]	Area [mAU*s]	Height [mAU]	Area %
1	8.681	VV	0.1516	3009.11206	301.17072	50.5264
2	11.050	BB	0.1945	2946.41626	230.20634	49.4736

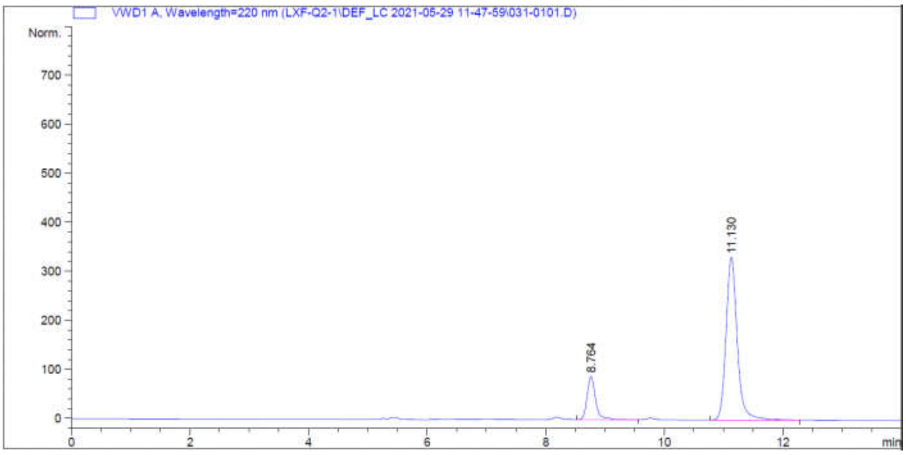

Peak #	RetTime [min]	Type	Width [min]	Area [mAU*s]	Height [mAU]	Area %
1	8.764	BB	0.1532	898.78680	88.69450	17.0972
2	11.130	BB	0.1999	4358.12305	331.87109	82.9028

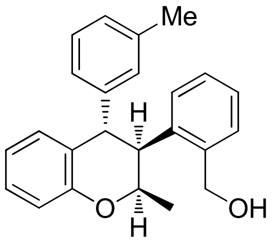

6

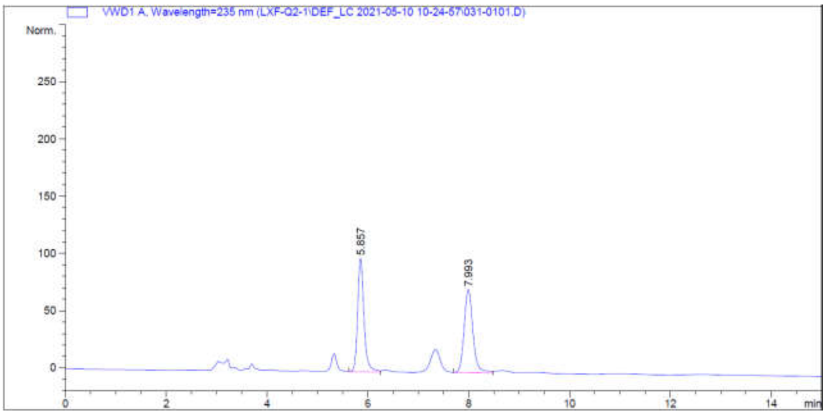

Peak #	RetTime [min]	Type	Width [min]	Area [mAU*s]	Height [mAU]	Area %
1	5.857	VV	0.1326	866.65814	99.10919	49.3528
2	7.993	VV	0.1878	889.38916	72.75034	50.6472

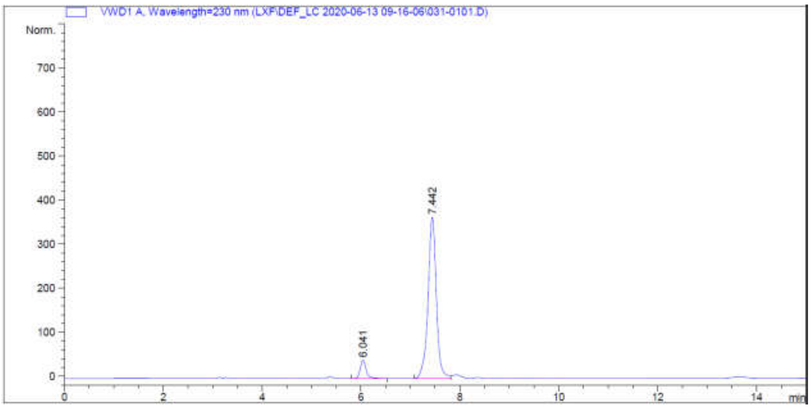

Peak #	RetTime [min]	Type	Width [min]	Area mAU *s	Height [mAU]	Area %
1	6.041	VV	0.1278	341.66910	41.00067	7.4255
2	7.442	VV	0.1765	4259.59766	366.07816	92.5745

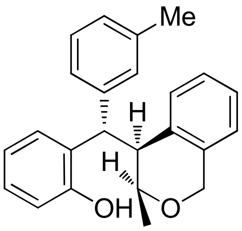

7

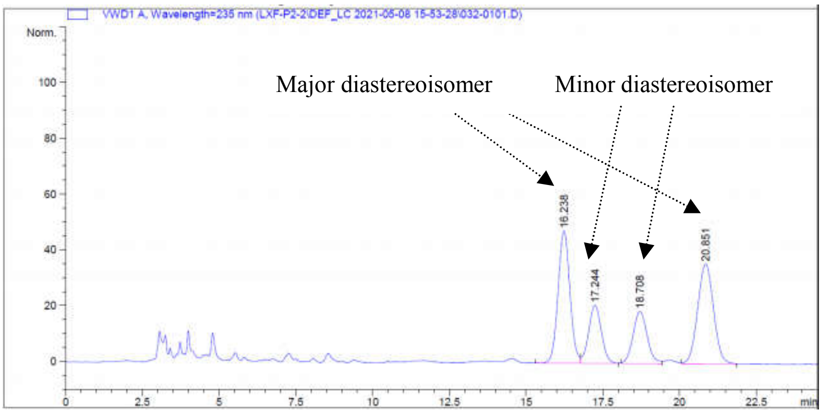

Peak #	RetTime [min]	Type	Width [min]	Area [mAU*s]	Height [mAU]	Area %
1	16.238	BV	0.4174	1275.40430	47.40894	33.9589
2	17.244	VB	0.4500	603.53424	20.84575	16.0697
3	18.708	BV	0.5013	596.26331	18.65038	15.8761
4	20.851	VB	0.5593	1280.52576	35.74601	34.0953

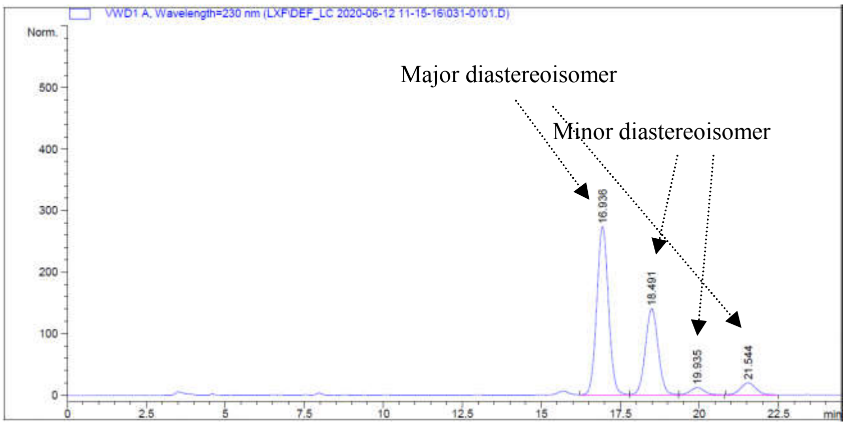

Peak #	RetTime [min]	Type	Width [min]	Area mAU *s	Height [mAU]	Area %
1	16.936	VV	0.3988	7024.20068	273.34683	58.5233
2	18.491	VV	0.4399	3960.47168	140.33821	32.9974
3	19.935	VV	0.4806	366.09647	11.82346	3.0502
4	21.544	VB	0.5215	651.62256	19.47241	5.4291

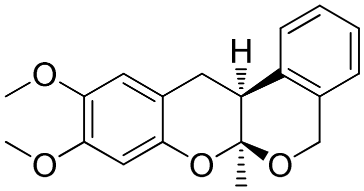

9a

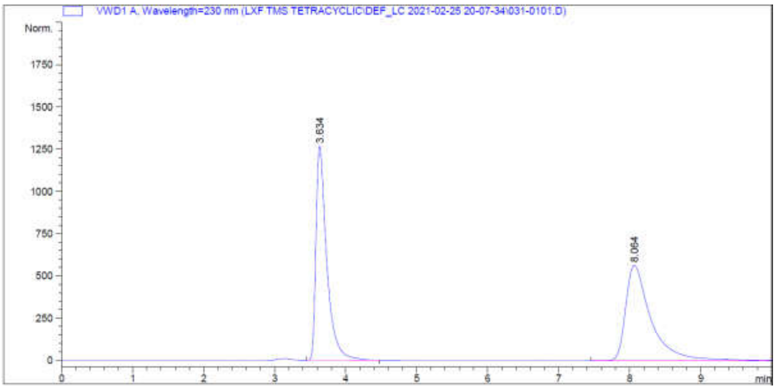

Peak #	RetTime [min]	Type	Width [min]	Area [mAU*s]	Height [mAU]	Area %
1	3.637	VV	0.1603	1.35074e4	1257.19385	49.4726
2	8.066	VV	0.3561	1.37954e4	562.58008	50.5274

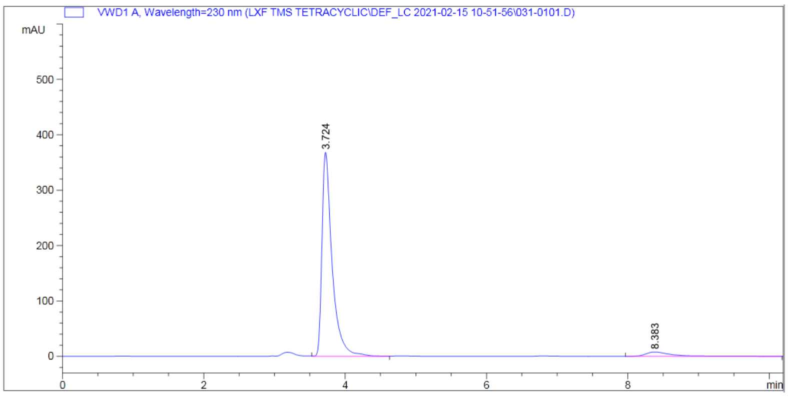

Peak #	RetTime [min]	Type	Width [min]	Area mAU *s	Height [mAU]	Area %
1	3.724	VV	0.1518	3688.82129	368.40384	94.9954
2	8.383	BB	0.3669	194.33629	7.78684	5.0046

9. HRMS (ESI) results

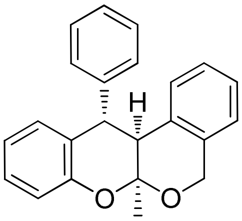

4a

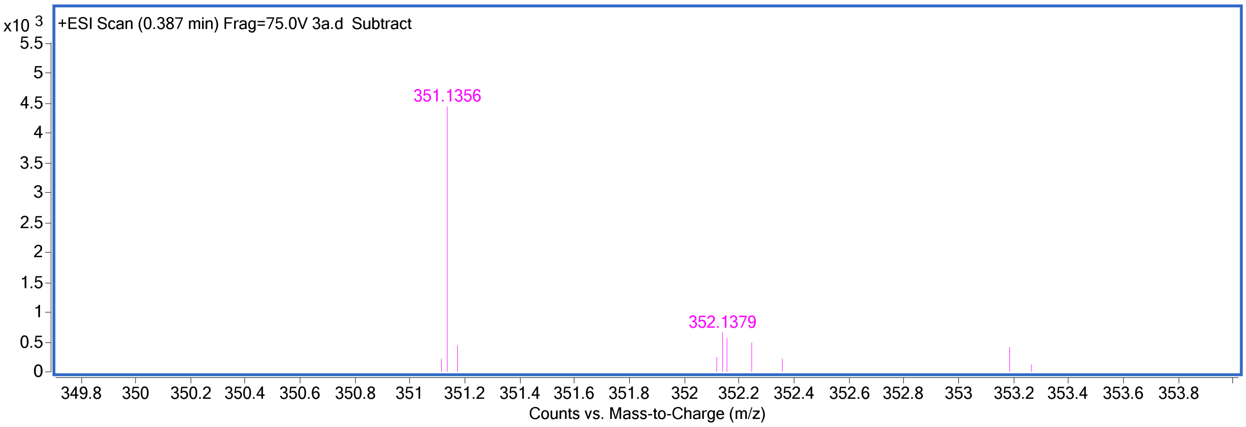

HRMS (ESI) m/z calcd for $C_{23}H_{20}O_2$, $[M+Na]^+$: 351.1356

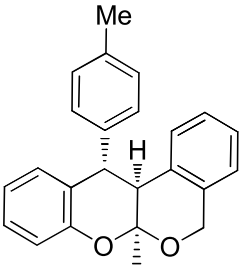

4b

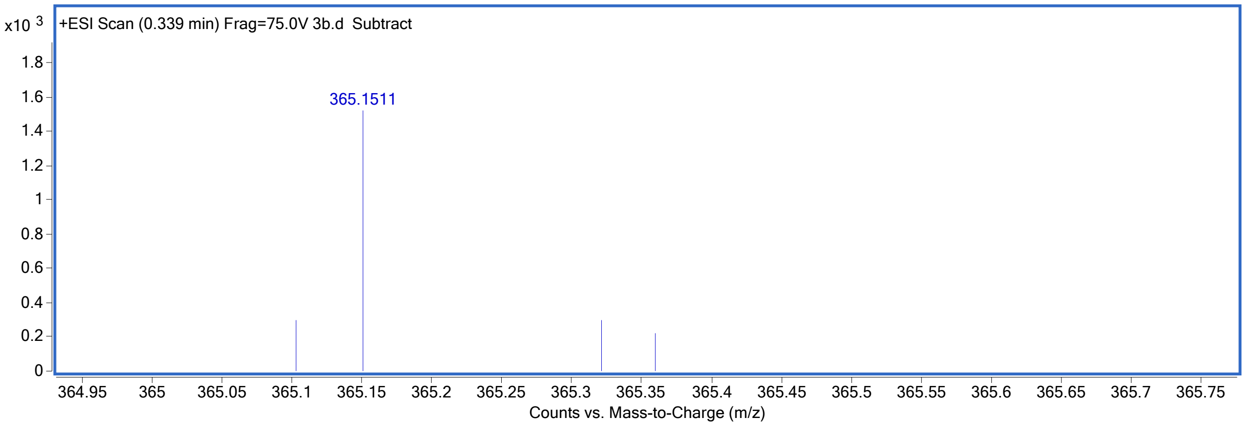

HRMS (ESI) m/z calcd for $C_{24}H_{22}O_2$, $[M+Na]^+$: 365.1512

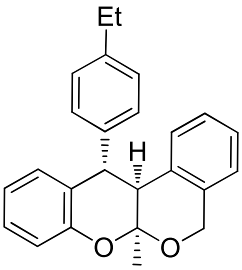

4c

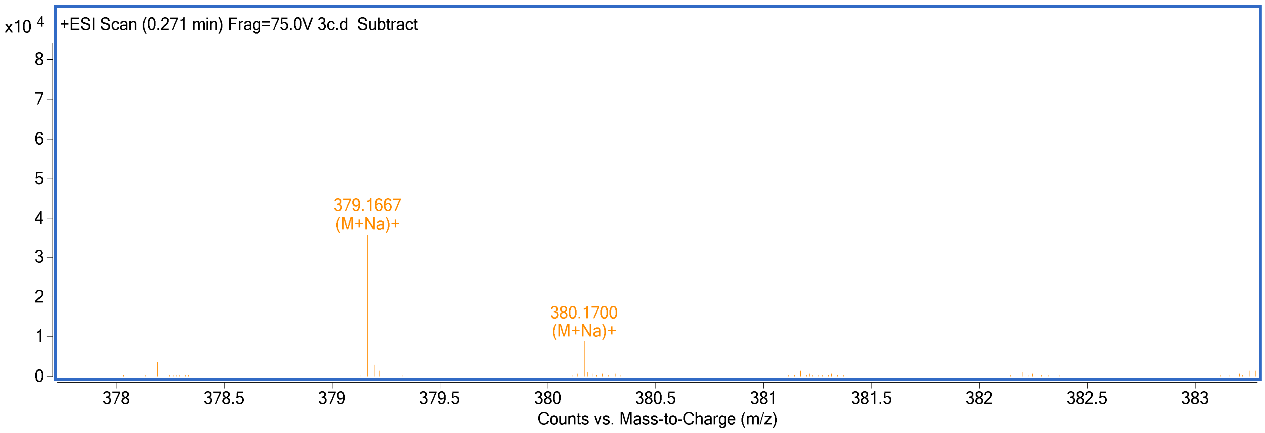

HRMS (ESI) m/z calcd for $C_{25}H_{24}O_2$, $[M+Na]^+$: 379.1669

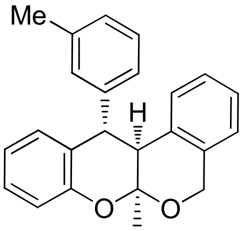

4d

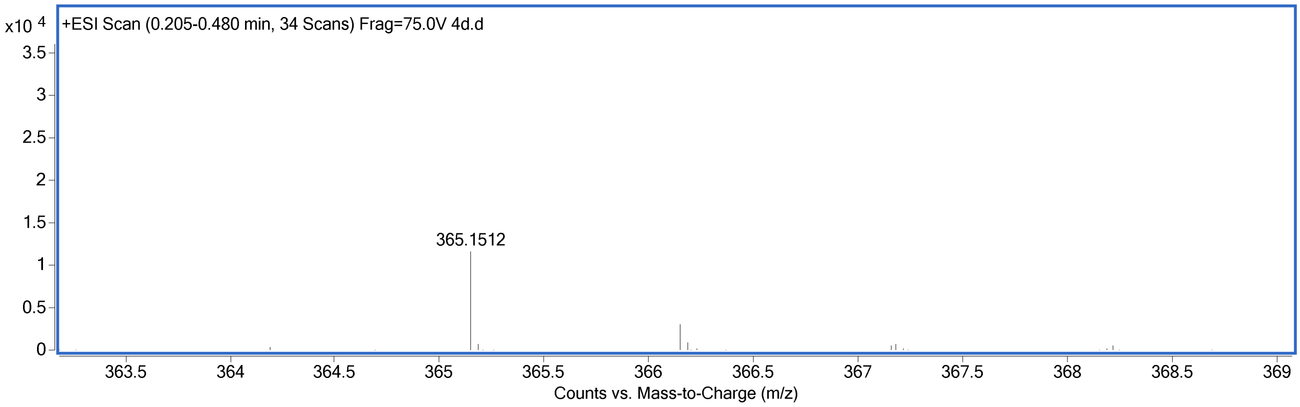

HRMS (ESI) *m/z* calcd for $C_{24}H_{22}O_2$, $[M+Na]^+$: 365.1512

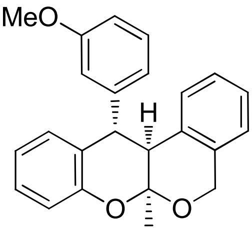

4e

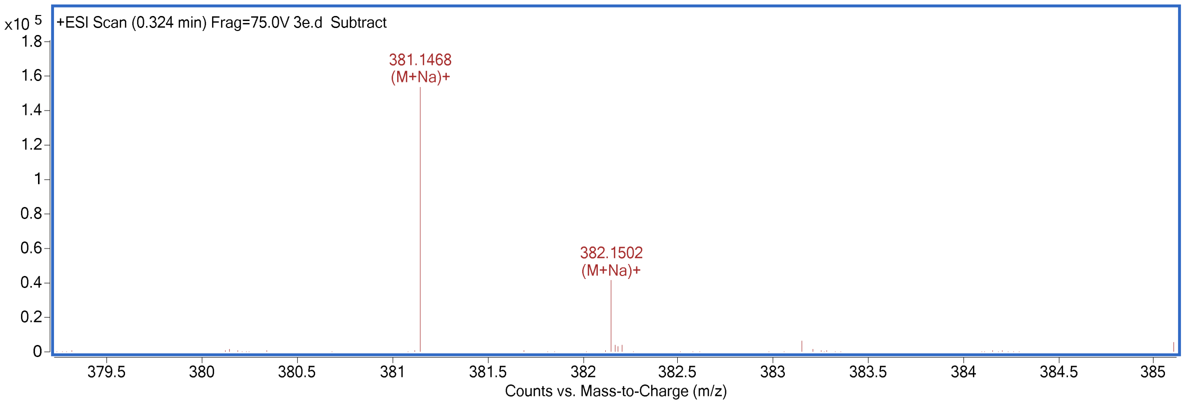

HRMS (ESI) *m/z* calcd for $C_{24}H_{22}O_3$, $[M+Na]^+$: 381.1461

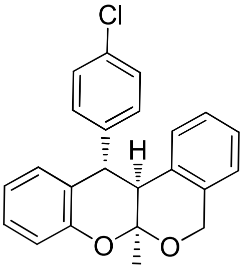

4f

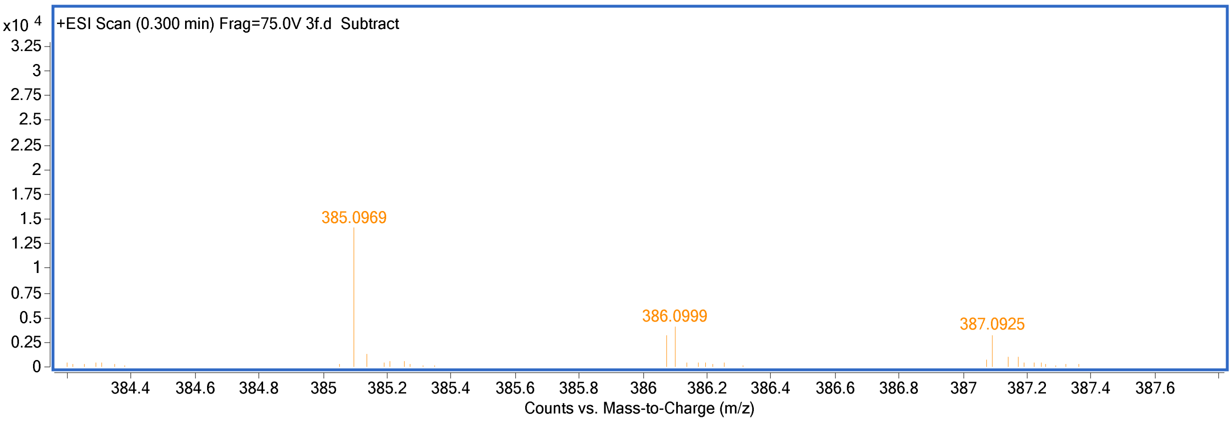

HRMS (ESI) *m/z* calcd for $C_{23}H_{19}O_2Cl$, $[M+Na]^+$: 385.0966

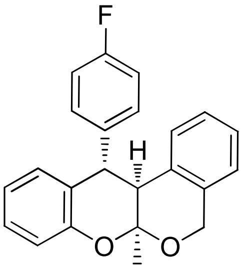

4g

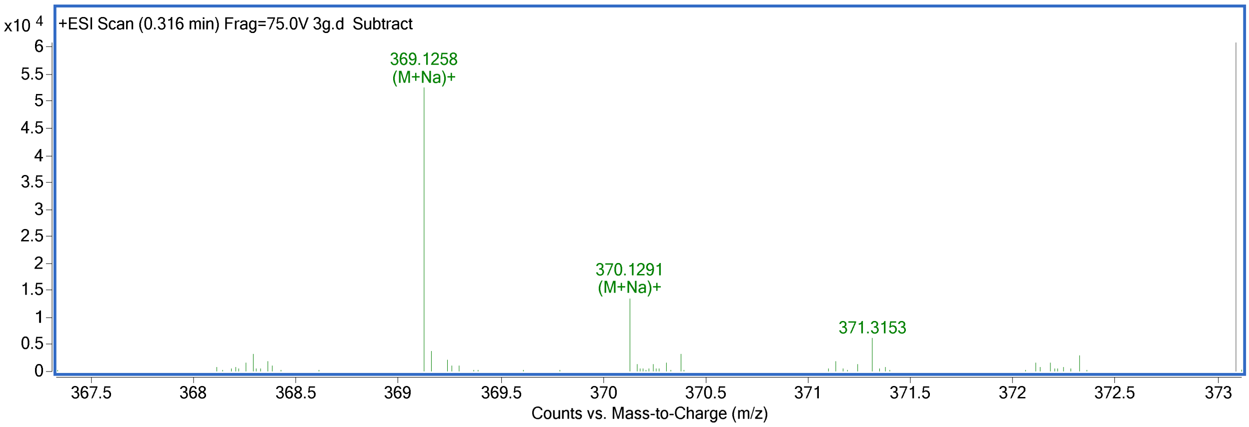

HRMS (ESI) *m/z* calcd for $C_{23}H_{19}O_2F$, $[M+Na]^+$: 369.1261

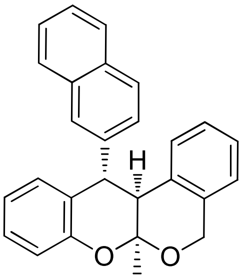

4h

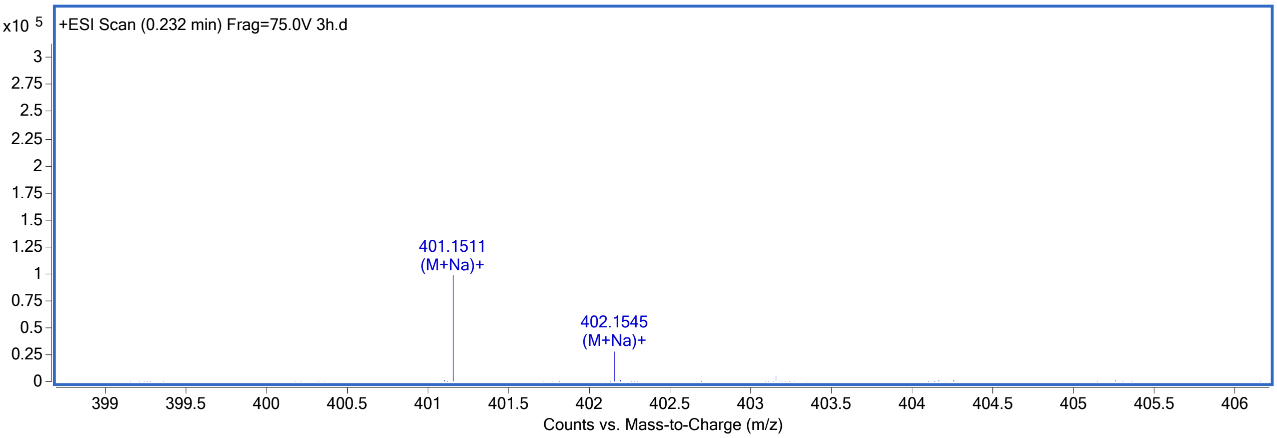

HRMS (ESI) *m/z* calcd for $C_{27}H_{22}O_2$, $[M+Na]^+$: 401.1512

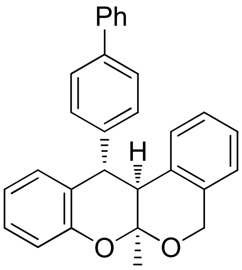

4i

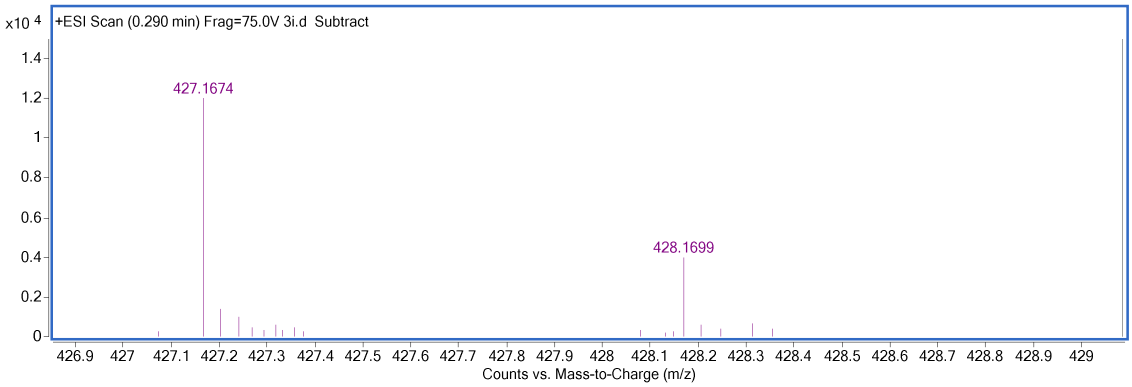

HRMS (ESI) *m/z* calcd for $C_{27}H_{22}O_2$, $[M+Na]^+$: 427.1669

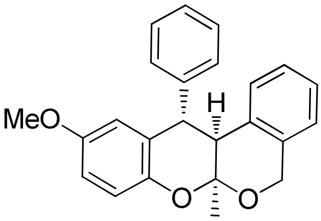

4j

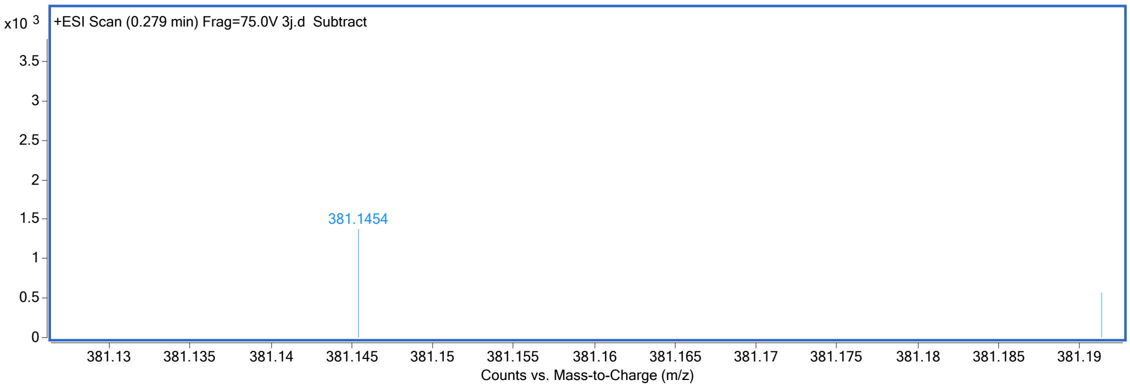

HRMS (ESI) *m/z* calcd for $C_{24}H_{22}O_3$, $[M+Na]^+$: 381.1461

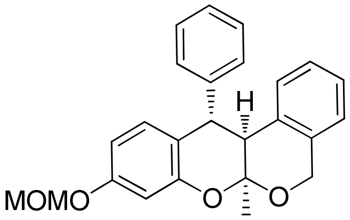

4k

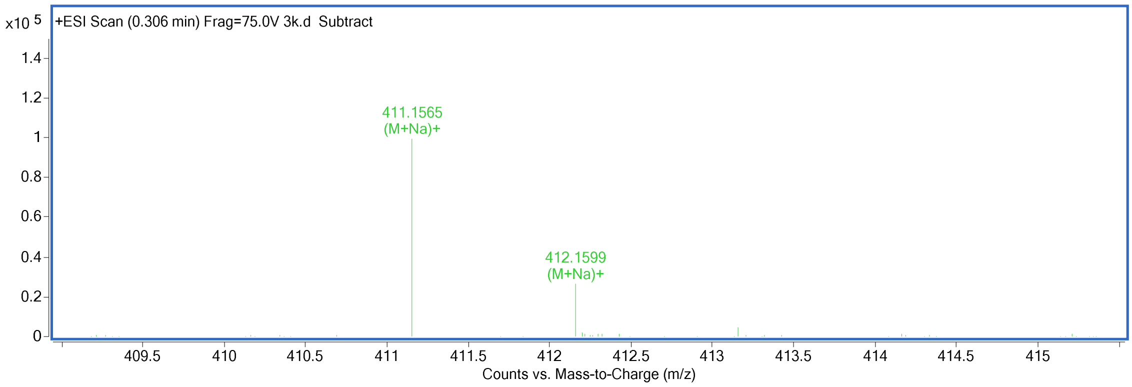

HRMS (ESI) *m/z* calcd for $C_{25}H_{24}O_4$, $[M+Na]^+$: 411.1567

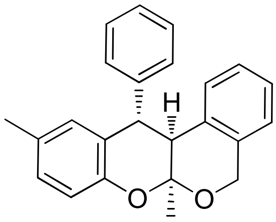

4l

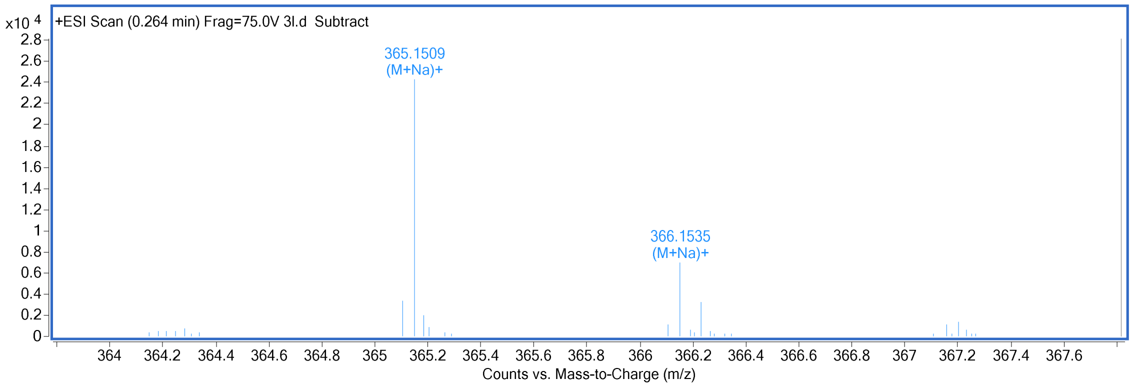

HRMS (ESI) *m/z* calcd for $C_{24}H_{22}O_2$, $[M+Na]^+$: 365.1512

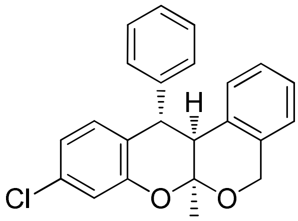

4m

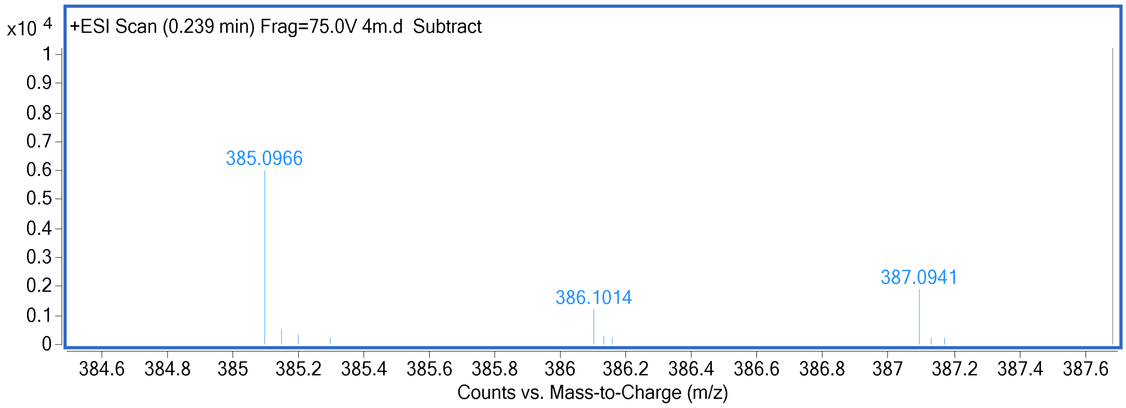

HRMS (ESI) *m/z* calcd for $C_{23}H_{19}O_2Cl$, $[M+Na]^+$: 385.0966

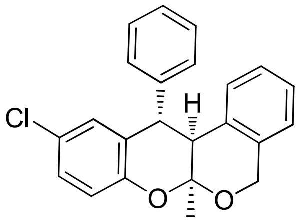

4n

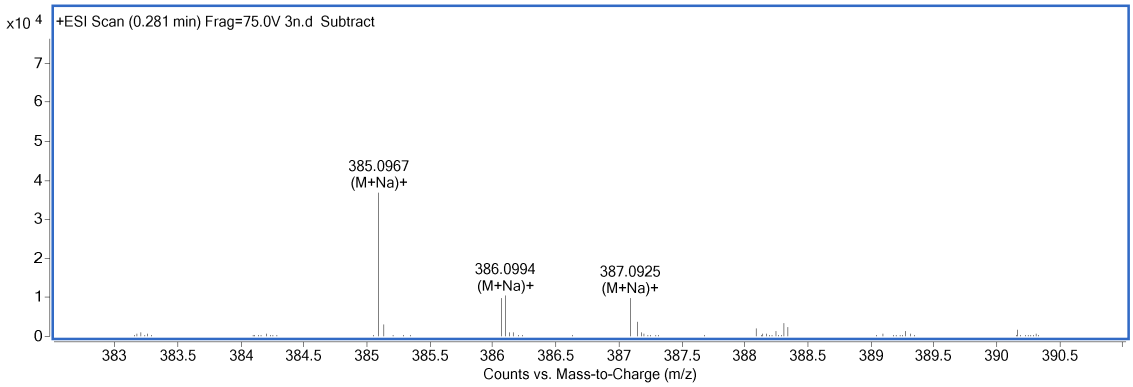

HRMS (ESI) *m/z* calcd for $C_{23}H_{19}O_2Cl$, $[M+Na]^+$: 385.0966

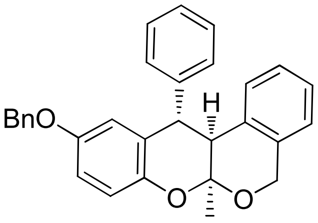

4o

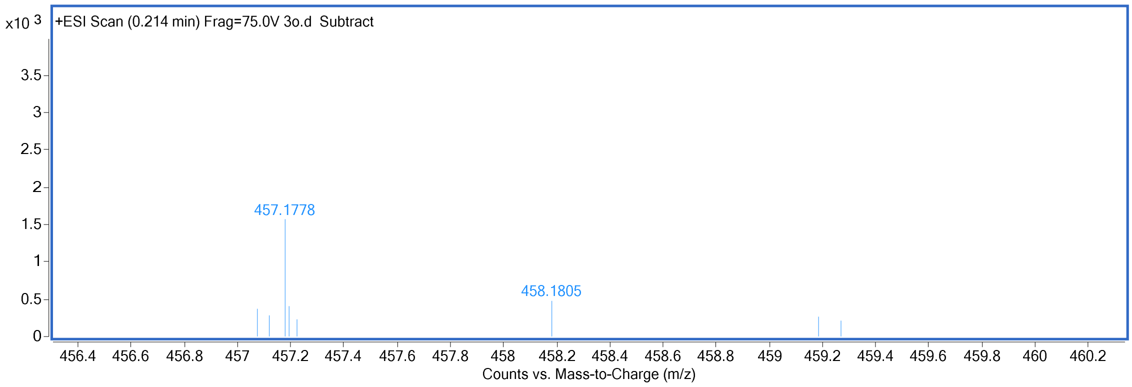

HRMS (ESI) *m/z* calcd for $C_{30}H_{26}O_3$, $[M+Na]^+$: 457.1774

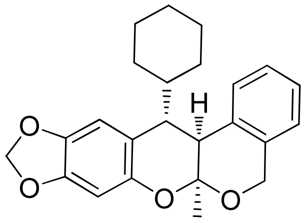

4p

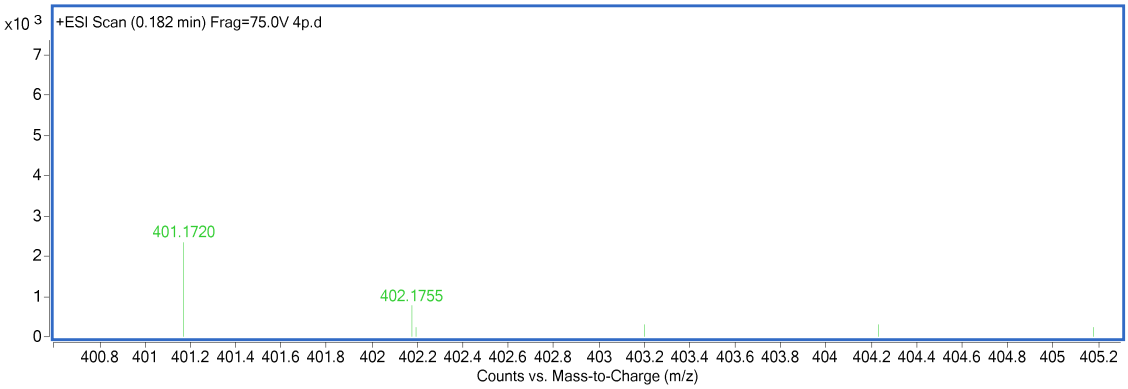

HRMS (ESI) *m/z* calcd for $C_{24}H_{26}O_4$, $[M+Na]^+$: 401.1723

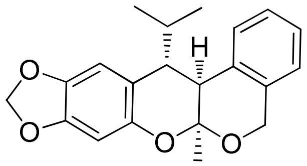

4q

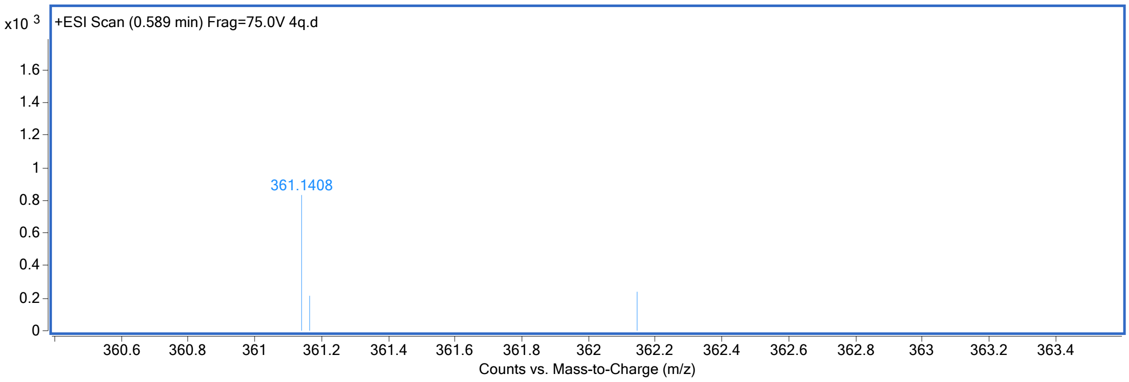

HRMS (ESI) *m/z* calcd for $C_{21}H_{22}O_4$, $[M+Na]^+$: 361.1410

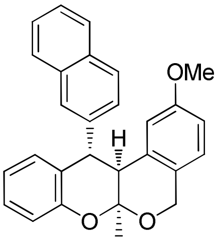

4r

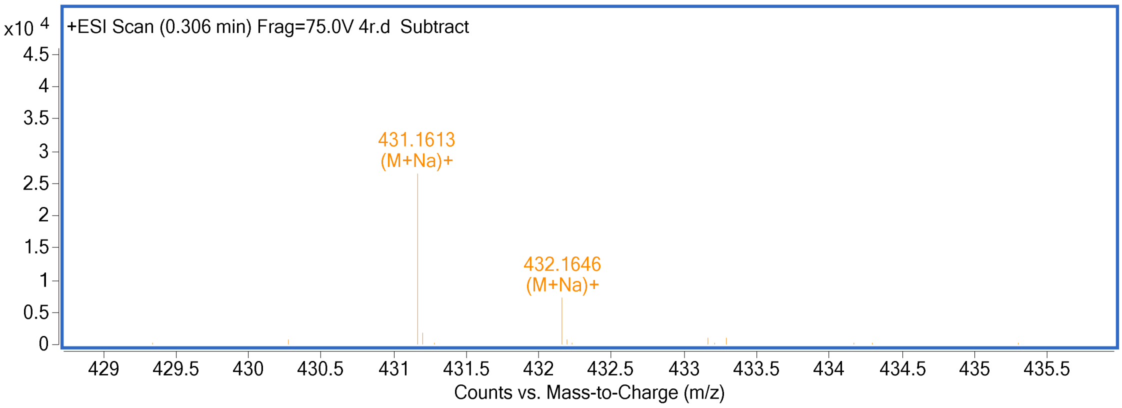

HRMS (ESI) *m/z* calcd for $C_{28}H_{24}O_3$, $[M+Na]^+$: 431.1618

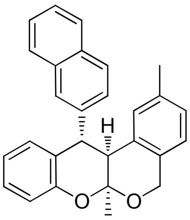

4s

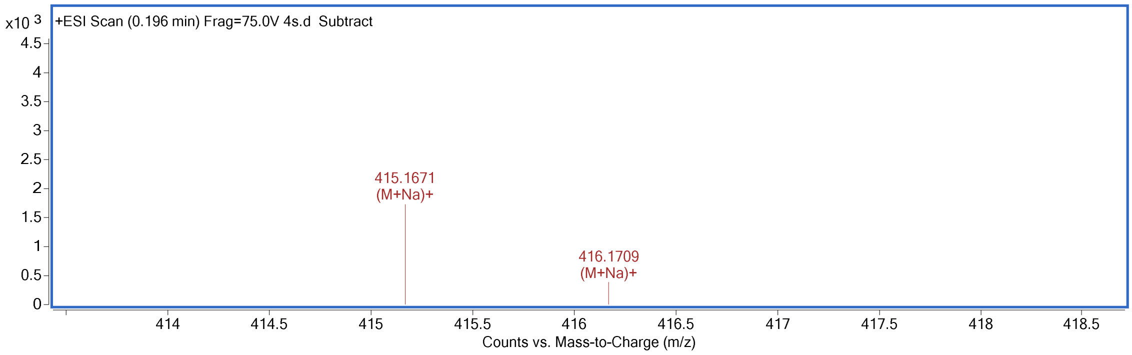

HRMS (ESI) *m/z* calcd for $C_{28}H_{24}O_2$, $[M+Na]^+$: 415.1669

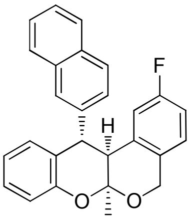

4t

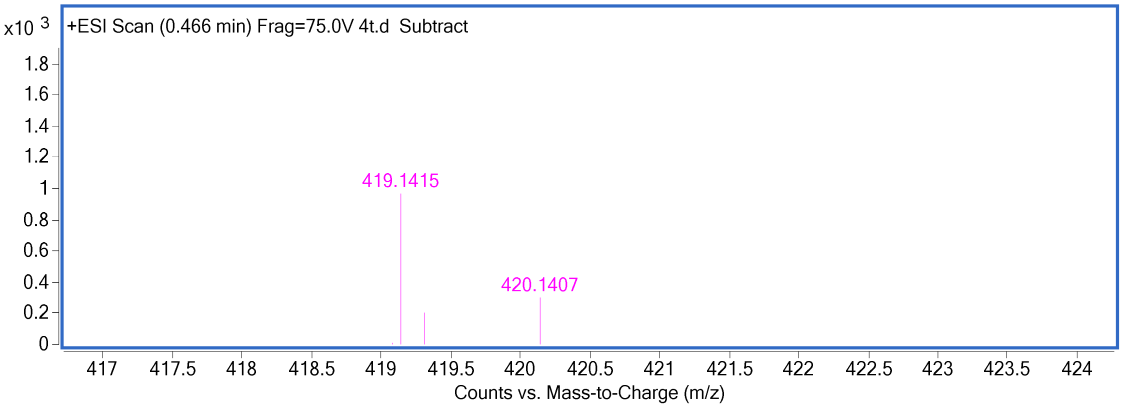

HRMS (ESI) *m/z* calcd for $C_{27}H_{21}O_2F$, $[M+Na]^+$: 419.1418

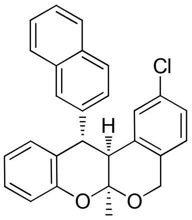

4u

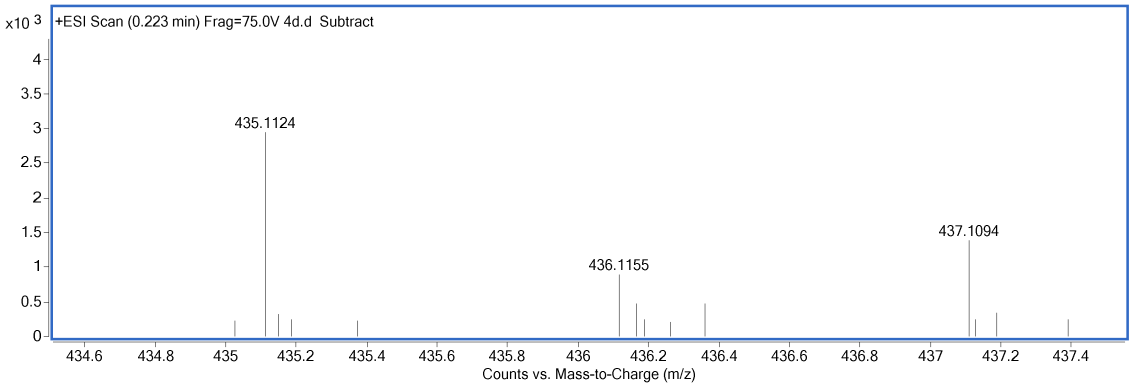

HRMS (ESI) *m/z* calcd for $C_{27}H_{21}O_2Cl$, $[M+Na]^+$: 435.1122

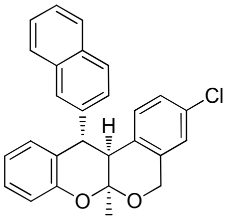

4v

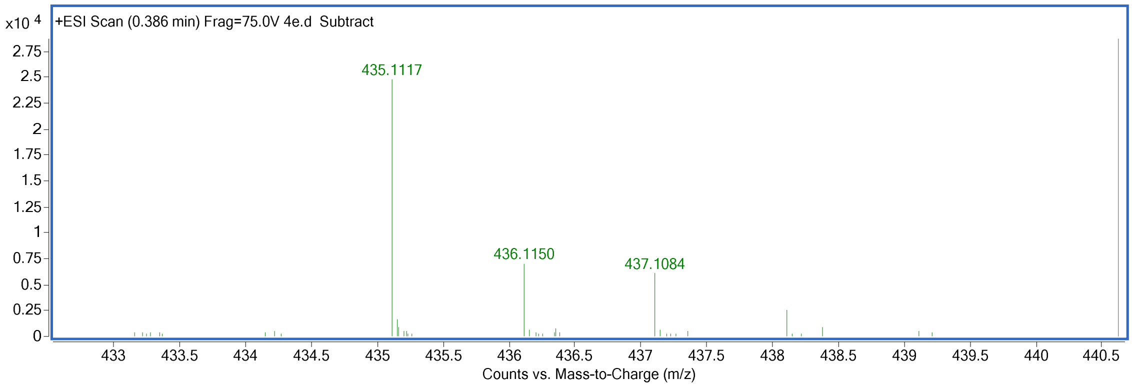

HRMS (ESI) *m/z* calcd for $C_{27}H_{21}O_2Cl$, $[M+Na]^+$: 435.1122

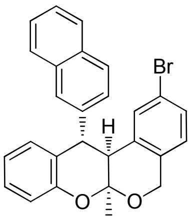

4w

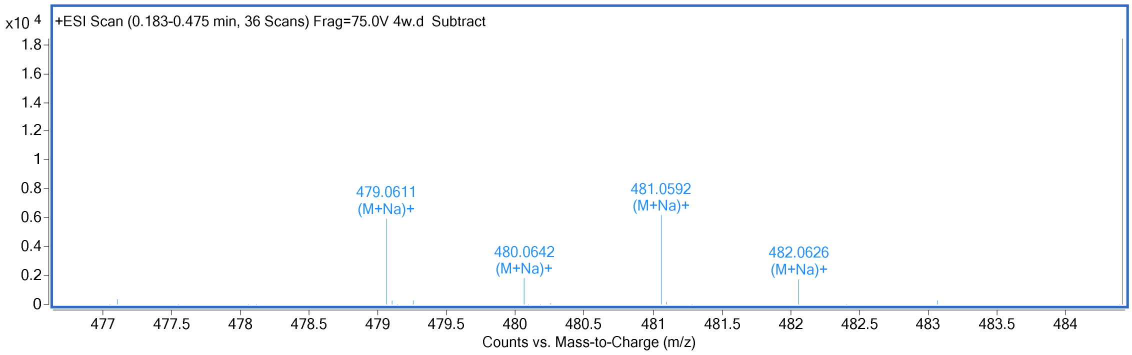

HRMS (ESI) *m/z* calcd for $C_{27}H_{21}O_2Br$, $[M+Na]^+$: 479.0617

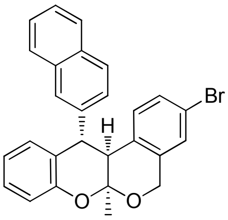

4x

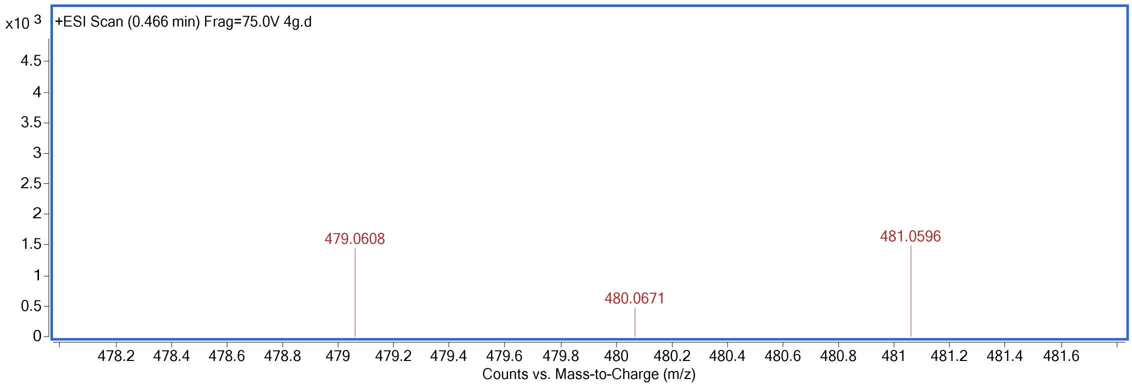

HRMS (ESI) *m/z* calcd for $C_{27}H_{21}O_2Br$, $[M+Na]^+$: 479.0617

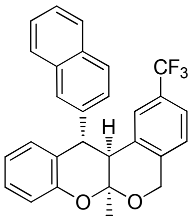

4y

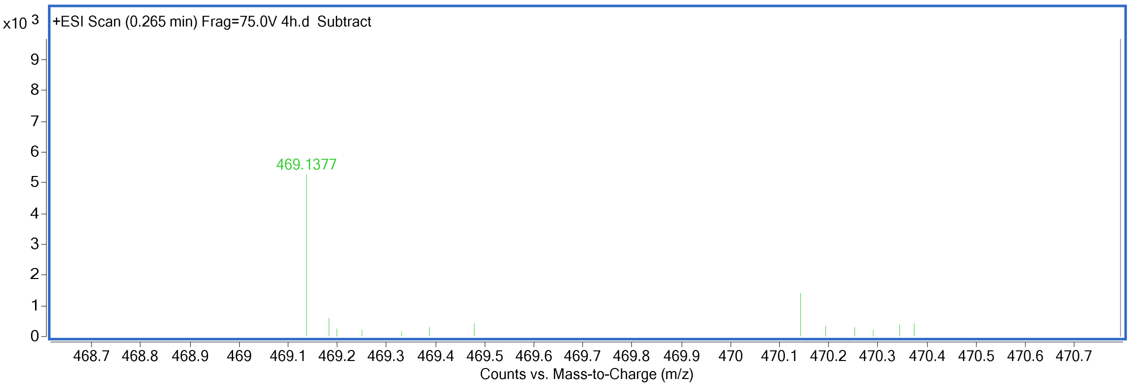

HRMS (ESI) *m/z* calcd for $C_{28}H_{21}O_2F_3$, $[M+Na]^+$: 469.1386

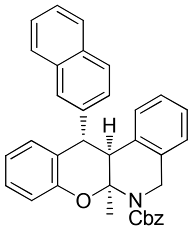

5a

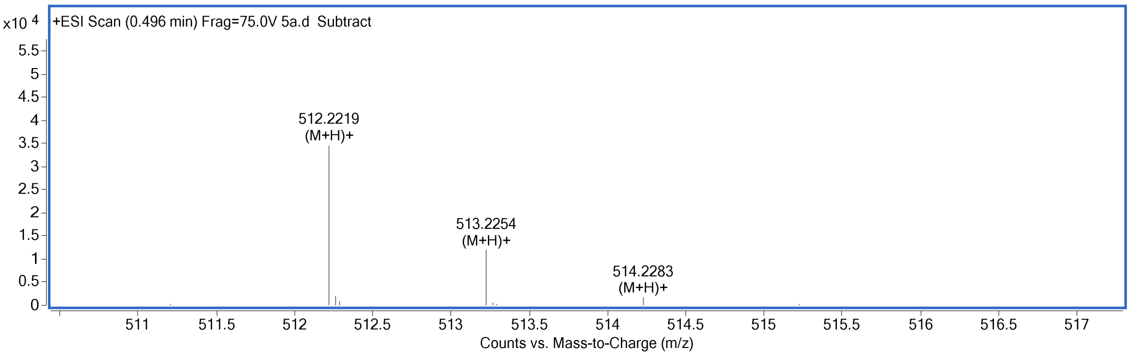

HRMS (ESI) *m/z* calcd for $C_{35}H_{29}NO_3$, $[M+H]^+$: 512.2220

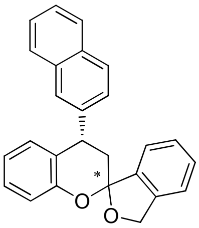

5b

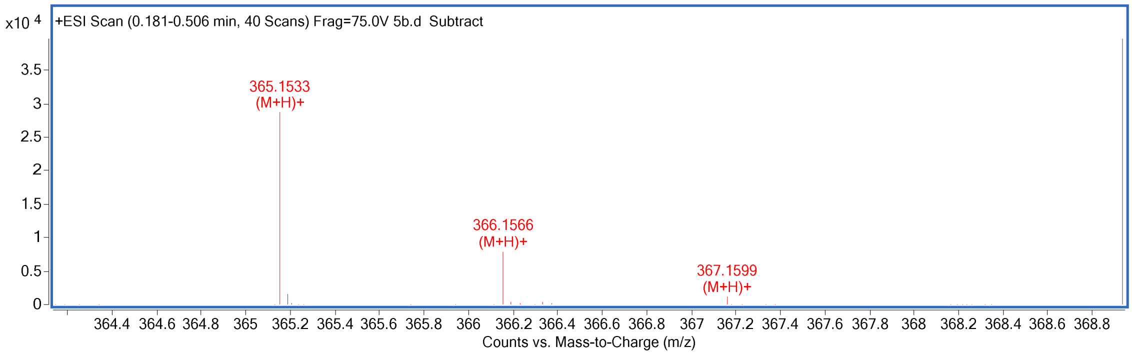

HRMS (ESI) *m/z* calcd for $C_{26}H_{20}O_2$, $[M+Na]^+$: 367.1536

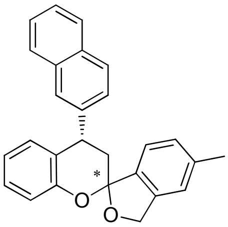

5c

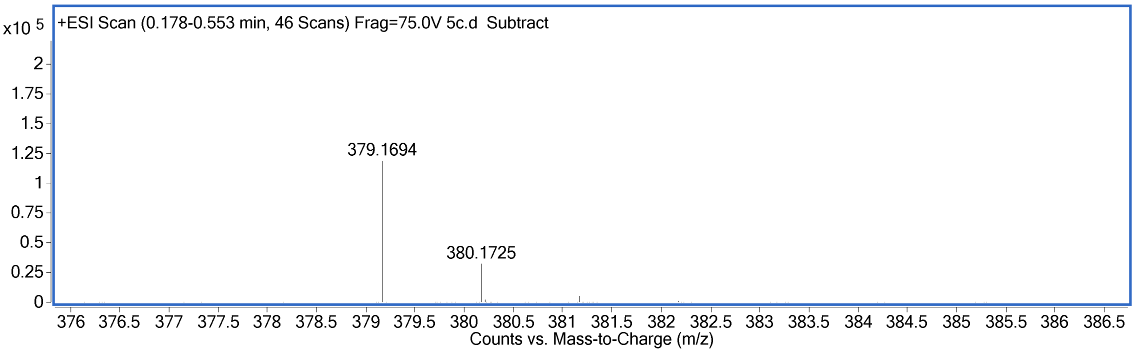

HRMS (ESI) *m/z* calcd for $C_{27}H_{22}O_2$, $[M+H]^+$: 379.1693

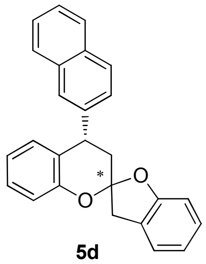

5d

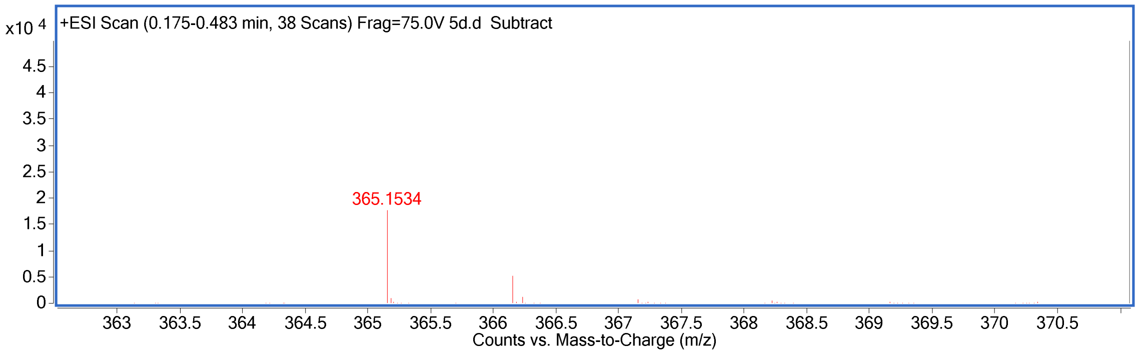

HRMS (ESI) *m/z* calcd for $C_{26}H_{20}O_2$, $[M+H]^+$: 365.1536

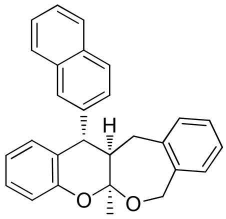

5e

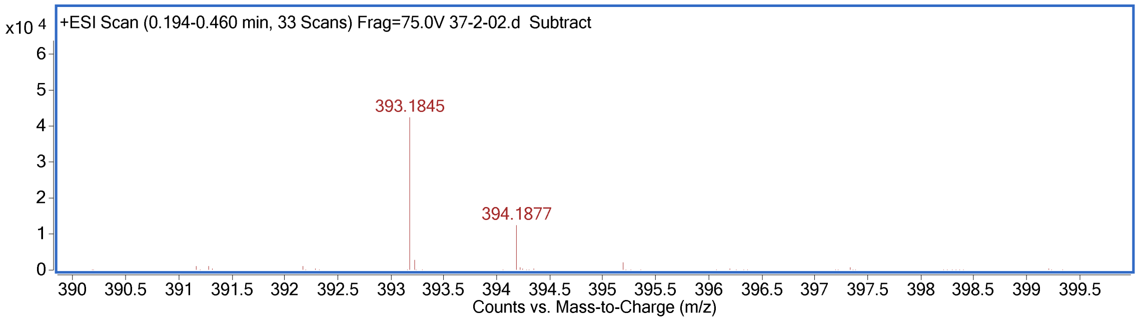

HRMS (ESI) *m/z* calcd for $C_{28}H_{24}O_2$, $[M+H]^+$: 393.1849

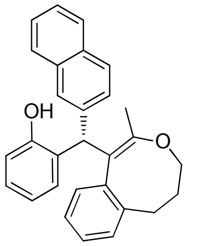

5f

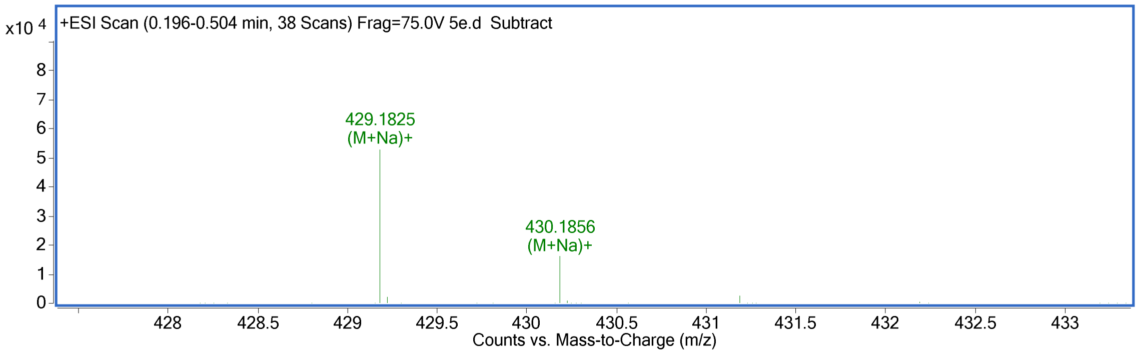

HRMS (ESI) *m/z* calcd for $C_{29}H_{26}O_2$, $[M+Na]^+$: 429.1825

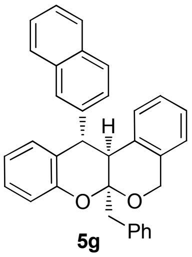

5g

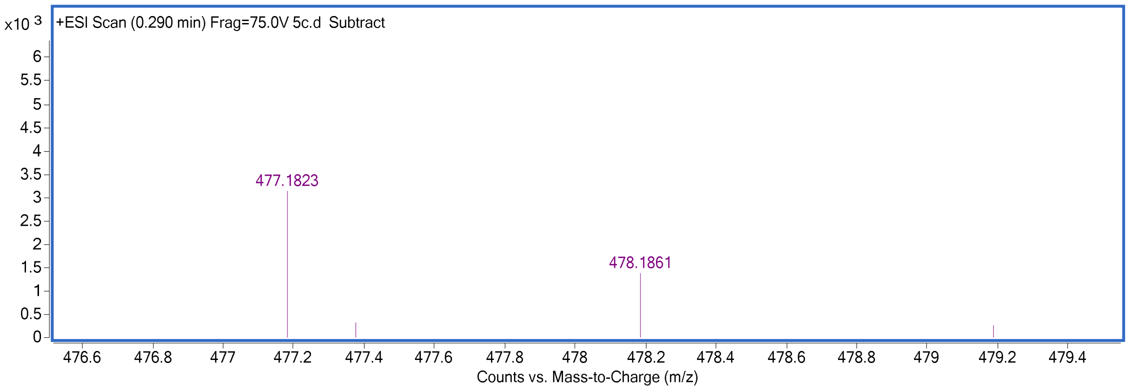

HRMS (ESI) *m/z* calcd for $C_{33}H_{26}O_2$, $[M+Na]^+$: 477.1825

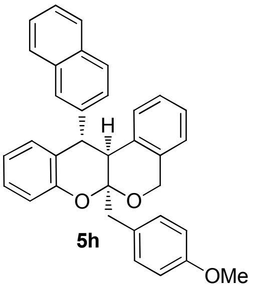

5h

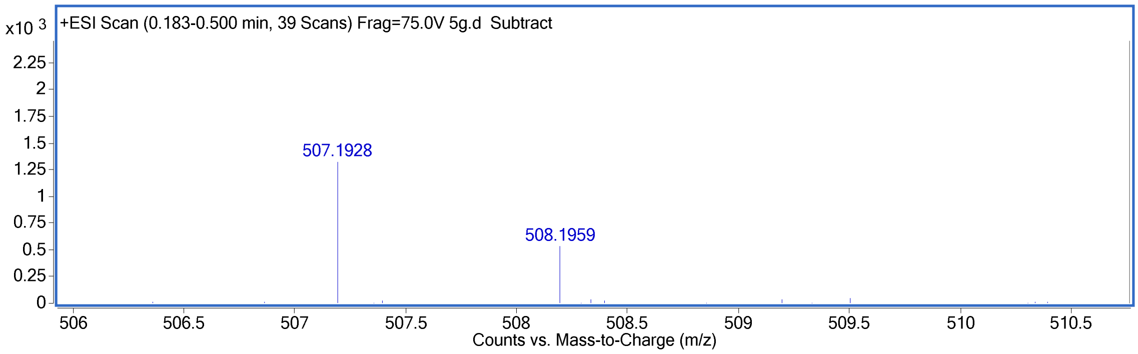

HRMS (ESI) *m/z* calcd for $C_{34}H_{28}O_3$, $[M+Na]^+$: 507.1931

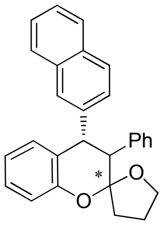

5i

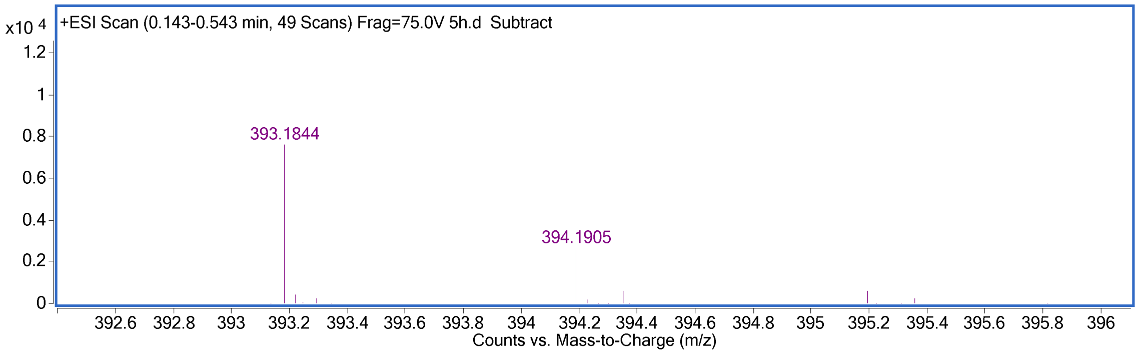

HRMS (ESI) *m/z* calcd for $C_{28}H_{24}O_2$, $[M+H]^+$: 393.1849

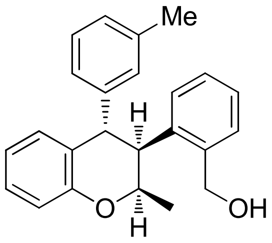

6

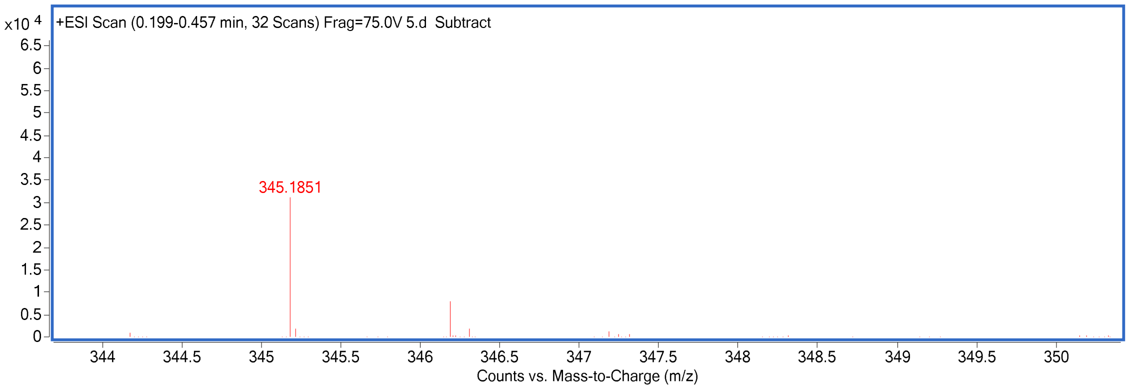

HRMS (ESI) *m/z* calcd for $C_{24}H_{24}O_2$, $[M+H]^+$: 345.1849

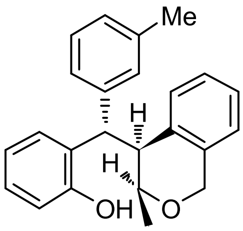

7

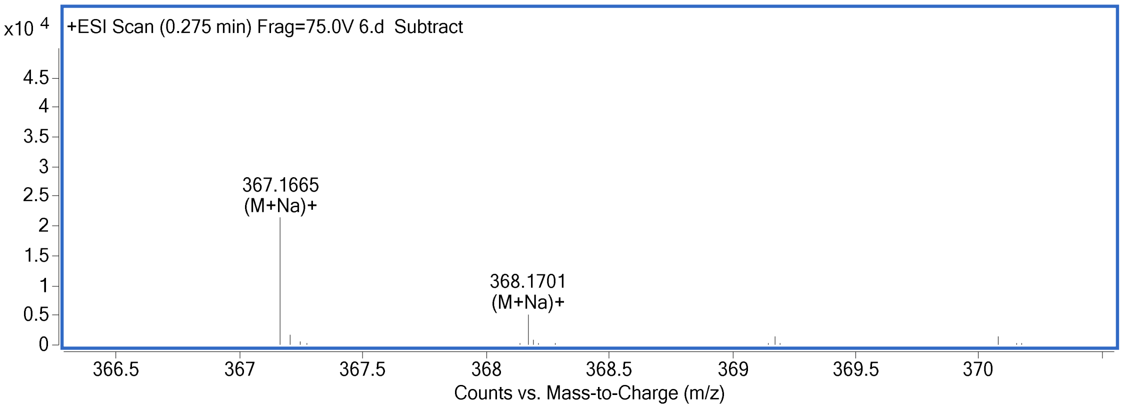

HRMS (ESI) *m/z* calcd for $C_{24}H_{24}O_2$, $[M+Na]^+$: 367.1669

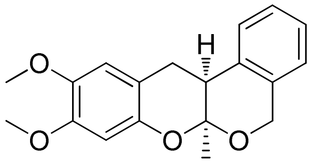

9a

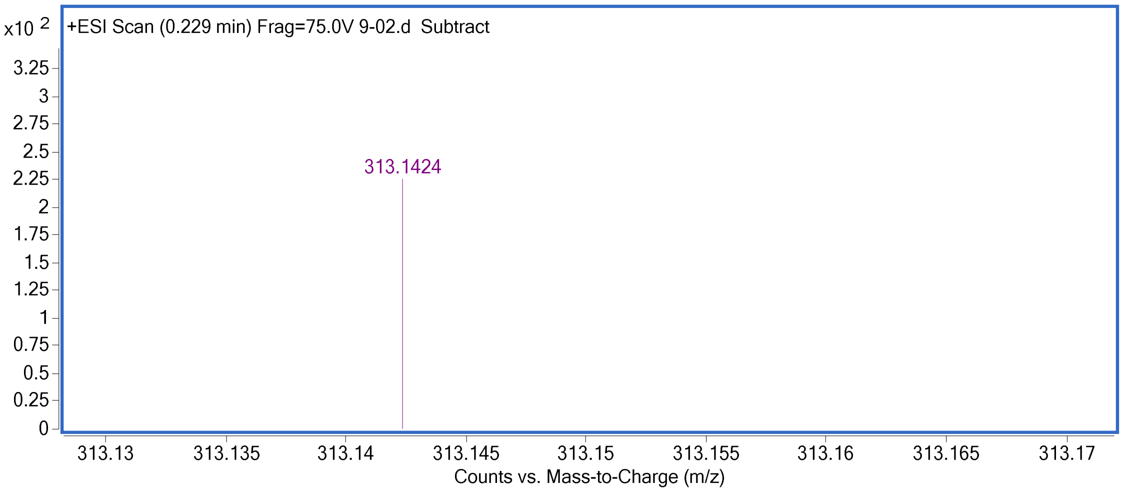

HRMS (ESI) *m/z* calcd for $C_{19}H_{20}O_4$, $[M+H]^+$: 313.1434

Supplementary References:

[1] Hsiao, C. C., Liao, H. H. & Rueping, M. Enantio- and diastereoselective access to distant stereocenters embedded within tetrahydroxanthenes: utilizing *ortho*-quinone methides as reactive intermediates in asymmetric Brønsted acid catalysis. *Angew. Chem. Int. Ed.* **53**, 13258-13263 (2014).

[2] Chang, S., Lee, M., Jung, D. Y., Yoo, E. J., Cho, S. H., & Han, S. K. Catalytic One-Pot Synthesis of Cyclic Amidines by Virtue of Tandem Reactions Involving Intramolecular Hydroamination under Mild Conditions. *J. Am. Chem. Soc.* **128**, 12366-12367 (2006).

[3] Patil, N. T., Lutete, L. M., Wu, H., Pahadi, N. K., Gridnev, I. D., & Yamamoto, Y. Palladium-Catalyzed Intramolecular Asymmetric Hydroamination, Hydroalkoxylation, and Hydrocarbonation of Alkynes. *J. Org. Chem.*, **71**, 4270-4279 (2006).

[4] Bartelson, K. J., Singh, R. P., Foxman, B. M. & Deng, L. Catalytic asymmetric 4+2 additions with aliphatic nitroalkenes. *Chem. Sci.* **2**, 1940-1944 (2011).

[5] Singleton, D. A. & Thomas, A. A. High-precision simultaneous determination of multiple small kinetic isotope effects at natural abundance. *J. Am. Chem. Soc.* **117**, 9357-9358 (1995).
